# Nickel-catalyzed C–H alkylation of indoles with unactivated alkyl chlorides: evidence of a Ni(i)/Ni(iii) pathway†
†Electronic supplementary information (ESI) available: Full experimental procedures and characterization data, including ^1^H and ^13^C NMR of all compounds. See DOI: 10.1039/c9sc01446b


**DOI:** 10.1039/c9sc01446b

**Published:** 2019-08-19

**Authors:** Dilip K. Pandey, Shidheshwar B. Ankade, Abad Ali, C. P. Vinod, Benudhar Punji

**Affiliations:** a Organometallic Synthesis and Catalysis Group , Chemical Engineering Division , CSIR–National Chemical Laboratory (CSIR–NCL) , Dr. Homi Bhabha Road , Pune 411 008 , Maharashtra , India . Email: b.punji@ncl.res.in; b Academy of Scientific and Innovative Research (AcSIR) , CSIR–NCL , Dr. Homi Bhabha Road , Pune , India; c Catalysis Division , CSIR–NCL , Dr. Homi Bhabha Road , Pune , India

## Abstract

A mild and efficient nickel-catalyzed method for the coupling of unactivated primary and secondary alkyl chlorides with the C–H bond of indoles and pyrroles is described which demonstrates a high level of chemo and regioselectivity. The reaction tolerates numerous functionalities, such as halide, alkenyl, alkynyl, ether, thioether, furanyl, pyrrolyl, indolyl and carbazolyl groups including acyclic and cyclic alkyls under the reaction conditions. Mechanistic investigation highlights that the alkylation proceeds through a single-electron transfer (SET) process with Ni(i)-species being the active catalyst. Overall, the alkylation follows a Ni(i)/Ni(iii) pathway involving the rate-influencing two-step single-electron oxidative addition of alkyl chlorides.

## Abstract 2

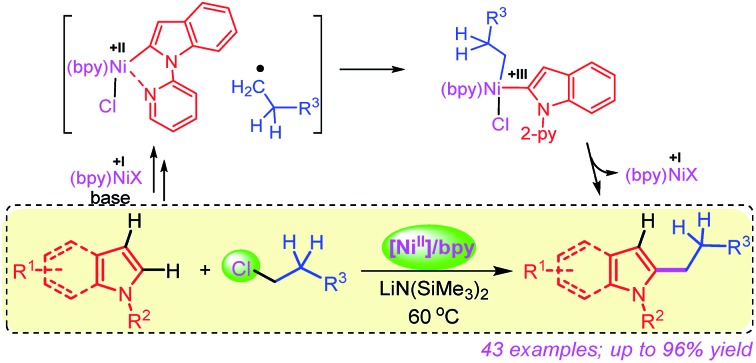
A mild and efficient nickel-catalyzed method for the chemo and regioselective coupling of unactivated alkyl chlorides with the C–H bond of indoles and pyrroles at 60 °C is described.

## Introduction

Functionalized heterocycles are core structures that are found in many natural products, vital drug candidates, and other compounds with significant biological activity.^1^ Therefore, direct and regioselective C–H functionalization of heteroarenes,^2^ including that of privileged indoles,^3^ by transition-metal catalysis has attracted considerable attention. Particularly, the regioselective alkylation of indoles to synthesize alkylated indoles is an important yet challenging reaction.^4^ The biggest hurdles in the development of an alkylation protocol using unactivated alkyl halides, especially those with β-hydrogen atoms, are the reluctance of these electrophiles to undergo oxidative addition, and their tendency to encounter competitive side reactions (β-hydrogen elimination and hydrodehalogenation).

Although the C-3 alkylation of indoles can be achieved by catalytic Friedel–Crafts alkylation, allylic alkylation, and conjugate addition,^5^ the regioselective direct C(2)–H alkylation of indoles with alkyl halides is extremely limited.^6^ For example, Bach has demonstrated the C-2 alkylation of indoles with alkyl bromides *via* a norbornene-mediated Catellani-type reaction,^7^ using a high loading of the precious Pd-catalyst. In contrast, nickel-catalyzed alkylation of indoles using alkyl iodides was demonstrated by us,^8^ wherein a high reaction temperature (150 °C) is essential for successful reaction.

Surprisingly, to date, the selective alkylation of indoles and other related arenes has primarily been achieved by employing reactive alkyl iodides or bromides.^9^ However, the attempt to use high-demand and inexpensive alkyl chlorides has been less successful.^10^ Therefore, a generalized protocol for the direct C-2 alkylation of privileged indoles and related heteroarenes using readily available, inexpensive and challenging alkyl chlorides under mild conditions is highly desirable. Herein, we report the first general method for the coupling of unactivated alkyl chlorides with the C–H bond of indoles and pyrroles using a naturally abundant and inexpensive Ni(ii)-catalyst at 60 °C. Notable aspects of this work include (i) mild and efficient C–H alkylation of indoles and pyrroles, (ii) ample scope with challenging alkyl chlorides with a high degree of tolerance in functionality, (iii) exceptional chemo and regioselectivity for C–halide activation, and (iv) mechanistic insights by detailed experimental study.

## Results and discussion

In general, most of the previous nickel-catalyzed C–H functionalizations employ high reaction temperatures (130–160 °C) to achieve the desired coupling products,^11^ which significantly limit the methodologies. To make the Ni-catalyzed C–H functionalization more general and practical, the major challenge is to perform the reaction under mild conditions employing a wide variety of inexpensive and high-demand unactivated chloro-electrophiles. To achieve this target for alkylation, we screened the coupling of alkyl chlorides with indoles in the presence of lithium bis(trimethylsilyl)amide (LiHMDS) at 60 °C. An optimization study was initiated for the coupling of 1-(pyridin-2-yl)-1*H*-indole (**1a**) with 1-chlorooctane (**2a**) employing a Ni(OAc)_2_/1,10-phenanthroline (phen) catalyst (Tables 1, and S1 in the ESI†). Initial experiments were conducted to screen suitable nickel precursors, such as (CH_3_CN)_2_NiBr_2_, Ni(OTf)_2_, (dme)_2_NiCl_2_ and (thf)_2_NiBr_2_, and it was found that the coupled product 2-octyl-1-(pyridin-2-yl)-1*H*-indole (**3aa**) could be obtained in 86% yield with a (thf)_2_NiBr_2_/phen catalyst system (Table 1, entries 1–5). Among various nitrogen ligands screened (entries 5–8), the 2,2′-bipyridine (bpy) was slightly superior and afforded **3aa** in 88% yield after 5 h (entry 9). The alkylation reaction was less effective when performed at 50 °C (entry 10). The defined complexes (bpy)NiBr_2_ and [(bpy)_3_Ni·NiBr_4_] as catalysts required longer reaction time (16 h) for the alkylation reaction (entries 11–14), which could be due to the slow formation of an active catalyst from (bpy)NiBr_2_ or [(bpy)_3_Ni·NiBr_4_]. The alkylation in the presence of other bases, such as NaO^*t*^Bu and KO^*t*^Bu, gave traces of product, whereas in the presence of mild bases, Na_2_CO_3_, K_2_CO_3_ and Cs_2_CO_3_, coupling was not observed. The nickel catalyst and LiHMDS were essential for the reaction, and without these alkylation did not occur (entries 15 and 16). The possibility of different substituents/directing groups at the *N*-center of indole moiety was explored (Fig. S1 in the ESI†). Notably, 2-pyrazinyl substituted indole afforded 29% of the desired alkylation product, whereas 2-pyrimidinyl indole was ineffective. Free-*N*H indole, *N*-Me indole or indole bearing *N*-acetyl, *N*-Boc, 3-pyridinyl, and 4-pyridinyl groups were unreactive or decomposed under the reaction conditions.

**Table 1:** Optimization of reaction conditions^*a*^

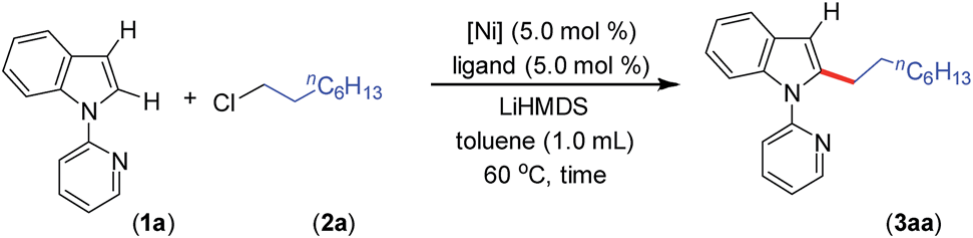				
Entry	[Ni]	Ligand	*t* (h)	**3aa** ^*b*^ (%)
1	Ni(OAc)_2_	phen	24	17
2	(CH_3_CN)_2_NiBr_2_	phen	24	38
3	Ni(OTf)_2_	phen	24	82
4	(dme)NiCl_2_	phen	24	72
5	(thf)_2_NiBr_2_	phen	24	86
6	(thf)_2_NiBr_2_	bpy	24	92
7	(thf)_2_NiBr_2_	d^*t*^Bu-bpy	24	87
8	(thf)_2_NiBr_2_	Neocuprine	24	90
9	(thf)_2_NiBr_2_	bpy	5	92 (88)^*c*^
10^*d*^	(thf)_2_NiBr_2_	bpy	12	57
11	(bpy)NiBr_2_	—	5	15
12	(bpy)NiBr_2_	—	16	86
13	(bpy)_3_Ni·NiBr_4_	—	5	52
14	(bpy)_3_Ni·NiBr_4_	—	16	86
15^*e*^	(thf)_2_NiBr_2_	bpy	5	—
16	—	bpy	5	—
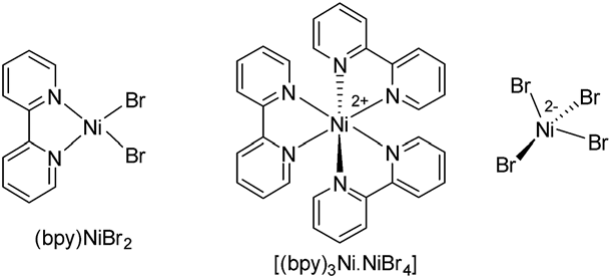				

^*a*^Reaction conditions: **1a** (0.039 g, 0.2 mmol), **2a** (0.059 g, 0.4 mmol), [Ni] cat (0.01 mmol, 5 mol%), ligand (0.01 mmol, 5 mol%), LiHMDS (0.067 g, 0.4 mmol).

^*b*^ ^1^H NMR yield using CH_2_Br_2_ as the internal standard.

^*c*^Isolated yield.

^*d*^Reaction performed at 50 °C.

^*e*^Without LiHMDS. LiHMDS = lithium bis(trimethylsilyl)amide, (LiN(SiMe_3_)_2_); dme = 1,2-dimethoxyethane, phen = 1,10-phenanthroline, and bpy = 2,2′-bipyridine.

**Scheme 1:**
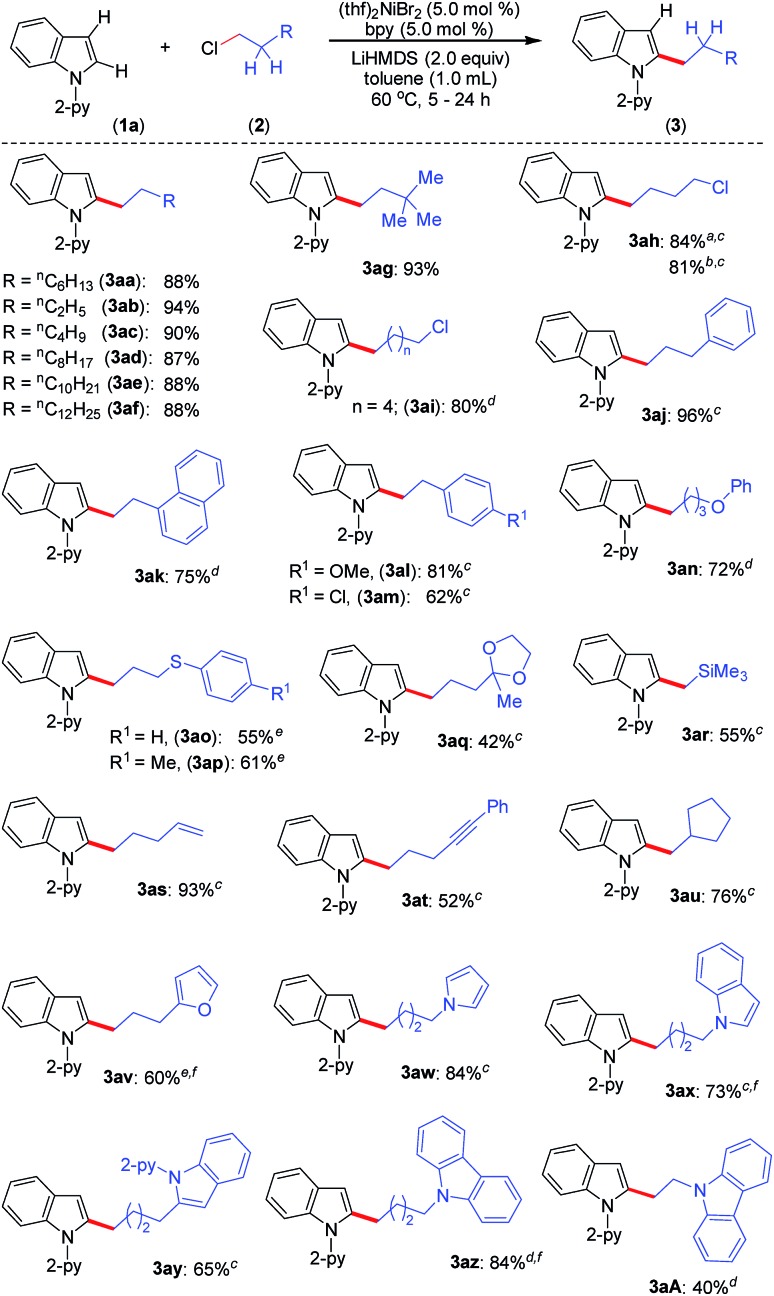
Scope of C-2 alkylation of indoles with primary alkyl chlorides. Reaction conditions: indole **1** (0.20 mmol), alkyl halide **2** (0.40 mmol), LiHMDS (0.067 g, 0.40 mmol), (thf)_2_NiBr_2_ (0.0037 g, 0.01 mmol), bpy (0.0016 g, 0.01 mmol), and toluene (1.0 mL). Yield of isolated compound. For **3aa–3ag**, the reaction time is 5 h. ^*a*^ Using 1-bromo-4-chlorobutane. ^*b*^ Using 1-iodo-4-chlorobutane. ^*c*^ Reaction time 16 h. ^*d*^ Reaction time 24 h. ^*e*^ Reaction performed at 80 °C for 24 h. ^*f* 1^H NMR yield using CH_2_Br_2_ as an internal standard.

Upon achieving the C-2 alkylation of indoles using an inexpensive (thf)_2_NiBr_2_/bpy catalyst under mild conditions, we explored the scope of the reaction with a variety of simple and functionalized alkyl chlorides (Scheme 1). Alkyl chlorides with different chain lengths or branching were efficiently coupled, and the reaction proceeded smoothly at 60 °C affording good to excellent yields of the products **3aa–3ag**. Similar product yields were observed in our previous protocol for the alkylation of indole, albeit using *n*-alkyl bromides at high reaction temperature (150 °C).^8^ 1-Bromo-4-chlorobutane and 1-iodo-4-chlorobutane electrophiles chemoselectively reacted with indole **1a** and produced 2-(4-chlorobutyl)-1-(pyridin-2-yl)-1*H*-indole (**3ah**) in good yield leaving the –Cl functionality untouched. Particularly, a low product yield was reported for the same reaction using an earlier method.^8^ The coupling partner 1,6-dichlorohexane efficiently reacted on one C(sp^3^)–Cl to give the product **3ai** in 80% yield. Notably, in all these cases exclusive mono-indolation products were obtained. The coupling of C(sp^3^)–Br and C(sp^3^)–I sites in the presence of a C(sp^3^)–Cl bond and selective one C(sp^3^)–Cl coupling (in the case of 1,6-dichlorohexane) are highly significant, as the resulting products can be used for further functionalization. Aryl-substituted alkyl chlorides also smoothly coupled with indole to deliver **3aj–3am**. Interestingly, the activation of the C(sp^3^)–Cl bond was highly selective in the presence of a C(sp^2^)–Cl bond (**3am**), and the coupling of C(sp^2^)–Cl with indole C(2)–H was not observed. The reaction delivered an array of 2-alkylated indoles with diverse functional groups, such as phenyl ether (**3an**), thioether (**3ao**, **3ap**), ketal (**3aq**), silyl (**3ar**), alkenyl (**3as**) and alkynyl (**3at**) groups. Tolerability of such functionalities is unprecedented in this type of transformation,^8^ including in previous examples using precious metal catalysts. The reaction was sensitive to ester, –CN, and –NO_2_ functionalities, and the electrophiles containing such groups were decomposed under the reaction conditions (for details see the ESI†). Notably, the use of 6-chlorohex-1-ene as the electrophile produced 2-(cyclopentyl-methyl)-1-(pyridin-2-yl)-1*H*-indole (**3au**) in 76% yield as the major product *via* radical cyclization, and only a trace (9%) of the direct alkylation product, 2-(hex-5-en-1-yl)-1-(pyridin-2-yl)-1*H*-indole, was observed. The alkyl chlorides containing heterocycles, such as furan, pyrrole, indole, and carbazole efficiently reacted with indole and delivered the desired alkylation products (**3av–3aA**) in moderate to good yields. Coupling of similar electrophiles was not demonstrated in our earlier protocol.^8^ The observed low yield during the alkylation of indole with 9-(2-chloroethyl)-9*H*-carbazole (**2A**) arises due to the severe proto-de-alkylation of the coupling partner resulting in the formation of 9*H*-carbazole. Notably, in all cases, regioselective C(2)–H alkylation was realized, and neither C-3 alkylation nor C-2/C-3 double alkylation was observed, which is very crucial as electrophilic activation can further functionalize the C(3)–H bond.

**Scheme 2:**
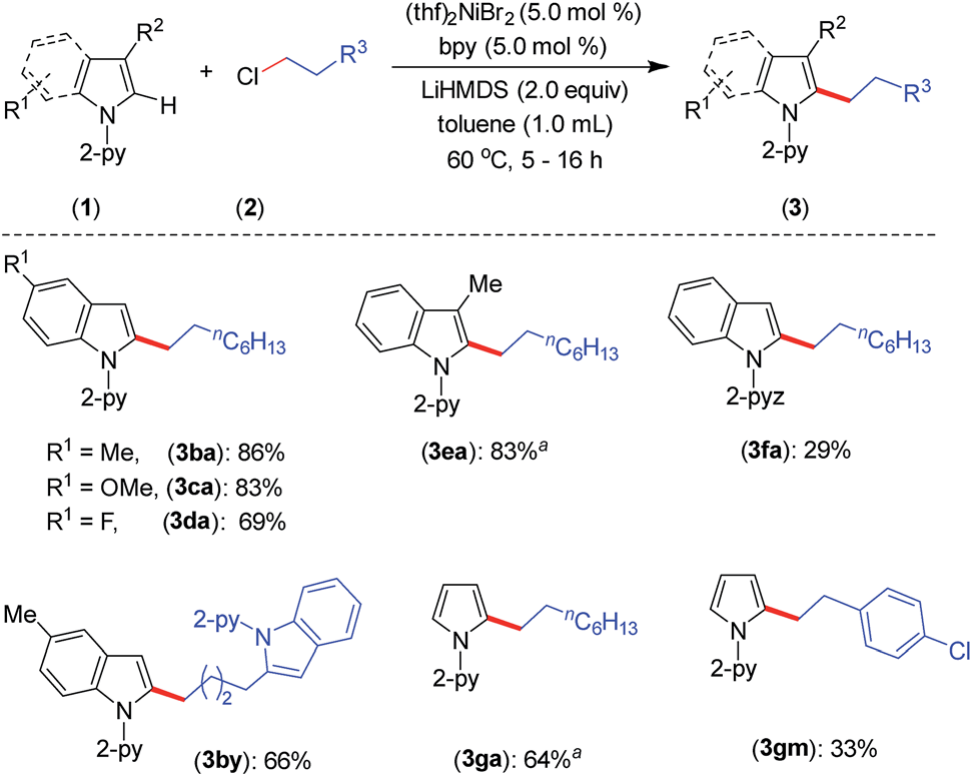
Scope for substituted indoles and pyrroles. Yield of isolated compound. ^*a* 1^H NMR yield using CH_2_Br_2_ as an internal standard. For **3ba–3fa**, the reaction time is 5 h. For **3da**, **3by**, **3ga** and **3gm**, the reaction time is 16 h.

Furthermore, the indoles with electron-donating and electron-withdrawing substituents at the C-5 position smoothly participated in the alkylation to deliver the products **3ba–3da** and **3by** in good to excellent yields (Scheme 2). The sterically hindered C-3 substituted indole (**1e**) could be alkylated with good activity, though the same substrate gave a very poor yield with an earlier protocol.^8^ In addition to indoles, the pyrrole moiety was alkylated at the C-2 position to deliver selective mono-alkylation products **3ga** and **3gm**. Notably, the reaction of compound **3ga** with 1-chlorooctane to achieve 2,5-dialkylation was unsatisfactory and resulted in 13% conversion. Other 2-pyridinyl-substituted heteroarenes, such as 1-(pyridin-2-yl)-7-azaindole, 2-(furan-2-yl)pyridine, 2-(thiophen-2-yl)pyridine and 2-(thiophen-3-yl)pyridine, were not competent under the catalytic conditions.

**Scheme 3:**
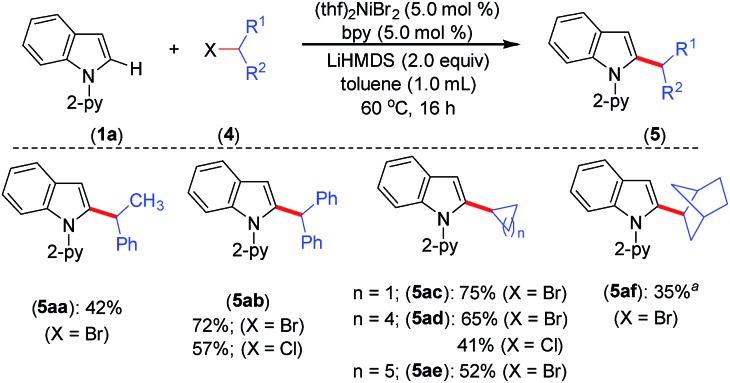
Scope for secondary alkyl halides. Yield of isolated compound. ^*a* 1^H NMR yield using CH_2_Br_2_ as an internal standard.

The optimized reaction conditions were further explored to the coupling of secondary alkyl chlorides and bromides with indole **1a** (Scheme 3). Thus, the acyclic phenyl-substituted secondary alkyl chlorides and bromides were coupled at the C-2 position of indole to afford products **5aa** and **5ab** in moderate to good yields. Similarly, cyclic secondary alkyl halides (**4c–e**) with different ring sizes were reacted to produce moderate to good yields. The electrophile 2-bromobicyclo[2.2.1]heptane (**4f**) gave the alkylated product **5af** in 35% yield. Notably, the coupling of acyclic secondary chlorides, such as 2-chlorobutane and 2-chloropentane, was inefficient and afforded only trace amounts of the product (<5%). Furthermore, coupling was not observed with tertiary alkyl chlorides, such as *tert*-butyl chloride and 1-chloroadamantane.

**Scheme 4:**
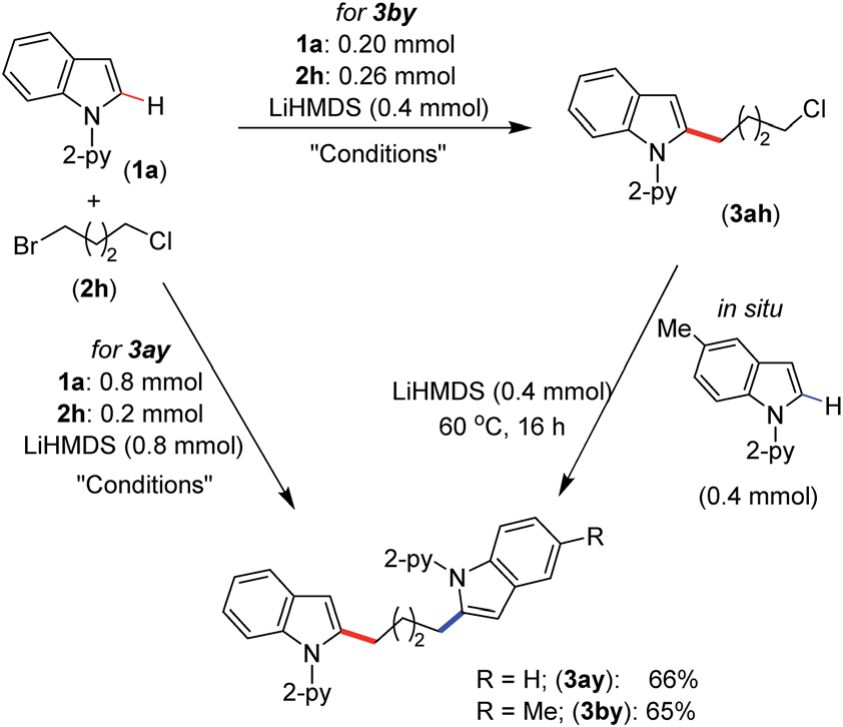
*In situ* synthesis of bis(indolyl)butane. Conditions: (thf)_2_NiBr_2_ (0.02 mmol), bpy (0.02 mmol), toluene (2.0 mL), 60 °C, 16 h.

We have demonstrated the synthesis of symmetrical and unsymmetrical bis(indolyl)butane derivatives in a single-pot using 1-bromo-4-chlorobutane (**2h**) (Scheme 4). Thus, the treatment of 4.0 equiv. of indole **1a** with 1-bromo-4-chlorobutane (**2h**, 1.0 equiv.) under standard alkylation conditions afforded symmetrical bis(indolyl)butane **3ay** in 66% yield. However, unsymmetrical bis(indolyl)butane **3by** was isolated in 65% yield by the sequential reaction of 1-bromo-4-chlorobutane (**2h**) with indoles **1a** and **1b** in one pot. The demonstrated protocol provided a unique approach for the synthesis of bis(indolyl)alkyl derivatives, which can be extended to the development of many other symmetrical and unsymmetrical scaffolds.

We have performed a gram-scale reaction to demonstrate the practical utility of the mild nickel catalyzed alkylation protocol. Thus, the reaction of 1.0 g of 1-pyridin-2-yl-1*H*-indole (**1a**, 5.15 mmol) with 1-chlorooctane (**2a**, 1.53 g, 10.30 mmol), employing (thf)_2_NiBr_2_/bpy (5 mol%) in the presence of LiHMDS (1.72 g, 10.30 mmol), afforded 1.24 g of the product **3aa**, which amounts to 79% yield.

**Scheme 5:**
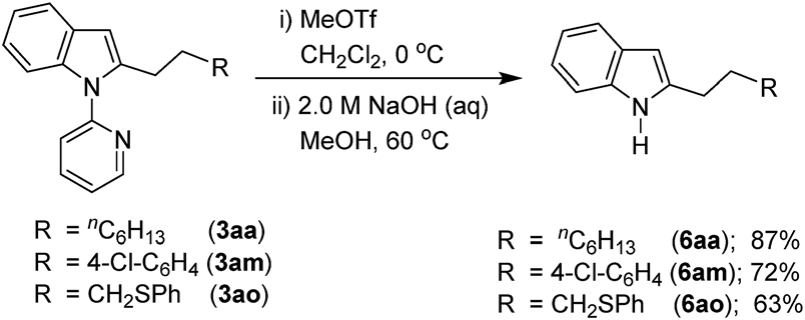
Removal of the 2-pyridinyl directing group.

Considering the importance of functionalized free *N*–H indoles, the removal of a 2-pyridinyl group was demonstrated. Thus, the C-2 alkylated indoles **3aa**, **3am**, and **3ao** were treated with MeOTf followed by reaction in NaOH (2.0 M) leading to the formation of C-2 alkylated free *N*–H indoles **6aa**, **6am**, and **6ao**, respectively (Scheme 5).

## Mechanistic investigations

A series of experiments have been conducted to probe the reaction mechanism. ^1^H NMR analysis of a mixture of (thf)_2_NiBr_2_, bpy and LiHMDS showed a single broad peak at 10.69 ppm for the (bpy)Ni protons, which accounts for >98% w.r.t. the internal standard (see, Fig. S4(B) in the ESI†). The broad single peak indicates that the generated nickel species could be a paramagnetic Ni(i) and/or tetrahedral Ni(ii) species^12^ (conclusive identification was demonstrated by EPR and XPS; discussed *vide infra*). Upon the addition of substrates **1a** and **2a** to the reaction mixture, the peak corresponding to (bpy)Ni completely disappeared. Notably, the reaction mixture was highly homogeneous, and the free ligand was not detected. We assume that the resulting nickel species in this reaction could be paramagnetic in nature and might be involved during the catalysis.

**Scheme 6:**
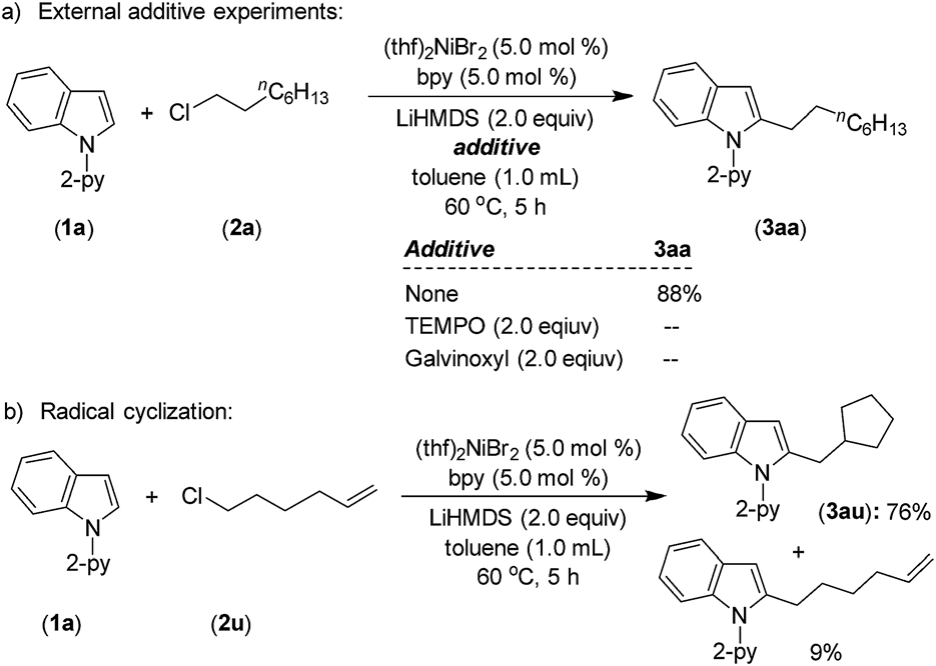
External additive and radical clock experiments.

A standard alkylation reaction was attempted in the presence of radical scavengers, TEMPO (2.0 equiv.) and galvinoxyl (2.0 equiv.), wherein the reaction was completely inhibited (Scheme 6a). Notably, the reaction of indole **1a** with 6-chlorohex-1-ene afforded 76% of 2-(cyclopentylmethyl)-1-(pyridin-2-yl)-1*H*-indole (**3au**) as a major product *via* radical cyclization (Scheme 6b), suggesting the involvement of an alkyl radical intermediate during the reaction.

**Fig. 1:**
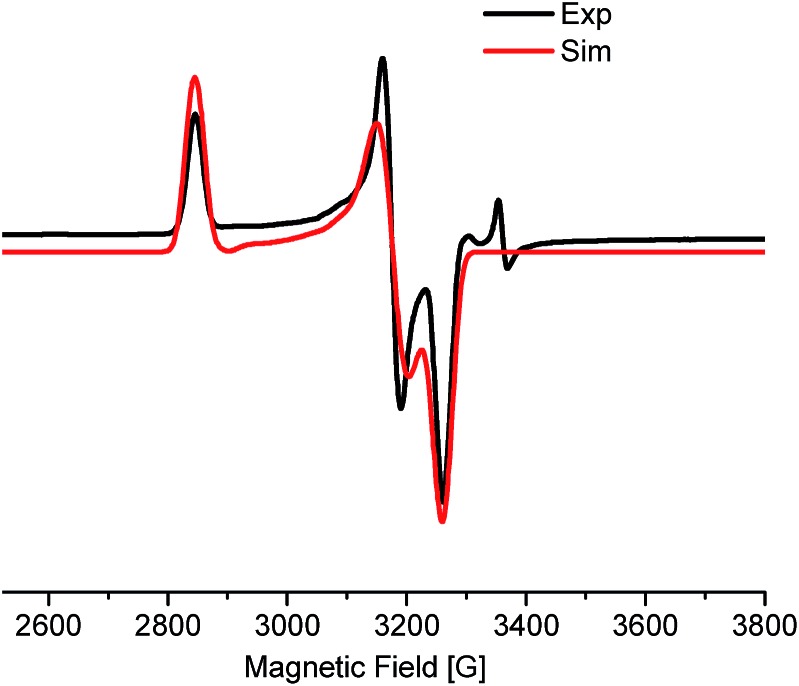
EPR spectrum of the incomplete alkylation reaction. Rhombic symmetry with *g* anisotropy values, *g*_*xx*_ (2.385), *g*_*yy*_ (2.137), and *g*_*zz*_ (2.082). The *g*-values are derived from simulation of the EPR spectrum.

The EPR measurement of the reaction mixture, (thf)_2_NiBr_2_, bpy and LiHMDS (60 °C, 30 min), exhibits a rhombic spectrum (*g*_1_ > *g*_2_ > *g*_3_) (Fig. 1; approximately 18% w.r.t. the external standard). The *g*-factor (*g*_av_ = 2.201) is suggestive of the unpaired spin residing in an orbital with significant metal characteristics (most likely Ni^I^).^13^ This observation highlights the feasibility of a one-electron reduction of Ni(ii) to Ni(i) in the presence of LiHMDS. Notably, EPR measurements of other control experiments, including that of (thf)_2_NiBr_2_/bpy, resulted in spectra that were too complicated to make any concrete judgments on the nature of the radical species.

**Scheme 7:**
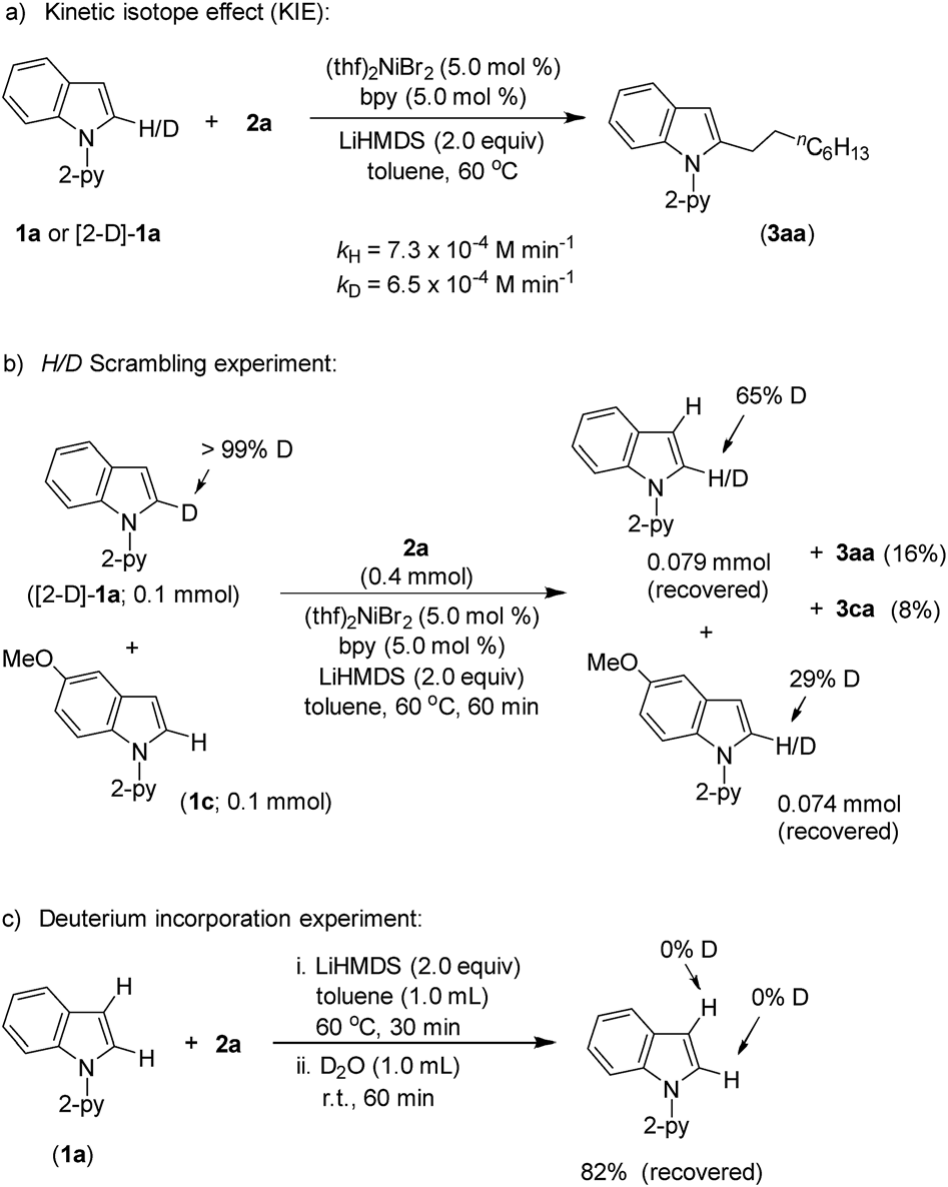
Deuterium labeling experiments.

**Scheme 8:**
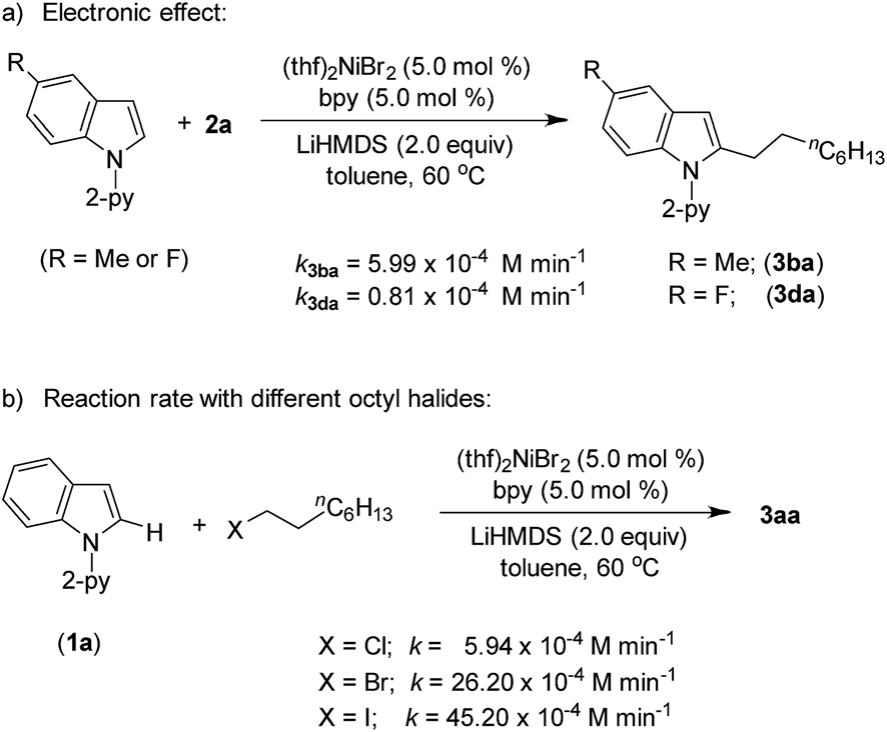
Reaction rates with different substrates.

Product (**3aa**) formation during the alkylation was consistent, and the initial rate of the reaction was 7.3 × 10^–4^ M min^–1^ (Fig. S5†). The kinetic isotope effect (KIE) value of 1.12 (from independent reaction rates of **1a** and [2-D]-**1a**) ruled out the involvement of C–H activation in the rate-limiting step (Scheme 7a).^14^ Furthermore, a substantial H/D exchange between indole [2-D]-**1a** and 5-methoxy-1-(pyridine-2-yl)-1*H*-indole (**1c**) at the C(2)–H position is indicative of reversible C–H nickelation (Scheme 7b). The attempted alkylation reaction (both in the presence and absence of (thf)_2_NiBr_2_/bpy) followed by quenching with D_2_O (Scheme 7c) shows no deuterium incorporation at the C(2)–H or C(3)–H of indole **1a**, which ruled out a simple base-mediated deprotonation pathway. Independent rate determination highlights that the electron-rich indole promotes alkylation, thus suggesting probable rate-influencing oxidative addition (Scheme 8a).^15^ This finding also suggests the improbability of reductive elimination as a rate-influencing step, because reductive elimination would preferably be assisted by an electron-deficient substituent on the substrate. Notably, 1-iodooctane and 1-bromooctane reacted 7.6 and 4.4 times faster, respectively, than 1-chlorooctane during the alkylation; hence, C–halide bond activation is assumed to be very crucial (Scheme 8b). Considering the unlikeliness of C–H nickelation or reductive elimination as the rate influencing step,^14^ we believe that C(alkyl)–Cl bond activation could be a probable rate-limiting step.

**Scheme 9:**
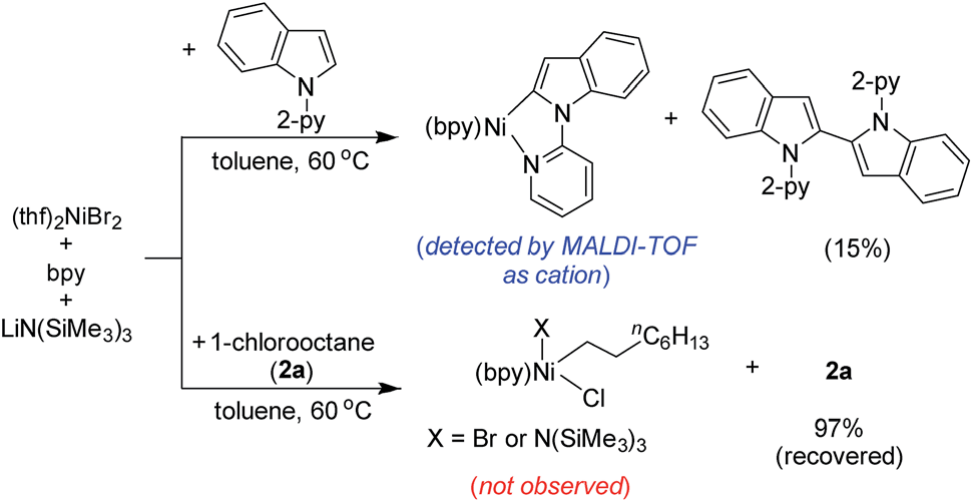
Controlled stoichiometric reactions.

The stoichiometric reaction of (thf)_2_NiBr_2_/bpy with indole **1a** in the presence of LiHMDS at 60 °C resulted in the formation of 1,1′-di(pyridin-2-yl)-1*H*,1′*H*-2,2′-biindole (15%; self-coupling of **1a**) (Scheme 9). We were unable to isolate the presumed intermediate (bpy)Ni(2-indolyl-2-pyridine); however, the same species was detected by MALDI-TOF analysis.^16^ Notably, the oxidative coupling of indole increases upon increasing the nickel loading, which led to the reduction in the yield of alkylation. This finding strongly suggests that a Ni(ii)-species is unlikely the active catalyst. Considering the detection of the intermediate (bpy)Ni(2-indolyl-2-pyridine) and EPR findings, we tentatively assumed a Ni(i)-species as the active catalyst. Notably, the treatment of (thf)_2_NiBr_2_/bpy with 1-chlorooctane in the presence of LiHMDS at 60 °C did not lead to any product.

**Fig. 2:**
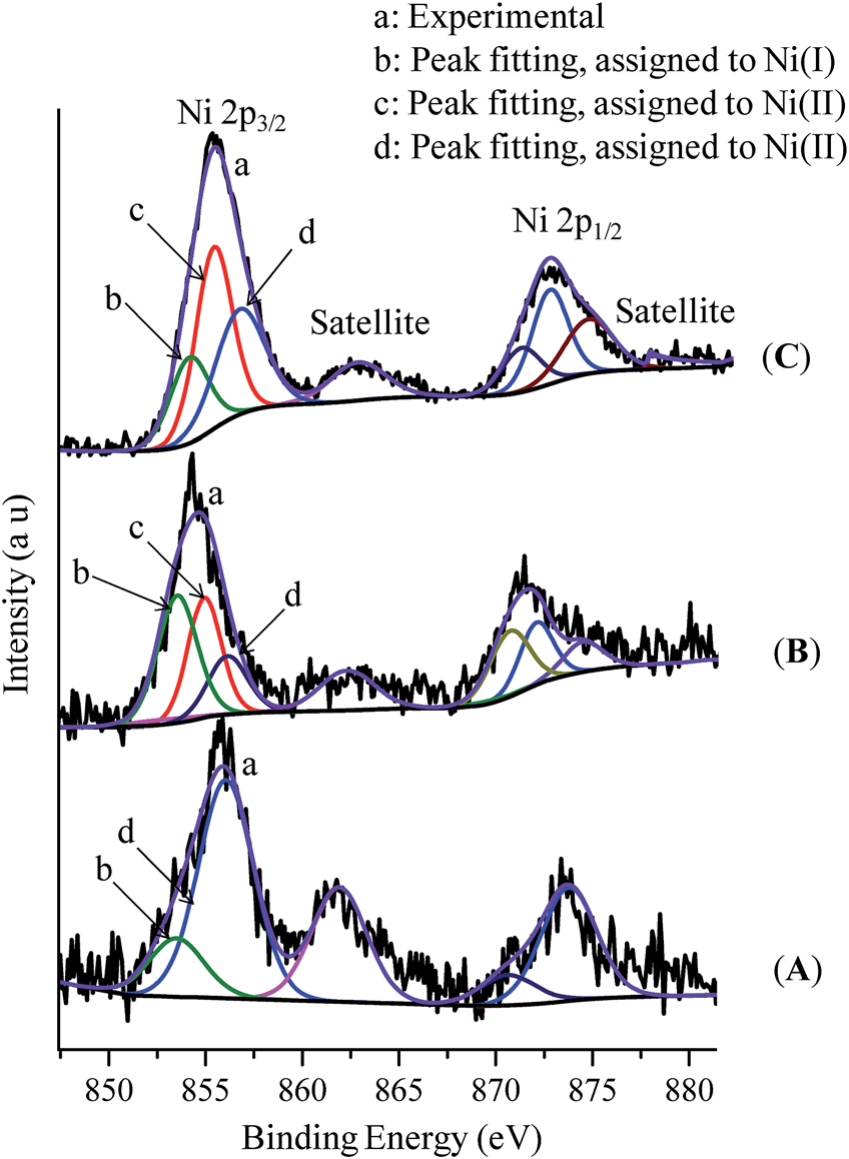
X-ray photoelectron spectra: (A) (thf)NiBr_2_ + bpy + LiHMDS, (B) (thf)NiBr_2_ + bpy + LiHMDS + **1a**, and (C) (thf)NiBr_2_ + bpy + LiHMDS + **1a** + **2a** (standard reaction).

We have extended our study to the XPS analysis of the reaction to establish the oxidation state of the involved Ni-species. The Ni 2p_3/2_ XPS spectrum of the complex (thf)_2_NiBr_2_ displays a sharp peak centered at around 856.0 eV for the Ni(ii)-species (Fig. S14(A) in the ESI†). 2p_1/2_ is also indexed at 873.2 eV (matching with a 17 eV separation between spin–orbit split) along with a satellite feature at 861 eV. Similarly, the Ni 2p_3/2_ XPS spectra of complexes (Ph_3_P)_3_NiCl and (cod)_2_Ni show peaks at 853.4 eV and 852.6 eV for Ni(i) and Ni(0), respectively (Fig. S13†).^17^ The XPS spectrum of the reaction mixture of (thf)_2_NiBr_2_, bpy and LiHMDS (10 equiv.) is much broader with a larger FWHM indicating the multiple oxidation states (Fig. 2A). We could fit two peaks in the main 2p_3/2_ photoelectron peak at 856.0 eV and 853.4 eV, which are assigned to (bpy)Ni(ii) and a Ni(i) (approx. 37%) intermediate, respectively. This observation is consistent with the EPR finding that an odd-electron nickel species, *i.e.* Ni(i), is generated from the Ni(ii) complex in the presence of LiHMDS. XPS analysis of the control reaction, (thf)_2_NiBr_2_ + bpy + LiHMDS + indole **1a**, shows three peaks at 853.4 eV, 854.8 eV and 856.0 eV (Fig. 2B). The peaks at 853.4 eV and 856.0 eV are for Ni(i) and Ni(ii), respectively. However, the peak at 854.8 eV has a slightly lower binding energy than the peaks observed for (bpy)Ni(ii) or (thf)_2_Ni(ii), and thus could be due to an electron-rich Ni(ii)-species, (bpy)Ni(ii)X(2-indolyl-2-pyridine).^17^ XPS analysis of the standard alkylation reaction, *i.e.* (thf)_2_NiBr_2_ + bpy + LiHMDS + **1a** + **2a**, also exhibited three peaks at 853.4 eV [Ni(i)], 854.8 eV [Ni(ii)] and 856.0 eV [Ni(ii)] (Fig. 2C). Notably, XPS analysis of the reaction without indole **1a**, but in the presence of **2a** [*i.e.* (thf)_2_NiBr_2_ + bpy + LiHMDS + **2a**], showed the presence of only Ni(ii) species (856.0 eV). Both the XPS and EPR findings strongly supported the involvement of a Ni(i) species that could be the probable active catalyst.

### Computational study

The intermediate structure **A** (X = Br; discussed in Catalytic cycle) was validated by density functional theory (DFT) based calculations. The study demonstrates that the charge on the Ni atom is 0.8*e* (accounting to a charge of nearly one electron). Frequency analysis showed all positive frequencies. Frontier orbital analysis (see Fig. S16 in the ESI†) of a singly occupied molecular orbital (SOMO) with one electron reveals contributions from Ni-d_*xy*_ and N-p_*y*_ orbitals. The lowest unoccupied molecular orbital (LUMO) is contributed by overlapping of Ni-d_*yz*_ and N-p_*z*_ orbitals. Finally, the paramagnetic nature of this species is corroborated from the EPR calculations, which gave a *g* value of 2.2. Thus, the analysis from DFT studies corroborates our experimental findings on the paramagnetic intermediate **A**.

### Catalytic cycle

**Fig. 3:**
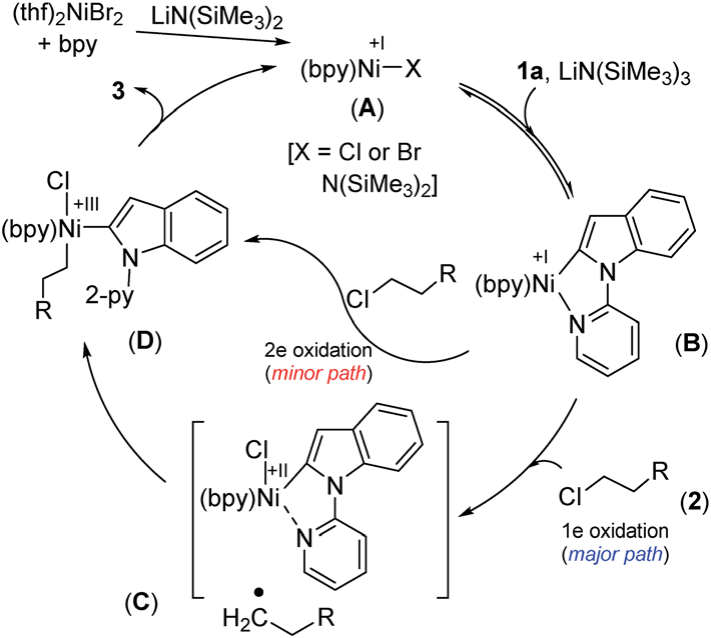
Plausible mechanistic pathways.

We have drawn tentative catalytic cycles based on our mechanistic study and literature precedents (Fig. 3).^18^ The Ni(ii) complex is first reduced to an active Ni(i)-species in the presence of LiN(SiMe_3_)_2_ (supported by both EPR and XPS analyses). One-electron reduction of Ni(ii) by LiN(SiMe_3_)_2_ could occur *via* the displacement of a ˙N(SiMe_3_)_3_ radical.13a,^19^ Indole **1a** then reacts reversibly with low-valent Ni(i) (**A**) to deliver the intermediate species **B**. The nickel complex **B** would prompt the radical generation from the alkyl chloride in the rate-limiting step, and undergo one-electron oxidation to produce the intermediate **C**. A radical clock experiment strongly supports the single-electron transfer pathway and the intermediacy of an alkyl radical. Nitrogen (N_2-py_)-decoordination and subsequent recombination of the alkyl radical would deliver the complex **D**. As a minor path, alkyl chloride **2** can undergo 2e oxidative addition to produce **D**. Upon reductive elimination of product **3** from **D**, the active catalyst **A** will be regenerated. Although our studies support a mono-nuclear SET pathway for the reaction, the possibility of a bimetallic pathway for the activation of alkyl chlorides cannot be ruled out.^20^

## Conclusions

In summary, we have developed a mild and efficient nickel-catalyzed protocol for the chemo- and regioselective coupling of unactivated alkyl chlorides with indoles and pyrroles. The reaction is compatible with a wide range of simple and functionalized primary and secondary alkyl chlorides as well as with electronically distinct indoles. The chemoselectivity with regard to alkyl halides is especially excellent with the selective C(sp^3^)–I, C(sp^3^)–Br or C(sp^3^)–Cl bond activation. Furthermore, a C(sp^3^)–Cl bond preferentially activated and coupled to the C(sp^2^)–Cl bond. The utility of this nickel-catalyzed protocol is demonstrated by the removal of the directing group. Mechanistic investigation of the alkylation reaction allowed us to propose a tentative catalytic cycle that follows a SET process involving the rate influencing alkyl–Cl bond activation. EPR and XPS analyses indicated the involvement of a Ni(i) active species, thus supporting a Ni(i)/Ni(iii) pathway. We firmly believe that the uniqueness of the demonstrated Ni-catalyzed protocol would provide an enormous boost for the development and exploration of many other catalysts.

## Conflicts of interest

There are no conflicts to declare.

## Supplementary Material

Supplementary information
