# Supplementary material for: Nickel-catalyzed C–H alkylation of indoles with unactivated alkyl chlorides: evidence of a Ni(i)/Ni(iii) pathway
Source: Chem Sci. 2019 Aug 19;10(41):9493–500. doi: 10.1039/c9sc01446b (PMC7017866; doi:10.1039/c9sc01446b)
Supplement: Supplementary file 1 [file SC-010-C9SC01446B-s001.pdf]

Electronic Supplementary Material (ESI) for Chemical Science.
This journal is © The Royal Society of Chemistry 2019

Supporting Information

Nickel-catalyzed C–H alkylation of indoles with unactivated alkyl chlorides: Evidence of Ni(I)/Ni(III) pathway

Dilip K. Pandey,[a,b] Shidheshwar B. Ankade,[a,b] Abad Ali,[a] C. P. Vinod,[c] and Benudhar Punji*,[a,b]

[a]Organometallic Synthesis and Catalysis Group, Chemical Engineering Division, [b]Academy of Scientific and Innovative Research (AcSIR), and [c]Catalysis Division, CSIR–National Chemical Laboratory (CSIR–NCL), Dr. Homi Bhabha Road, Pune 411 008, India

Phone: + 91-20-2590 2733, Fax: + 91-20-2590 2621.

E-mail: b.punji@ncl.res.in

Contents

		Page no
1.	General experimental	S3
2.	Reaction optimization	S5
3.	Representative procedure for alkylation	S7
4.	Characterization data of alkylated compounds	S8
5.	Other indoles and alkyl chlorides employed	S39
6.	Procedure for one pot double alkylation	S40
7.	Procedure for removal of directing group	S41
8.	Procedure for NMR tube experiment	S43
9.	Procedure for stoichiometric reaction	S45
10.	External additive experiments	S46
11	Radical clock experiment	S46
12.	Kinetics experiments	S47
13.	Deuterium labeling experiments	S52
14.	Procedure for EPR study	S56
15.	Procedure for XPS analysis	S57
16.	Computational method	S60
17.	Procedure for MALDI-TOF analysis	S61
18.	References	S61
19.	MALDI-TOF spectra of Ni-intermediates	S63
20.	^{1}H and ^{13}C NMR spectra	S66

1. General experimental

All manipulations were conducted under an argon atmosphere either in a glove box or using standard Schlenk techniques in pre-dried glass wares. The catalytic reactions were performed in flame-dried reaction vessels with Teflon screw cap. Solvents were dried over Na/benzophenone and distilled prier to use. Liquid reagents were flushed with argon prior to use. Base LiHMDS was sublimed and used fresh. All other chemicals were obtained from commercial sources and were used without further purification. The alkyl chloride electrophiles **2k**,[S1] **2l**,[S2] **2m**,[S3] **2n**,[S4] **2o-p**,[S5] **2s**,[S6] **2t**,[S7] **2v**,[S8] **2w**,[S9] **2x**,[S9] **2z**,[S9] **2a'**[S10] indole derivatives[S11] and nickel complexes, (bpy)$NiBr_2$,[S12] $(bpy)_{1.5}NiBr_2$,[S13] ($^{Q}NNN^{Et2}$)NiCl[S14] and $(Ph_3P)_3NiCl$[S15] were prepared following the literature procedure. High resolution mass spectrometry (HRMS) mass spectra were recorded on a Thermo Scientific Q-Exactive, Accela 1250 pump. NMR: (^{1}H and ^{13}C) spectra were recorded at 400 or 500 MHz (^{1}H), 100 or 125 MHz {^{13}C, DEPT (distortionless enhancement by polarization transfer)}, 377 MHz (^{19}F), respectively in $CDCl_3$ solutions, if not otherwise specified; chemical shifts (δ) are given in ppm. The ^{1}H and ^{13}C NMR spectra are referenced to residual solvent signals ($CDCl_3$: δ H = 7.26 ppm, δ C = 77.2 ppm). EPR spectra were recorded on JES - FA200 ESR Spectrometer with X and Q band (Standard Frequency (X band) - 8.75-9.65 GHz) at 100 K. MALDI-TOF-MS was performed on ABSCIEX TOF/TOF TM5800 and 2,5-dihyroxybenzoic acid used as a matrix.

XPS analysis. All the spectra were collected using Thermo Kalpha$^+$ spectrometer with a mono chromated Al Kα X-ray source with energy 1486.6 eV. The pass energy for the acquisition was 50 eV for the individual core-level. All the spectral acquisition was done in the presence of

ultra-low energy co-axial electron gun for charge compensation. The peak fitting of the individual core-levels were done using Avantage software with a Shirley type background.

GC method. Gas Chromatography analyses were performed using a Shimadzu GC-2010 gas chromatograph equipped with a Shimadzu AOC-20s auto sampler and a Restek RTX-5 capillary column (30 m x 250 μm). The instrument was set to an injection volume of 1 μL, an inlet split ratio of 10:1, and inlet and detector temperatures of 250 and 320 °C, respectively. UHP-grade argon was used as carrier gas with a flow rate of 30 mL/min. The temperature program used for all the analyses is as follows: 80 °C, 1 min; 30 °C/min to 200 °C, 2 min; 30 °C/min to 260 °C, 3 min; 30 °C/min to 300 °C, 3 min. Response factors for all the necessary compounds with respect to standard *n*-hexadecane were calculated from the average of three independent GC runs.

2. Reaction optimization

Table S1 Optimization of reaction parameters[a]

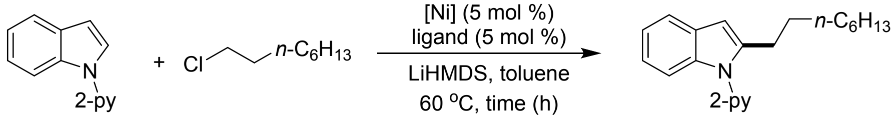

1a **2a** **3aa**

entry	[Ni]	ligand	time (h)	**3aa** (%)[b]
1	$Ni(OAc)_2$	phen	24	17
2	$(CH_3CN)_2NiBr_2$	phen	24	38
3	$Ni(OTf)_2$	phen	24	82
4	$(dme)NiCl_2$	phen	24	72
5	$(thf)_2NiBr_2$	phen	24	86
6	$(thf)_2NiBr_2$	bpy	24	92
7	$(thf)_2NiBr_2$	d^tBu-bpy	24	87
8	$(thf)_2NiBr_2$	neocuprine	24	90
9	$(thf)_2NiBr_2$	bpy	16	90
10	$(thf)_2NiBr_2$	bpy	12	89
11	$(thf)_2NiBr_2$	bpy	8	89
**12**	**$(thf)_2NiBr_2$**	**bpy**	**5**	**88**[c]
13	$(thf)_2NiBr_2$	bpy	4	84
14[d]	$(thf)_2NiBr_2$	bpy	12	57
15[e]	$(thf)_2NiBr_2$	bpy	12	--
16[f]	$(thf)_2NiBr_2$	bpy	5	65
17[g]	$(thf)_2NiBr_2$	bpy	5	36
18[h]	$(thf)_2NiBr_2$	bpy	5	55

19	(bpy)$NiBr_2$	--	5	15
20	(bpy)$NiBr_2$	--	16	86
21	$(bpy)_{1.5}NiBr_2$	--	5	52
22	$(bpy)_{1.5}NiBr_2$	--	16	86
23	(^{Q}NNN^{Et2})NiCl	--	24	50
24[i]	$(thf)_2NiBr_2$	bpy	5	< 5
25[j]	$(thf)_2NiBr_2$	bpy	5	--
26[k]	$(thf)_2NiBr_2$	bpy	5	--
27	--	bpy	5	--

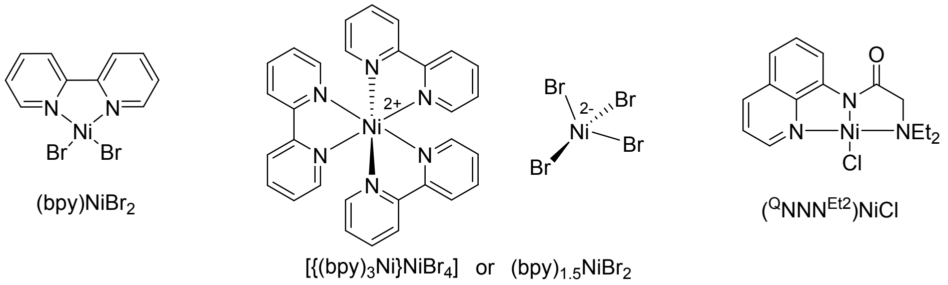

[a]Reaction conditions: Indole **1a** (0.039 g, 0.20 mmol), **2a** (0.059 g, 0.40 mmol), [Ni] cat (0.01 mmol, 5 mol %), ligand (0.01 mmol, 5 mol %), LiHMDS (0.067 g, 0.40 mmol), solvent (1.0 mL). [b]NMR yield using CH_2Br_2 as internal standard. [c]Isolated yield. [d]Reaction performed at 50 ^{o}C. [e]Reaction performed at 40 ^{o}C. [f]*p*-Xylene as solvent. [g]Mesitylene as solvent. [h]*o*-Xylene as solvent. [i]Using LiOtBu or NaOtBu as base. [j]Using Na_2CO_3, NaOAc, K_2CO_3 or Cs_2CO_3 as base. [k]Without base. LiHMDS = Lithium bis(trimethylsilyl)amide {$LiN(SiMe_3)_2$}, dme = 1,2-dimethoxyethane, phen = 1,10-phenanthroline, bpy = 2,2-bipyridine.

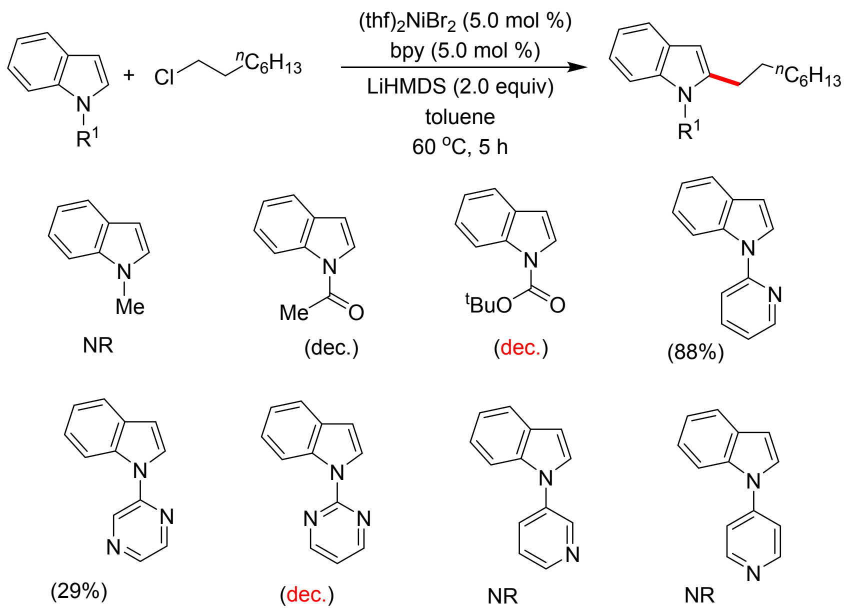

Figure S1. Reactivity of *N*-substituted indoles.

3. Representative procedure for alkylation

Synthesis of 2-octyl-1-(pyridin-2-yl)-1*H*-indole (3aa): To a flame-dried screw-cap tube (5 mL) equipped with a magnetic stir bar were introduced 1-pyridin-2-yl-1*H*-indole (**1a**, 0.039 g, 0.20 mmol), 1-chlorooctane (**2a**, 0.059 g, 0.40 mmol), $(thf)_2NiBr_2$ (0.0037 g, 0.01 mmol, 5.0 mol %), bpy (0.0016 g, 0.01 mmol) and LiHMDS (0.067 g, 0.40 mmol) inside the glove box. To the above mixture in the tube was added toluene (1.0 mL), and the resultant reaction mixture was stirred at 60 °C in a preheated oil bath for 5 h. At ambient temperature, the reaction mixture was quenched with distilled H_2O (10 mL) and neutralized with 2N HCl (0.5 mL). The crude product was then extracted with EtOAc (20 mL x 3). The combined organic extract was dried over Na_2SO_4, and the volatiles were evaporated *in vacuo*. The remaining residue was purified by column chromatography on neutral alumina (petroleum ether/EtOAc: 50/1) to yield **3aa** (0.054 g, 88%) as a light yellow liquid. ^{1}H-NMR (500 MHz, $CDCl_3$): δ = 8.69 (d, J = 3.4 Hz, 1H, Ar–H), 7.94-7.83 (td, J = 7.8, 1.6 Hz, 1H, Ar–H), 7.65-7.56 (m, 1H, Ar–H), 7.44 (d, J = 8.0 Hz, 1H,

Ar–H), 7.39-7.29 (m, 2H, Ar–H), 7.20-7.08 (m, 2H, Ar–H), 6.48 (s, 1H, Ar–H), 2.87 (t, J = 7.6 Hz, 2H, CH_2), 1.59 (quin, J = 7.4 Hz, 2H, CH_2), 1.40-1.28 (m, 4H, CH_2), 1.27-1.23 (m, 6H, CH_2), 0.90 (t, J = 7.1 Hz, 3H, CH_3). $^{13}C\{^1H\}$-NMR (125 MHz, $CDCl_3$): δ = 151.8 (C_q), 149.8 (CH), 141.9 (C_q), 138.4 (CH), 137.4 (C_q), 128.8 (C_q), 122.1 (CH), 121.6 (CH), 121.3 (CH), 120.7 (CH), 120.0 (CH), 110.2 (CH), 102.2 (CH), 32.0 (CH_2), 29.5 (2C, CH_2), 29.3 (CH_2), 28.7 (CH_2), 27.6 (CH_2), 22.8 (CH_2), 14.3 (CH_3). HRMS (ESI): m/z Calcd for $C_{21}H_{26}N_2+H^+$ $[M+H]^+$ 307.2169; Found 307.2160. The ^1H and ^{13}C spectra are consistent with those reported in the literature.[S14]

4. Characterization data of alkylated compounds

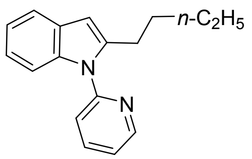

2-Butyl-1-(pyridin-2-yl)-1*H*-indole (3ab): The representative procedure was followed, using substrate **1a** (0.039 g, 0.20 mmol) and 1-chlorobutane (**2b**; 0.037 g, 0.40 mmol), and the reaction mixture was stirred for 5 h. Purification by column chromatography on neutral alumina (petroleum ether/EtOAc: 50/1) yielded **3ab** (0.047 g, 94%) as a light yellow liquid. ^1H-NMR (500 MHz, $CDCl_3$): δ = 8.68 (dd, J = 4.6, 1.1 Hz, 1H, Ar–H), 7.89 (td, J = 7.7, 2.1 Hz, 1H, Ar–H), 7.66-7.56 (m, 1H, Ar–H), 7.44 (d, J = 8.0 Hz, 1H, Ar–H), 7.38-7.28 (m, 2H, Ar–H), 7.21-7.07 (m, 2H, Ar–H), 6.47 (s, 1H, Ar–H), 2.86 (t, J = 7.6 Hz, 2H, CH_2), 1.57 (quin, J = 7.6 Hz, 2H, CH_2), 1.41-1.32 (m, 2H, CH_2), 0.88 (t, J = 7.2 Hz, 3H, CH_3). $^{13}C\{^1H\}$-NMR (125 MHz, $CDCl_3$): δ = 151.8 (C_q), 149.8 (CH), 141.9 (C_q), 138.4 (CH), 137.4 (C_q), 128.8 (C_q), 122.2 (CH), 121.7 (CH), 121.3 (CH), 120.7 (CH), 120.0 (CH), 110.2 (CH), 102.2 (CH), 30.9 (CH_2), 27.3

(CH_2), 22.5 (CH_2), 14.0 (CH_3). HRMS (ESI): *m/z* Calcd for $C_{17}H_{18}N_2$+H^+ $[M+H]^+$ 251.1543; Found 251.1543. The ^{1}H and ^{13}C spectra are consistent with those reported in the literature.[S14]

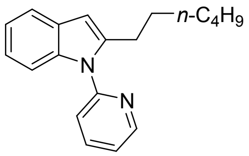

2-Hexyl-1-(pyridin-2-yl)-1*H*-indole (3ac): The representative procedure was followed, using substrate **1a** (0.039 g, 0.20 mmol) and 1-chlorohexane (**2c**; 0.048 g, 0.40 mmol), and the reaction mixture was stirred for 5 h. Purification by column chromatography on neutral alumina (petroleum ether/EtOAc: 50/1) yielded **3ac** (0.050 g, 90%) as a light yellow liquid. ^{1}H-NMR (500 MHz, $CDCl_3$): δ = 8.68 (dd, J = 4.8, 1.0 Hz, 1H, Ar–H), 7.88 (td, J = 7.7, 1.7 Hz, 1H, Ar–H), 7.65-7.56 (m, 1H, Ar–H), 7.44 (d, J = 8.0 Hz, 1H, Ar–H), 7.39-7.28 (m, 2H, Ar–H), 7.21-7.09 (m, 2H, Ar–H), 6.48 (s, 1H, Ar–H), 2.86 (t, J = 7.6 Hz, 2H, CH_2), 1.59 (quin, J = 7.6 Hz, 2H, CH_2), 1.35-1.17 (m, 6H, CH_2), 0.88 (t, J = 6.9 Hz, 3H, CH_3). $^{13}C\{^{1}H\}$-NMR (125 MHz, $CDCl_3$): δ = 151.8 (C_q), 149.8 (CH), 141.9 (C_q), 138.4 (CH), 137.4 (C_q), 128.8 (C_q), 122.1 (CH), 121.7 (CH), 121.3 (CH), 120.7 (CH), 120.0 (CH), 110.2 (CH), 102.2 (CH), 31.7 (CH_2), 29.1 (CH_2), 28.7 (CH_2), 27.6 (CH_2), 22.7 (CH_2), 14.2 (CH_3). HRMS (ESI): *m/z* Calcd for $C_{19}H_{22}N_2$+H^+ $[M+H]^+$ 279.1856; Found 279.1853. The ^{1}H and ^{13}C spectra are consistent with those reported in the literature.[S14]

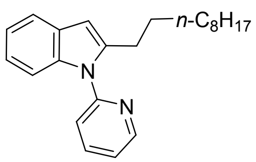

2-Decyl-1-(pyridin-2-yl)-1*H*-indole (3ad): The representative procedure was followed, using substrate **1a** (0.039 g, 0.20 mmol) and 1-chlorodecane (**2d**; 0.071 g, 0.40 mmol), and the reaction mixture was stirred for 5 h. Purification by column chromatography on neutral alumina (petroleum ether/EtOAc: 50/1) yielded **3ad** (0.058 g, 87%) as a light yellow liquid. ^{1}H-NMR (500 MHz, $CDCl_3$): δ = 8.68 (br s, 1H, Ar–H), 7.89 (t, J = 7.3 Hz, 1H, Ar–H), 7.64-7.54 (m, 1H, Ar–H), 7.43 (d, J = 7.9 Hz, 1H, Ar–H), 7.33 (d, J = 4.9 Hz, 2H, Ar–H), 7.21-7.07 (m, 2H, Ar–H), 6.46 (s, 1H, Ar–H), 2.85 (t, J = 7.6 Hz, 2H, CH_2), 1.66-1.54 (m, 2H, CH_2), 1.32-1.22 (m, 14H, CH_2), 0.95-0.85 (m, 3H, CH_3). $^{13}C\{^1H\}$-NMR (100 MHz, $CDCl_3$): δ = 151.7 (C_q), 149.8 (CH), 141.9 (C_q), 138.4 (CH), 137.4 (C_q), 128.8 (C_q), 122.2 (CH), 121.6 (CH), 121.3 (CH), 120.7 (CH), 120.0 (CH), 110.2 (CH), 102.2 (CH), 32.0 (CH_2), 29.7 (CH_2), 29.6 (CH_2), 29.5 (3C, CH_2), 28.7 (CH_2), 27.6 (CH_2), 22.8 (CH_2), 14.3 (CH_3). HRMS (ESI): *m/z* Calcd for $C_{23}H_{30}N_2+H^+$ $[M+H]^+$ 335.2482; Found 335.2479. The ^{1}H and ^{13}C spectra are consistent with those reported in the literature.[S14]

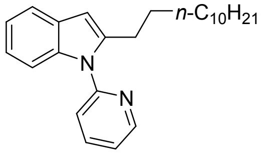

2-Dodecyl-1-(pyridin-2-yl)-1*H*-indole (3ae): The representative procedure was followed, using substrate **1a** (0.039 g, 0.20 mmol) and 1-chlorododecane (**2e**; 0.082 g, 0.40 mmol), and the reaction mixture was stirred for 5 h. Purification by column chromatography on neutral alumina (petroleum ether/EtOAc: 50/1) yielded **3ae** (0.064 g, 88%) as a light yellow liquid. ^{1}H-NMR

(500 MHz, $CDCl_3$): δ = 8.72-8.64 (m, 1H, Ar–H), 7.88 (td, J = 7.8, 1.9 Hz, 1H, Ar–H), 7.63-7.56 (m, 1H, Ar–H), 7.44 (d, J = 8.0 Hz, 1H, Ar–H), 7.38-7.29 (m, 2H, Ar–H), 7.18-7.10 (m, 2H, Ar–H), 6.48 (s, 1H, Ar–H), 2.86 (t, J = 7.4 Hz, 2H, CH_2), 1.64-1.54 (m, 2H, CH_2), 1.35-1.23 (m, 18H, CH_2), 0.92 (td, J = 6.9, 1.5 Hz, 3H, CH_3). $^{13}C\{^1H\}$-NMR (125 MHz, $CDCl_3$): δ = 151.8 (C_q), 149.8 (CH), 141.9 (C_q), 138.4 (CH), 137.4 (C_q), 128.8 (C_q), 122.1 (CH), 121.6 (CH), 121.3 (CH), 120.6 (CH), 120.0 (CH), 110.2 (CH), 102.2 (CH), 32.1 (CH_2), 29.8 (2C, CH_2), 29.6 (CH_2), 29.5 (3C, CH_2), 29.5 (CH_2), 28.7 (CH_2), 27.6 (CH_2), 22.8 (CH_2), 14.3 (CH_3). HRMS (ESI): *m/z* Calcd for $C_{25}H_{34}N_2$+H^+ $[M+H]^+$ 363.2795; Found 363.2791. The ^1H and ^{13}C spectra are consistent with those reported in the literature.[S14]

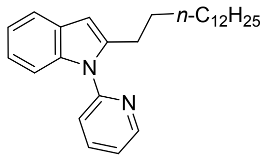

2-Tetradecyl-1-(pyridin-2-yl)-1*H*-indole (3af): The representative procedure was followed, using substrate **1a** (0.039 g, 0.20 mmol) and 1-chlorotetradecane (**2f**; 0.093 g, 0.40 mmol), and the reaction mixture was stirred for 5 h. Purification by column chromatography on neutral alumina (petroleum ether/EtOAc: 50/1) yielded **3af** (0.069 g, 88%) as a light yellow liquid. ^1H-NMR (500 MHz, $CDCl_3$): δ = 8.67 (d, J = 4.6 Hz, 1H, Ar–H), 7.92-7.86 (m, 1H, Ar–H), 7.61-7.56 (m, 1H, Ar–H), 7.43 (d, J = 8.0 Hz, 1H, Ar–H), 7.32 (dd, J = 7.4, 4.4 Hz, 2H, Ar–H), 7.19-7.06 (m, 2H, Ar–H), 6.45 (s, 1H, Ar–H), 2.84 (t, J = 7.6 Hz, 2H, CH_2), 1.62-1.50 (m, 2H, CH_2), 1.36-1.24 (m, 22H, CH_2), 0.90 (t, J = 6.9 Hz, 3H, CH_3). $^{13}C\{^1H\}$-NMR (125 MHz, $CDCl_3$): δ = 151.8 (C_q), 149.8 (CH), 142.0 (C_q), 138.4 (CH), 137.5 (C_q), 128.9 (C_q), 122.2 (CH), 121.7 (CH), 121.4 (CH), 120.7 (CH), 120.0 (CH), 110.2 (CH), 102.2 (CH), 32.1 (CH_2), 29.8 (5C, CH_2), 29.7 (CH_2), 29.5 (3C, CH_2), 28.8 (CH_2), 27.6 (CH_2), 22.9 (CH_2), 14.3 (CH_3). HRMS (ESI): *m/z* Calcd

for $C_{27}H_{38}N_2+H^+$ $[M+H]^+$ 391.3005; Found 391.2980. The ^1H and ^{13}C spectra are consistent with those reported in the literature.[S14]

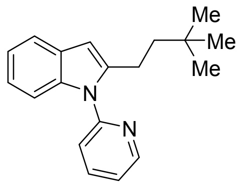

2-(3,3-Dimethylbutyl)-1-(pyridin-2-yl)-1*H*-indole (3ag): The representative procedure was followed, using substrate **1a** (0.039 g, 0.20 mmol), 1-chloro-3,3-dimethylbutane (**2g**; 0.048 g, 0.40 mmol) and the reaction mixture was stirred at 60 °C for 5 h. Purification by column chromatography on neutral alumina (petroleum ether/EtOAc: 50/1) yielded **3ag** (0.052 g, 93%) as a light yellow liquid. ^1H-NMR (400 MHz, $CDCl_3$): δ = 8.67 (dd, J = 4.6, 1.5 Hz, 1H, Ar–H), 7.89 (td, J = 7.6, 2.3 Hz, 1H, Ar–H), 7.57 (t, J = 4.6 Hz, 1H, Ar–H), 7.45 (d, J = 7.6 Hz, 1H, Ar–H), 7.34-7.31 (m, 2H, Ar–H), 7.14-7.11 (m, 2H, Ar-H), 6.45 (s, 1H, Ar–H), 2.85-2.81 (m, 2H, CH_2), 1.47-1.43 (m, 2H, CH_2), 0.84 (s, 9H, CH_3). $^{13}C\{^1H\}$-NMR (100 MHz, $CDCl_3$): δ = 151.6 (C_q), 149.7 (CH), 142.4 (C_q), 138.3 (CH), 137.3 (C_q), 128.7 (C_q), 122.1 (CH), 121.5 (CH), 121.2 (CH), 120.6 (CH), 119.9 (CH), 110.0 (CH), 102.0 (CH), 43.2 (CH_2), 30.4 (C_q), 29.2 (3C, CH_3), 22.9 (CH_2). HRMS (ESI): *m/z* Calcd for $C_{19}H_{22}N_2+H^+$ $[M+H]^+$ 279.1856; Found 279.1854.

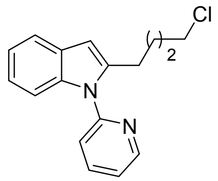

2-(4-Chlorobutyl)-1-(pyridin-2-yl)-1*H*-indole (3ah): The representative procedure was followed, using substrate **1a** (0.039 g, 0.20 mmol) and 1-bromo-4-chlorobutane (**2h**; 0.068 g, 0.40 mmol) or 1-iodo-4-chlorobutane (**2h'**; 0.087 g, 0.40 mmol), and the reaction mixture was stirred for 16 h. Purification by column chromatography on neutral alumina (petroleum ether/EtOAc: 50/1) yielded **3ah** {0.048 g, 84% (using **2h**) and 0.046 g, 81% (using **2h'**)} as a light yellow liquid. ^{1}H-NMR (500 MHz, $CDCl_3$): δ = 8.67 (dd, J = 4.8, 1.3 Hz, 1H, Ar–H), 7.90 (td, J = 7.6, 1.9 Hz, 1H, Ar–H), 7.63-7.55 (m, 1H, Ar–H), 7.45 (d, J = 8.0 Hz, 1H, Ar–H), 7.37-7.29 (m, 2H, Ar–H), 7.19-7.10 (m, 2H, Ar–H), 6.47 (s, 1H, Ar–H), 3.49 (t, J = 6.5 Hz, 2H, CH_2), 2.90 (t, J = 7.4 Hz, 2H, CH_2), 1.84-1.76 (m, 2H, CH_2), 1.76-1.68 (m, 2H, CH_2). ^{13}C{^{1}H}-NMR (125 MHz, $CDCl_3$): δ = 151.6 (C_q), 149.8 (CH), 140.9 (C_q), 138.5 (CH), 137.4 (C_q), 128.7 (C_q), 122.3 (CH), 121.9 (CH), 121.3 (CH), 120.8 (CH), 120.1 (CH), 110.2 (CH), 102.6 (CH), 44.9 (CH_2), 32.2 (CH_2), 26.8 (CH_2), 25.9 (CH_2). HRMS (ESI): *m/z* Calcd for $C_{17}H_{17}N_2Cl+H^+$ $[M+H]^+$ 285.1153; Found 285.1156.

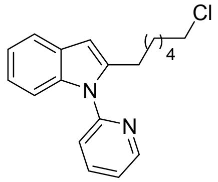

2-(6-Chlorohexyl)-1-(pyridin-2-yl)-1*H*-indole (3ai): The representative procedure was followed, using substrate **1a** (0.039 g, 0.20 mmol), 1,6-dichlorohexane (**2i**; 0.062 g, 0.40 mmol) and the reaction mixture was stirred at 60 ^{o}C for 24 h. Purification by column chromatography

on neutral alumina (petroleum ether/EtOAc: 50/1) yielded **3ai** (80%, ^{1}H NMR yield) as a light yellow liquid. ^{1}H-NMR (400 MHz, $CDCl_3$): δ = 8.67-8.66 (m, 1H, Ar–H), 7.89 (td, J = 7.6, 2.3 Hz, 1H, Ar–H), 7.58-7.56 (m, 1H, Ar–H), 7.44-7.42 (m, 1H, Ar–H), 7.34-7.30 (m, 2H, Ar–H), 7.13-7.11 (m, 2H, Ar-H), 6.44 (s, 1H, Ar–H), 3.48 (t, J = 6.9 Hz, 2H, CH_2), 2.85 (t, J = 7.6 Hz, 2H, CH_2), 1.74-1.67 (m, 2H, CH_2), 1.61-1.53 (m, 2H, CH_2), 1.40-1.30 (m, 4H, CH_2). ^{13}C{^{1}H}-NMR (100 MHz, $CDCl_3$): δ = 151.6 (C_q), 149.7 (CH), 141.5 (C_q), 138.4 (CH), 137.3 (C_q), 128.7 (C_q), 122.1 (CH), 121.7 (CH), 121.2 (CH), 120.6 (CH), 119.9 (CH), 110.14 (CH), 102.2 (CH), 45.1 (CH_2), 32.5 (CH_2), 28.5 (CH_2), 28.4 (CH_2), 27.3 (CH_2), 26.6 (CH_2). HRMS (ESI): *m/z* Calcd for $C_{19}H_{21}ClN_2+H^+$ $[M+H]^+$ 313.1466; Found 313.1464.

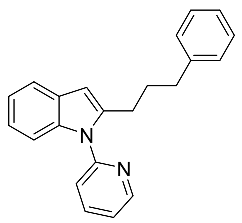

2-(3-Phenylpropyl)-1-(pyridin-2-yl)-1*H*-indole (3aj): The representative procedure was followed, using substrate **1a** (0.039 g, 0.20 mmol) and (3-chloropropyl)benzene (**2j**; 0.062 g, 0.40 mmol), and the reaction mixture was stirred for 16 h. Purification by column chromatography on neutral alumina (petroleum ether/EtOAc: 50/1) yielded **3aj** (0.060 g, 96%) as a light yellow liquid. ^{1}H-NMR (500 MHz, $CDCl_3$): δ = 8.71-8.61 (m, 1H, Ar–H), 7.86 (td, J = 7.8, 1.9 Hz, 1H, Ar–H), 7.65-7.57 (m, 1H, Ar–H), 7.42 (d, J = 8.0 Hz, 1H, Ar–H), 7.39-7.34 (m, 1H, Ar–H), 7.34-7.30 (m, 1H, Ar–H), 7.30-7.25 (m, 2H, Ar–H), 7.24-7.07 (m, 5H, Ar–H), 6.51 (s, 1H, Ar–H), 2.93 (t, J = 7.6 Hz, 2H, CH_2), 2.66 (t, J = 7.6 Hz, 2H, CH_2), 1.92 (quin, J = 7.6 Hz, 2H, CH_2). ^{13}C{^{1}H}-NMR (125 MHz, $CDCl_3$): δ = 151.6 (C_q), 149.8 (CH), 142.1 (C_q), 141.3 (C_q), 138.4 (CH), 137.4 (C_q), 128.8 (C_q), 128.6 (2C, CH), 128.4 (2C, CH), 125.9 (CH), 122.1

(CH), 121.8 (CH), 121.2 (CH), 120.8 (CH), 120.1 (CH), 110.2 (CH), 102.5 (CH), 35.5 (CH_2), 30.5 (CH_2), 27.2 (CH_2). HRMS (ESI): *m/z* Calcd for $C_{22}H_{20}N_2+H^+$ $[M+H]^+$ 313.1699; Found 313.1696. The ^1H and ^{13}C spectra are consistent with those reported in the literature.[S14]

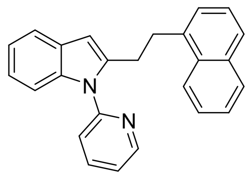

2-(2-(Naphthalen-1-yl)ethyl)-1-(pyridin-2-yl)-1*H*-indole (3ak): The representative procedure was followed using, substrate **1a** (0.039 g, 0.20 mmol), 1-(2-chloroethyl)naphthalene (**2k**; 0.076 g, 0.40 mmol) and the reaction mixture was stirred for 24 h. Purification by column chromatography on neutral alumina (petroleum ether/EtOAc: 50/1) yielded **3ak** (0.052 g, 75%) as a pale yellow solid. ^1H-NMR (500 MHz, $CDCl_3$): δ = 8.65 (d, J = 3.8 Hz, 1H, Ar–H), 7.90 (d, J = 8.4 Hz, 1H, Ar–H), 7.87-7.83 (m, 1H, Ar–H), 7.83-7.77 (m, 1H, Ar–H), 7.71 (d, J = 8.0 Hz, 1H, Ar–H), 7.69-7.61 (m, 1H, Ar–H), 7.47 (m, 2H, Ar–H), 7.40-7.32 (m, 3H, Ar–H), 7.31-7.22 (m, 2H, Ar–H), 7.22-7.13 (m, 2H, Ar–H), 6.64 (s, 1H, Ar–H), 3.46-3.37 (m, 2H, CH_2), 3.36-3.27 (m, 2H, CH_2). $^{13}C\{^1H\}$-NMR (125 MHz, $CDCl_3$): δ = 151.5 (C_q), 149.8 (CH), 141.2 (C_q), 138.4 (CH), 137.7 (C_q), 137.4 (C_q), 134.0 (C_q), 131.9 (C_q), 128.9 (CH), 128.8 (C_q), 127.0 (CH), 126.3 (CH), 126.0 (CH), 125.7 (CH), 125.6 (CH), 123.8 (CH), 122.2 (CH), 122.0 (CH), 121.3 (CH), 120.9 (CH), 120.2 (CH), 110.2 (CH), 102.7 (CH), 33.2 (CH_2), 29.0 (CH_2). HRMS (ESI): *m/z* Calcd for $C_{25}H_{20}N_2+H^+$ $[M+H]^+$ 348.1699; Found 348.1696.

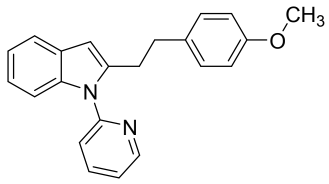

2-(4-Methoxyphenethyl)-1-(pyridin-2-yl)-1*H*-indole (3al): The representative procedure was followed using, substrate **1a** (0.039 g, 0.20 mmol), 1-(2-chloroethyl)-4-methoxybenzene (**2l**; 0.068 g, 0.40 mmol) and the reaction mixture was stirred for 16 h. Purification by column chromatography on neutral alumina (petroleum ether/EtOAc: 20/1) yielded **3al** (0.053 g, 81%) as a white solid. ^{1}H-NMR (400 MHz, $CDCl_3$): δ = 8.69 (d, J = 3.7 Hz, 1H, Ar–H), 7.88 (t, J = 7.3 Hz, 1H, Ar–H), 7.72-7.55 (m, 1H, Ar–H), 7.42 (d, J = 7.9 Hz, 1H, Ar–H), 7.40-7.28 (m, 2H, Ar–H), 7.23-7.13 (m, 2H, Ar–H), 7.06 (d, J = 8.5 Hz, 2H, Ar–H), 6.82 (d, J = 7.9 Hz, 2H, Ar–H), 6.53 (s, 1H, Ar–H), 3.79 (s, 3H, OCH_3), 3.17 (t, J = 7.9 Hz, 2H, CH_2), 2.97-2.83 (m, 2H, CH_2). $^{13}C\{^1H\}$-NMR (100 MHz, $CDCl_3$): δ = 158.0 (C_q), 151.6 (C_q), 149.8 (CH), 141.0 (C_q), 138.4 (CH), 137.3 (C_q), 133.7 (C_q), 129.4 (2C, CH), 128.7 (C_q), 122.2 (CH), 121.9 (CH), 121.2 (CH), 120.8 (CH), 120.2 (CH), 113.9 (2C, CH), 110.2 (CH), 102.6 (CH), 55.3 (OCH_3), 34.5 (CH_2), 30.0 (CH_2). HRMS (ESI): *m/z* Calcd for $C_{22}H_{20}N_2O$+H^+ $[M+H]^+$ 329.1648; Found 329.1645.

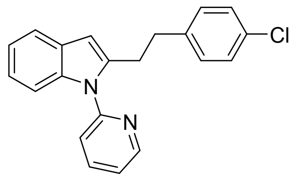

2-(4-Chlorophenethyl)-1-(pyridin-2-yl)-1*H*-indole (3am): The representative procedure was followed using, substrate **1a** (0.039 g, 0.20 mmol), 1-chloro-4-(2-chloroethyl)benzene (**2m**; 0.070 g, 0.40 mmol) and the reaction mixture was stirred for 16 h. Purification by column

chromatography on neutral alumina (petroleum ether/EtOAc: 30/1) yielded **3am** (0.041 g, 62%) as a pale yellow solid. ^{1}H-NMR (400 MHz, $CDCl_3$): δ = 8.69 (br s, 1H, Ar–H), 7.89 (br s, 1H, Ar–H), 7.62 (br s, 1H, Ar–H), 7.51-7.30 (m, 3H, Ar–H), 7.30-7.12 (m, 4H, Ar–H), 7.03 (br s, 2H, Ar–H), 6.50 (br s, 1H, Ar–H), 3.19 (br s, 2H, CH_2), 3.07-2.79 (m, 2H, CH_2). $^{13}C\{^1H\}$-NMR (100 MHz, $CDCl_3$): δ = 151.3 (C_q), 149.6 (CH), 140.5 (C_q), 139.9 (C_q), 138.7 (CH), 137.3 (C_q), 131.8 (C_q), 129.9 (2C, CH), 128.7 (C_q), 128.5 (2C, CH), 122.2 (CH), 122.0 (CH), 121.2 (CH), 120.9 (CH), 120.2 (CH), 110.2 (CH), 102.9 (CH), 34.8 (CH_2), 29.6 (CH_2). HRMS (ESI): *m/z* Calcd for $C_{21}H_{17}ClN_2$+H^+ $[M+H]^+$ 333.1153; Found 333.1151.

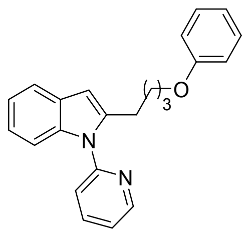

2-(4-Phenoxybutyl)-1-(pyridin-2-yl)-1*H*-indole (3an): The representative procedure was followed, using substrate **1a** (0.039 g, 0.20 mmol) and (4-chlorobutoxy)benzene (**2n**; 0.074 g, 0.40 mmol), and the reaction mixture was stirred for 24 h. Purification by column chromatography on neutral alumina (petroleum ether/EtOAc: 20/1) yielded **3an** (0.049 g, 72%) as a light yellow liquid. ^{1}H-NMR (400 MHz, $CDCl_3$): δ = 8.63 (d, J = 3.7 Hz, 1H, Ar–H), 7.99-7.77 (m, 1H, Ar–H), 7.69-7.52 (m, 1H, Ar–H), 7.43 (d, J = 7.9 Hz, 1H, Ar–H), 7.38-7.20 (m, 4H, Ar–H), 7.20-7.05 (m, 2H, Ar–H), 6.92 (t, J = 7.0 Hz, 1H, Ar–H), 6.84 (d, J = 7.9 Hz, 2H, Ar–H), 6.48 (s, 1H, Ar–H), 3.90 (t, J = 5.5 Hz, 2H, CH_2), 2.94 (t, J = 7.0 Hz, 2H, CH_2), 1.83-1.67 (m, 4H, CH_2). $^{13}C\{^1H\}$-NMR (125 MHz, $CDCl_3$): δ = 159.1 (C_q), 151.7 (C_q), 149.8 (CH), 141.2 (C_q), 138.4 (CH) 137.4 (C_q), 129.5 (2C, CH), 128.7 (C_q), 122.2 (CH), 121.8 (CH), 121.2 (CH), 120.8 (CH), 120.7 (CH), 120.1 (CH), 114.6 (2C, CH), 110.2 (CH), 102.5 (CH), 67.5 (CH_2), 29.0

(CH_2), 27.3 (CH_2), 25.3 (CH_2). HRMS (ESI): *m/z* Calcd for $C_{23}H_{22}N_2O+H^+$ $[M+H]^+$ 343.1805; Found 343.1803.

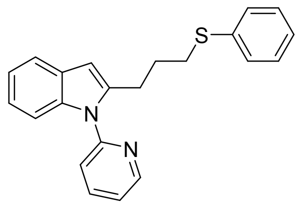

2-(3-(Phenylthio)propyl)-1-(pyridin-2-yl)-1*H*-indole (3ao): The representative procedure was followed, using substrate **1a** (0.039 g, 0.20 mmol), (3-chloropropyl)(phenyl)sulfane (**2o**; 0.075 g, 0.40 mmol) and the reaction mixture was stirred at 80 °C for 24 h. Purification by column chromatography on neutral alumina (petroleum ether/EtOAc: 10/1) yielded **3ao** (0.038 g, 55%) as a light yellow liquid. ^{1}H-NMR (500 MHz, $CDCl_3$): δ= 8.67 (d, J = 3.4 Hz, 1H, Ar–H), 7.88 (td, J = 7.6, 1.9 Hz, 1H, Ar–H), 7.62 (m, 1H, Ar–H), 7.45 (d, J = 8.0 Hz, 1H, Ar–H), 7.38-7.31 (m, 2H, Ar–H), 7.30-7.29 (m, 4H, Ar-H), 7.22-7.18 (m, 3H, Ar–H), 6.50 (s, 1H, Ar–H), 3.05 (t, J = 7.6 Hz, 2H, CH_2), 2.96 (t, J = 7.2 Hz, 2H, CH_2), 1.99-1.93 (m, 2H, CH_2). $^{13}C\{^1H\}$-NMR (125 MHz, $CDCl_3$): δ= 151.4 (C_q), 149.7 (CH), 140.2 (C_q), 138.3 (CH), 137.3 (C_q), 136.5 (C_q), 129.1 (2C, CH), 128.9 (2C, CH), 128.6 (C_q), 125.8 (CH), 122.1 (CH), 121.8 (CH), 121.0 (CH), 120.0 (CH), 121.0 (CH), 110.12 (CH), 102.7 (CH), 33.0 (CH_2), 28.1 (CH_2), 26.4 (CH_2). HRMS (ESI): *m/z* Calcd for $C_{22}H_{20}N_2S+H^+$ $[M+H]^+$ 345.1420; Found 345.1420.

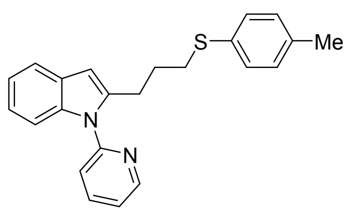

1-(Pyridin-2-yl)-2-(3-(*p*-tolylthio)propyl)-1*H*-indole (3ap): The representative procedure was followed, using substrate **1a** (0.039 g, 0.20 mmol), (3-chloropropyl)(p-tolyl)sulfane (**2p**; 0.080 g, 0.40 mmol) and the reaction mixture was stirred at 80 °C for 24 h. Purification by column chromatography on neutral alumina (petroleum ether/EtOAc: 10/1) yielded **3ap** (0.044 g, 61%) as a light yellow liquid. ^{1}H-NMR (500 MHz, $CDCl_3$): δ= 8.66 (br s, 1H, Ar–H), 7.89 (t, J = 7.6 Hz, 1H, Ar–H), 7.60 (m, 1H, Ar–H), 7.44 (d, J = 7.6 Hz, 1H, Ar–H), 7.35-7.32 (m, 2H, Ar–H), 7.22 (m, 2H, Ar-H), 7.16 (m, 2H, Ar–H), 7.09 (d, J = 7.6 Hz, 2H, Ar-H), 6.46 (s, 1H, Ar–H), 3.03 (t, J = 7.2 Hz, 2H, CH_2), 2.90 (t, J = 7.2 Hz, 2H, CH_2), 2.35 (s, 3H, CH_3), 1.93-1.90 (m, 2H, CH_2). $^{13}C\{^1H\}$-NMR (125 MHz, $CDCl_3$): δ= 151.4 (C_q), 149.6 (CH), 140.2 (C_q), 138.2 (CH), 137.3 (C_q), 136.0 (C_q), 132.5 (C_q), 130.0 (2C, CH), 129.6 (2C, CH), 128.5 (C_q), 122.0 (CH), 121.7 (CH), 121.0 (CH), 120.6 (CH), 119.9 (CH), 110.0 (CH), 102.6 (CH), 33.8 (CH_2), 28.2 (CH_2), 26.4 (CH_2), 21.0 (CH_3). HRMS (ESI): *m/z* Calcd for $C_{23}H_{22}N_2S+H^+$ $[M+H]^+$ 359.1576; Found 359.1575.

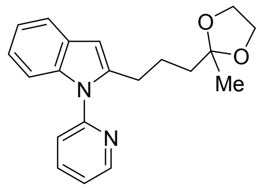

2-(3-(2-Methyl-1,3-dioxolan-2-yl)propyl)-1-(pyridin-2-yl)-1*H*-indole (3aq): The representative procedure was followed, using substrate **1a** (0.039 g, 0.20 mmol) and 2-(3-chloropropyl)-2-methyl-1,3-dioxolane (**2q**; 0.066 g, 0.40 mmol), and the reaction mixture was

stirred for 16 h. Purification by column chromatography on neutral alumina (petroleum ether/EtOAc: 50/1) yielded **3aq** (0.027 g, 42%) as a light yellow liquid. ^{1}H-NMR (500 MHz, $CDCl_3$): δ = 8.72-8.54 (m, 1H, Ar–H), 7.89 (br s, 1H, Ar–H), 7.64-7.52 (m, 1H, Ar–H), 7.44 (d, J = 7.6 Hz, 1H, Ar–H), 7.38-7.28 (m, 2H, Ar–H), 7.17-7.03 (m, 2H, Ar–H), 6.48 (s, 1H, Ar–H), 4.01-3.77 (m, 4H, CH_2), 2.86 (d, J = 6.9 Hz, 2H, CH_2), 1.68 (br s, 4H, CH_2), 1.27 (s, 3H, CH_3). ^{13}C{^{1}H}-NMR (125 MHz, $CDCl_3$): δ = 151.7 (C_q), 149.8 (CH), 141.4 (C_q), 138.4 (CH), 137.4 (C_q), 128.8 (C_q), 122.2 (CH), 121.7 (CH), 121.3 (CH), 120.7 (CH), 120.1 (CH), 110.2 (CH), 110.1 (C_q), 102.4 (CH), 64.8 (2C, CH_2), 38.8 (CH_2), 27.7 (CH_2), 24.0 (CH_3), 23.1 (CH_2). HRMS (ESI): *m/z* Calcd for $C_{20}H_{22}O_2N_2$+Na^+ $[M+Na]^+$ 345.1573; Found 345.1567.

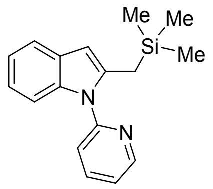

1-(Pyridin-2-yl)-2-((trimethylsilyl)methyl)-1*H*-indole (3ar): The representative procedure was followed, using substrate **1a** (0.039 g, 0.20 mmol) and (chloromethyl)trimethylsilane (**2r**; 0.049 g, 0.40 mmol), and the reaction mixture was stirred for 16 h. Purification by column chromatography on neutral alumina (petroleum ether/EtOAc: 50/1) yielded **3ar** (0.031 g, 55%) as a light yellow liquid. ^{1}H-NMR (400 MHz, $CDCl_3$): δ = 8.68 (dt, J = 5.0, 0.9 Hz, 1H, Ar–H), 7.88 (td, J = 7.8, 1.8 Hz, 1H, Ar–H), 7.54 (d, J = 7.8 Hz, 1H, Ar–H), 7.47-7.41 (m, 1H, Ar–H), 7.35-7.28 (m, 2H, Ar–H), 7.17-7.06 (m, 2H, Ar–H), 6.31 (s, 1H, Ar–H), 2.46 (s, 2H, CH_2), –0.09 (s, 9H, CH_3). ^{13}C{^{1}H}-NMR (100 MHz, $CDCl_3$): δ = 151.85 (C_q), 149.6 (CH), 139.8 (C_q), 138.2 (CH), 137.2 (C_q), 129.3 (C_q), 122.0 (CH), 121.7 (CH), 120.9 (CH), 120.8 (CH), 119.3 (CH),

110.1 (CH), 101.7 (CH), 17.5 (CH_2), −1.3 (3C, CH_3). HRMS (ESI): *m/z* Calcd for $C_{17}H_{20}N_2Si+H^+$ $[M+H]^+$ 281.1469; Found 281.1471.

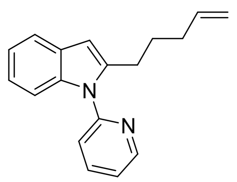

2-(Pent-4-en-1-yl)-1-(pyridin-2-yl)-1*H*-indole (3as): The representative procedure was followed using, substrate **1a** (0.039 g, 0.20 mmol), 5-chloropent-1-ene (**2s**; 0.042 g, 0.40 mmol) and the reaction mixture was stirred for 16 h. Purification by column chromatography on neutral alumina (petroleum ether/EtOAc: 50/1) yielded **3as** (0.049 g, 93%) as a pale yellow solid. ^{1}H-NMR (500 MHz, $CDCl_3$): δ = 8.68 (d, J = 4.6 Hz, 1H, Ar–H), 7.89 (t, J = 7.6 Hz, 1H, Ar–H), 7.66-7.55 (m, 1H, Ar–H), 7.45 (d, J = 7.6 Hz, 1H, Ar–H), 7.40-7.27 (m, 2H, Ar–H), 7.07-7.20 (m, 2H, Ar–H), 6.49 (s, 1H, Ar–H), 5.85-5.70 (m, 1H, CH), 4.89-5.06 (m, 2H, CH_2), 2.89 (t, J = 7.6 Hz, 2H, CH_2), 2.10 (quin, J = 6.9 Hz, 2H, CH_2), 1.69 (quin, J = 7.5 Hz, 2H, CH_2). ^{13}C{^{1}H}-NMR (125 MHz, $CDCl_3$): δ = 151.6 (C_q), 149.8 (CH), 141.4 (C_q), 138.5 (CH), 138.4 (CH), 137.4 (C_q), 128.7 (C_q), 122.2 (CH), 121.7 (CH), 121.2 (CH), 120.7 (CH), 120.0 (CH), 115.0 (CH_2), 110.2 (CH), 102.4 (CH), 33.4 (CH_2), 27.9 (CH_2), 27.0 (CH_2). HRMS (ESI): *m/z* Calcd for $C_{18}H_{18}N_2+H^+$ $[M+H]^+$ 263.1543; Found 263.1541. The ^{1}H and ^{13}C spectra are consistent with those reported in the literature.[S14]

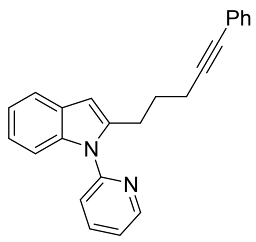

2-(5-Phenylpent-4-yn-1-yl)-1-(pyridin-2-yl)-1*H*-indole (3at): The representative procedure was followed, using substrate **1a** (0.039 g, 0.20 mmol) and (5-chloropent-1-yn-1-yl)benzene (**2t**; 0.071 g, 0.40 mmol), and the reaction mixture was stirred for 16 h. Purification by column chromatography on neutral alumina (petroleum ether/EtOAc: 20/1) yielded **3at** (0.035 g, 52%) as a light yellow liquid. ^{1}H-NMR (400 MHz, $CDCl_3$): δ = 8.76-8.53 (m, 1H, Ar–H), 7.85 (td, J = 7.6, 1.8 Hz, 1H, Ar–H), 7.66-7.54 (m, 1H, Ar–H), 7.49-7.39 (m, 1H, Ar–H), 7.39-7.30 (m, 3H, Ar–H), 7.30-7.21 (m, 4H, Ar–H), 7.20-7.06 (m, 2H, Ar–H), 6.51 (s, 1H, Ar–H), 3.04 (t, J = 7.6 Hz, 2H, CH_2), 2.43 (t, J = 6.9 Hz, 2H, CH_2), 1.97-1.75 (m, 2H, CH_2). $^{13}C\{^1H\}$-NMR (100 MHz, $CDCl_3$): δ = 151.6 (C_q), 149.9 (CH), 140.6 (C_q), 138.5 (CH), 137.4 (C_q), 131.7 (2C, CH), 128.7 (C_q), 128.3 (2C, CH), 127.7 (CH), 124.0 (C_q), 122.3 (CH), 121.9 (CH), 121.3 (CH), 120.8 (CH), 120.1 (CH), 110.3 (CH), 102.8 (CH), 89.8 (C_q), 81.3 (C_q), 29.9 (CH_2), 28.0 (CH_2), 26.7 (CH_2). HRMS (ESI): *m/z* Calcd for $C_{24}H_{20}N_2+H^+$ $[M+H]^+$ 337.1699; Found 337.1699.

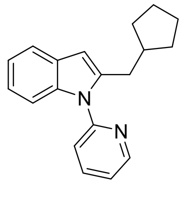

2-(Cyclopentylmethyl)-1-(pyridin-2-yl)-1*H*-indole (3au): The representative procedure was followed, using substrate **1a** (0.039 g, 0.20 mmol), 6-chloro-1-hexene (**2u**; 0.047 g, 0.40 mmol) and the reaction mixture was stirred for 16 h. Purification by column chromatography on neutral alumina (petroleum ether/EtOAc: 50/1) yielded **3au** (0.042 g, 76%) as a light yellow liquid. ^{1}H-

NMR (400 MHz, $CDCl_3$): δ= 8.87-8.57 (m, 1H, Ar–H), 7.89 (t, J = 7.3 Hz, 1H, Ar–H), 7.59 (d, J = 4.9 Hz, 1H, Ar–H), 7.43 (d, J = 7.9 Hz, 1H, Ar–H), 7.39-7.28 (m, 2H, Ar–H), 7.23-7.07 (m, 2H, Ar–H), 6.48 (s, 1H, Ar–H), 2.86 (d, J = 6.7 Hz, 2H, CH_2), 2.22-1.93 (m, 1H, CH), 1.73 (d, J = 6.7 Hz, 2H, CH_2), 1.66-1.47 (m, 4H, CH_2), 1.25-1.00 (m, 2H, CH_2). $^{13}C\{^1H\}$-NMR (125 MHz, $CDCl_3$): δ= 151.8 (C_q), 149.8 (CH), 141.4 (C_q), 138.4 (CH), 137.4 (C_q), 128.8 (C_q), 122.2 (CH), 121.6 (CH), 121.4 (CH), 120.7 (CH), 120.0 (CH), 110.2 (CH), 102.9 (CH), 39.3 (CH), 33.8 (CH_2), 32.8 (2C, CH_2), 25.2 (2C, CH_2). HRMS (ESI): *m/z* Calcd for $C_{19}H_{20}N_2$+H^+ $[M+H]^+$ 277.1699; Found 277.1700. The ^1H and ^{13}C spectra are consistent with those reported in the literature.[S14]

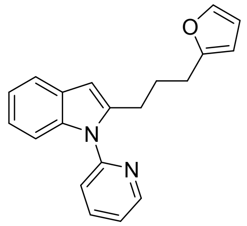

2-(3-(Furan-2-yl)propyl)-1-(pyridin-2-yl)-1*H*-indole (3av): The representative procedure was followed, using substrate **1a** (0.039 g, 0.20 mmol), 2-(3-chloropropyl)furan (**2v**; 0.058 g, 0.40 mmol) and the reaction mixture was stirred at 80 °C for 16 h. Purification by column chromatography on neutral alumina (petroleum ether/EtOAc: 5/1) yielded **3av** (60%; ^1H NMR yield using CH_2Br_2 as internal standard) as a light yellow liquid. ^1H-NMR (400 MHz, $CDCl_3$): δ= 8.64 (dd, J = 5.3, 2.3 Hz, 1H, Ar–H), 7.90-7.86 (td, J = 7.6, 1.5 Hz, 1H, Ar–H), 7.58 (t, J = 4.6 Hz, 1H, Ar–H), 7.42 (d, J = 7.6 Hz, 1H, Ar–H), 7.34-7.30 (m, 2H, Ar–H), 7.27-2.25 (m, 1H, Ar-H), 7.16-7.11 (m, 2H, Ar-H), 6.49 (s, 1H, Ar–H), 6.26-6.25 (m, 1H, Ar-H), 5.93 (d, J = 3.0 Hz, 1H, Ar-H), 2.90 (t, J = 7.6 Hz, 2H, CH_2), 2.64 (t, J = 7.6 Hz, 2H, CH_2), 1.91 (pent, J = 7.6 Hz, 2H, CH_2). $^{13}C\{^1H\}$-NMR (100 MHz, $CDCl_3$): δ= 155.7 (C_q), 151.5 (C_q), 149.7 (CH), 140.9

(C_q), 140.9 (CH), 138.4 (CH), 137.3 (C_q), 128.7 (C_q), 122.1 (CH), 121.7 (CH), 121.1 (CH), 120.7 (CH), 120.0 (CH), 110.2 (CH), 110.1 (CH), 105.0 (CH), 102.5 (CH), 27.5 (CH_2), 27.1 (CH_2), 26.9 (CH_2). HRMS (ESI): *m/z* Calcd for $C_{20}H_{18}N_2O+H^+$ $[M+H]^+$ 303.1492; Found 303.1490.

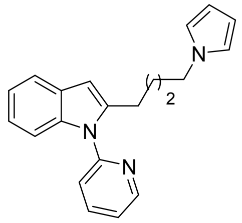

2-(4-(1*H*-Pyrrol-1-yl)butyl)-1-(pyridin-2-yl)-1*H*-indole (3aw): The representative procedure was followed, using substrate **1a** (0.039 g, 0.20 mmol) and 1-(4-chlorobutyl)-1*H*-pyrrole (**2w**; 0.063 g, 0.40 mmol), and the reaction mixture was stirred for 16 h. Purification by column chromatography on neutral alumina (petroleum ether/EtOAc: 20/1) yielded **3aw** (0.053 g, 84%) as a light yellow liquid. ^{1}H-NMR (400 MHz, $CDCl_3$): δ = 8.66 (br s, 1H, Ar–H), 8.01-7.79 (m, 1H, Ar–H), 7.79-7.61 (m, 2H, Ar–H), 7.46-7.28 (m, 3H, Ar–H), 7.28-7.15 (m, 3H, Ar–H), 7.08 (br s, 1H, Ar–H), 6.53 (br s, 1H, Ar–H), 6.47 (br s, 1H, Ar–H), 4.23-3.99 (m, 2H, CH_2), 3.14-2.84 (m, 2H, CH_2), 2.03-1.81 (m, 2H, CH_2), 1.63 (d, J = 7.3 Hz, 2H, CH_2). $^{13}C\{^1H\}$-NMR (100 MHz, $CDCl_3$): δ = 151.5 (C_q), 149.7 (CH), 140.9 (C_q), 138.5 (CH), 137.3 (C_q), 128.6 (C_q), 122.2 (CH), 121.8 (CH), 121.2 (CH), 120.8 (CH), 120.5 (2C, CH), 120.0 (CH), 110.2 (CH), 108.0 (2C, CH), 102.5 (CH), 49.3 (CH_2), 31.1 (CH_2), 27.0 (CH_2), 25.8 (CH_2). HRMS (ESI): *m/z* Calcd for $C_{21}H_{21}N_3+H^+$ $[M+H]^+$ 316.1808; Found 316.1806.

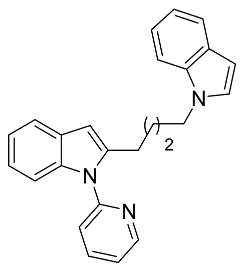

2-(4-(1*H*-Indol-1-yl)butyl)-1-(pyridin-2-yl)-1*H*-indole (3ax): The representative procedure was followed, using substrate **1a** (0.039 g, 0.20 mmol) and 1-(4-chlorobutyl)-1*H*-indole (**2x**; 0.083 g, 0.40 mmol), and the reaction mixture was stirred for 16 h. Purification by column chromatography on neutral alumina (petroleum ether/EtOAc: 20/1) yielded **3ax** (73%; ^{1}H NMR yield using CH_2Br_2 as internal standard) as a light yellow liquid. ^{1}H-NMR (400 MHz, $CDCl_3$): δ = 8.65 (d, J = 3.7 Hz, 1H, Ar–H), 7.95-7.78 (m, 1H, Ar–H), 7.70 (d, J = 7.3 Hz, 1H, Ar–H), 7.67-7.58 (m, 1H, Ar–H), 7.42-7.28 (m, 4H, Ar–H), 7.28-7.12 (m, 4H, Ar–H), 7.07 (d, J = 3.1 Hz, 1H, Ar–H), 6.52 (d, J = 2.4 Hz, 1H, Ar–H), 6.46 (s, 1H, Ar–H), 4.11 (t, J = 7.0 Hz, 2H, Ar–H), 2.93 (t, J = 7.6 Hz, 2H, CH_2), 2.02-1.80 (m, 2H, CH_2), 1.70-1.54 (m, 2H, CH_2). $^{13}C\{^1H\}$-NMR (125 MHz, $CDCl_3$): δ = 151.4 (C_q), 149.7 (CH), 140.8 (C_q), 138.4 (CH), 137.4 (C_q), 136.0 (C_q), 128.7 (C_q), 128.6 (C_q), 127.9 (CH), 122.2 (CH), 121.9 (CH), 121.4 (CH), 121.1 (CH), 121.1 (CH), 120.8 (CH), 120.1 (CH), 119.3 (CH), 110.2 (CH), 109.5 (CH), 102.5 (CH), 101.0 (CH), 46.2 (CH_2), 29.8 (CH_2), 27.1 (CH_2), 26.0 (CH_2). HRMS (ESI): *m/z* Calcd for $C_{25}H_{23}N_3+H^+$ $[M+H]^+$ 366.1965; Found 366.1967.

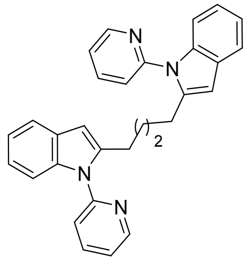

1,4-Bis(1-(pyridin-2-yl)-1*H*-indol-2-yl)butane (3ay): The representative procedure was followed, using substrate **1a** (0.039 g, 0.20 mmol) and 2-(4-chlorobutyl)-1-(pyridin-2-yl)-1*H*-indole (**2y**, 0.114 g, 0.40 mmol), and the reaction mixture was stirred for 16 h. Purification by column chromatography on neutral alumina (petroleum ether/EtOAc: 10/1) yielded **3ay** (0.058 g, 65%) as a light yellow solid. ^{1}H-NMR (500 MHz, $CDCl_3$): δ = 8.56 (dd, *J* = 5.0, 0.8 Hz, 2H, Ar–H), 7.75 (td, *J* = 7.7, 2.1 Hz, 2H, Ar–H), 7.59-7.50 (m, 2H, Ar–H) 7.34 (d, *J* = 8.0 Hz, 2H, Ar–H), 7.31-7.27 (m, 2H, Ar–H), 7.27-7.21 (m, 2H, Ar–H), 7.16-7.08 (m, 4H, Ar–H), 6.34 (s, 2H, Ar–H), 2.81 (br s, 4H, CH_2), 1.55-1.48 (m, 4H, CH_2). $^{13}C\{^1H\}$-NMR (125 MHz, $CDCl_3$): δ = 151.6 (2C, C_q), 149.7 (2C, CH), 141.3 (2C, C_q), 138.4 (2C, CH), 137.4 (2C, C_q), 128.7 (2C, C_q), 122.1 (2C, CH), 121.7 (2C, CH), 121.2 (2C, CH), 120.7 (2C, CH), 120.0 (2C, CH), 110.2 (2C, CH), 102.4 (2C, CH), 28.1 (2C, CH_2), 27.3 (2C, CH_2). HRMS (ESI): *m/z* Calcd for $C_{30}H_{26}N_4+H^+$ $[M+H]^+$ 443.2230; Found 443.2235.

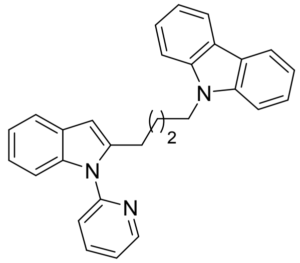

9-(4-(1-(Pyridin-2-yl)-1*H*-indol-2-yl)butyl)-9*H*-carbazole (3az): The representative procedure was followed, using substrate **1a** (0.039 g, 0.20 mmol) and 9-(4-chlorobutyl)-8a,9a-dihydro-9*H*-carbazole (**2z**; 0.103 g, 0.40 mmol), and the reaction mixture was stirred for 24 h. Purification by column chromatography on neutral alumina (petroleum ether/EtOAc: 10/1) yielded **3az** (84%; ^{1}H NMR yield using CH_2Br_2 as internal standard) as a pale yellow liquid. ^{1}H-NMR (500 MHz, $CDCl_3$): δ = 8.71-8.49 (m, 1H, Ar–H), 8.19 (d, J = 7.6 Hz, 2H, Ar–H), 7.76 (t, J = 7.4 Hz, 1H, Ar–H), 7.66 (d, J = 6.9 Hz, 1H, Ar–H), 7.52 (t, J = 7.4 Hz, 2H, Ar–H), 7.43-7.36 (m, 3H, Ar–H), 7.35-7.27 (m, 3H, Ar–H), 7.28- 7.16 (m, 3H, Ar–H), 6.46 (s, 1H, Ar–H), 4.30 (t, J = 6.7 Hz, 2H, CH_2), 2.95 (t, J = 7.2 Hz, 2H, CH_2), 2.03-1.87 (m, 2H, CH_2), 1.73-1.57 (m, 2H, CH_2). $^{13}C\{^1H\}$-NMR (125 MHz, $CDCl_3$): δ = 151.4 (C_q), 149.6 (CH), 140.8 (C_q), 140.5 (2C, C_q), 138.3 (CH), 137.3 (C_q), 128.6 (C_q), 125.7 (2C, CH), 122.9 (2C, C_q), 122.1 (CH), 121.8 (CH), 120.9 (CH), 120.8 (CH), 120.4 (2C, CH), 120.1 (CH), 118.9 (2C, CH), 110.1 (CH), 108.8 (2C, CH), 102.5 (CH), 42.8 (CH_2), 28.7 (CH_2), 27.3 (CH_2), 26.3 (CH_2). HRMS (ESI): *m/z* Calcd for $C_{29}H_{25}N_3+H^+$ $[M+H]^+$ 416.2121; Found 416.2121. The ^{1}H and ^{13}C spectra are consistent with those reported in the literature.[S14]

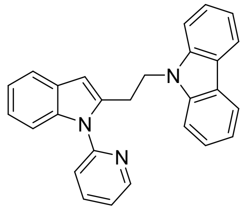

9-(4-(1-(Pyridin-2-yl)-1*H*-indol-2-yl)ethyl)-9*H*-carbazole (3aA): The representative procedure was followed, using substrate **1a** (0.039 g, 0.20 mmol) and 9-(2-chloroethyl)-9*H*-carbazole (**2A**; 0.092 g, 0.40 mmol), and the reaction mixture was stirred for 24 h. Purification by column chromatography on neutral alumina (petroleum ether/EtOAc: 20/1) yielded **3aA** (0.031 g, 40%) as a light yellow liquid. ^{1}H-NMR (500 MHz, $CDCl_3$): δ = 8.59 (d, J = 3.8 Hz, 1H, Ar–H), 8.06 (d, J = 7.6 Hz, 2H, Ar–H), 7.71 (s, 1H, Ar–H), 7.67-7.58 (m, 1H, Ar–H), 7.45-7.34 (m, 2H, Ar–H), 7.31 (d, J = 7.2 Hz, 1H, Ar–H), 7.25-7.14 (m, 7H, Ar–H), 7.09 (d, J = 8.0 Hz, 1H, Ar–H), 6.66 (s, 1H, Ar–H), 4.61 (t, J = 7.6 Hz, 2H, CH_2), 3.46 (t, J = 7.4 Hz, 2H, CH_2). ^{13}C{^{1}H}-NMR (125 MHz, $CDCl_3$): δ = 151.1 (C_q), 149.6 (CH), 140.2 (2C, C_q), 138.5 (CH), 138.2 (C_q), 137.3 (C_q), 128.7 (C_q), 125.7 (2C, CH), 123.1 (2C, C_q), 122.3 (CH), 122.0 (CH), 121.1 (CH), 120.7 (CH), 120.4 (CH), 120.4 (2C, CH), 119.1 (2C, CH), 110.3 (CH), 108.6 (2C, CH), 103.9 (CH), 43.9 (CH_2), 27.0 (CH_2). HRMS (ESI): *m/z* Calcd for $C_{27}H_{22}N_2+H^+$ $[M+H]^+$ 388.1808; Found 388.1810.

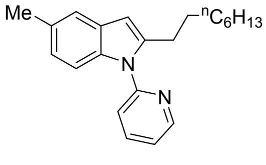

2-Octyl-1-(5-methylpyridin-2-yl)-1*H*-indole (3ba): The representative procedure was followed, using substrate **1b** (0.042 g, 0.20 mmol) and 1-chlorooctane (**2a**; 0.059 g, 0.40 mmol), and the reaction mixture was stirred for 5 h. Purification by column chromatography on neutral

alumina (petroleum ether/EtOAc: 50/1) yielded **3ba** (0.055 g, 86%) as a light yellow liquid. ^{1}H-NMR (400 MHz, $CDCl_3$): δ = 8.69 (d, J = 4.2 Hz, 1H, Ar–H), 7.98-7.85 (m, 1H, Ar–H), 7.50-7.38 (m, 2H, Ar–H), 7.33 (dd, J = 7.0, 5.2 Hz, 1H, Ar–H), 7.27 (d, J = 8.5 Hz, 1H, Ar–H), 6.99 (d, J = 7.9 Hz, 1H, Ar–H), 6.41 (s, 1H, Ar–H), 2.88 (t, J = 7.6 Hz, 2H, CH_2), 2.49 (s, 3H, CH_3), 1.67-1.53 (m, 2H, CH_2), 1.44-1.20 (m, 10H, CH_2), 0.92 (t, J = 6.7 Hz, 3H, CH_3). $^{13}C\{^{1}H\}$-NMR (100 MHz, $CDCl_3$): δ = 151.9 (C_q), 149.7 (CH), 142.0 (C_q) , 138.3 (CH), 135.7 (C_q), 129.9 (C_q), 129.1 (C_q), 123.1 (CH), 121.9 (CH), 121.1 (CH), 119.8 (CH), 109.9 (CH), 101.9 (CH), 32.0 (CH_2), 29.5 (2C, CH_2), 29.3 (CH_2), 28.8 (CH_2), 27.7 (CH_2), 22.8 (CH_2), 21.5 (CH_3), 14.3 (CH_3). HRMS (ESI): m/z Calcd for $C_{22}H_{28}N_2$+H^+ $[M+H]^+$ 321.2325; Found 321.2323. The ^{1}H and ^{13}C spectra are consistent with those reported in the literature.[S14]

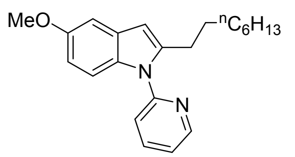

2-Octyl-1-(5-methoxypyridin-2-yl)-1*H*-indole (3ca): The representative procedure was followed, using substrate **1c** (0.045 g, 0.20 mmol) and 1-chlorooctane (**2a**; 0.059 g, 0.40 mmol), and the reaction mixture was stirred for 5 h. Purification by column chromatography on neutral alumina (petroleum ether/EtOAc: 50/1) yielded **3ca** (0.056 g, 83%) as a light yellow liquid. ^{1}H NMR (400 MHz, $CDCl_3$): δ = 8.68 (d, J = 3.7 Hz, 1H, Ar–H), 8.03-7.79 (m, 1H, Ar–H), 7.43 (d, J = 7.9 Hz, 1H, Ar–H), 7.38-7.21 (m, 2H, Ar–H), 7.09 (s, 1H, Ar–H), 6.90-6.73 (m, 1H, Ar–H), 6.41 (s, 1H, Ar–H), 3.88 (s, 3H, OCH_3), 2.86 (t, J = 7.6 Hz, 2H, CH_2), 1.70-1.50 (m, 2H, CH_2), 1.41-1.19 (m, 10H, CH_2), 0.90 (t, J = 6.7 Hz, 3H, CH_3). $^{13}C\{^{1}H\}$-NMR (100 MHz, $CDCl_3$): δ = 154.9 (C_q), 151.9 (C_q), 149.7 (CH), 142.5 (C_q), 138.4 (CH), 132.5 (C_q), 129.3 (C_q), 121.9 (CH), 121.0 (CH), 111.1 (CH), 111.0 (CH), 102.3 (CH), 102.1 (CH), 56.0 (OCH_3), 32.0 (CH_2), 29.5

(2C, CH_2), 29.3 (CH_2), 28.8 (CH_2), 27.7 (CH_2), 22.8 (CH_2), 14.2 (CH_3). HRMS (ESI): *m/z* Calcd for $C_{22}H_{28}N_2O+H^+$ $[M+H]^+$ 337.2274; Found 337.2270. The ^1H and ^{13}C spectra are consistent with those reported in the literature.[S14]

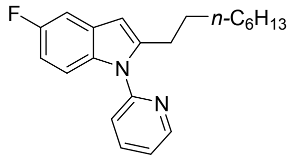

2-Octyl-1-(5-fluoropyridin-2-yl)-1*H*-indole (3da): The representative procedure was followed, using substrate **1d** (0.042 g, 0.20 mmol) and 1-chlorooctane (**2a**; 0.059 g, 0.40 mmol), and the reaction mixture was stirred for 16 h. Purification by column chromatography on neutral alumina (petroleum ether/EtOAc: 50/1) yielded **3da** (0.045 g, 69%) as a light yellow liquid. ^1H-NMR (400 MHz, $CDCl_3$): δ = 8.84-8.60 (m, 1H, Ar–H), 8.07-7.81 (m, 1H, Ar–H), 7.54-7.34 (m, 2H, Ar–H), 7.33-7.14 (m, 2H, Ar–H), 7.00-6.78 (m, 1H, Ar–H), 6.43 (s, 1H, Ar–H), 3.05- 2.75 (m, 2H, CH_2), 1.78-1.49 (m, 2H, CH_2), 1.26 (br s, 10H, CH_2), 1.06-0.81 (m, 3H, CH_3). $^{13}C\{^1H\}$-NMR (100 MHz, $CDCl_3$): δ = 158.6 (d, $^1J_{C-F}$ = 235.0 Hz, C_q), 151.6 (C_q), 149.9 (CH), 143.6 (C_q), 138.5 (CH), 134.0 (C_q), 129.2 (d, $^3J_{C-F}$ = 10.0 Hz, C_q), 122.4 (CH), 121.2 (CH), 110.9 (d, $^3J_{C-F}$ = 10.0 Hz, CH), 109.5 (d, $^2J_{C-F}$ = 25.4 Hz, CH), 105.1 (d, $^2J_{C-F}$ = 24.0 Hz, CH), 102.1 (d, $^4J_{C-F}$ = 4.0 Hz, CH), 32.0 (CH_2), 29.5 (2C, CH_2), 29.3 (CH_2), 28.7 (CH_2), 27.7 (CH_2), 22.8 (CH_2), 14.3 (CH_3). ^{19}F-NMR (377 MHz, $CDCl_3$): δ = –124.2 (s). HRMS (ESI): *m/z* Calcd for $C_{21}H_{25}FN_2+H^+$ $[M+H]^+$ 325.2075; Found 325.2073. The ^1H and ^{13}C spectra are consistent with those reported in the literature.[S14]

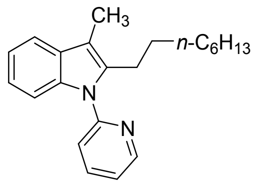

3-Methyl-2-octyl-1-(pyridin-2-yl)-1*H*-indole (3ea): The representative procedure was followed, using substrate **1e** (0.042 g, 0.20 mmol) and 1-chlorooctane (**2a**; 0.059 g, 0.40 mmol), and the reaction mixture was stirred for 5 h. Purification by column chromatography on neutral alumina (petroleum ether/EtOAc: 50/1) yielded **3ea** (83%; ^{1}H NMR yield using CH_2Br_2 as internal standard) as a light yellow liquid. ^{1}H NMR (400 MHz, $CDCl_3$): δ = 8.67 (d, J = 3.7 Hz, 1H, Ar–H), 7.88 (t, J = 7.6 Hz, 1H, Ar–H), 7.58 (d, J = 7.9 Hz, 1H, Ar–H), 7.44 (d, J = 7.9 Hz, 1H, Ar–H), 7.35 (d, J = 7.3 Hz, 1H, Ar–H) 7.33-7.27 (m, 1H, Ar–H), 7.23-7.12 (m, 2H, Ar–H), 2.94 (t, J = 7.6 Hz, 2H, CH_2), 2.36 (s, 3H, CH_3), 1.44-1.34 (m, 2H, CH_2), 1.33-1.15 (m, 10H, CH_2), 0.90 (t, J = 7.0 Hz, 3H, CH_3). $^{13}C\{^{1}H\}$-NMR (125 MHz, $CDCl_3$): δ = 152.1 (C_q), 149.7 (CH), 138.3 (CH), 137.4 (C_q), 136.7 (C_q), 129.6 (C_q), 121.8 (CH), 121.8 (CH), 121.2 (CH), 120.2 (CH), 118.3 (CH), 110.0 (CH), 109.8 (C_q), 32.0 (CH_2), 29.5 (CH_2), 29.3 (2C, CH_2), 29.2 (CH_2), 24.8 (CH_2), 22.8 (CH_2), 14.2 (CH_3), 9.0 (CH_3). HRMS (ESI): *m/z* Calcd for $C_{22}H_{28}N_2$+H^+ $[M+H]^+$ 321.2325; Found 321.2323. The ^{1}H and ^{13}C spectra are consistent with those reported in the literature.[S14]

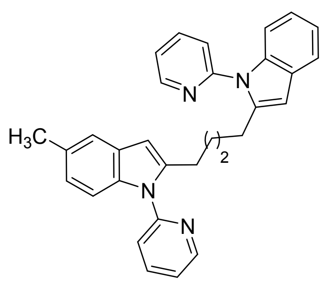

5-Methyl-1-(pyridin-2-yl)-2-(4-(1-(pyridin-2-yl)-1*H*-indol-2-yl)butyl)-1*H*-indole (3by): The representative procedure was followed, using 5-methyl-1-(pyridin-2-yl)-1*H*-indole (**1b**; 0.042 g,

0.20 mmol), 2-(4-chlorobutyl)-1-(pyridin-2-yl)-1*H*-indole (**2y**, 0.114 g, 0.40 mmol), and the reaction mixture was stirred for 16 h. Purification by column chromatography on neutral alumina (petroleum ether/EtOAc: 10/1) yielded **3by** (0.060 g, 66%) as a light yellow solid. ^{1}H-NMR (500 MHz, $CDCl_3$): δ = 8.64-8.49 (m, 2H, Ar–H), 7.80-7.71 (m, 2H, Ar–H), 7.59-7.50 (m, 1H, Ar–H), 7.37-7.27 (m, 4H, Ar–H), 7.25-7.16 (m, 3H, Ar–H), 7.16-7.08 (m, 2H, Ar–H), 6.94 (d, *J* = 8.4 Hz, 1H, Ar–H), 6.34 (s, 1H, Ar–H), 6.27 (s, 1H, Ar–H), 2.80 (br s, 4H, CH_2), 2.44 (s, 3H, CH_3), 1.48-1.56 (m, 4H, CH_2). $^{13}C\{^1H\}$-NMR (125 MHz, $CDCl_3$): δ = 151.8 (C_q), 151.6 (C_q), 149.7 (CH), 149.6 (CH), 141.3 (2C, C_q), 138.3 (2C, CH), 137.4 (C_q), 135.7 (C_q), 129.9 (C_q), 129.0 (C_q), 128.7 (C_q), 123.1 (CH), 122.1 (CH), 121.9 (CH), 121.7 (CH), 121.3 (CH), 121.0 (CH), 120.7 (CH), 120.0 (CH), 119.8 (CH), 110.2 (CH), 109.9 (CH), 102.4 (CH), 102.1 (CH), 28.1 (2C, CH_2), 27.4 (CH_2), 27.3 (CH_2), 21.6 (CH_3). HRMS (ESI): *m/z* Calcd for $C_{31}H_{28}N_4+H^+$ $[M+H]^+$ 457.2387; Found 457.2388.

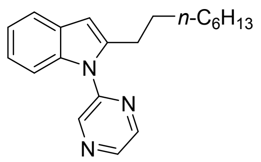

2-Octyl-1-(pyrazin-2-yl)-1*H*-indole (3fa): The representative procedure was followed, using substrate 1-(pyrazin-2-yl)-1*H*-indole (**1f**; 0.039 g, 0.20 mmol), 1-chlorooctane (**2a**; 0.059 g, 0.40 mmol) and the reaction mixture was stirred for 5 h. Purification by column chromatography on neutral alumina (petroleum ether/EtOAc: 50/1) yielded **3fa** (0.018 g, 29%) as a brown liquid. ^{1}H-NMR (400 MHz, $CDCl_3$): δ= 8.79 (s, 1H, Ar–H), 8.65-8.60 (m, 1H, Ar–H), 8.56 (d, *J* = 3.0 Hz, 1H, Ar–H), 7.61-7.54 (m, 1H, Ar–H), 7.41-7.34 (m, 1H, Ar–H), 7.19-7.12 (m, 2H, Ar–H), 6.49 (s, 1H, Ar–H), 2.82 (t, *J* = 7.6 Hz, 2H, CH_2), 1.57 (pent, *J* = 7.6 Hz, 2H, CH_2), 1.29-1.22 (m, 10H, CH_2), 0.86 (t, *J* = 6.9 Hz, 3H, CH_3). $^{13}C\{^1H\}$-NMR (100 MHz, $CDCl_3$): δ= 148.4 (C_q),

143.7 (CH), 142.6 (CH), 142.2 (CH), 141.7 (C_q), 137.1 (C_q), 129.0 (C_q), 122.2 (CH), 121.4 (CH), 120.2 (CH), 109.9 (CH), 103.5 (CH), 31.8 (CH_2), 29.7 (CH_2), 29.3 (CH_2), 29.2 (CH_2), 28.7 (CH_2), 27.5 (CH_2), 22.7 (CH_2), 14.1 (CH_3). HRMS (ESI): *m/z* Calcd for $C_{20}H_{25}N_3+H^+$ $[M+H]^+$ 308.2121; Found 308.2119.

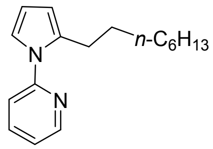

2-(2-Octyl-1*H*-pyrrol-1-yl)pyridine (3ga): The representative procedure was followed, using substrate **1g** (0.029 g, 0.20 mmol) and 1-chlorooctane (**2a**; 0.059 g, 0.40 mmol), and the reaction mixture was stirred for 16 h. Purification by column chromatography on neutral alumina (petroleum ether/EtOAc: 50/1) yielded **3ga** (64%; ^{1}H NMR yield using CH_2Br_2 as internal standard) as a light yellow liquid. ^{1}H-NMR (500 MHz, $CDCl_3$) δ = 8.52 (d, J = 3.4 Hz, 1H, Ar–H), 7.78 (t, J = 6.9 Hz, 1H, Ar–H), 7.29 (d, J = 8.0 Hz, 1H, Ar–H), 7.20 (dd, J = 6.9, 5.3 Hz, 1H, Ar–H), 7.01 (br s, 1H, Ar–H), 6.24 (t, J = 2.9 Hz, 1H, Ar–H), 6.09 (br s, 1H, Ar–H), 2.81 (t, J = 7.6 Hz, 2H, CH_2), 1.62-1.49 (m, 2H, CH_2), 1.30-1.22 (m, 10H, CH_2), 0.87 (t, J = 6.9 Hz, 3H, CH_3). $^{13}C\{^1H\}$-NMR (125 MHz, $CDCl_3$): δ = 153.2 (C_q), 149.0 (CH), 138.3 (CH), 134.6 (C_q), 121.2 (CH), 120.6 (CH), 117.8 (CH), 109.1 (CH), 108.8 (CH), 32.0 (CH_2), 29.6 (CH_2), 29.5 (CH_2), 29.4 (2C, CH_2), 27.7 (CH_2), 22.8 (CH_2), 14.3 (CH_3). HRMS (ESI): *m/z* Calcd for $C_{17}H_{24}N_2+H^+$ $[M+H]^+$ 257.2012; Found 257.2012.

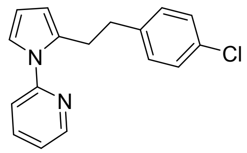

2-(2-(4-Chlorophenethyl)-1*H*-pyrrol-1-yl)pyridine (3gm): The representative procedure was followed, using substrate **1g** (0.029 g, 0.20 mmol), 1-chloro-4-(2-chloroethyl)benzene (**2m**; 0.070 g, 0.40 mmol) and the reaction mixture was stirred for 24 h. Purification by column chromatography on neutral alumina (petroleum ether/EtOAc: 50/1) yielded **3gm** (0.019 g, 33%) as a brown liquid. ^{1}H-NMR (500 MHz, CDCl$_3$): δ = 8.54 (d, J = 4.2 Hz, 1H, Ar–H), 7.79 (t, J = 7.4 Hz, 1H, Ar–H), 7.30-7.25 (m, 1H, Ar–H), 7.23-7.19 (m, 3H, Ar–H), 7.08-7.03 (m, 2H, Ar–H), 7.01 (s, 1H, Ar–H), 6.26 (s, 1H, Ar–H), 6.13 (s, 1H, Ar–H), 3.17 (t, J = 7.9 Hz, 2H, CH$_2$), 2.86 (t, J = 7.4 Hz, 2H, CH$_2$). ^{13}C{^{1}H}-NMR (125 MHz, CDCl$_3$): δ = 152.9 (C$_q$), 148.7 (CH), 140.3 (C$_q$), 138.3 (CH), 133.2 (C$_q$), 131.5 (C$_q$), 129.7 (2C, CH), 128.3 (2C, CH), 121.1 (CH), 120.5 (CH), 117.3 (CH), 109.4 (CH), 109.2 (CH), 35.4 (CH$_2$), 29.6 (CH$_2$). HRMS (ESI): *m/z* Calcd for $C_{17}H_{15}ClN_2+H^+$ [M+H]$^+$ 283.0997; Found 283.0996.

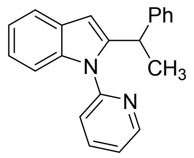

2-(1-Phenylethyl)-1-(pyridin-2-yl)-1*H*-indole (5aa): The representative procedure was followed, using substrate **1a** (0.039 g, 0.20 mmol), (1-bromoethyl)benzene (**4a**; 0.074 g, 0.40 mmol) and the reaction mixture was stirred for 16 h. Purification by column chromatography on neutral alumina (petroleum ether/EtOAc: 50/1) yielded **5aa** (0.025 g, 42%) as a light yellow liquid. ^{1}H-NMR (400 MHz, CDCl$_3$): δ = 8.58 (br s, 1H, Ar–H), 8.17 (d, J = 7.9 Hz, 1H, Ar–H), 7.89-7.77 (m, 1H, Ar–H), 7.63 (s, 1H, Ar–H), 7.52 (d, J = 8.5 Hz, 1H), 7.44-7.23 (m, 6H, Ar–H),

7.22-7.03 (m, 3H, Ar–H), 4.41 (q, J = 7.3 Hz, 1H, CH), 1.77 (d, J = 7.3 Hz, 3H, CH_3). $^{13}C\{^1H\}$-NMR (100 MHz, $CDCl_3$): δ = 152.7 (C_q), 149.2 (CH) 146.4 (C_q), 138.5 (CH) 135.9 (C_q), 129.9 (C_q), 128.6 (2C, CH), 127.7 (2C, CH), 126.3 (CH), 124.2 (C_q), 123.3 (CH), 123.1 (CH), 121.0 (CH), 120.2 (CH), 119.9 (CH), 114.6 (CH), 113.0 (CH), 37.1 (CH), 22.5 (CH_3). HRMS (ESI): *m/z* Calcd for $C_{21}H_{18}N_2$+H^+ $[M+H]^+$ 299.1543; Found 299.1541. The ^1H and ^{13}C spectra are consistent with those reported in the literature.[S14]

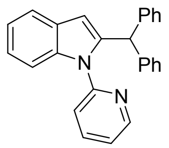

2-Benzhydryl-1-(pyridin-2-yl)-1*H*-indole (5ab): The representative procedure was followed using, substrate **1a** (0.039 g, 0.20 mmol), (bromomethylene)dibenzene (**4b**; 0.099 g, 0.40 mmol) or (chloromethylene)dibenzene (0.081 g, 0.40 mmol) and the reaction mixture was stirred for 16 h. Purification by column chromatography on neutral alumina (petroleum ether/EtOAc: 50/1) yielded **5ab** (0.052 g, 72%; 57% using chloro-electrophile) as a pale yellow solid. ^1H-NMR (500 MHz, $CDCl_3$): δ = 8.65 (d, J = 3.8 Hz, 1H, Ar–H), 8.37 (d, J = 8.4 Hz, 1H, Ar–H), 7.91-7.79 (m, 1H, Ar–H), 7.53-7.34 (m, 14H, Ar–H), 7.23 (t, J = 6.9 Hz, 2H, Ar–H), 5.86 (s, 1H, CH). $^{13}C\{^1H\}$-NMR (125 MHz, $CDCl_3$): δ = 152.6 (C_q), 149.0 (CH), 143.5 (2C, C_q), 138.4 (CH), 136.0 (C_q), 129.80 (C_q), 129.2 (4C, CH), 128.6 (4C, CH), 126.6 (2C, CH), 125.7 (CH), 123.5 (CH), 122.8 (C_q), 121.2 (CH), 120.4 (CH), 119.9 (CH), 114.6 (CH), 113.3 (CH), 48.9 (CH). HRMS (ESI): *m/z* Calcd for $C_{26}H_{20}N_2$+H^+ $[M+H]^+$ 361.1699; Found 361.1698. The ^1H and ^{13}C spectra are consistent with those reported in the literature.[S14]

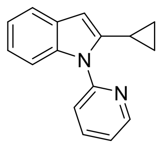

2-Cyclopropyl-1-(pyridin-2-yl)-1*H*-indole (5ac): The representative procedure was followed, using substrate **1a** (0.039 g, 0.20 mmol) and bromocyclopropane (**4c**, 0.048 g, 0.40 mmol) and the reaction mixture was stirred for 16 h. Column chromatography on neutral alumina (petroleum ether/EtOAc: 50/1) afforded the **5ac** (0.035 g, 75%). ^{1}H-NMR (500 MHz, $CDCl_3$): δ = 8.69 (d, J = 5.0 Hz, 1H, Ar–H), 7.93-7.82 (m, 1H, Ar–H), 7.60-7.51 (m, 2H, Ar–H), 7.46 (dd, J = 6.3, 2.5 Hz, 1H, Ar–H), 7.32 (dd, J = 7.1, 5.1 Hz, 1H, Ar–H), 7.19-7.08 (m, 2H, Ar–H), 6.29 (s, 1H, Ar–H), 2.03 (br s, 1H, CH), 0.95-0.86 (m, 2H, CH_2), 0.71-0.83 (m, 2H, CH_2). $^{13}C\{^{1}H\}$-NMR (125 MHz, $CDCl_3$): δ = 151.9 (C_q), 149.6 (CH), 143.4 (C_q), 138.2 (CH), 137.5 (C_q), 128.6 (C_q), 121.9 (2C, CH), 121.2 (CH), 120.9 (CH), 120.0 (CH), 110.7 (CH), 100.0 (CH), 8.8 (CH), 8.4 (2C, CH_2). HRMS (ESI): *m/z* Calcd for $C_{16}H_{14}N_2+H^+$ $[M+H]^+$ 235.1230; Found 235.1232. The ^{1}H and ^{13}C spectra are consistent with those reported in the literature.[S14]

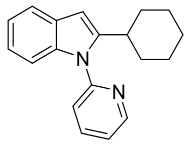

2-Cyclohexyl-1-(pyridin-2-yl)-1*H*-indole (5ad): The representative procedure was followed, using substrate **1a** (0.039 g, 0.20 mmol) and bromocyclohexane (**4d**; 0.065 g, 0.40 mmol) or chlorocyclohexane (0.047 g, 0.40 mmol), and the reaction mixture was stirred for 16 h. Purification by column chromatography on neutral alumina (petroleum ether/EtOAc: 50/1) yielded **5ad** (0.036 g, 65%; 41% using chloro-electrophile) as a light yellow liquid. ^{1}H-NMR (400 MHz, $CDCl_3$): δ = 8.76-8.62 (m, 1H, Ar–H), 7.90 (td, J = 7.7, 1.7 Hz, 1H, Ar–H), 7.60 (dd,

J = 5.9, 2.9 Hz, 1H, Ar–H), 7.45 (d, J = 7.6 Hz, 1H, Ar–H), 7.34 (dd, J = 7.1, 5.1 Hz, 1H, Ar–H), 7.32-7.23 (m, 1H, Ar–H), 7.18-7.07 (m, 2H, Ar–H), 6.46 (s, 1H, Ar–H), 3.07-2.96 (m, 1H, CH), 1.92 (d, J = 12.6 Hz, 2H, CH_2), 1.75 (dd, J = 8.6, 3.6 Hz, 2H, CH_2), 1.48-1.36 (m, 2H, CH_2), 1.31-1.17 (m, 4H, CH_2). $^{13}C\{^1H\}$-NMR (125 MHz, $CDCl_3$): δ = 151.9 (C_q), 149.8 (CH), 147.4 (C_q), 138.5 (CH), 137.5 (C_q), 128.7 (C_q), 122.3 (CH), 121.7 (CH), 121.6 (CH), 120.6 (CH), 120.1 (CH), 110.1 (CH), 100.0 (CH), 35.8 (CH), 33.4 (2C, CH_2), 26.6 (2C, CH_2), 26.3 (CH_2). HRMS (ESI): *m/z* Calcd for $C_{19}H_{20}N_2$+H^+ $[M+H]^+$ 277.1699; Found 277.1700. The ^1H and ^{13}C spectra are consistent with those reported in the literature.[S14]

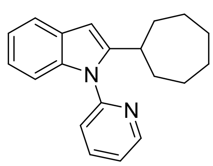

2-Cycloheptyl-1-(pyridin-2-yl)-1*H*-indole (5ae): The representative procedure was followed, using substrate **1a** (0.039 g, 0.20 mmol), bromocycloheptane (**4e**; 0.071 g, 0.40 mmol) and the reaction mixture was stirred for 16 h. Purification by column chromatography on neutral alumina (petroleum ether/EtOAc: 50/1) yielded **5ae** (0.030 g, 52%) as a light yellow liquid. ^1H-NMR (400 MHz, $CDCl_3$): δ = 8.69 (d, J = 3.7 Hz, 1H, Ar–H), 7.91 (t, J = 7.0 Hz, 1H, Ar–H), 7.63-7.50 (m, 1H, Ar–H), 7.44 (d, J = 7.9 Hz, 1H, Ar–H), 7.40-7.32 (m, 1H, Ar–H), 7.31-7.20 (m, 1H, Ar–H), 7.19-7.04 (m, 2H, Ar–H), 6.47 (s, 1H, Ar–H), 3.33-3.09 (m, 1H, CH), 1.97 (dt, J = 6.9, 3.6 Hz, 2H, CH_2), 1.80-1.65 (m, 4H, CH_2), 1.57 (br s, 4H, CH_2), 1.42-1.31 (m, 2H, CH_2). $^{13}C\{^1H\}$-NMR (100 MHz, $CDCl_3$): δ = 151.9 (C_q), 149.9 (CH), 148.5 (C_q), 138.5 (CH) 137.5 (C_q), 128.7 (C_q), 122.3 (CH), 121.9 (CH), 121.6 (CH), 120.6 (CH), 120.1 (CH), 110.1 (CH), 100.0 (CH), 37.0 (CH), 35.0 (2C, CH_2), 28.4 (2C, CH_2), 26.8 (2C, CH_2). HRMS (ESI): *m/z*

Calcd for $C_{20}H_{22}N_2$+H^+ $[M+H]^+$ 291.1856; Found 291.1855. The ^{1}H and ^{13}C spectra are consistent with those reported in the literature.[S14]

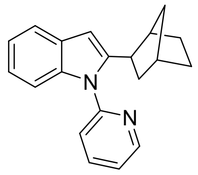

2-((1*R*,2*R*,4*S*)-Bicyclo[2.2.1]heptan-2-yl)-1-(pyridin-2-yl)-1*H*-indole (5af): The representative procedure was followed, using substrate **1a** (0.039 g, 0.20 mmol), 2-bromobicyclo[2.2.1]heptane (**4f**; 0.070 g, 0.40 mmol) and the reaction mixture was stirred for 16 h. Purification by column chromatography on neutral alumina (petroleum ether/EtOAc: 50/1) yielded **5af** (35%; ^{1}H NMR yield using CH_2Br_2 as internal standard) as a pale yellow solid. ^{1}H-NMR (500 MHz, $CDCl_3$): δ = 8.73-8.64 (m, 1H, Ar–H), 7.90 (td, J = 7.7, 1.7 Hz, 1H, Ar–H), 7.61-7.55 (m, 1H, Ar–H), 7.44 (d, J = 8.0 Hz, 1H, Ar–H), 7.34 (dd, J = 7.2, 5.3 Hz, 1H, Ar–H), 7.27-7.22 (m, 1H, Ar–H), 7.15-7.07 (m, 2H, Ar–H), 6.43 (s, 1H, Ar–H), 3.12 (dd, J = 8.4, 5.7 Hz, 1H, CH), 2.33 (br s, 1H, CH), 2.28 (br s, 1H, CH), 1.67-1.56 (m, 2H, CH_2), 1.55-1.38 (m, 3H, CH_2), 1.21-1.10 (m, 3H, CH_2). $^{13}C\{^1H\}$-NMR (125 MHz, $CDCl_3$): δ = 152.0 (C_q), 149.8 (CH), 146.9 (C_q), 138.4 (CH), 137.8 (C_q), 128.6 (C_q), 122.3 (CH), 121.9 (CH), 121.7 (CH), 120.6 (CH), 120.1 (CH), 110.1 (CH), 100.4 (CH), 42.3 (CH) 39.8 (CH) 38.3 (CH_2) 36.8 (CH) 36.4 (CH_2), 29.9 (CH_2), 28.9 (CH_2). HRMS (ESI): *m/z* Calcd for $C_{20}H_{20}N_2$+H^+ $[M+H]^+$ 289.1699; Found 289.1700.

5. Other indoles and alkyl chlorides employed

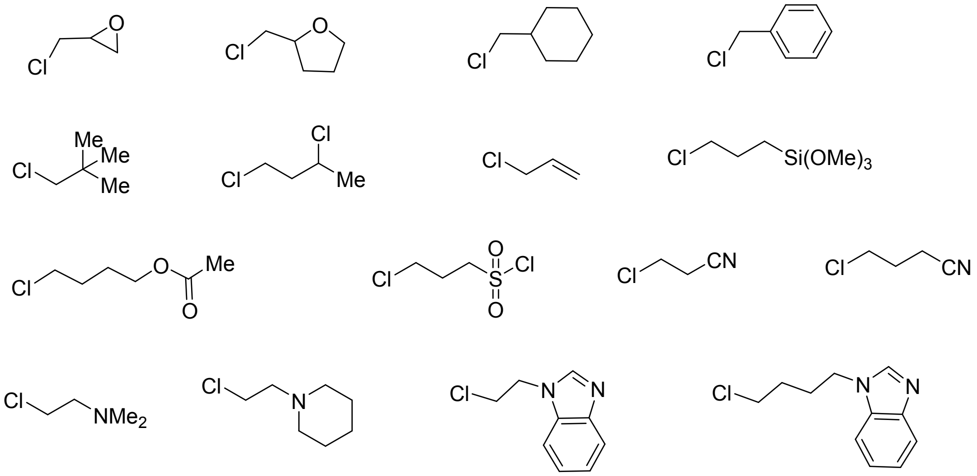

Figure S2. List of alkyl chlorides that are non-reactive under the reaction conditions.

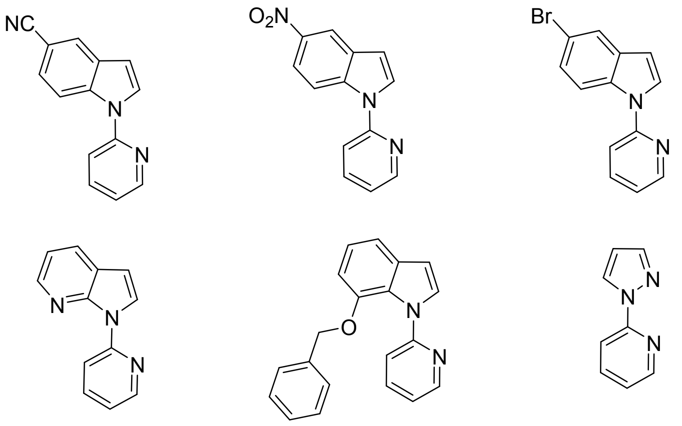

Figure S3. List of indoles that are non-reactive under the reaction conditions.

6. Procedure for one pot double alkylation

Synthesis of 1,4-bis(1-(pyridin-2-yl)-1*H*-indol-2-yl)butane (3ay): To a screw-cap tube (5 mL) equipped with a magnetic stir bar were introduced 1-bromo-4-chlorobutane (**2h**, 0.035 g, 0.20 mmol), 1-pyridin-2-yl-1*H*-indole (**1a**, 0.156 g, 0.80 mmol), $(thf)_2NiBr_2$ (0.0073 g, 0.02 mmol), bpy (0.003 g, 0.02 mmol), LiHMDS (0.134 g, 0.80 mmol) inside the glove box. To the above mixture in the tube was added toluene (2.0 mL), and the resultant reaction mixture was stirred at 60 °C in a preheated oil bath for 16 h. At ambient temperature, the reaction mixture was quenched with distilled H_2O (10 mL) and then neutralized with 2N HCl (2.0 mL). The crude product was then extracted with EtOAc (20 mL x 3). The combined organic extract was dried over Na_2SO_4, and the volatiles were evaporated *in vacuo*. The remaining residue was purified by column chromatography on basic alumina (petroleum ether/EtOAc 10/1) to yield **3ay** (0.058 g, 66%) as a light yellow solid.

Synthesis of 5-methyl-1-(pyridin-2-yl)-2-(4-(1-(pyridin-2-yl)-1*H*-indol-2-yl)butyl)-1*H*-indole (3by): To a screw-cap tube (5 mL) equipped with a magnetic stir bar were introduced 1-pyridin-2-yl-1*H*-indole (**1a**, 0.039 g, 0.20 mmol), 1-bromo-4-chlorobutane (**2h**, 0.045 g, 0.26 mmol), $(thf)NiBr_2$ (0.0073 g, 0.02 mmol), bpy (0.003 g, 0.02 mmol), LiHMDS (0.067 g, 0.40 mmol) inside the glove box. To the above mixture in the tube was added toluene (2.0 mL), and the resultant reaction mixture was stirred at 60 °C in a preheated oil bath for 16 h. At ambient temperature, the reaction tube was transferred to the glove box and 5-methyl-1-(pyridin-2-yl)-1*H*-indole (**1b**, 0.083 g, 0.40 mmol) and LiHMDS (0.067 g, 0.40 mmol) were added into it. The resultant reaction mixture was again stirred at 60 °C for 16 h. At ambient temperature, the reaction mixture was quenched with distilled H_2O (10 mL) and then neutralized with 2N HCl (2.0 mL). The crude product was then extracted with EtOAc (20 mL x 3). The combined organic

extract was dried over Na_2SO_4, and the volatiles were evaporated *in vacuo*. The remaining residue was purified by column chromatography on basic alumina (petroleum ether/EtOAc 10/1) to yield **3by** (0.059 g, 65%) as a light yellow solid.

7. Procedure for removal of directing group

Representative procedure: synthesis of 2-octyl-1*H*-indole (6aa): To an oven dried Schlenk tube, **3aa** (0.035 g, 0.11 mmol) was introduced and CH_2Cl_2 (5.0 mL) was added into it. Methyl trifluoromethanesulfonate, MeOTf (0.036 g, 0.22 mmol) was added drop wise *via* a syringe to the reaction mixture at 0 °C and the resultant reaction mixture was stirred at room temperature for 16 h. The volatiles were removed under vacuum and the residue was redissolved in MeOH (2.0 mL). To the resultant mixture, NaOH (2M aq, 2.0 mL) solution was added and the reaction mixture was stirred at 60 °C for 12 h. At ambient temperature, the volatiles were evaporated under reduced pressure, and the resulting residue was extracted with EtOAc (15 mL x 3). The combined organic extract was washed with brine, dried over Na_2SO_4 and the volatiles were evaporated in *vacuo*. The remaining residue was purified by column chromatography on neutral alumina (petroleum ether/EtOAc: 50/1) to yield **6aa** (0.022 g, 87%) as a light yellow liquid.

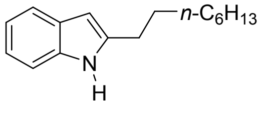

2-Octyl-1*H*-indole (6aa): ^{1}H-NMR (400 MHz, $CDCl_3$): δ = 7.85 (br s, 1H, NH), 7.53 (d, J = 7.3 Hz, 1H, Ar–H), 7.30 (d, J = 7.9 Hz, 1H, Ar–H), 7.08 (d, J = 7.9 Hz, 1H, Ar–H), 7.11 (d, J = 8.5 Hz, 1H, Ar–H), 6.24 (s, 1H, Ar–H), 2.75 (t, J = 7.6 Hz, 2H, CH_2), 1.91-1.63 (m, 2H, CH_2), 1.28

(br s, 10H, CH_2), 0.89 (t, J = 6.4 Hz, 3H, CH_3). $^{13}C\{^1H\}$-NMR (100 MHz, $CDCl_3$): δ = 140.2 (C_q), 136.0 (C_q), 129.1 (C_q), 121.1 (CH), 119.9 (CH), 119.7 (CH), 110.4 (CH), 99.6 (CH), 32.0 (CH_2), 29.9 (CH_2), 29.6 (CH_2), 29.5 (CH_2), 29.4 (CH_2), 28.5 (CH_2), 22.8 (CH_2), 14.3 (CH_3). HRMS (ESI): *m/z* Calcd for $C_{16}H_{23}N+H^+$ $[M+H]^+$ 230.1903 Found 230.1905. The ^1H and ^{13}C spectra are consistent with those reported in the literature.[S14]

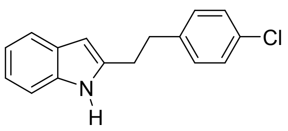

2-(4-Chlorophenethyl)-1*H*-indole (6am): The representative procedure was followed, using substrate **3am** (0.035 g, 0.105 mmol) and methyl trifluoromethanesulfonate, MeOTf (0.069 g, 0.42 mmol). Purification by column chromatography on neutral alumina (petroleum ether/EtOAc: 50/1) yielded **6am** (0.0193 g, 72%) as a light yellow solid. ^1H-NMR (500 MHz, $CDCl_3$): δ = 7.95 (br s, 1H, NH), 7.72 (d, J = 7.9 Hz, 1H, Ar–H), 7.54-7.38 (m, 3H, Ar–H), 7.38-7.22 (m, 4H, Ar–H), 6.44 (s, 1H, Ar–H), 3.20-2.93 (m, 4H, CH_2). $^{13}C\{^1H\}$-NMR (125 MHz, $CDCl_3$): δ = 139.7 (C_q), 138.6 (C_q), 136.0 (C_q), 132.2 (C_q), 130.0 (2C, CH), 128.8 (2C, CH), 128.7 (C_q), 121.4 (CH), 120.1 (CH), 119.9 (CH), 110.5 (CH), 100.2 (CH), 35.2 (CH_2), 30.2 (CH_2). HRMS (ESI): *m/z* Calcd for $C_{16}H_{14}NCl+H^+$ $[M+H]^+$ 256.0888; Found 256.0891.

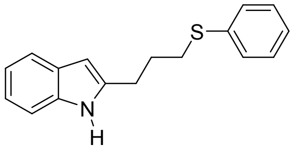

2-(3-(Phenylthio)propyl)-1*H*-indole (6ao): The representative procedure was followed, using substrate **3ao** (0.04 g, 0.116 mmol) and methyl trifluoromethanesulfonate, MeOTf (0.076 g, 0.464 mmol). Purification by column chromatography on neutral alumina (petroleum

ether/EtOAc: 50/1) yielded **6ao** (0.0196 g, 63%) as a brown solid. ^{1}H-NMR (500 MHz, $CDCl_3$): δ = 7.88 (br s, 1H, NH), 7.55 (d, J = 7.9 Hz, 1H, Ar–H), 7.40-7.29 (m, 5H, Ar–H), 7.26-7.20 (m, 1H, Ar–H), 7.19-7.08 (m, 2H, Ar–H), 6.28 (s, 1H, Ar– H), 3.02 (t, J = 7.3 Hz, 2H, CH_2), 2.95 (t, J = 7.3 Hz, 2H, CH_2), 2.09 (app. pent, J = 7.3 Hz, 2H, CH_2). $^{13}C\{^1H\}$-NMR (125 MHz, $CDCl_3$): δ = 138.3 (C_q), 136.1 (C_q), 135.9 (C_q), 129.4 (2C, CH), 128.9 (2C, CH), 128.8 (C_q), 126.1 (CH), 121.2 (CH), 119.8 (CH), 119.7 (CH), 110.3 (CH), 110.0 (CH), 32.9 (CH_2), 28.5 (CH_2), 26.8 (CH_2). HRMS (ESI): *m/z* Calcd for $C_{17}H_{17}NS+H^+$ $[M+H]^+$ 268.1154; Found 268.1155.

8. Procedure for NMR tube experiment

To a J-Young NMR tube, the ligand bpy (0.006 g, 0.04 mmol) was introduced, and 4-MeO-C_6H_5-Me (0.005 mL, 0.04 mmol, internal standard) and toluene-d^8 (0.5 mL) was added into it. The ^{1}H NMR analysis shows the expected signals for the bpy compound (Figure S4A). To the NMR tube, $(thf)_2NiBr_2$ (0.015 g, 0.04 mmol) and LiHMDS (0.027 g, 0.16 mmol) were added and the reaction mixture was heated at 60 ^oC for 15 min. The ^{1}H NMR analysis shows a broad signal at 10.69 ppm for all the bpy protons (coordinated to Ni) and a signal at 0.08 ppm for the unreacted LiHMDS (Figure S4B). In addition, de-coordinated THF protons are visible. This suggests the probable formation of a paramagnetic bpy-coordinated nickel species during the reaction. To the same NMR tube, indole **1a** (0.008 g, 0.04 mmol) and 1-chlorooctane (**2a**; 0.006 g, 0.04 mmol) were added and the reaction was further heated at 60 ^oC for 15 min in a preheated oil bath. The ^{1}H NMR analysis of the reaction mixture shows peaks for product **3aa**, however, the peak corresponding to the (bpy)Ni was disappeared (Figure S4C). This strongly suggests the existence of a paramagnetic (bpy)Ni species during the reaction.

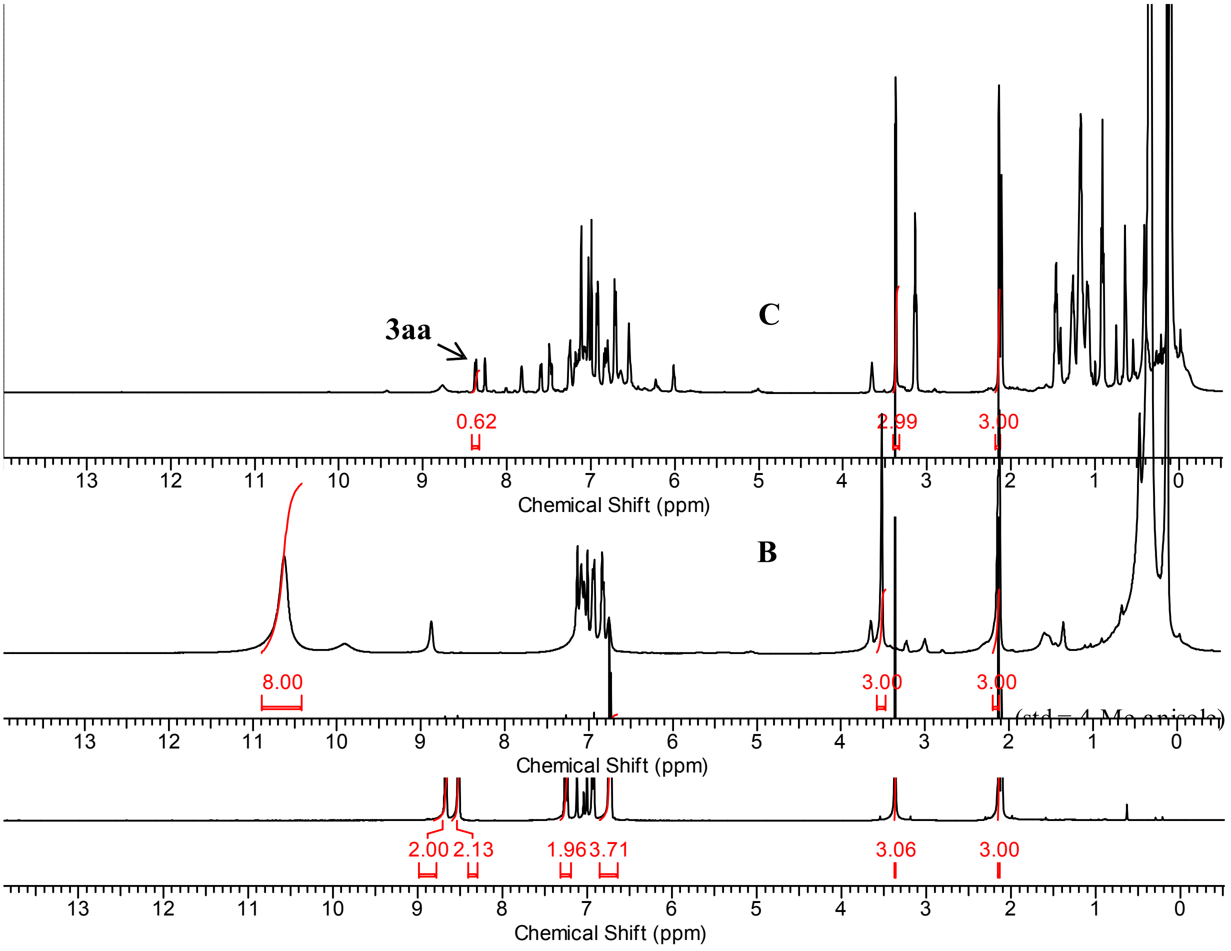

Figure S4. ^{1}H NMR spectra of controlled alkylation reaction: (A) bpy in toluene-d_8, (B) bpy + $(thf)_2NiBr_2$ + LiHMDS in toluene-d_8 after heating 15 min at 60 °C, (C) bpy + $(thf)_2NiBr_2$ + LiHMDS + indole **1a** + 1-chlorooctane (**2a**) in toluene-d_8 after heating 15 min at 60 °C.

9. Procedure for stoichiometric reaction

Procedure (without addition of 1-chlorooctane, Scheme 9). To a Teflon-screw capped tube equipped with magnetic stir bar were introduced $(thf)_2NiBr_2$ (0.037 g, 0.10 mmol), bpy (0.016 g, 0.10 mmol), LiHMDS (0.033 g, 0.20 mmol), indole **1a** (0.039 g, 0.20 mmol) and *n*-hexadecane (0.025 mL, 0.085 mmol, internal standard), and toluene (1.0 mL) was added. The reaction mixture was then stirred at 60 °C for 4 h in a pre-heated oil bath. At ambient temperature, the reaction was quenched with H_2O (5 mL) and an aliquot was subjected to GC analysis. The GC spectrum shows the presence of dimer of indole (15%) and indole **1a** (57%).

Procedure (without addition of indole, Scheme 9). To a Teflon-screw capped tube equipped with magnetic stir bar were introduced $(thf)_2NiBr_2$ (0.037 g, 0.10 mmol), bpy (0.016 g, 0.10 mmol), LiHMDS (0.033 g, 0.20 mmol), 1-chlorooctane (0.030 g, 0.20 mmol) and *n*-hexadecane (0.025 mL, 0.085 mmol, internal standard), and toluene (1.0 mL). The reaction mixture was then stirred at 60 °C for 4 in a pre-heated oil bath. At ambient temperature, the reaction was quenched with H_2O (5 mL) and an aliquot was subjected to GC analysis. The GC spectrum shows the presence of 1-chlorooctane (97%), and no other products (1-octene or *n*-octane) from this reaction were observed.

10. External additive experiments

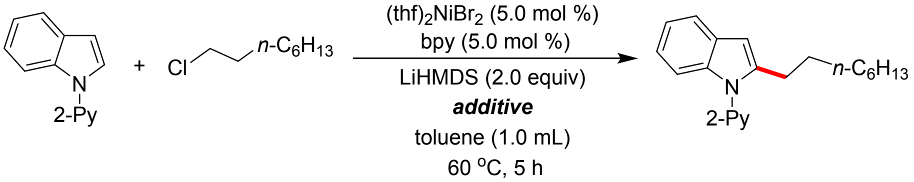

Additive	Product (3aa)
None	88%
TEMPO (2.0 equiv)	--
Galvinoxyl (2.0 equiv)	--

Procedure: The representative procedure of the alkylation reaction was followed using indole **1a** (0.039 g, 0.20 mmol), 1-chlorooctane (0.059 g, 0.40 mmol), $(thf)_2NiBr_2$ (0.0037 g, 0.01 mmol, 5.0 mol %), bpy (0.0016 g, 0.01 mmol, 5.0 mol %), LiHMDS (0.067 g, 0.40 mmol), TEMPO (0.062 g, 0.40 mmol) or galvinoxyl (0.169 g, 0.40 mmol) and the reaction mixture was stirred for 5 h. The GC analysis of the crude reaction mixture does not show the formation of product **3aa**. In addition, the coupled product of TEMPO and galvinoxyl with *n*-octyl was not detected in the GCMS.

11. Radical clock experiment

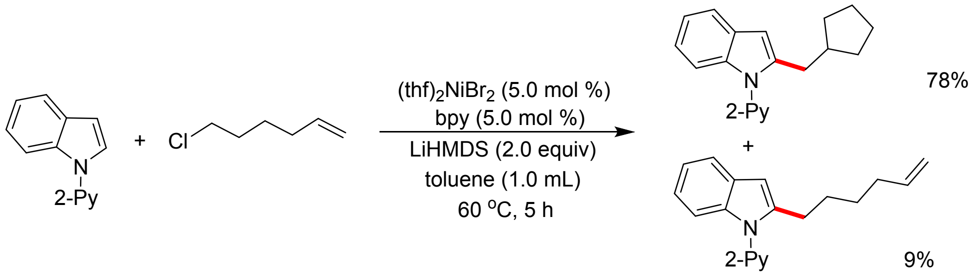

Procedure: The representative procedure of the alkylation reaction was followed, using indole **1a** (0.039 g, 0.20 mmol), 6-chloro-1-hexene (**2u**; 0.047 g, 0.40 mmol) and the reaction mixture was stirred for 16 h. Purification by column chromatography on neutral alumina

(petroleum ether/EtOAc 50/1) yielded **3au** (0.043 g, 78%) and direct coupled product 2-(hex-5-en-1-yl)-1-(pyridin-2-yl)-1*H*-indole (0.005 g, 9% (GC yield)).

12. Kinetics experiments

12.1 Reaction profile for indole alkylation

12.1.1 Representative procedure (*using in-situ generated catalyst*): To a Teflon-screw capped tube equipped with magnetic stir bar were introduced $(thf)_2NiBr_2$ (0.0037 g, 0.01 mmol, 0.01 M), bpy (0.0016 g, 0.01 mmol), LiHMDS (0.067 g, 0.40 mmol), indole **1a** (0.039 g, 0.20 mmol, 0.2 M), 1-chlorooctane (0.0.059 g, 0.40 mmol, 0.4 M) and *n*-hexadecane (0.025 mL, 0.085 mmol, 0.085 M, internal standard), and toluene (0.87 mL) was added to make the total volume to 1.0 mL. The reaction mixture was then stirred at 60 ^{o}C in a pre-heated oil bath. At regular intervals (10, 20, 30, 40, 50, 60, 90, 120, 180, 240, 300 min) the reaction vessel was cooled to ambient temperature and an aliquot of sample was withdrawn to the GC vial. The sample was diluted with acetone and subjected to GC analysis. The concentration of the product **3aa** obtained in each sample was determined with respect to the internal standard *n*-hexadecane. The data of the concentration of the product *vs* time (min) plot was drawn (Table S2 and Figure S5) with Origin Pro 8. The data's were taken from the average of two independent experiments.

Table S2 Concentration of product **3aa** at different time intervals using $(thf)_2NiBr_2$/bpy.

Time (min)	Conc. of **3aa** [M]
10	0.003
20	0.007
30	0.012
40	0.017
50	0.025
60	0.033
90	0.057
120	0.082
180	0.118
240	0.134
300	0.141

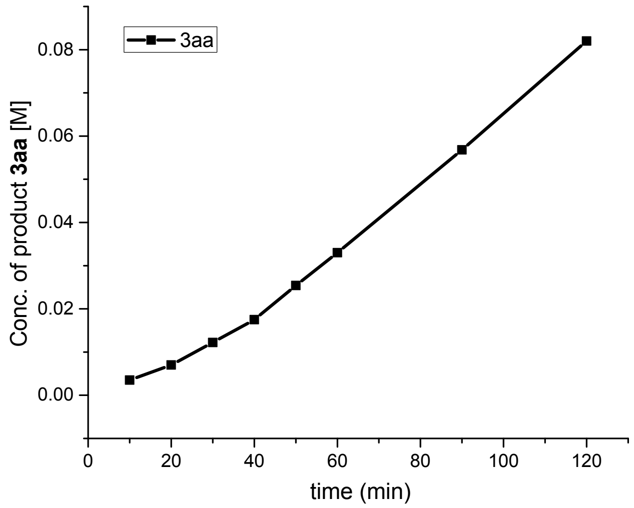

Figure S5. Time-dependent formation of **3aa** using $(thf)_2NiBr_2$/bpy system (shown up to 120 min).

12.1.2 Procedure for kinetic experiment (*using isolated complex*): Representative procedure of kinetic experiment (Sec 12.1.1) was followed using (bpy)$NiBr_2$ (0.0037 g, 0.01 mmol, 0.01 M), LiHMDS (0.067 g, 0.40 mmol), indole **1a** (0.039 g, 0.20 mmol, 0.2 M) and 1-chlorooctane (0.059 g, 0.40 mmol, 0.4 M) and *n*-hexadecane (0.025 mL, 0.085 mmol, 0.085 M, internal standard). An aliquot of sample was withdrawn to the GC vial at regular intervals (10, 20, 30, 40, 50, 60, 90, 120 min, etc.). The data of the concentration of the product *vs* time (min) plot was drawn (Table S3 and Figure S6(B)) and fitted linear with Origin Pro 8, and the rate was determined by the initial rate method (up to 360 minutes). The slope of the linear fitting represents the reaction rate. These data were taken from the average of two independent experiments.

Table S3 Concentration of product **3aa** at different time intervals using $(thf)_2NiBr_2$/bpy and (bpy)$NiBr_2$.

$(thf)_2NiBr_2$/bpy		(bpy)$NiBr_2$	
Time (min)	Conc. of **3aa** [M]	Time (min)	Conc. of **3aa** [M]
10	0.003	30	0.004
20	0.007	60	0.010
30	0.012	90	0.015
40	0.017	120	0.020
50	0.025	150	0.026
60	0.033	180	0.031
90	0.057	210	0.036
120	0.082	240	0.042
		300	0.052
		360	0.063

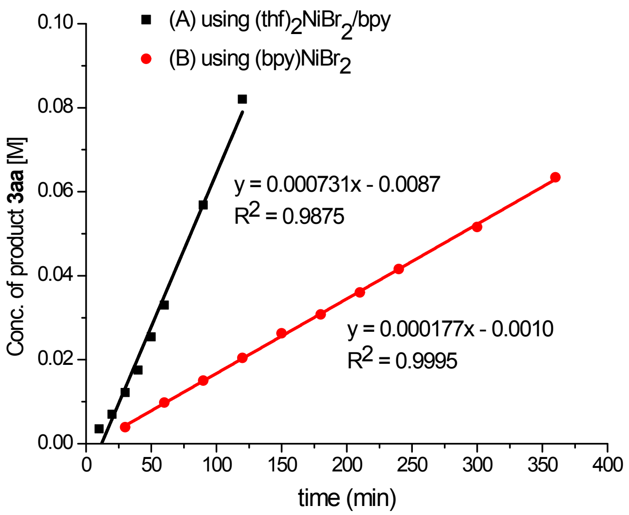

Figure S6. Time-dependent formation of **3aa** using (A) $(thf)_2NiBr_2$/bpy system and (B) $(bpy)NiBr_2$ catalyst.

12.2 Kinetics for electronic effect study

Procedure for reaction rates with different indole derivatives: Representative procedure of the kinetic experiment (Sec 12.1.1) was followed using $(thf)_2NiBr_2$ (0.0037 g, 0.01 mmol, 0.01 M), bpy (0.0016 g, 0.01 mmol), LiHMDS (0.067 g, 0.40 mmol), substrate 5-methyl-1-(pyridin-2-yl)-1*H*-indole (**1b**; 0.042 g, 0.20 mmol, 0.2 M) or 5-fluoro-1-(pyridin-2-yl)-1*H*-indole (**1d**; 0.042 g, 0.20 mmol, 0.2 M), 1-chlorooctane (0.0.059 g, 0.40 mmol, 0.4 M) and *n*-hexadecane (0.025 mL, 0.085 mmol, 0.085 M, internal standard), and toluene (0.87 mL). The concentration of the product **3ba** (or **3da**) obtained in each sample was determined with respect to the internal standard *n*-hexadecane. The data were collected till 120 min. The data of the concentration of the product *vs* time (min) plot was drawn (Figure S7) and fitted linear with Origin Pro 8, and the rate was determined by the initial rate method (up to 120 minutes). The slope of the linear fitting represents the reaction rate. These data were taken from the average of three independent experiments.

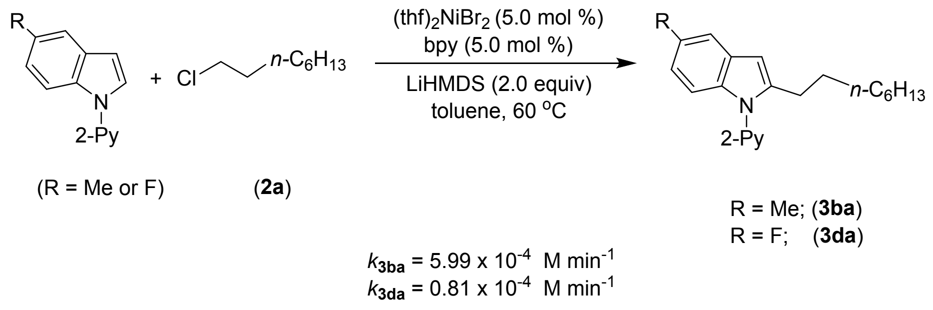

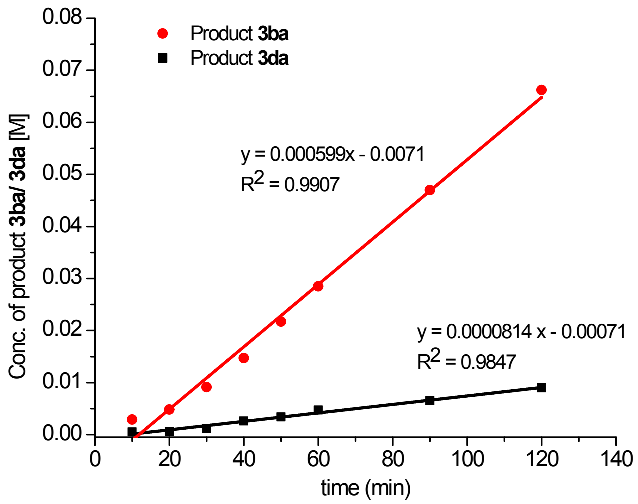

Figure S7. Time-dependent formation of products **3ba** and **3da**.

Procedure for the reaction rates with different octyl halides: Representative procedure of kinetic experiment (Sec 12.1.1) was followed using $(thf)_2NiBr_2$ (0.0037 g, 0.01 mmol, 0.01 M), bpy (0.0016 g, 0.01 mmol), LiHMDS (0.067 g, 0.40 mmol), substrate **1a** (0.039 g, 0.20 mmol, 0.2 M) 1-bromooctane (0.077 g, 0.40 mmol, 0.4 M) or 1-iodooctane (0.096 g, 0.40 mmol, 0.4 M) and *n*-hexadecane (0.025 mL, 0.085 mmol, 0.085 M, internal standard), and toluene (0.87 mL). The concentration of the product **3aa** obtained in each sample was determined with respect to the internal standard *n*-hexadecane. The data were collected till 120 min. The data of the concentration of the product *vs* time (min) plot was drawn (Figure S8) and

fitted linear with Origin Pro 8, and the rate was determined by the initial rate method (up to 120 minutes). The slope of the linear fitting represents the reaction rate. These data were taken from the average of three independent experiments.

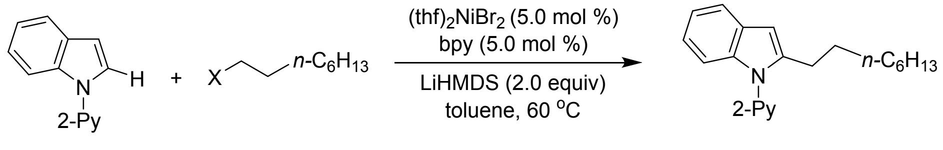

(**1a**) (**3aa**)

X = Cl; k_{3aa} = 0.59 x 10^{-3} M min^{-1}
X = Br; k_{3aa} = 2.62 x 10^{-3} M min^{-1}
X = I; k_{3aa} = 4.52 x 10^{-3} M min^{-1}

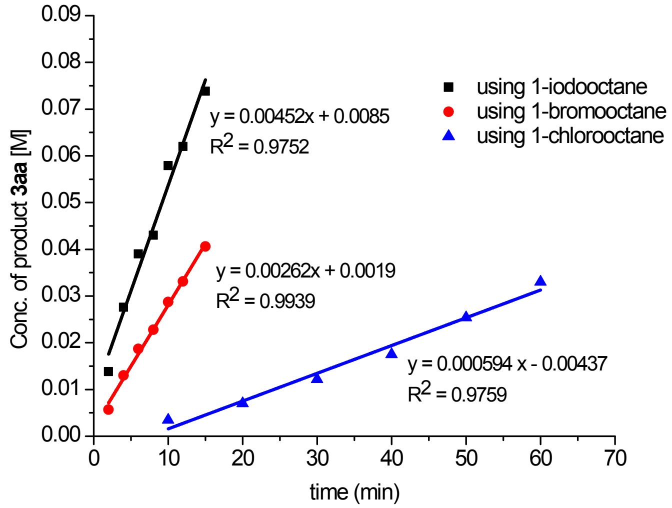

Figure S8. Time-dependent formation of products **3aa** using different *n*-octyl halides.

13. Deuterium labeling experiments

Procedure for kinetic isotope effect (KIE) study: Representative procedure of kinetic experiment (Sec 12.1.1) was followed using $(thf)_2NiBr_2$ (0.0037 g, 0.01 mmol, 0.01 M), bpy (0.0016 g, 0.01 mmol), LiHMDS (0.067 g, 0.40 mmol), indole **1a** (0.039 g, 0.20 mmol, 0.2 M) or

[2-D]-**1a** (0.039 g, 0.20 mmol, 0.2 M), and 1-chlorooctane (0.0.059 g, 0.40 mmol, 0.4 M), and *n*-hexadecane (0.025 mL, 0.085 mmol, 0.085 M, internal standard), and toluene (0.87 mL). The concentration of the product **3aa** obtained in each sample was determined with respect to the internal standard *n*-hexadecane. The data of the concentration of the product *vs* time (min) plot was drawn (Figure S9) and fitted linear with Origin Pro 8, and the rate was determined by initial rate method (up to 120 minutes). The slope of the linear fitting represents the reaction rate.

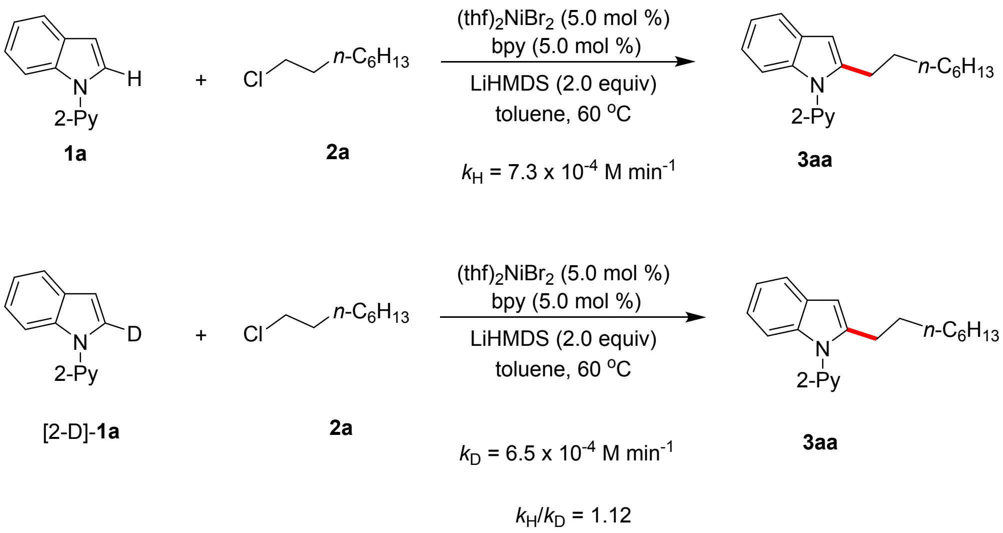

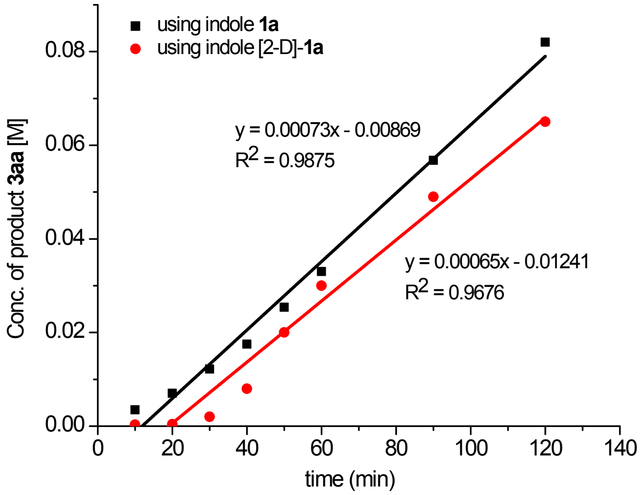

Figure S9. Time-dependent formation of product **3aa** using indoles **1a** and [2-D]-**1a**.

Procedure for H/D scrambling experiment (intermolecular): To a screw-capped tube equipped with magnetic stir bar were introduced 1-(pyridine-2-yl)-1*H*-indole-2-*d* ([2-D]-**1a**; 0.019 g, 0.10 mmol), 5-methoxy-1-(pyridine-2-yl)-1*H*-indole (**1c**; 0.022 g, 0.10 mmol), 1-chlorooctane (**2a**; 0.059 g, 0.40 mmol), $(thf)_2NiBr_2$ (0.0037 g, 0.01 mmol, 0.01 M), bpy (0.0016 g, 0.01 mmol), LiHMDS (0.067 g, 0.40 mmol) inside the glove-box. To the above mixture toluene (1.0 mL) was added and the resultant reaction mixture was stirred at 60 °C in a preheated oil bath for 60 min. At ambient temperature, the reaction mixture was quenched with distilled H_2O (10 mL). The crude product was then extracted with EtOAc (20 mL x 3). The combined organic extract was dried over Na_2SO_4 and the volatiles were evaporated in *vacuo*. The remaining residue was subjected to column chromatography on neutral alumina (petroleum ether/EtOAc: 20/1) to recover the starting compounds. The ^{1}H NMR analysis of the recovered compound **1c** shows 29% incorporation of deuterium at the C(2)–H, whereas compound [2-D]-**1a** shows 35% loss of deuterium (Figure S10).

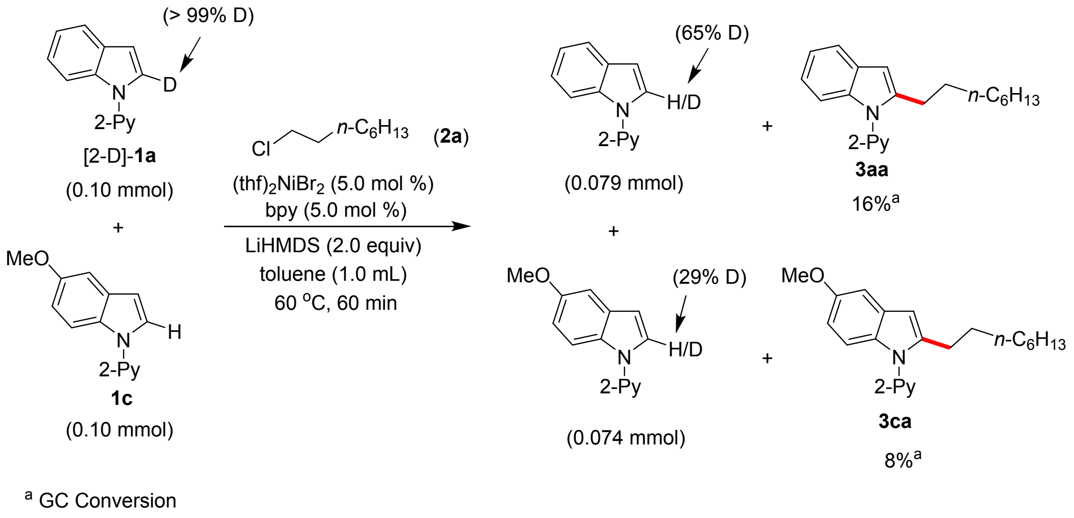

Figure S10. H/D scrambling experiment.

Procedure for deuterium incorporation experiment (using D_2O): To a screw-capped tube equipped with magnetic stir bar were introduced 1-(pyridine-2-yl)-1*H*-indole (**1a**; 0.039 g, 0.20 mmol), 1-chlorooctane (0.059 g, 0.40 mmol), $(thf)_2NiBr_2$ (0.0037 g, 0.01 mmol, 0.01 M), bpy (0.0016 g, 0.01 mmol), LiHMDS (0.067 g, 0.40 mmol) inside the glove-box. To the above mixture toluene (1.0 mL) was added and the resultant reaction mixture was stirred at 60 °C in a preheated oil bath for 30 min. At ambient temperature, D_2O (1.0 mL) was added to the reaction mixture under argon and was stirred for 1 h. Reaction mixture was quenched with distilled H_2O (10 mL). The crude product was then extracted with EtOAc (20 mL x 3). The combined organic extract was dried over Na_2SO_4 and the volatiles were evaporated in *vacuo*. The ^1H NMR analysis of the recovered compound does not show incorporation of deuterium at the C(2)–H of indole **1a** (Figure S11). This experiment in the absence of nickel catalyst $(thf)_2NiBr_2$/bpy also shows the same result.

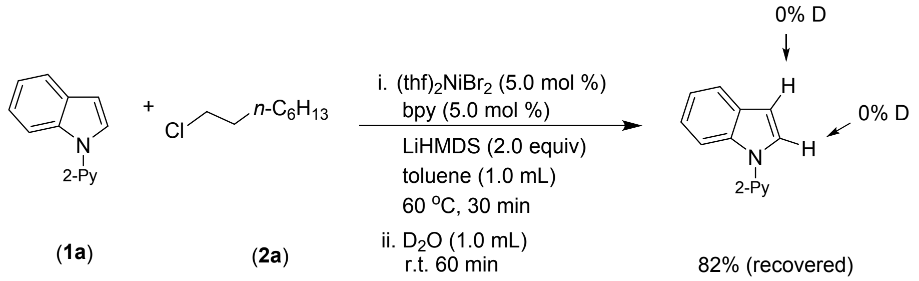

Figure S11. Deuterium incorporation experiment.

14. Procedure for EPR study

Representative procedure: To a flame-dried screw-cap tube equipped with magnetic stir bar were introduced 1-(pyridin-2-yl)-1*H*-indole (**1a**; 0.019 g, 0.10 mmol), 1-chlorooctane (**2a**; 0.030 g, 0.20 mmol), $(thf)_2NiBr_2$ (0.011 g, 0.03 mmol, 30.0 mol %), bpy (0.005 g, 0.03 mmol, 30.0 mol %) and LiHMDS (0.033 g, 0.20 mmol) inside the glove box. To the above mixture in the tube was added toluene (1.0 mL) and the resultant reaction mixture was immersed in a preheated oil bath at 60 °C and stirred for 30 min. At ambient temperature, the reaction tube was transferred to the glove box, and the reaction mixture was transferred to an EPR tube and frozen at 100 K, which was then subjected to EPR measurement.

The representative procedure was followed to perform EPR experiments for other controlled reactions:

(i) $(thf)_2NiBr_2$ + bpy

(ii) $(thf)_2NiBr_2$ + bpy + LiHMDS

(iii) $(thf)_2NiBr_2$ + bpy + LiHMDS + indole **1a**

(iv) $(thf)_2NiBr_2$ + bpy + LiHMDS + 1-chlorooctane (**2a**)

EPR for external standard: The $Cu(NO_3)_2.3H_2O$ (0.0072 g, 0.03 mmol) was dissolved in DMF (1.0 mL) and was transferred to an EPR tube and frozen at 100 K, which was then subjected to EPR measurement (Figure S12).

The intensity of the EPR spectrum obtained from the mixture $(thf)_2NiBr_2$ + bpy + LiHMDS (presumed intermediate **A**) was approximately 18% compared to that of the standard $Cu(NO_3)_2.3H_2O$.

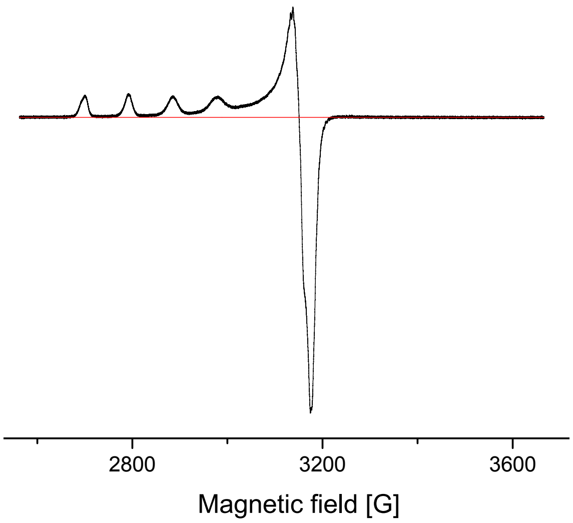

Figure S12. EPR spectrum of standard complex, $Cu(NO_3)_2.3H_2O$.

15. Procedure for XPS analysis

Representative procedure: To a flame-dried screw-cap tube equipped with magnetic stir bar were introduced 1-(pyridin-2-yl)-*1H*-indole (**1a**; 0.019 g, 0.10 mmol), 1-chlorooctane (**2a**; 0.030 g, 0.20 mmol), $(thf)_2NiBr_2$ (0.009 g, 0.025 mmol), bpy (0.004 g, 0.025 mol) and LiHMDS (0.033 g, 0.2 mmol) inside the glove box. To the above mixture in tube was added toluene (0.5 mL). The resultant reaction mixture in the tube was immersed in a preheated oil bath at 60 ^{o}C and stirred for 30 min. At ambient temperature, the reaction tube was transferred to the glove box. The sample for XPS analysis was prepared inside the glove box. After sample preparation, the sample was transferred to a vacuum transfer module which was subsequently evacuated in the antechamber of the glove box. The samples were loaded onto the spectrometer using this vacuum transfer module and subsequently pumped down by turbo molecular pumps connected to the load lock chamber. This allowed efficient transfer of the samples without being exposed to the atmosphere. The spectra were collected using Thermo Kalpha^{+} spectrometer with a mono chromated Al Kα X-ray source with energy 1486.6 eV. The pass energy for the acquisition was

50 eV for the individual core-level. All the spectral acquisition was done in the presence of ultra-low energy co-axial electron gun for charge compensation. The peak fitting of the individual core-levels were done using Avantage software with a Shirley type background.

The representative procedure was followed to perform XPS experiments for other controlled reaction (Figure 2 and Figures S13- S15):

(i) $(COD)_2Ni$: peak value 852.6 eV (Ni^0) [Figure S13 (B)]

(ii) $(Ph_3P)_3NiCl$: peak value 853.4 eV (Ni^I) [Figure S13 (A)]

(iii) $(thf)_2NiBr_2$: peak value 856.0 eV (Ni^{II}) [Figure S14 (A)]

(iv) $(thf)_2NiBr_2$ + bpy + LiHMDS + 1-chlorooctane (**2a**): peak value 856.0 eV (Ni^{II}) [Figure S14 (B)]

(v) Square planar complex, $[Et_2NCH_2C(O)$-(μ-N)-$C_9H_6N]Ni(OAc)$:[S14] peak value 855.6 eV (Figure S15)

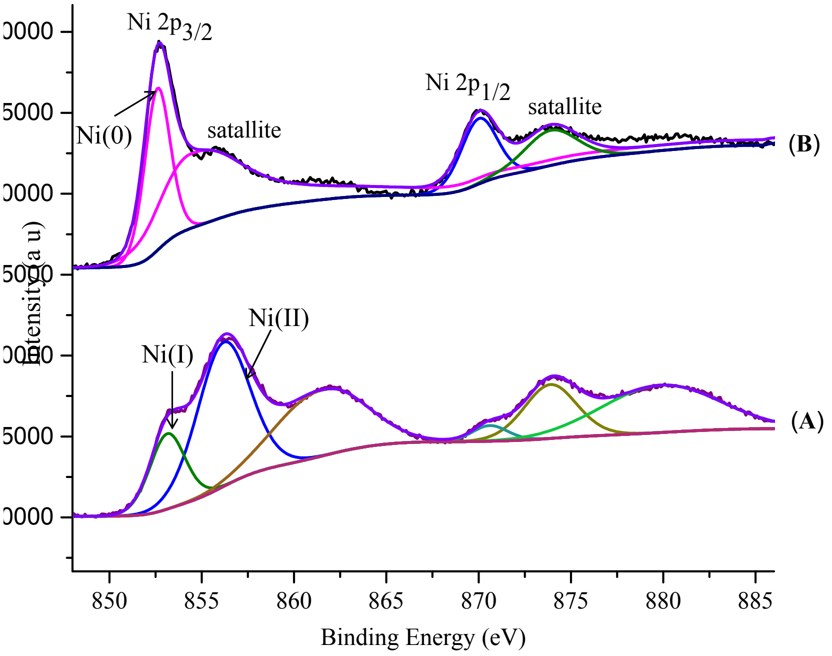

Figure S13. X-ray photoelectron spectra: (A) for $(Ph_3P)_3NiCl$ [shows some Ni(II) also], (B) for $Ni(COD)_2$.

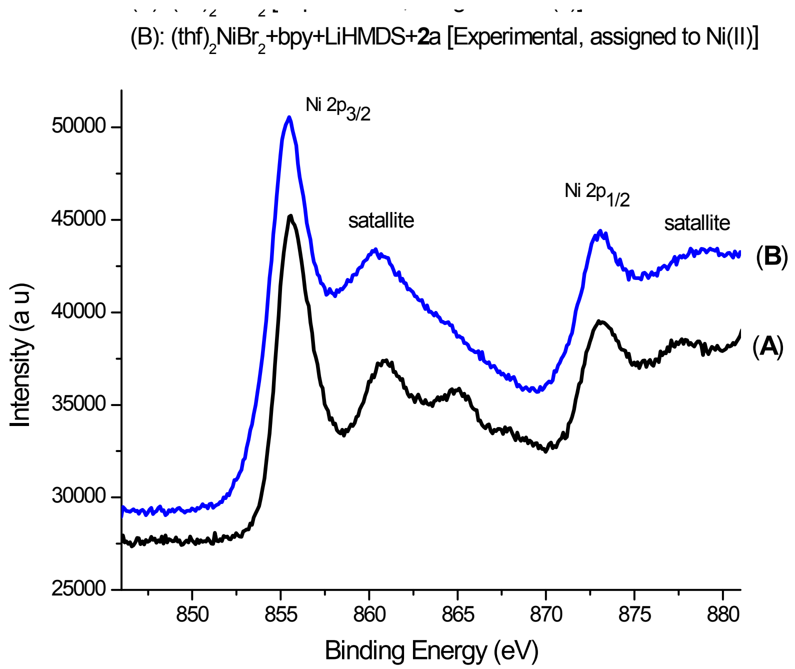

Figure S14. X-ray photoelectron spectra: (A) for $(thf)_2NiBr_2$, (B) $(thf)_2NiBr_2$ + bpy + LiHMDS + 1-chlorooctane (**2a**).

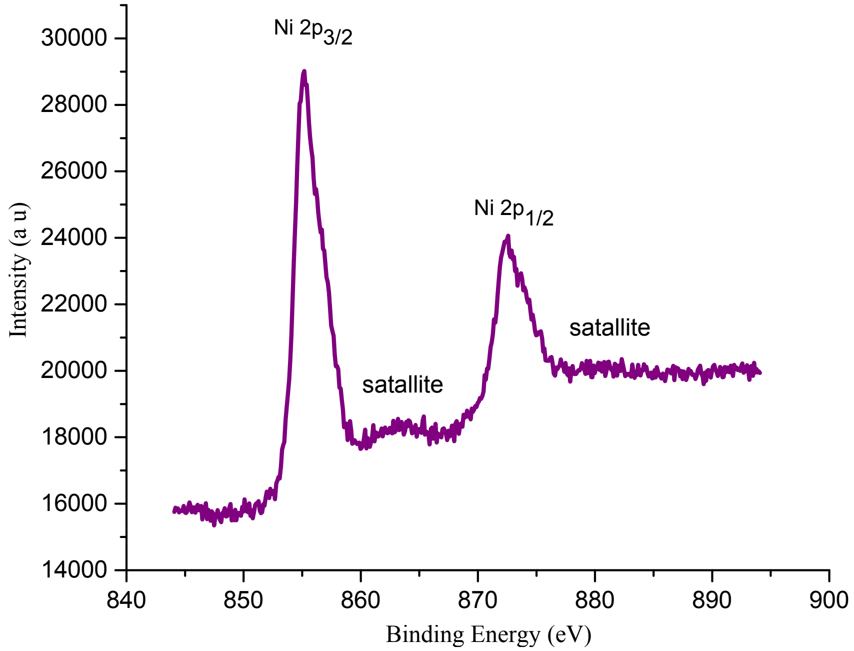

Figure S15. X-ray photoelectron spectrum of square planar complex, $[Et_2NCH_2C(O)$-(μ-N)-$C_9H_6N]Ni(OAc)$.[S14]

16. Computational method

The geometry of intermediate **A** (X = Br) is optimized under the framework of Density Functional Theory (DFT) using a linear combination of Gaussian orbitals as implemented in deMon 5.0 code.[S16] For all the calculations, PBE exchange correlation functional was used.[S17] Atomic orbitals of Ni are described through effective core potentials with ECP SD basis set for the valence orbitals.[S18] The H, C, N, Br atoms are treated with DZVP basis set. The local minima of the optimized geometry have been validated through the calculation of vibrational frequencies. All frequencies are found to be positive. The Gen-A2* auxiliary functions are used to fit the charge density.[S19] The structure having odd number of electron is considered to have a multiplicity of 2, for which Restricted Open shell Kohn Sham (ROKS) formalism is used. The EPR calculations are performed using ORCA 4.2.[S20]

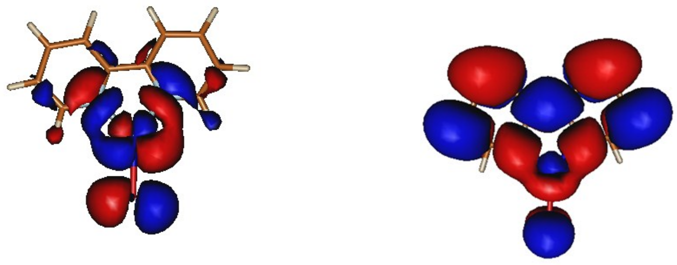

HOMO LUMO

Figure S16. HOMO LUMO of the intermediate **A** (X = Br).

17. Procedure for MALDI-TOF-MS experiment

To a Teflon-screw capped tube equipped with magnetic stir bar were introduced $(thf)_2NiBr_2$ (0.037 g, 0.10 mmol), bpy (0.016 g, 0.10 mmol), LiHMDS (0.033 g, 0.20 mmol), indole **1a** (0.039 g, 0.20 mmol) and 1-chlorooctane (0.030 g, 0.20 mmol). Toluene (1.0 mL) was added to the reaction mixture and stirred at 60 ^{o}C for 15 min in a pre-heated oil bath. At ambient temperature, an aliquot of sample was withdrawn and subjected for the MALDI-TOF-MS analysis. The MALDI-TOF-MS was performed on ABSCIEX TOF/TOF TM5800 and 2,5-dihyroxybenzoic acid used as a matrix.

18. References

S1. G. A. Olah and B. P. Singh, *J. Am. Chem. Soc.*, 1982, **104**, 5168-5172.

S2. T. C. Atack and S. P. Cook, *J. Am. Chem. Soc.*, 2016, **138**, 6139-6142.

S3. T. Nguyen, T. Yang and M.-L. Go, *Bioorganic Med. Chem. Lett.*, 2014, **24**, 1830-1838.

S4. F. Foubelo, S. A. Saleh and M. Yus, *J. Org. Chem.*, 2000, **65**, 3478-3483.

S5. Vasu D., Yorimitsu H. and O. A., *Synthesis*, 2015, **47**, 3286-3291.

S6. S. A. Green, J. L. M. Matos, A. Yagi and R. A. Shenvi, *J. Am. Chem. Soc.*, 2016, **138**, 12779-12782.

S7. Y. Ashikari, T. Nokami and J.-I. Yoshida, *Org. Lett.*, 2012, **14**, 938-941.

S8. A. L. Silberstein, S. D. Ramgren and N. K. Garg, *Org. Lett.*, 2012, **14**, 3796-3799.

S9. A. R. O. Venning, P. T. Bohan and E. J. Alexanian, *J. Am. Chem. Soc.*, 2015, **137**, 3731-3734.

S10. X. Sun, B. Zhang, X. Li, C. O. Trindle and G. Zhang, *The J. Phys. Chem. A*, 2016, **120**, 5791-5797.

S11. (a) L. Ackermann and A. V. Lygin, *Org. Lett.*, 2011, **13**, 3332-3335; (b) Y.-C. Teo, F.-F. Yong and S. Sim, *Tetrahedron*, 2013, **69**, 7279-7284; (c) V. K. Tiwari, N. Kamal and M. Kapur, *Org. Lett.*, 2015, **17**, 1766-1769.

S12. (a) J. A. Broomhead and F. P. Dwyer, *Aust. J. Chem.*, 1961, **14**, 250-252; (b) M. Khrizanforov, V. Khrizanforova, V. Mamedov, N. Zhukova, S. Strekalova, V. Grinenko, T. Gryaznova, O. Sinyashin and Y. Budnikova, *J. Org. Chem.*, 2016, **820**, 82-88.

S13. L. Liu, X. Zhang, H. Liu, X. Zhang, G. Sun and H. Zhang, *J. Appl. Polym. Sci.*, 2014, **131**, 40511.

S14. V. Soni, R. A. Jagtap, R. G. Gonnade and B. Punji, *ACS Catal.*, 2016, **6**, 5666-5672.

S15. S. J. Hwang, D. C. Powers, A. G. Maher and D. G. Nocera, *Chem. Sci.*, 2015, **6**, 917-922.

S16. A. M. Koster, P. Calaminici, M. E. Casida, R. F. Moreno, G. Geudtner, A. Goursot, T. Heine, A. Ipatov, F. Janetzko, J. M. del Campo, S. Patchkovskii, J. U. Reveles, A. Vela and D. R. Salahub, *deMon2k deMon Developers*, 2006.

S17. J. P. Perdew, K. Burke and M. Ernzerhof, *Phy. Rev. Lett.*, 1996, **77**, 3865-3868.

S18. W. J. Stevens, H. Basch and M. Krauss, *J. Chem. Phys.*, 1984, **81**, 6026-6033.

S19. N. Godbout, D. R. Salahub, J. Andzelm and E. Wimmer, *Can. J. Chem.*, 1992, **70**, 560-571.

S20. F. Neese, ORCA – An Ab Initio, DFT. Semiempirical Program Package. Institute for Physical and Theoretical Chemistry, University of Bonn, Bonn, Germany, 2008.

19. MALDI-TOF spectra of Ni-intermediates

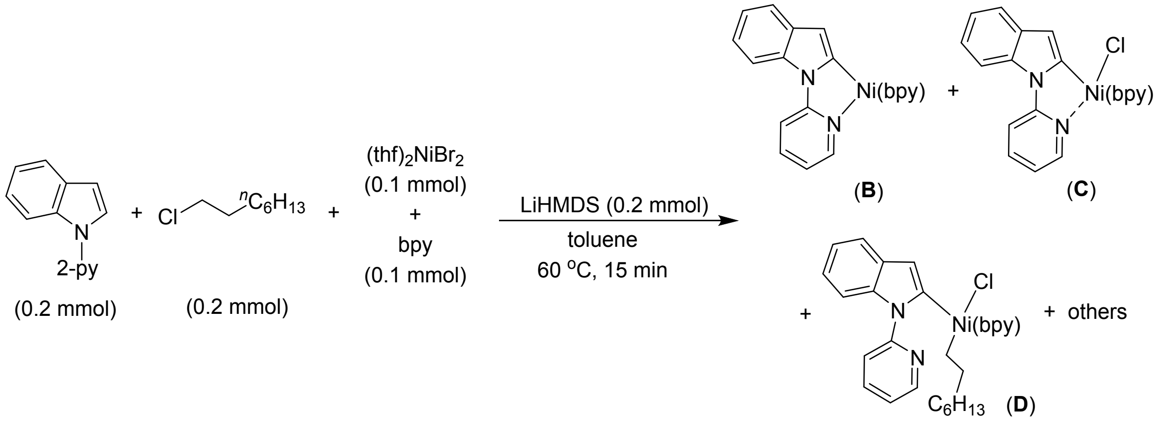

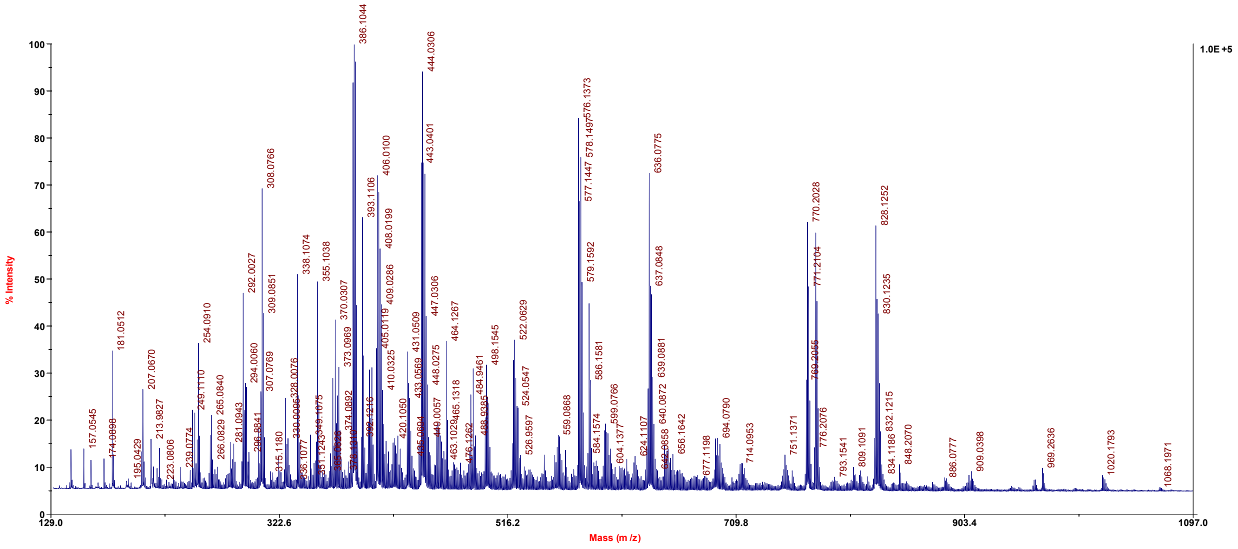

Expanded portion of MALDI-TOF-MS spectrum

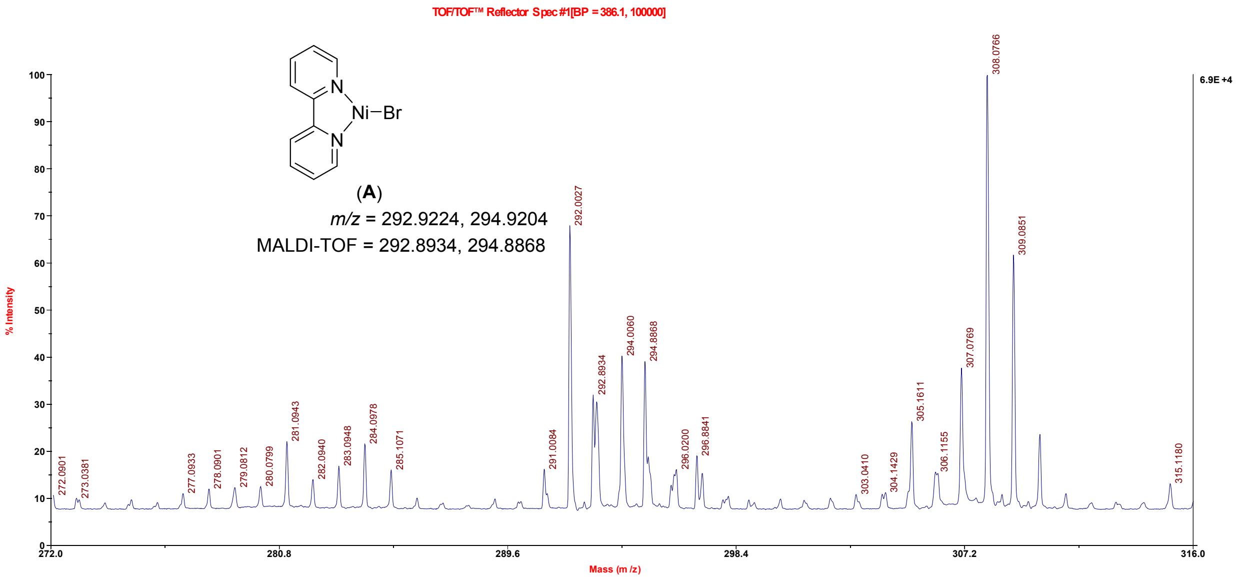

Expanded portion of MALDI-TOF-MS spectrum

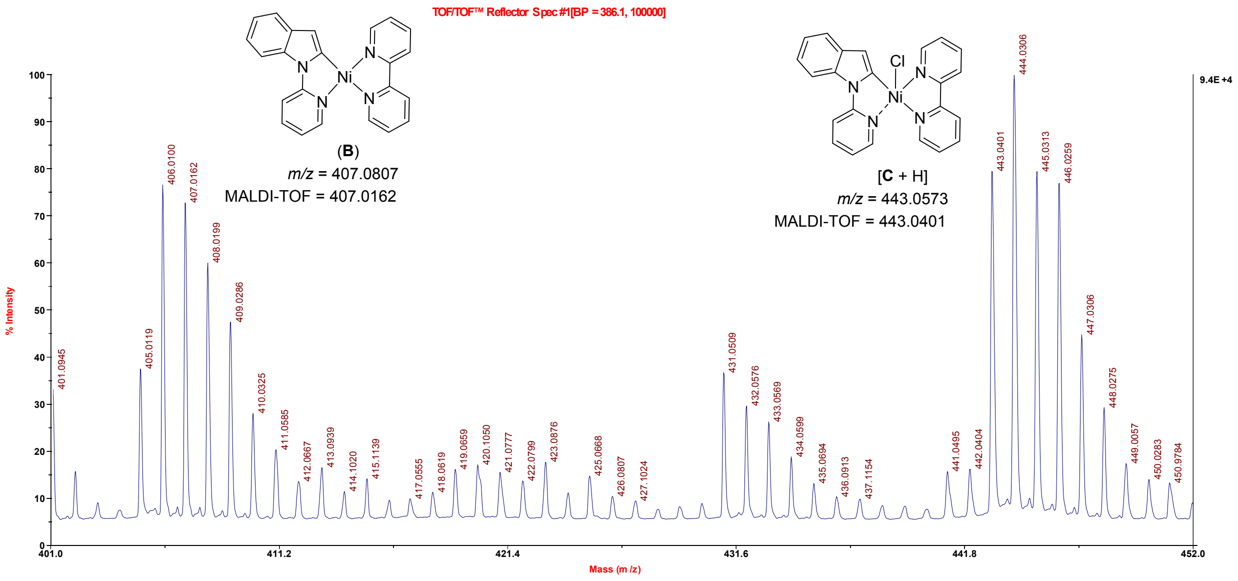

Expanded portion of MALDI-TOF-MS spectrum

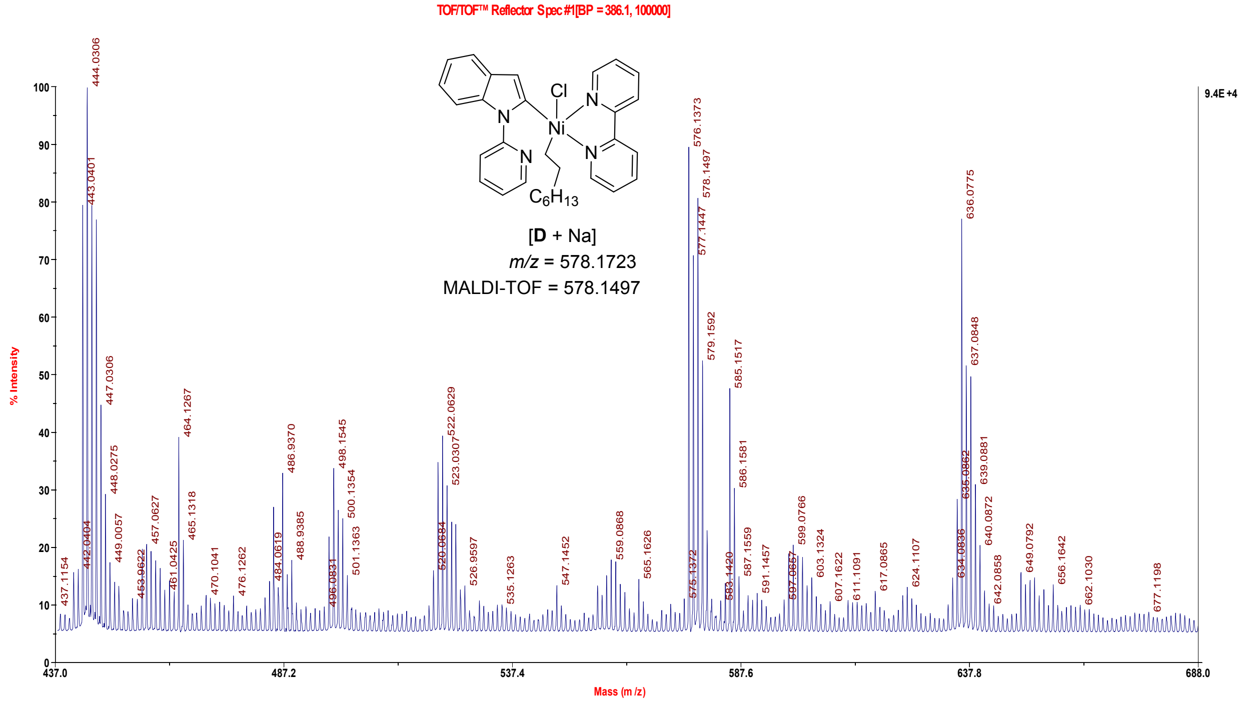

20. ^{1}H and ^{13}C NMR spectra

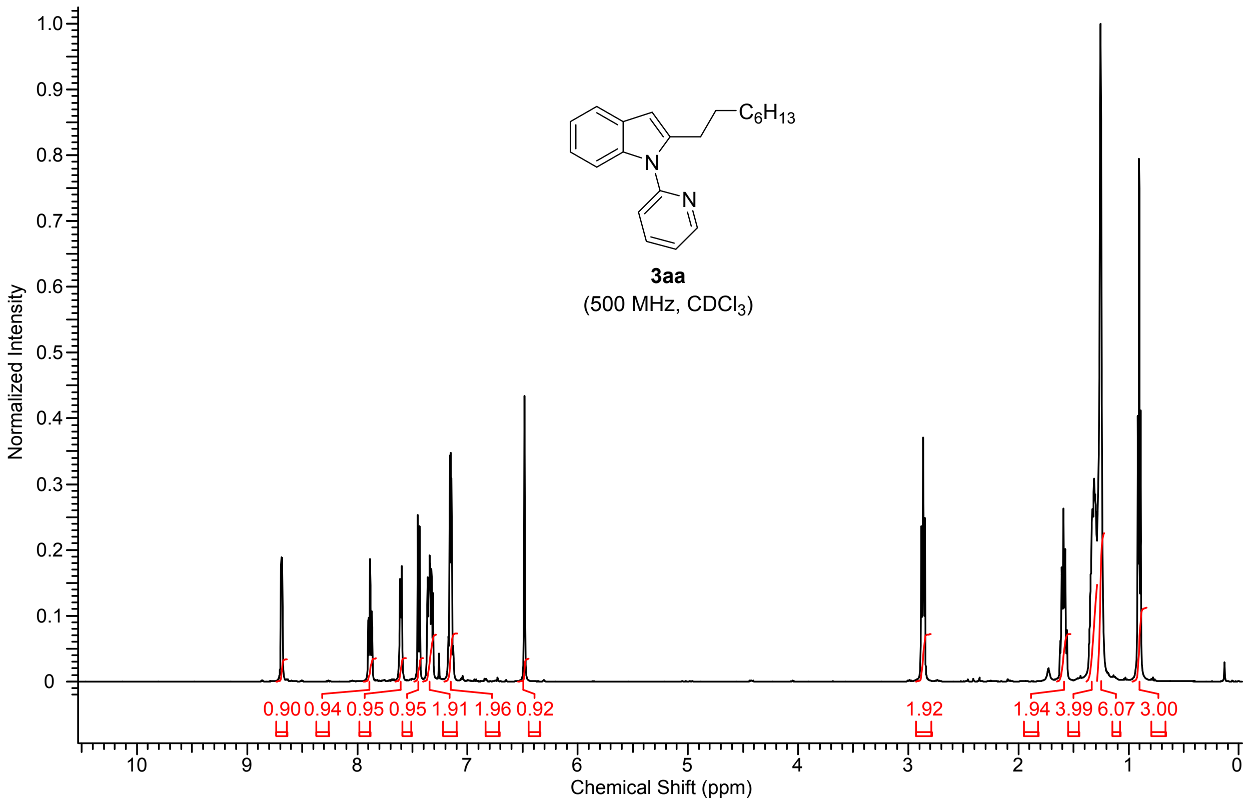

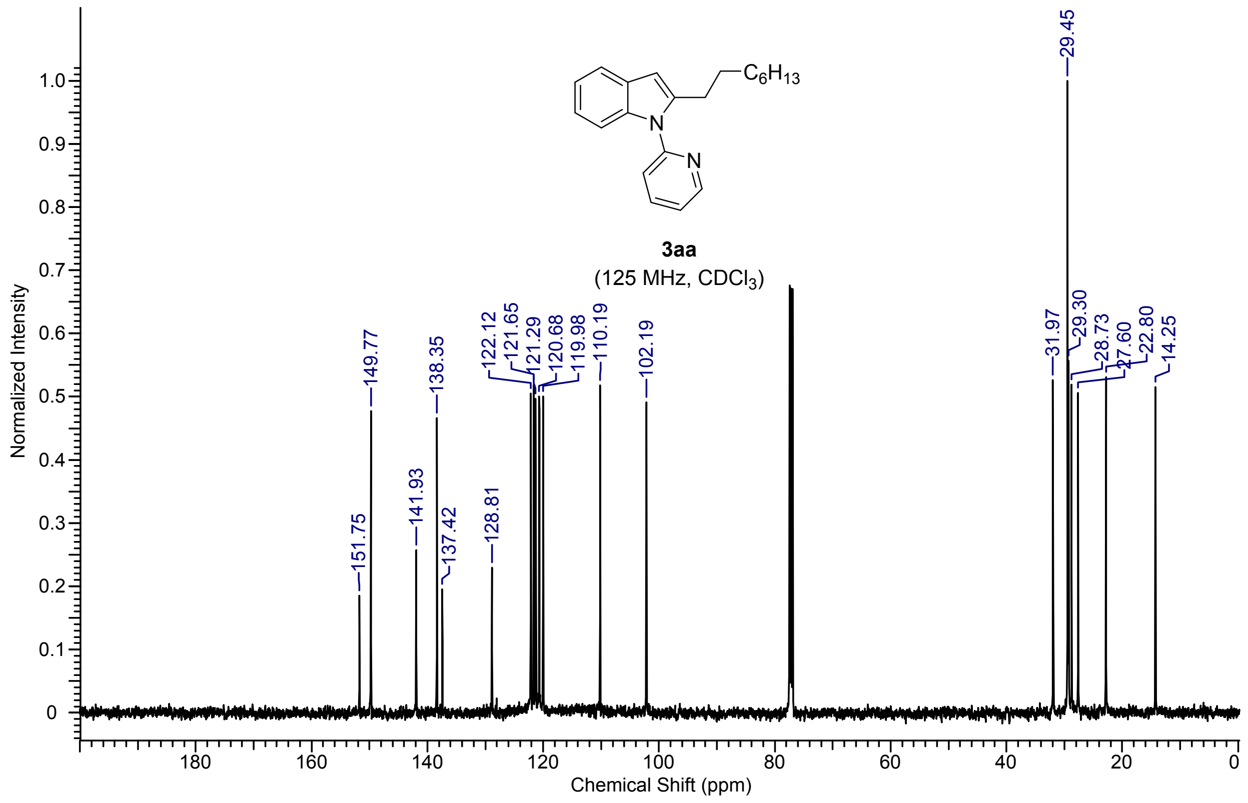

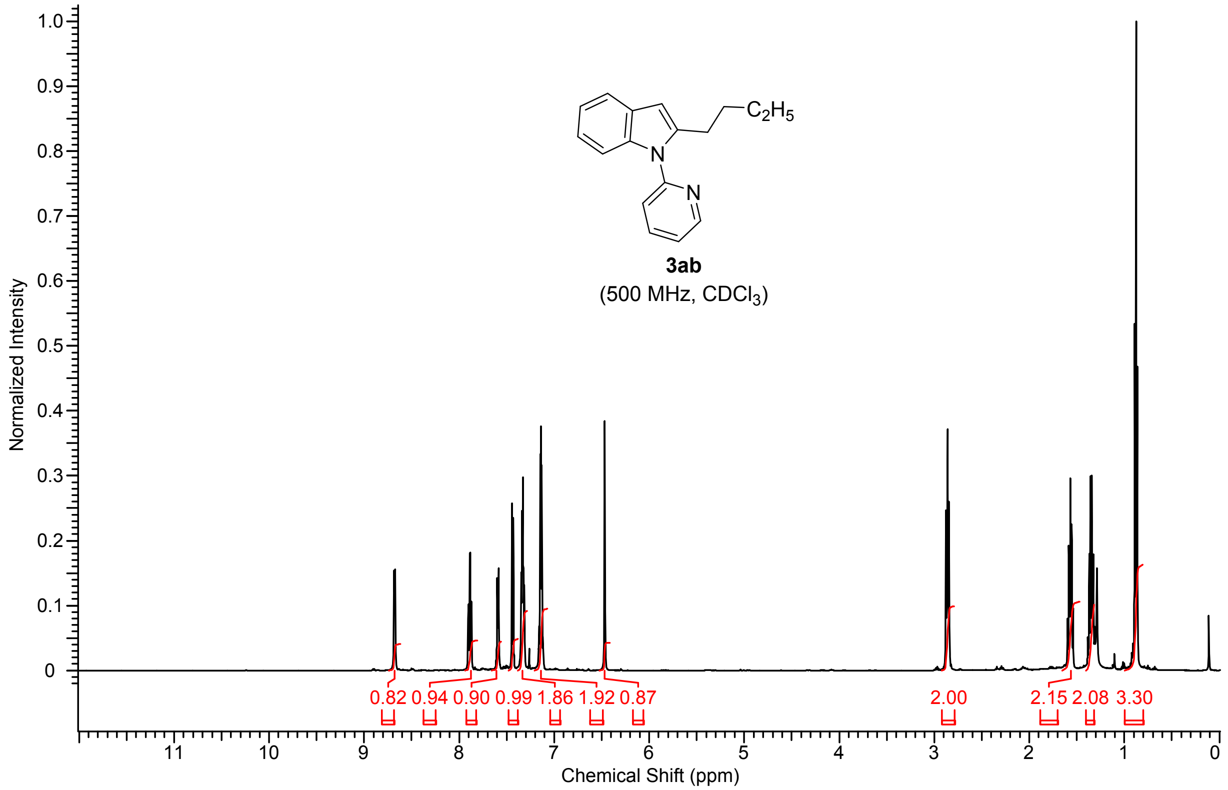

C2H5
N
N
3ab
(500 MHz, CDCl3)
0.82 0.94 0.90 0.99 1.86 1.92 0.87
2.00
2.15 2.08 3.30
Normalized Intensity
Chemical Shift (ppm)

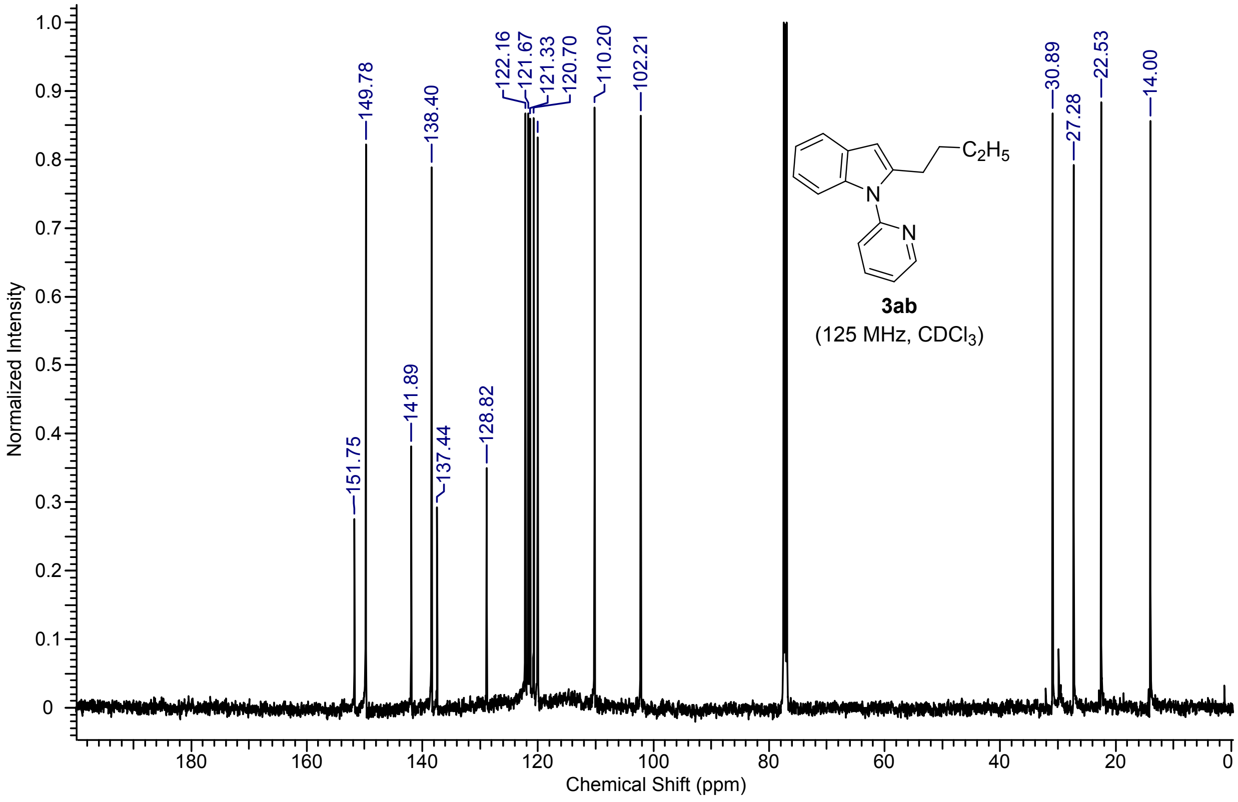

151.75
149.78
141.89
138.40
137.44
128.82
122.16
121.67
121.33
120.70
110.20
102.21
30.89
27.28
22.53
14.00
C2H5
N
N
3ab
(125 MHz, CDCl3)
Normalized Intensity
Chemical Shift (ppm)

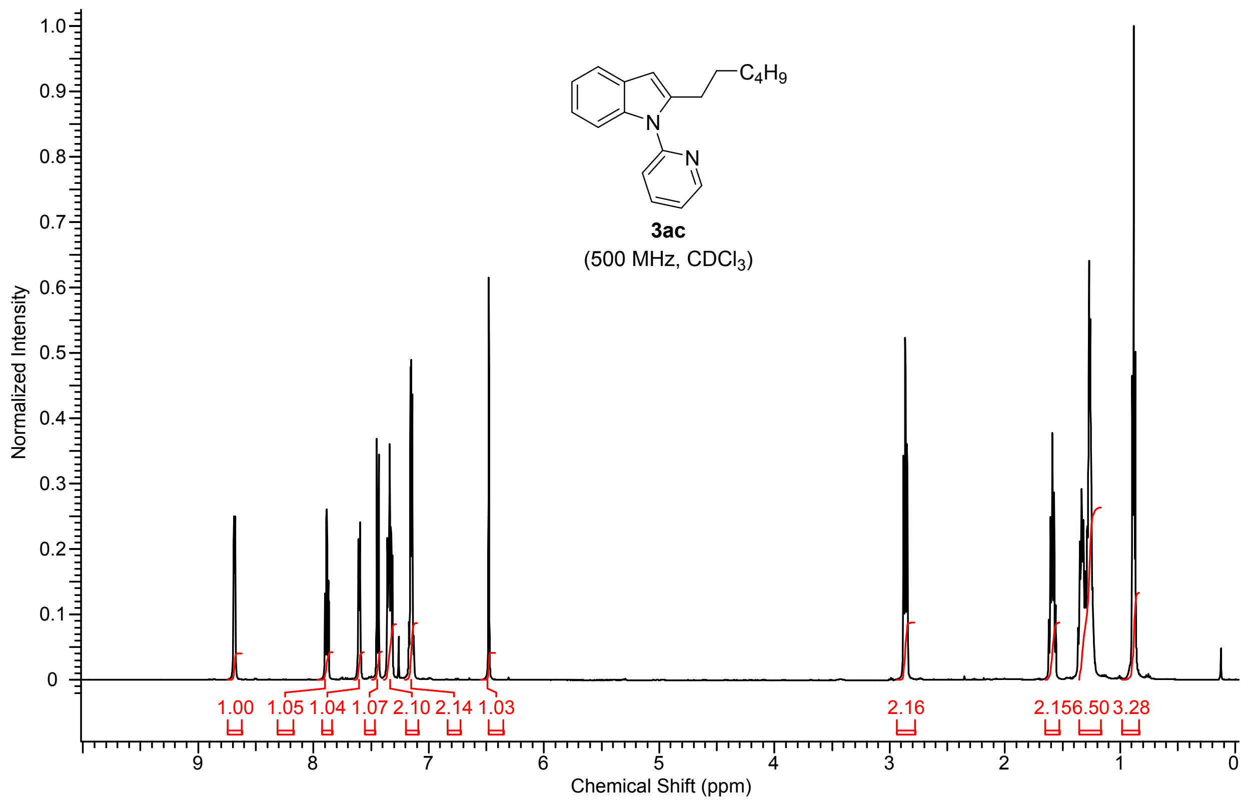
C4H9
N
N
3ac
(500 MHz, CDCl3)
Normalized Intensity
1.00 1.05 1.04 1.07 2.10 2.14 1.03 2.16 2.15 6.50 3.28
Chemical Shift (ppm)

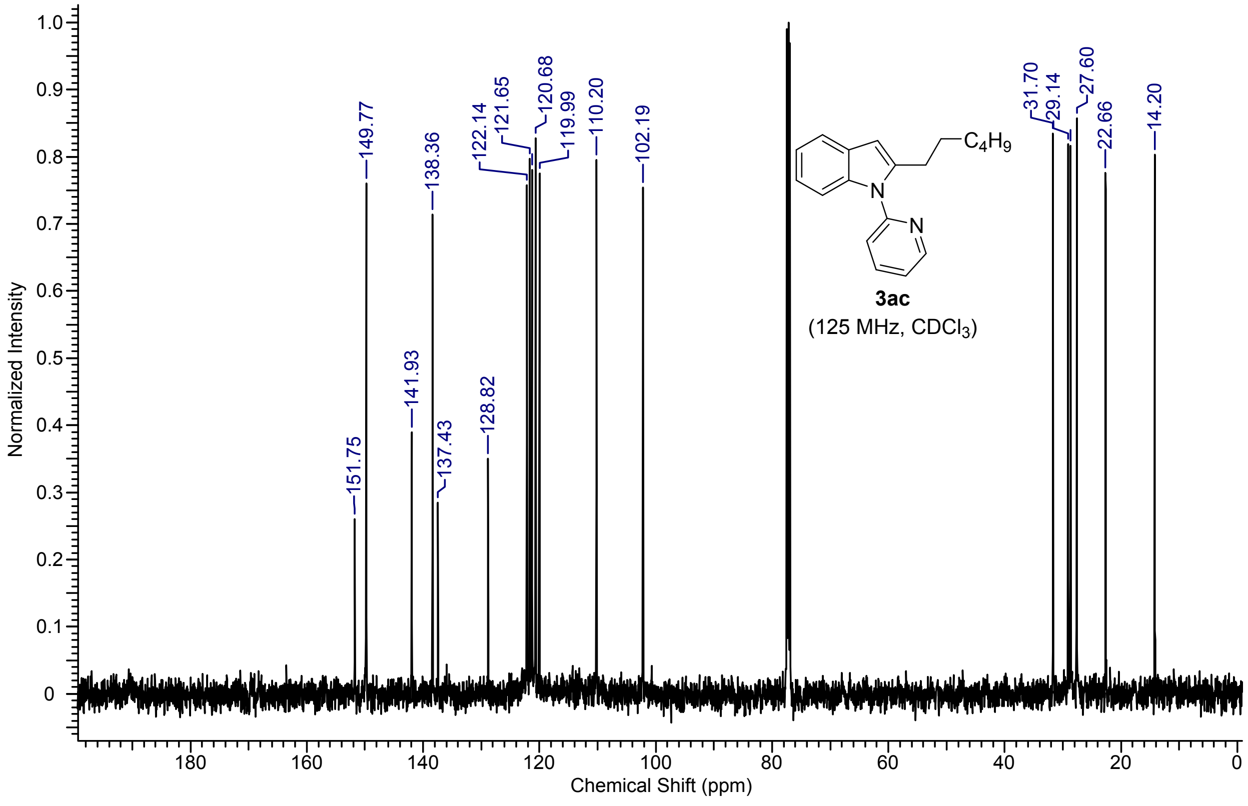
151.75
149.77
141.93
138.36
137.43
128.82
122.14
121.65
120.68
119.99
110.20
102.19
31.70
29.14
27.60
22.66
14.20
C4H9
N
N
3ac
(125 MHz, CDCl3)
Normalized Intensity
Chemical Shift (ppm)

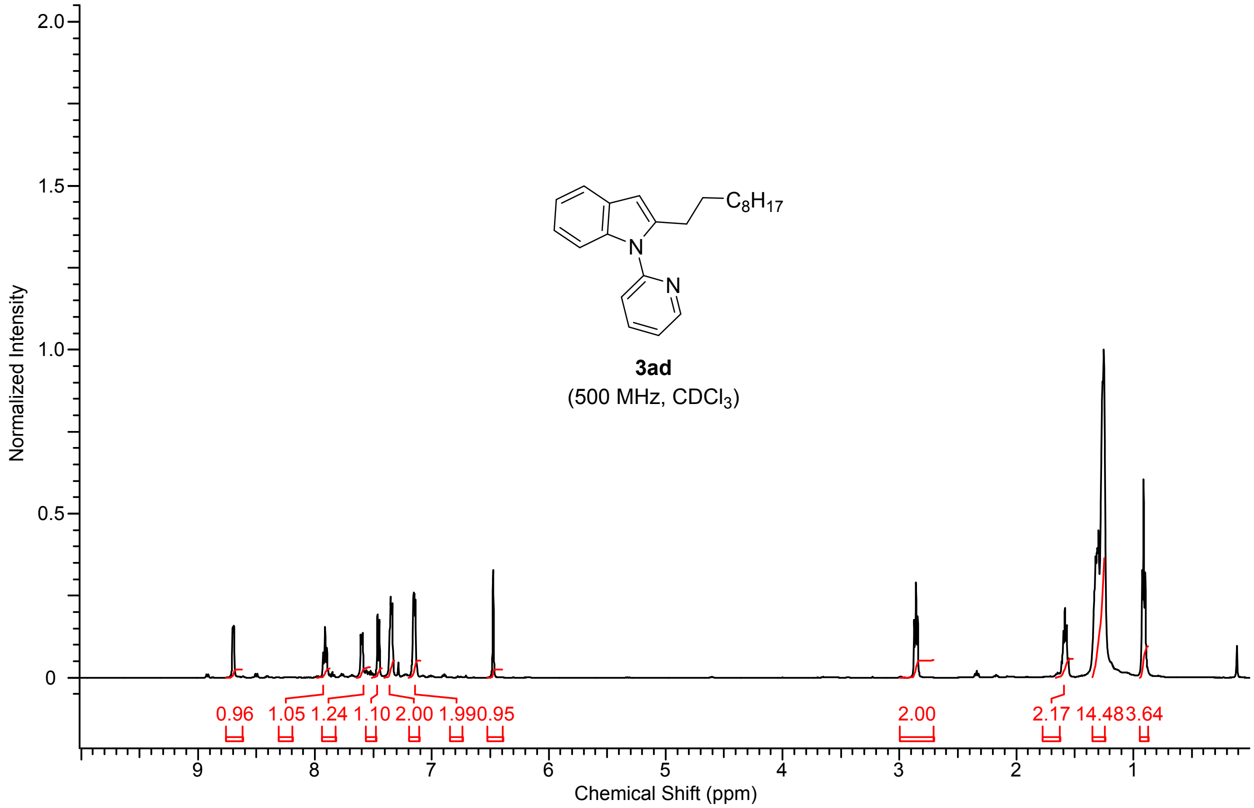
C8H17
N
N
3ad
(500 MHz, CDCl3)
Normalized Intensity
Chemical Shift (ppm)
0.96
1.05
1.24
1.10
2.00
1.99
0.95
2.00
2.17
14.48
3.64

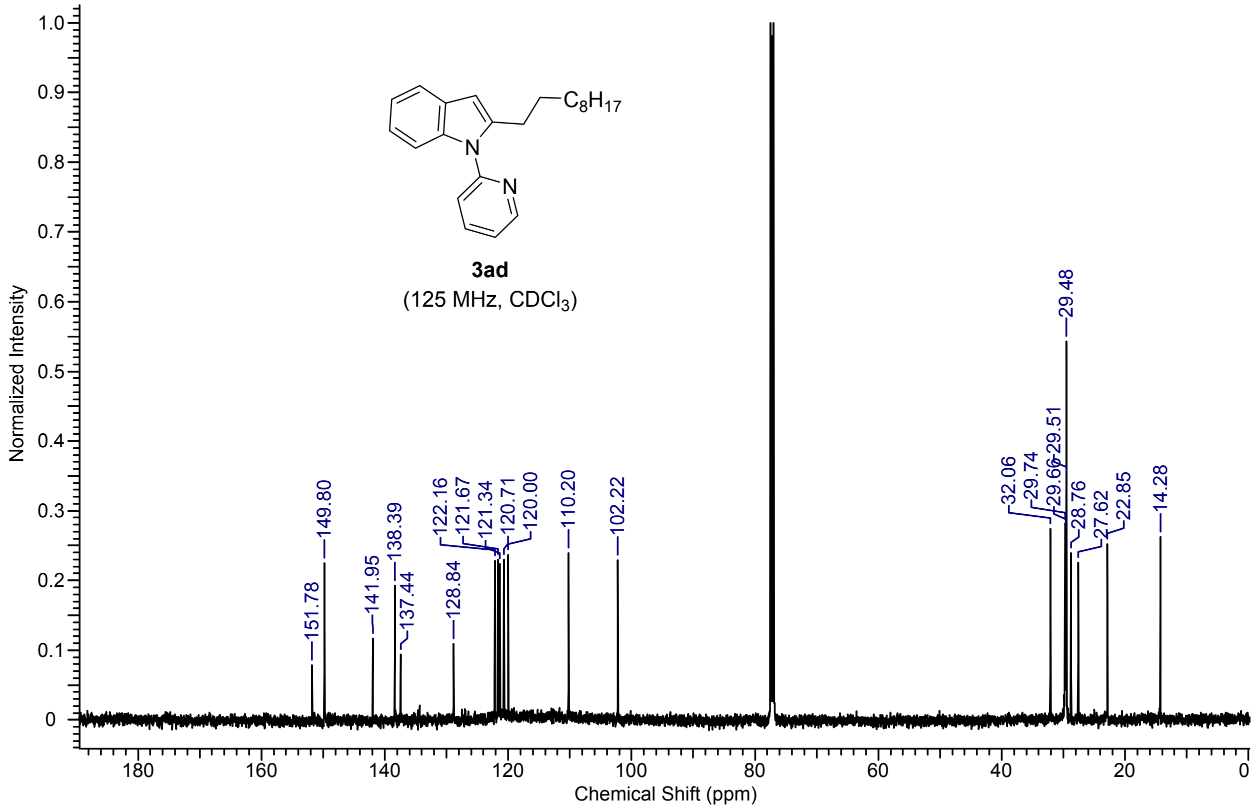
C8H17
N
N
3ad
(125 MHz, CDCl3)
Normalized Intensity
Chemical Shift (ppm)
151.78
149.80
141.95
138.39
137.44
128.84
122.16
121.67
121.34
120.71
120.00
110.20
102.22
32.06
29.74
29.66
29.51
29.48
28.76
27.62
22.85
14.28

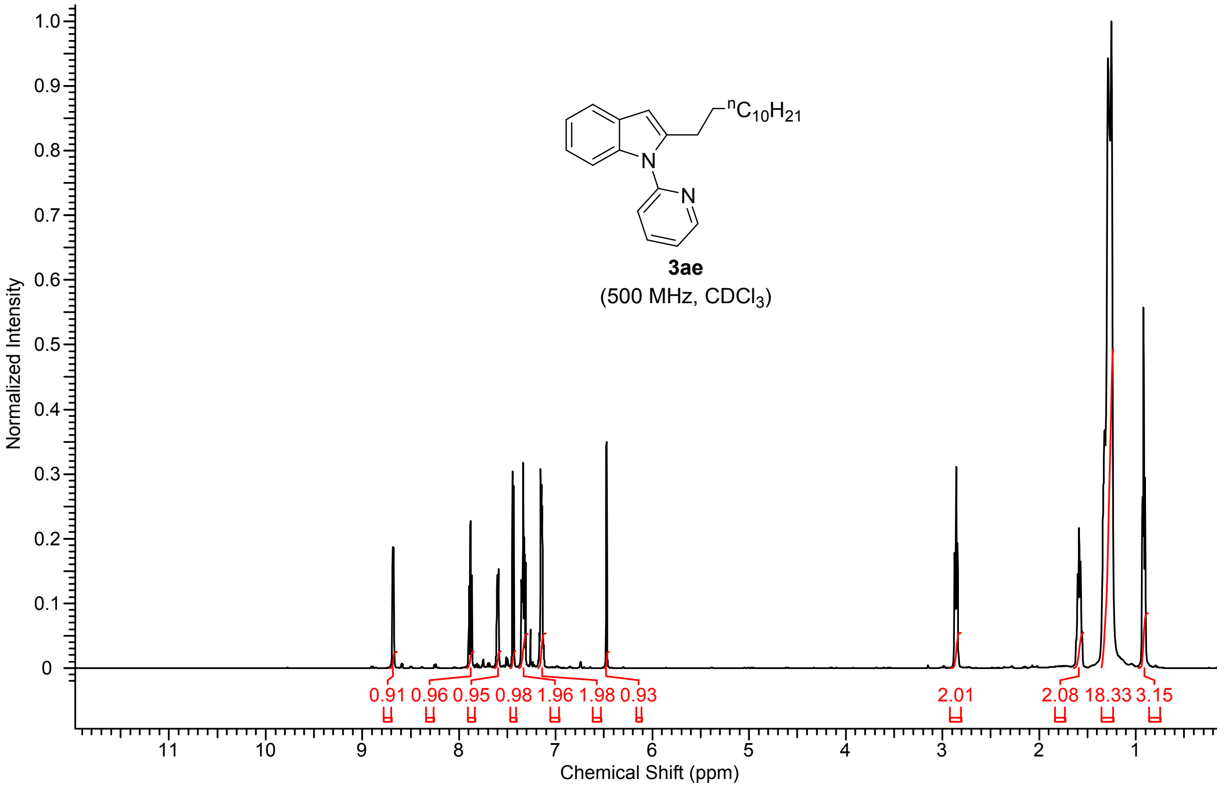
nC10H21
N
N
3ae
(500 MHz, CDCl3)
Normalized Intensity
Chemical Shift (ppm)
0.91 0.96 0.95 0.98 1.96 1.98 0.93
2.01
2.08 18.33 3.15

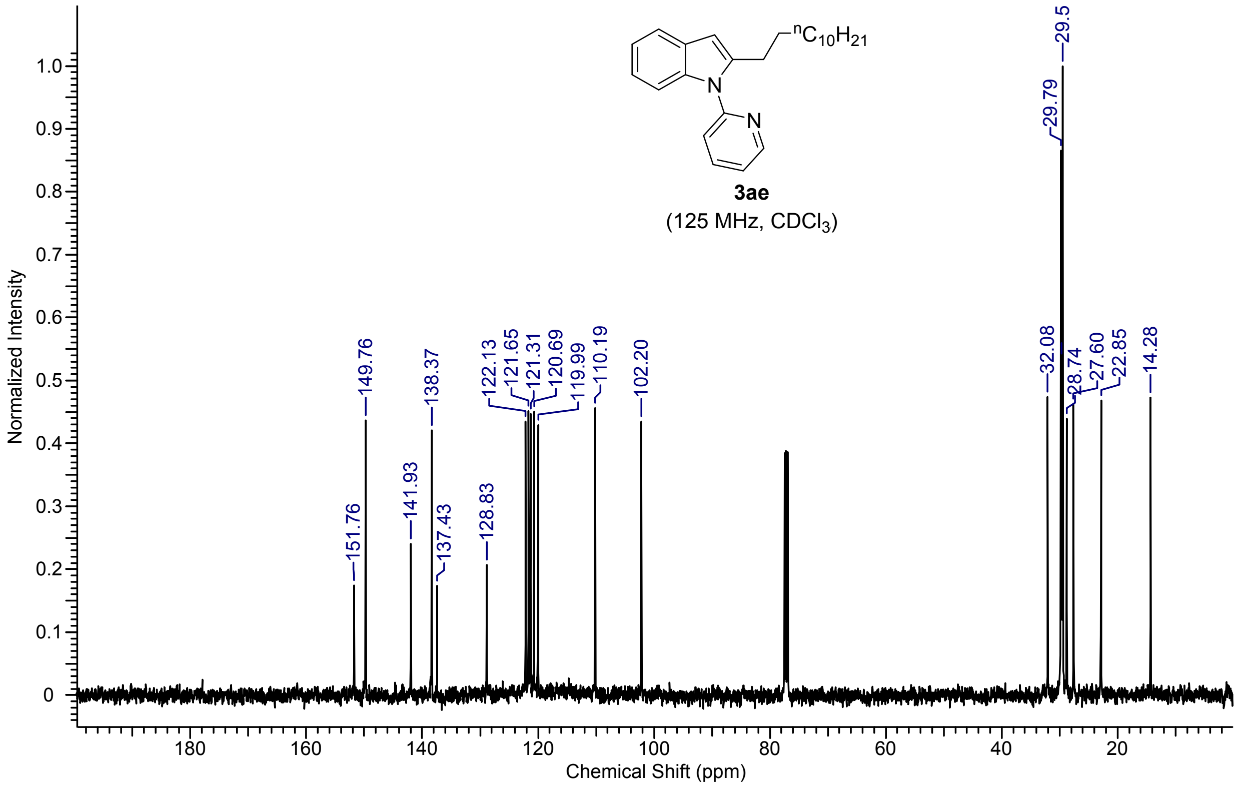
nC10H21
N
N
3ae
(125 MHz, CDCl3)
Normalized Intensity
Chemical Shift (ppm)
151.76
149.76
141.93
138.37
137.43
128.83
122.13
121.65
121.31
120.69
119.99
110.19
102.20
32.08
29.79
29.5
28.74
27.60
22.85
14.28

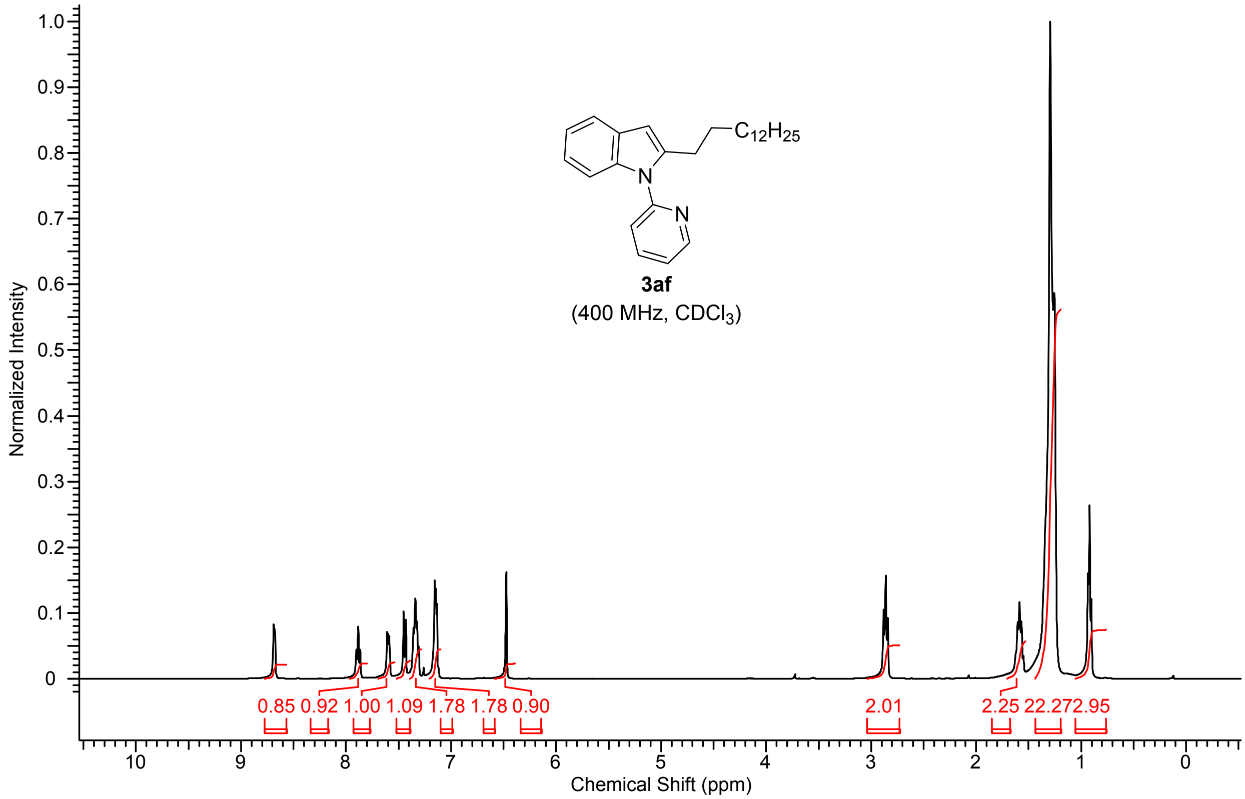

3af
(400 MHz, CDCl3)
Normalized Intensity
Chemical Shift (ppm)
0.85 0.92 1.00 1.09 1.78 1.78 0.90
2.01
2.25 22.27 2.95

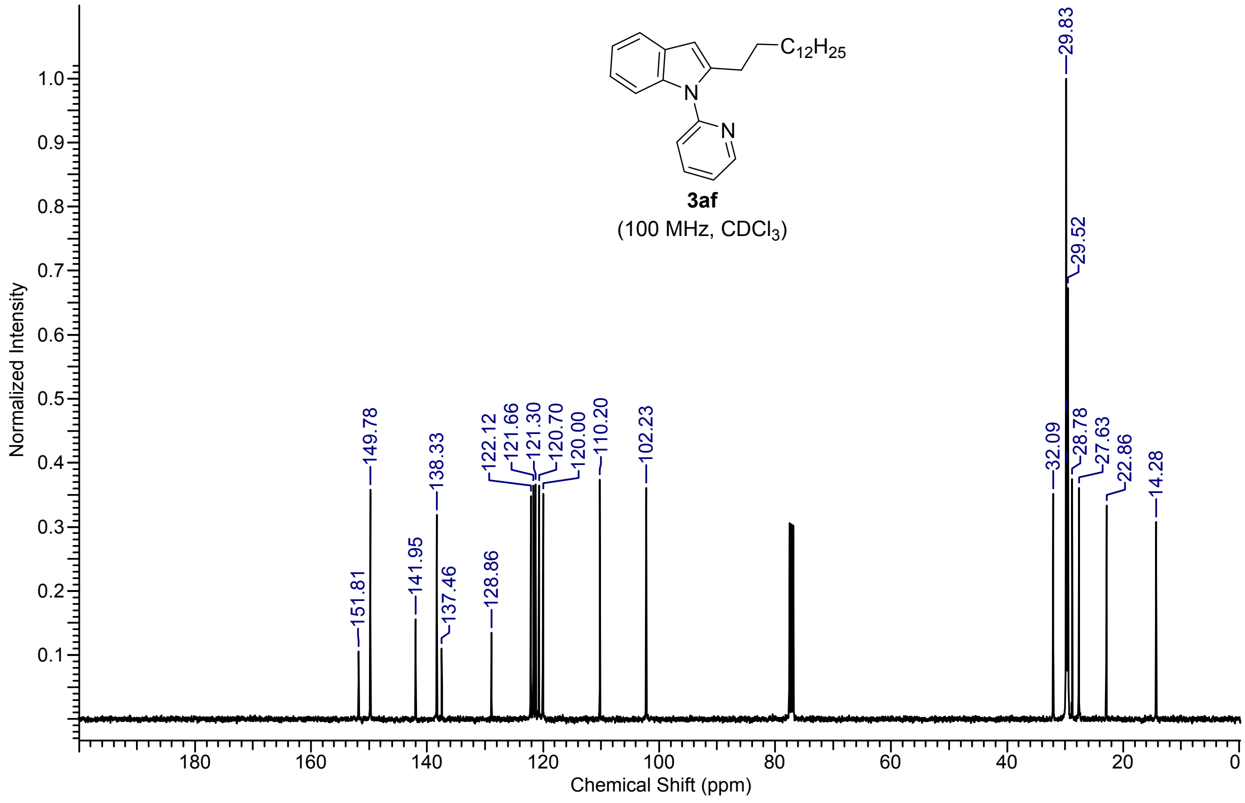

3af
(100 MHz, CDCl3)
Normalized Intensity
Chemical Shift (ppm)
151.81
149.78
141.95
138.33
137.46
128.86
122.12
121.66
121.30
120.70
120.00
110.20
102.23
32.09
29.83
29.52
28.78
27.63
22.86
14.28

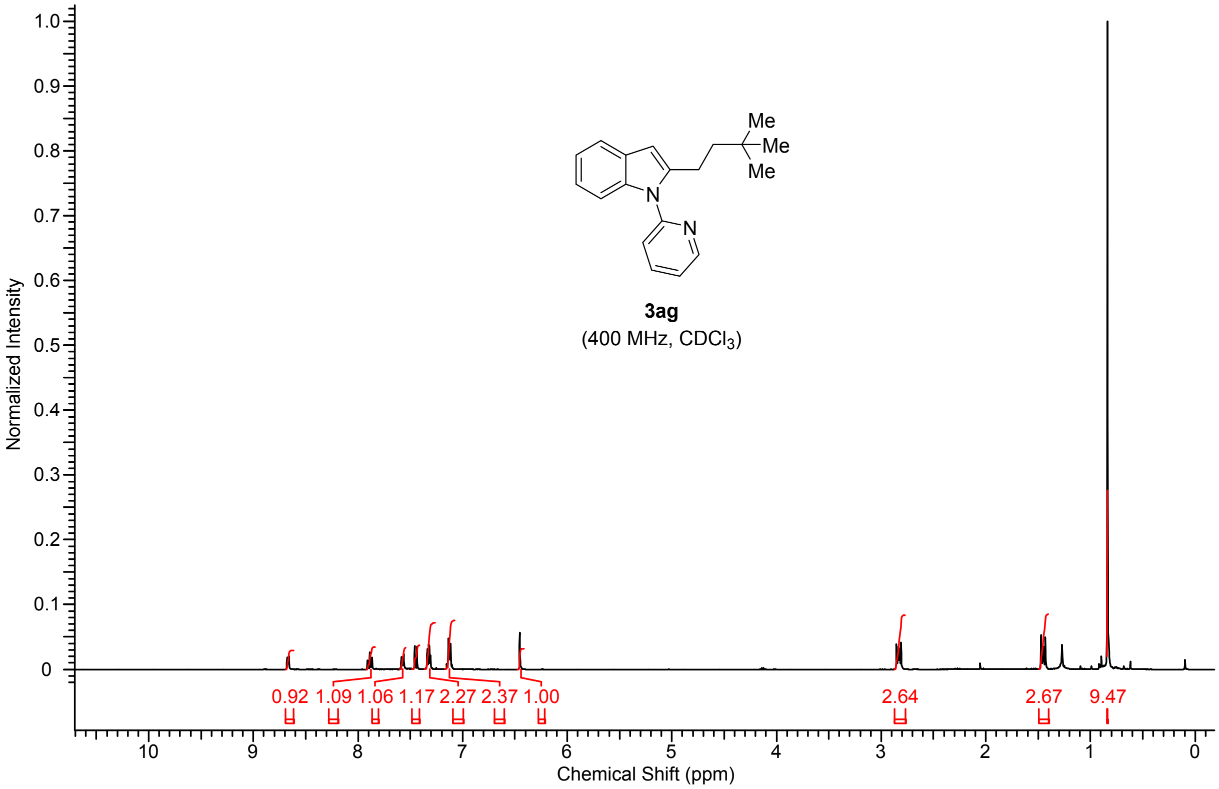
Me
Me
Me
N
N
3ag
(400 MHz, CDCl3)
Normalized Intensity
Chemical Shift (ppm)
0.92 1.09 1.06 1.17 2.27 2.37 1.00
2.64
2.67
9.47

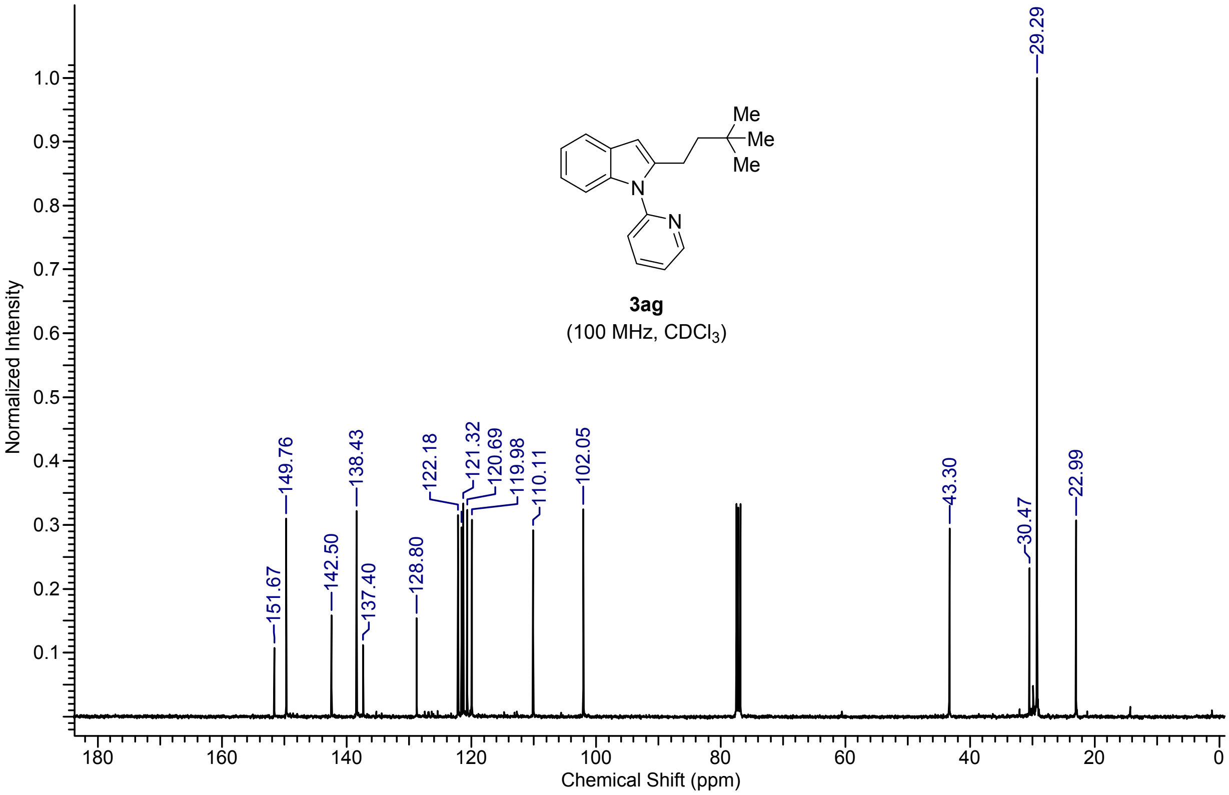
Me
Me
Me
N
N
3ag
(100 MHz, CDCl3)
Normalized Intensity
Chemical Shift (ppm)
151.67
149.76
142.50
138.43
137.40
128.80
122.18
121.32
120.69
119.98
110.11
102.05
43.30
30.47
29.29
22.99

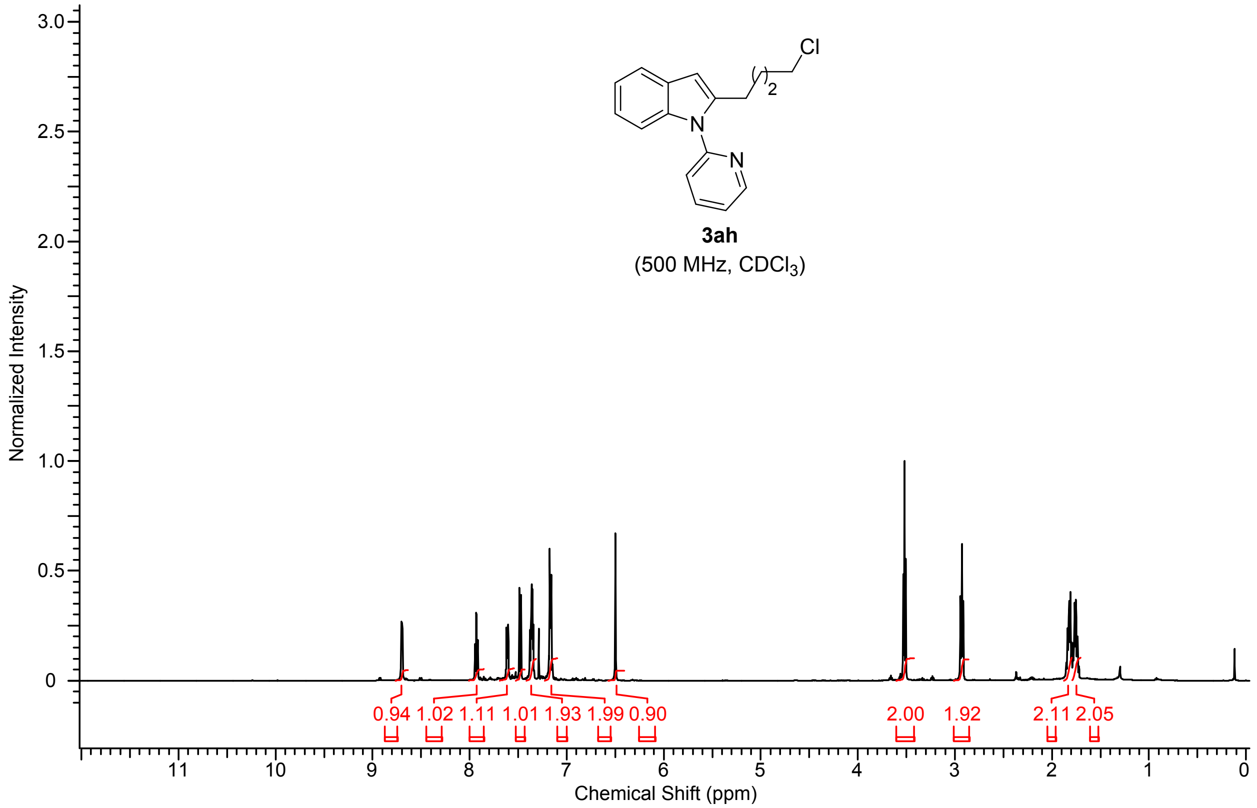
Cl
2
N
N
3ah
(500 MHz, CDCl3)
Normalized Intensity
0.94 1.02 1.11 1.01 1.93 1.99 0.90
2.00 1.92 2.11 2.05
Chemical Shift (ppm)

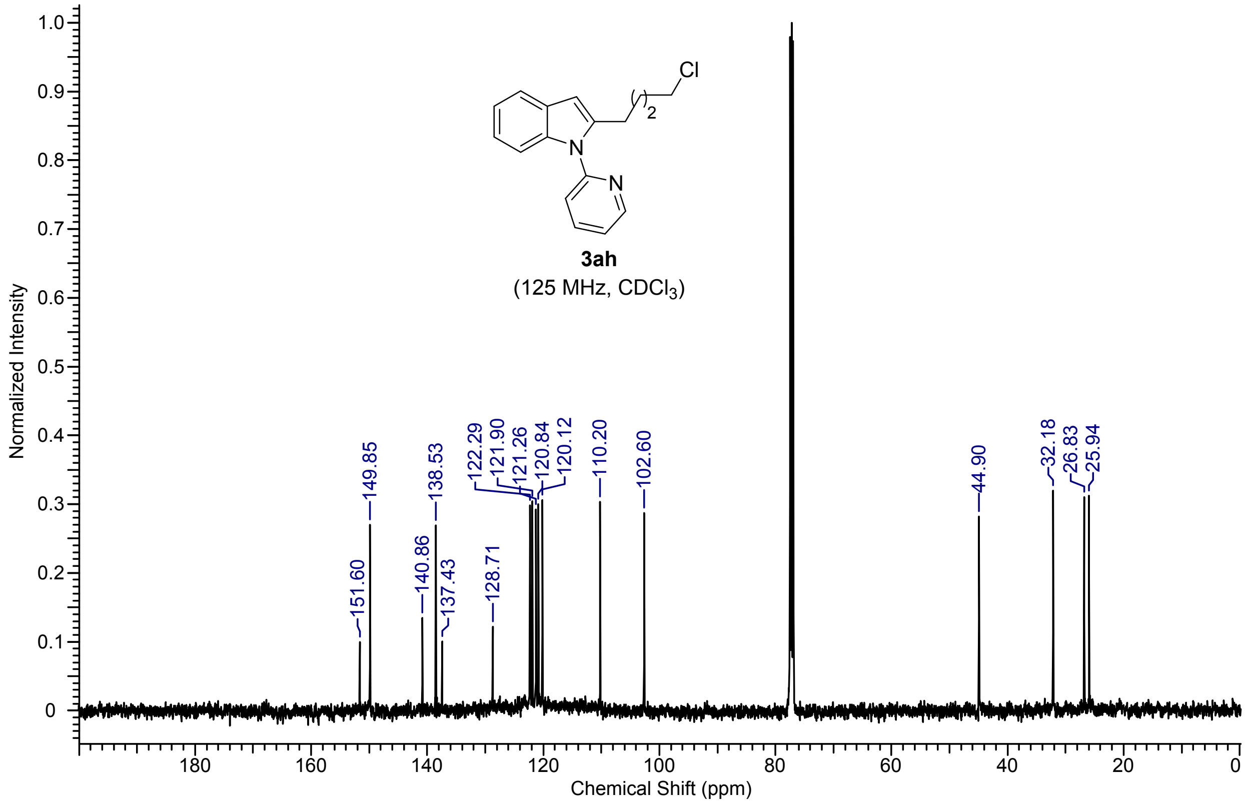
Cl
2
N
N
3ah
(125 MHz, CDCl3)
Normalized Intensity
151.60
149.85
140.86
138.53
137.43
128.71
122.29
121.90
121.26
120.84
120.12
110.20
102.60
44.90
32.18
26.83
25.94
Chemical Shift (ppm)

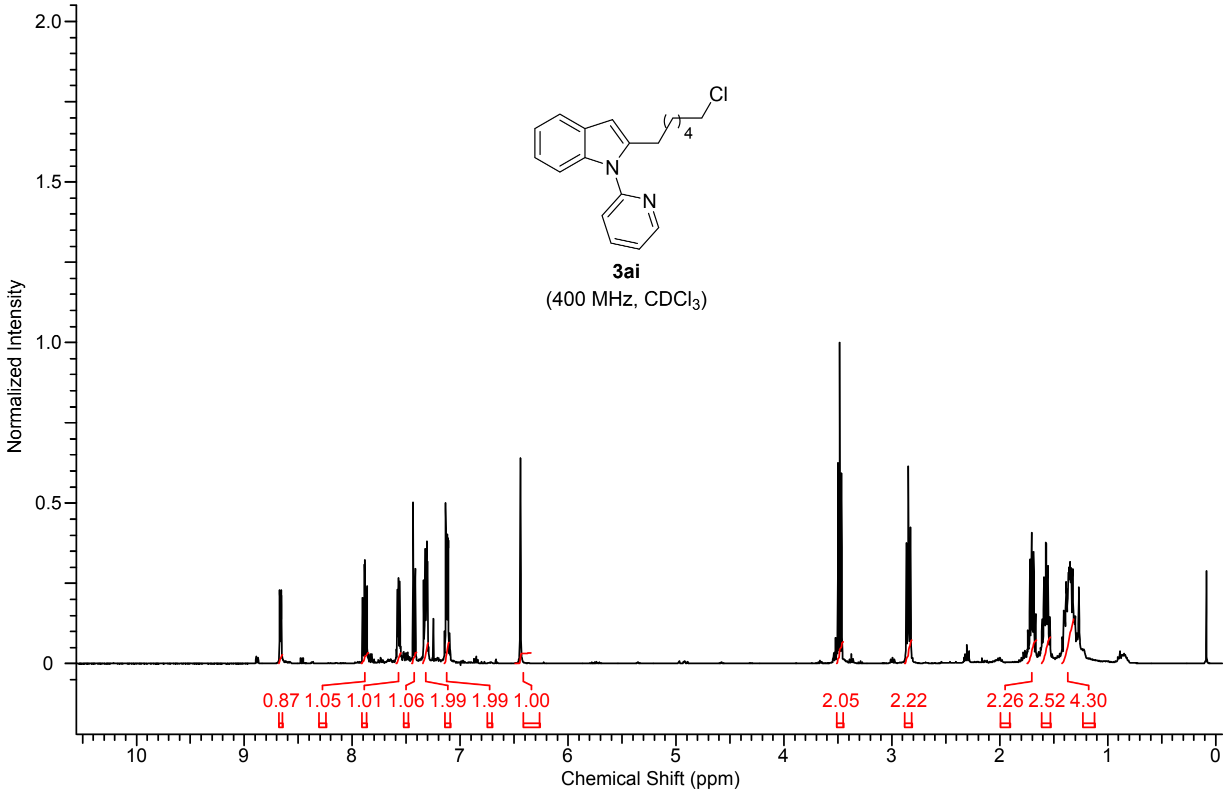
Cl
4
N
N
3ai
(400 MHz, CDCl3)
Normalized Intensity
0.87 1.05 1.01 1.06 1.99 1.99 1.00
2.05
2.22
2.26 2.52 4.30
Chemical Shift (ppm)

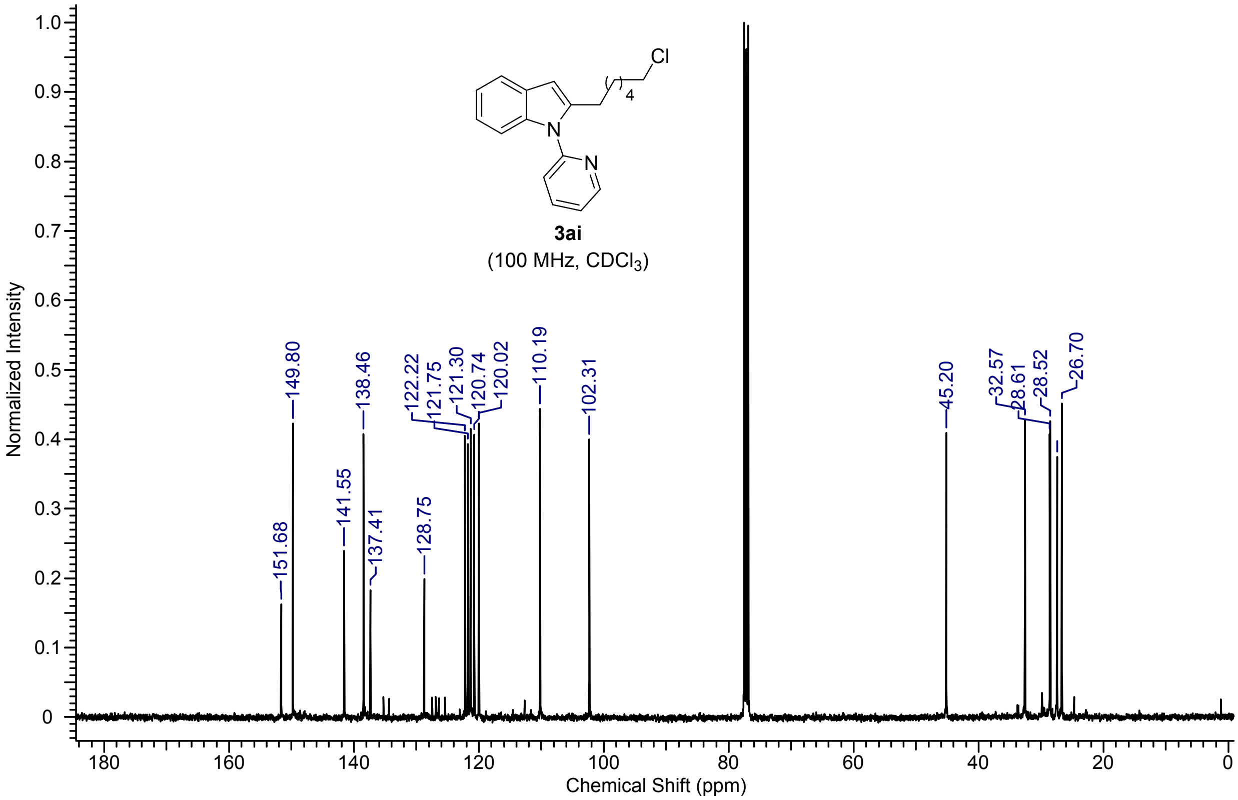
Cl
4
N
N
3ai
(100 MHz, CDCl3)
Normalized Intensity
151.68
149.80
141.55
138.46
137.41
128.75
122.22
121.75
121.30
120.74
120.02
110.19
102.31
45.20
32.57
28.61
28.52
26.70
Chemical Shift (ppm)

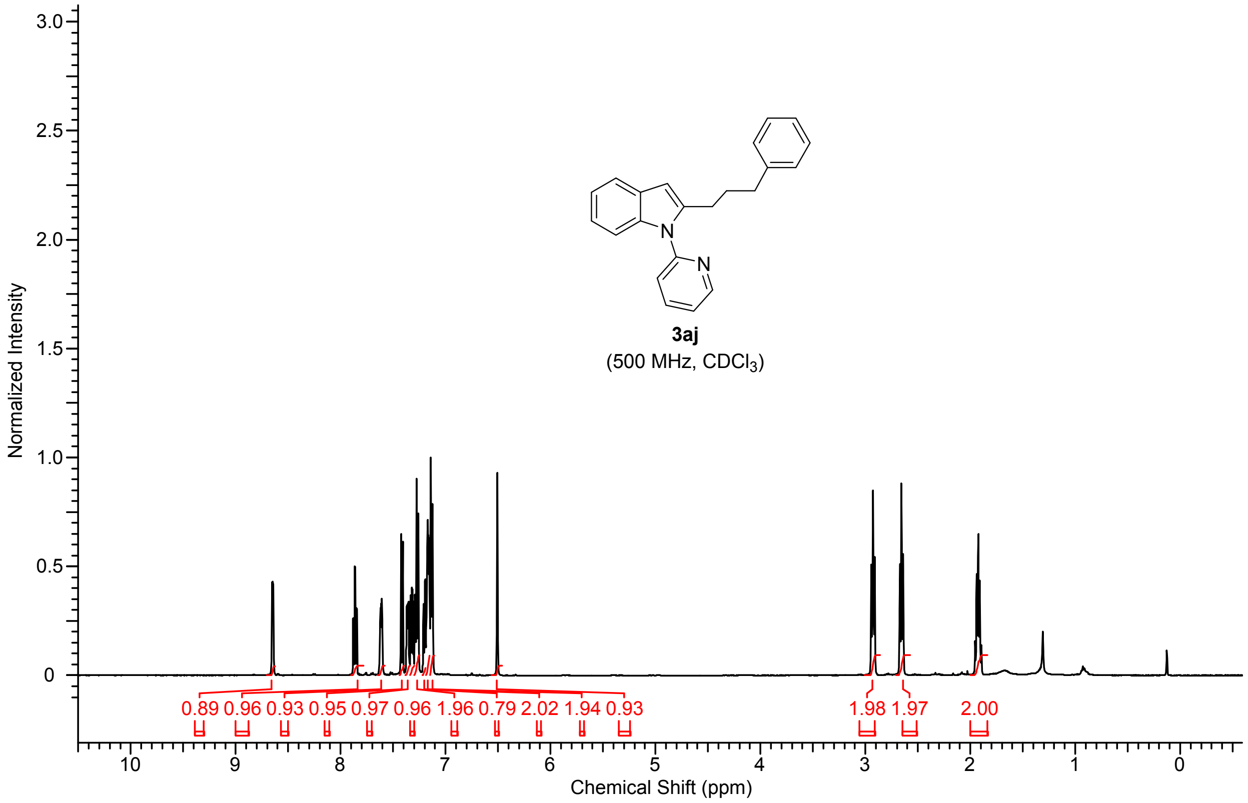
N
N
3aj
(500 MHz, CDCl3)
Normalized Intensity
Chemical Shift (ppm)
0.89 0.96 0.93 0.95 0.97 0.96 1.96 0.79 2.02 1.94 0.93
1.98 1.97
2.00

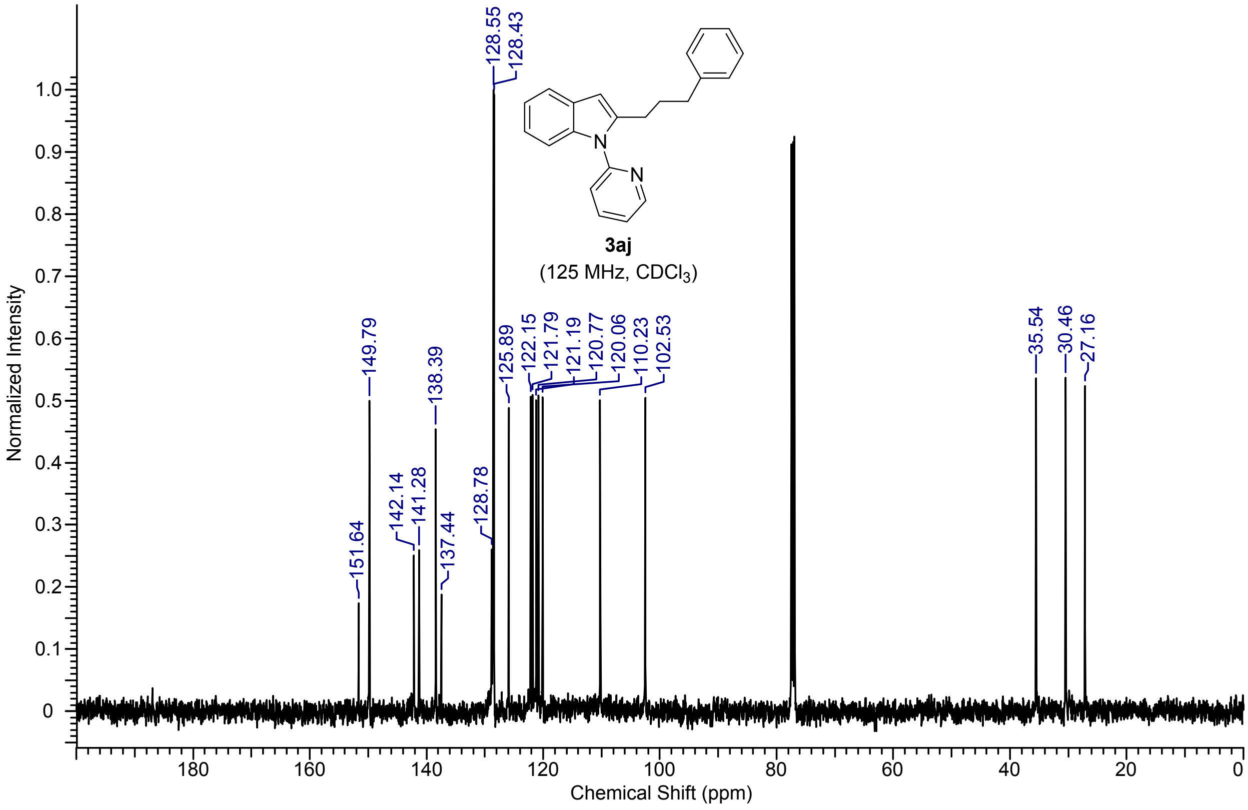
N
N
3aj
(125 MHz, CDCl3)
Normalized Intensity
Chemical Shift (ppm)
151.64
149.79
142.14
141.28
138.39
137.44
128.78
128.55
128.43
125.89
122.15
121.79
121.19
120.77
120.06
110.23
102.53
35.54
30.46
27.16

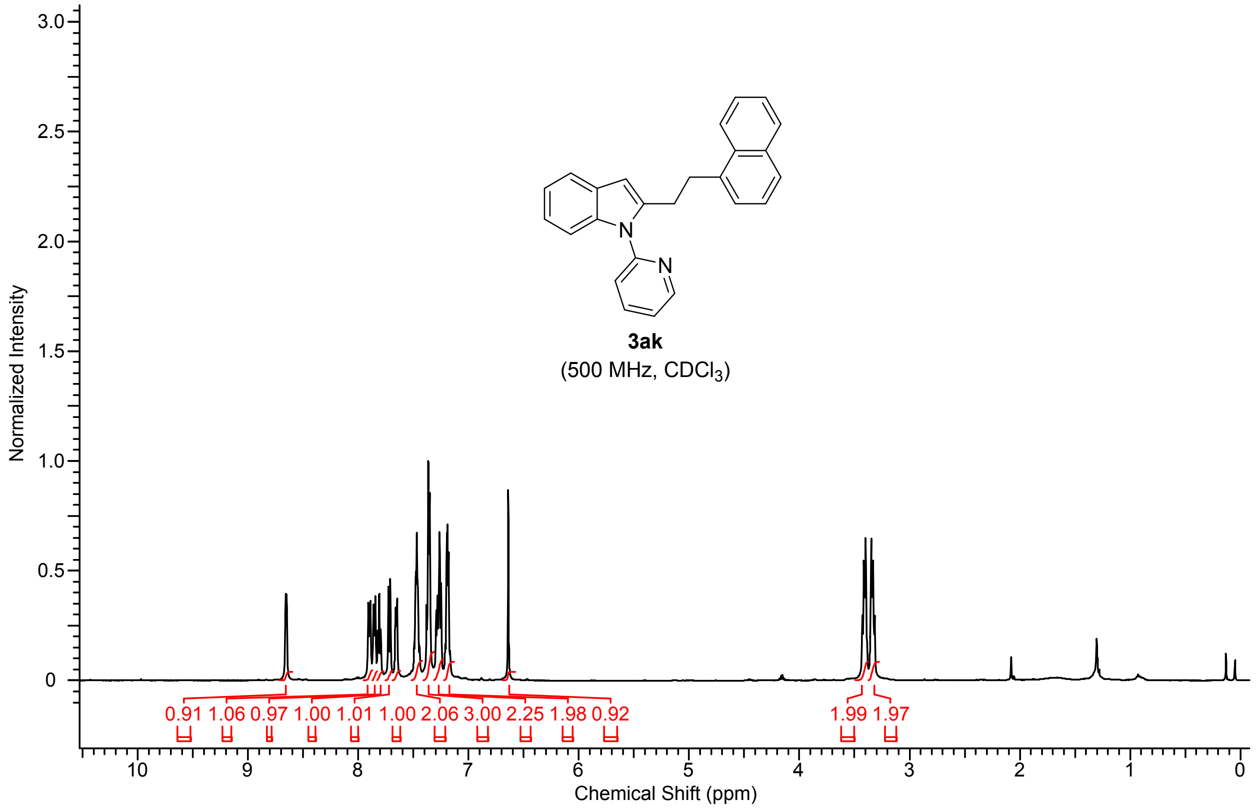

3ak
(500 MHz, CDCl3)
Normalized Intensity
Chemical Shift (ppm)
0.91 1.06 0.97 1.00 1.01 1.00 2.06 3.00 2.25 1.98 0.92
1.99 1.97

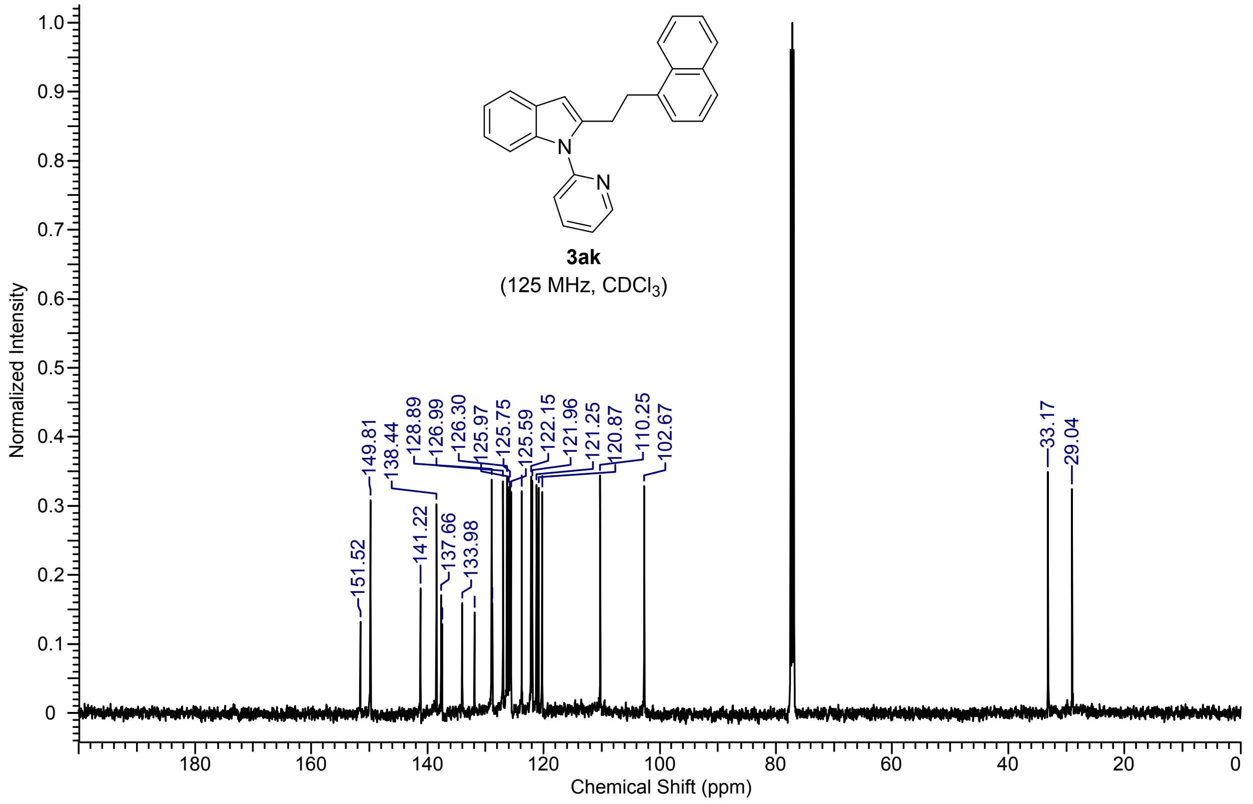

3ak
(125 MHz, CDCl3)
Normalized Intensity
Chemical Shift (ppm)
151.52
149.81
141.22
138.44
137.66
133.98
128.89
126.99
126.30
125.97
125.75
125.59
122.15
121.96
121.25
120.87
110.25
102.67
33.17
29.04

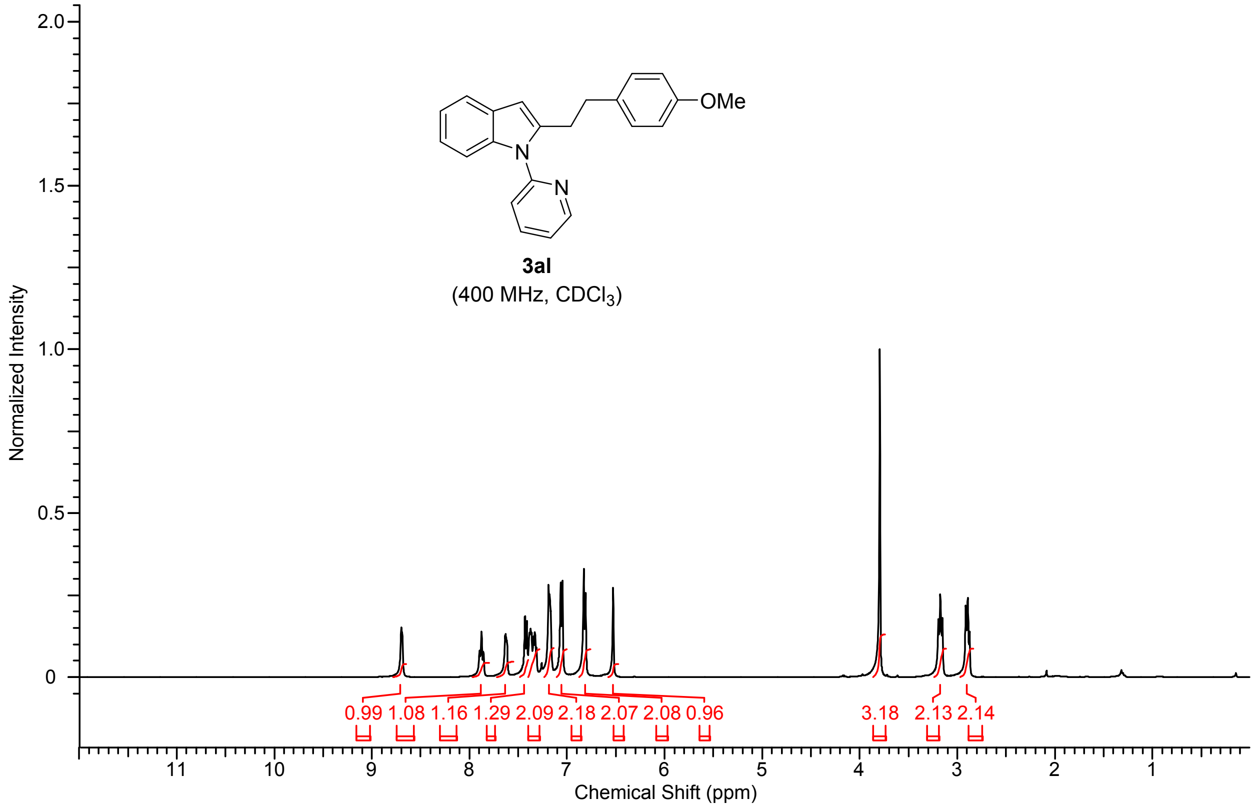

OMe
N
N
3al
(400 MHz, CDCl3)
Normalized Intensity
2.0
1.5
1.0
0.5
0
0.99 1.08 1.16 1.29 2.09 2.18 2.07 2.08 0.96
3.18 2.13 2.14
11 10 9 8 7 6 5 4 3 2 1
Chemical Shift (ppm)

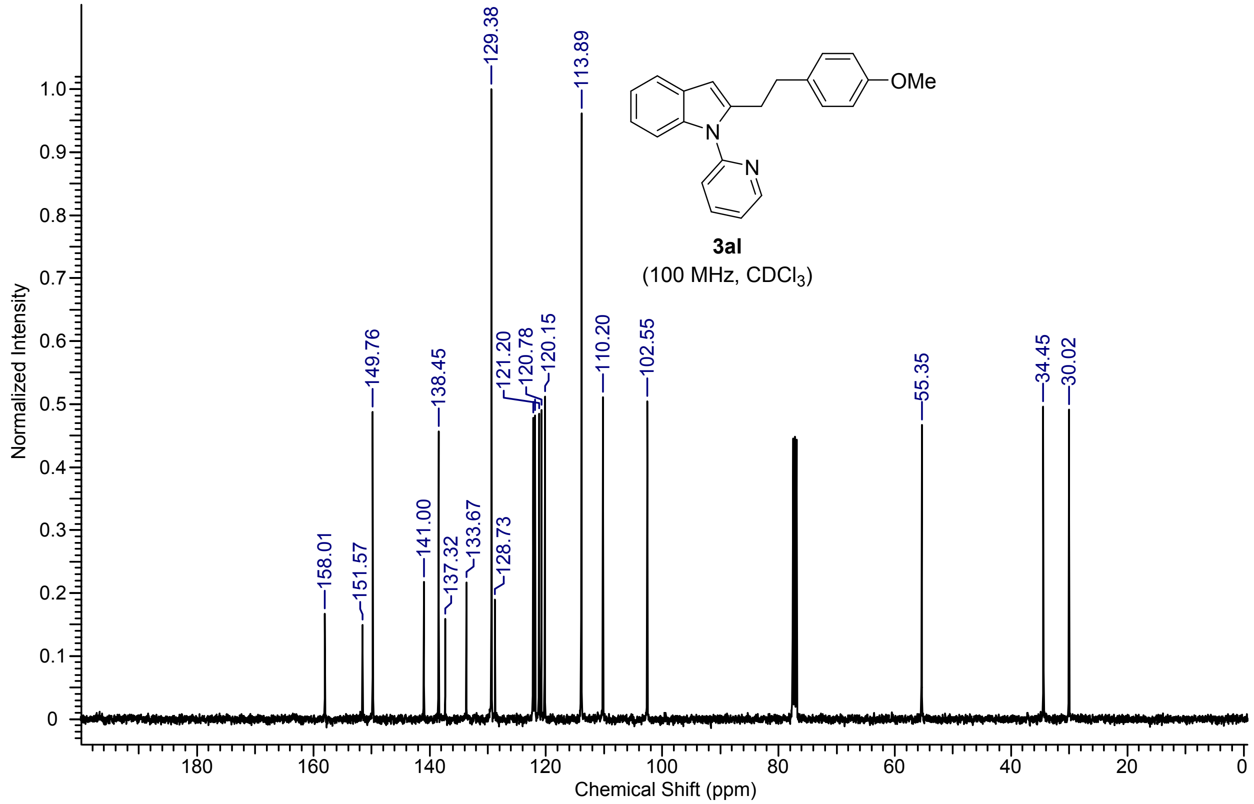

OMe
N
N
3al
(100 MHz, CDCl3)
Normalized Intensity
1.0 0.9 0.8 0.7 0.6 0.5 0.4 0.3 0.2 0.1 0
158.01
151.57
149.76
141.00
138.45
137.32
133.67
129.38
128.73
121.20
120.78
120.15
113.89
110.20
102.55
55.35
34.45
30.02
180 160 140 120 100 80 60 40 20 0
Chemical Shift (ppm)

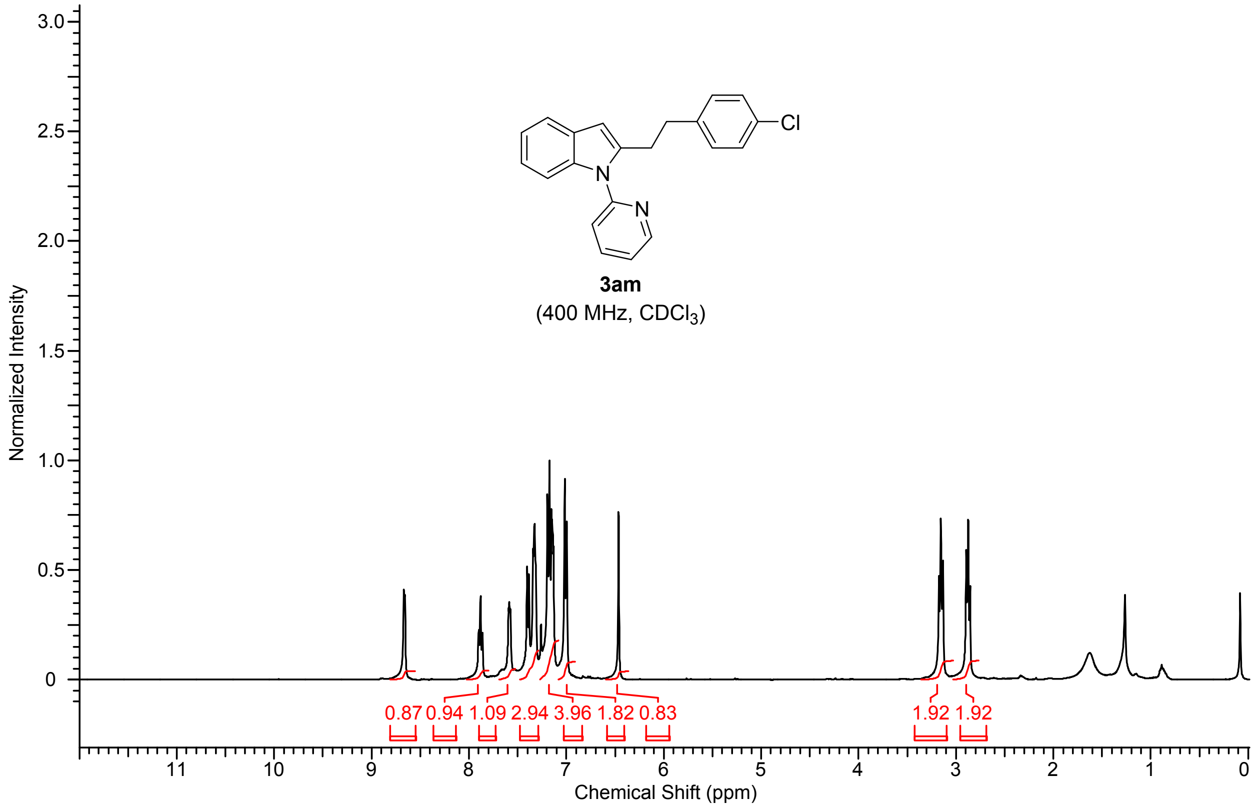
Cl
N
N
3am
(400 MHz, $CDCl_3$)
Normalized Intensity
Chemical Shift (ppm)
0.87 0.94 1.09 2.94 3.96 1.82 0.83
1.92 1.92

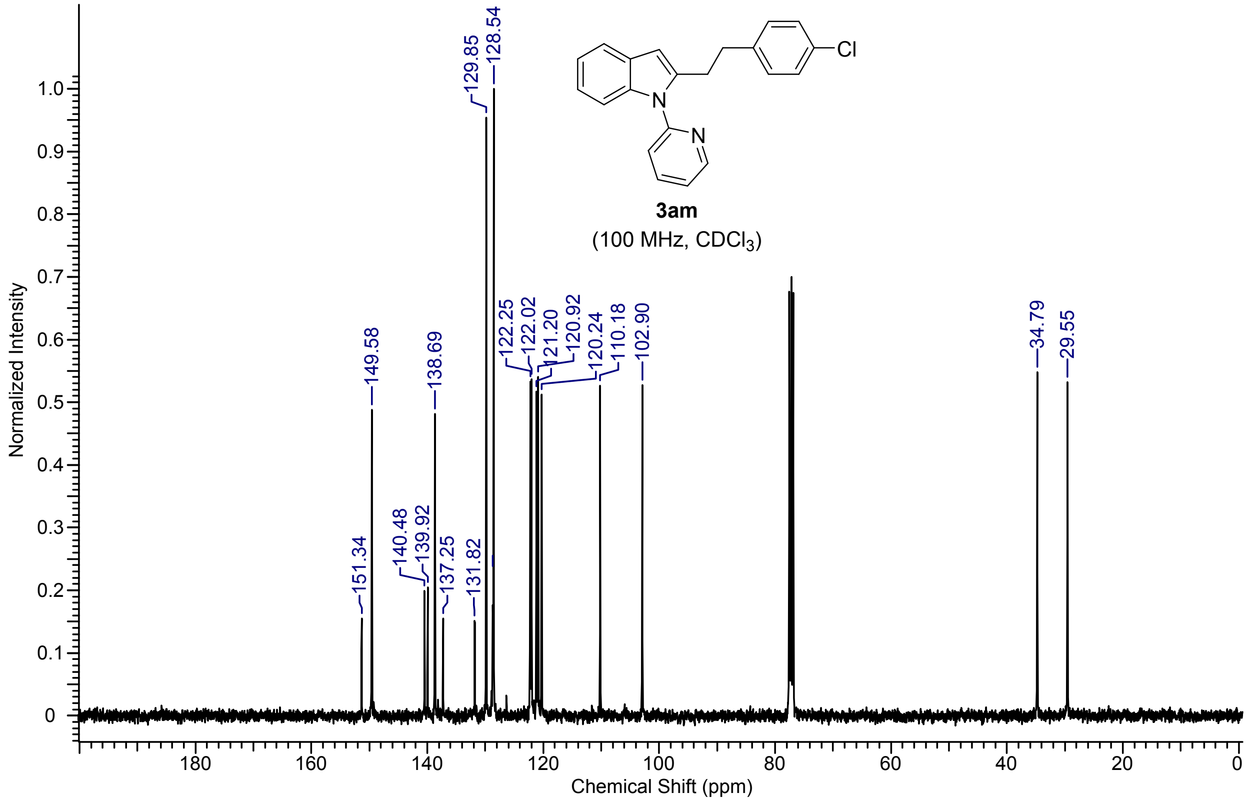
Cl
N
N
3am
(100 MHz, $CDCl_3$)
Normalized Intensity
Chemical Shift (ppm)
151.34
149.58
140.48
139.92
138.69
137.25
131.82
129.85
128.54
122.25
122.02
121.20
120.92
120.24
110.18
102.90
34.79
29.55

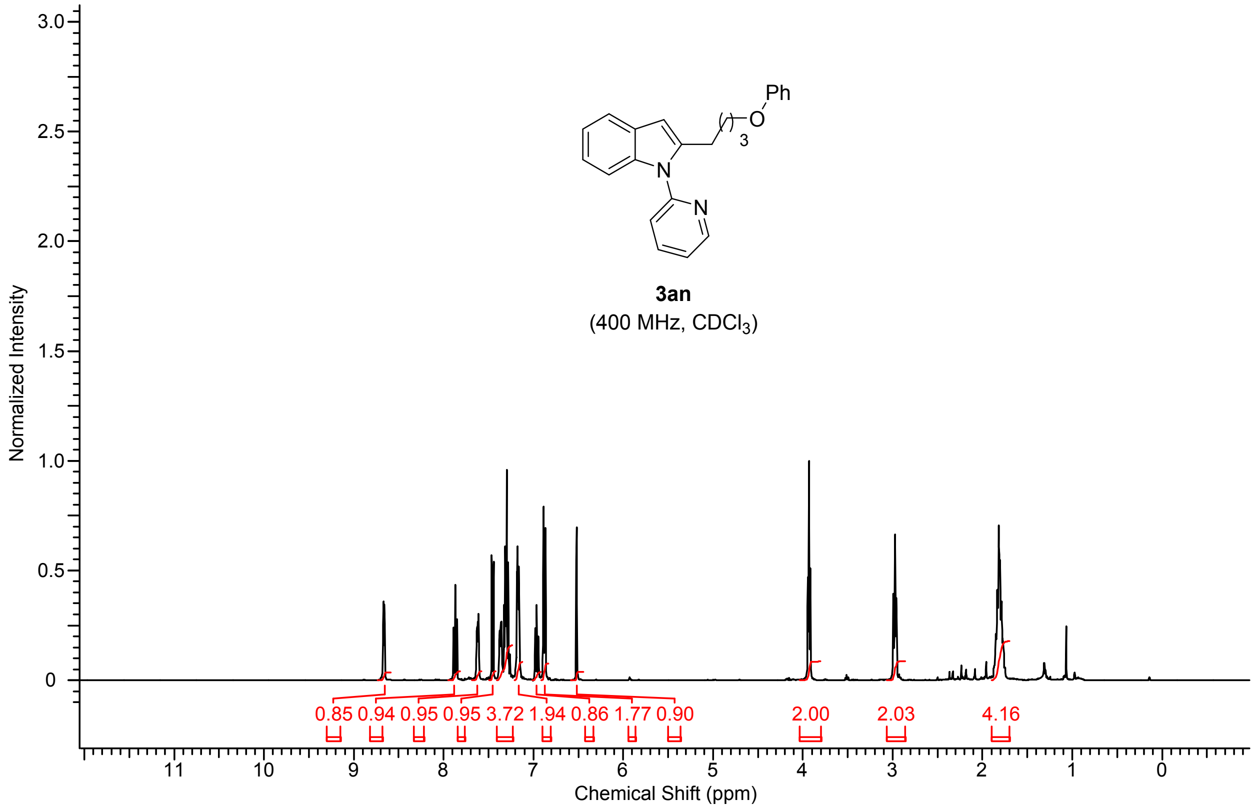

Ph
O
3
N
N
3an
(400 MHz, $CDCl_3$)
Normalized Intensity
0.85 0.94 0.95 0.95 3.72 1.94 0.86 1.77 0.90
2.00
2.03
4.16
Chemical Shift (ppm)

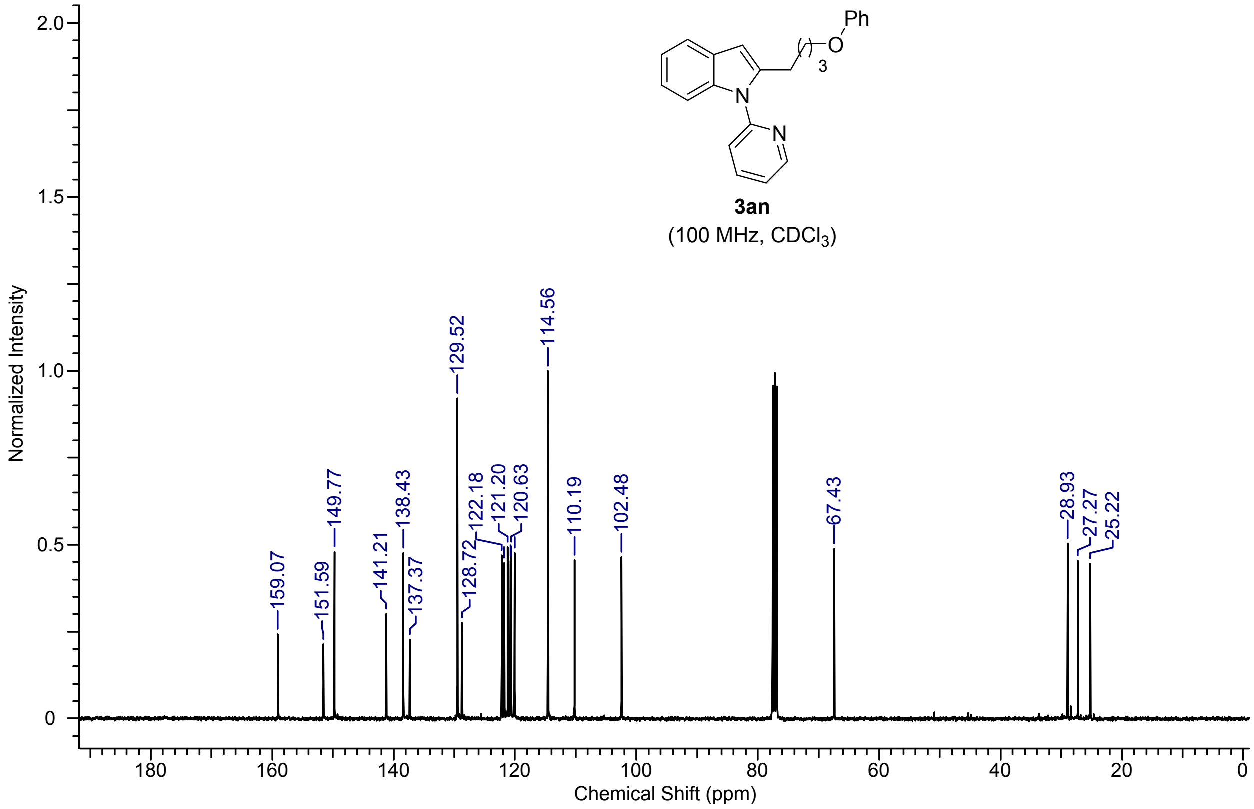

Ph
O
3
N
N
3an
(100 MHz, $CDCl_3$)
Normalized Intensity
159.07
151.59
149.77
141.21
138.43
137.37
129.52
128.72
122.18
121.20
120.63
114.56
110.19
102.48
67.43
28.93
27.27
25.22
Chemical Shift (ppm)

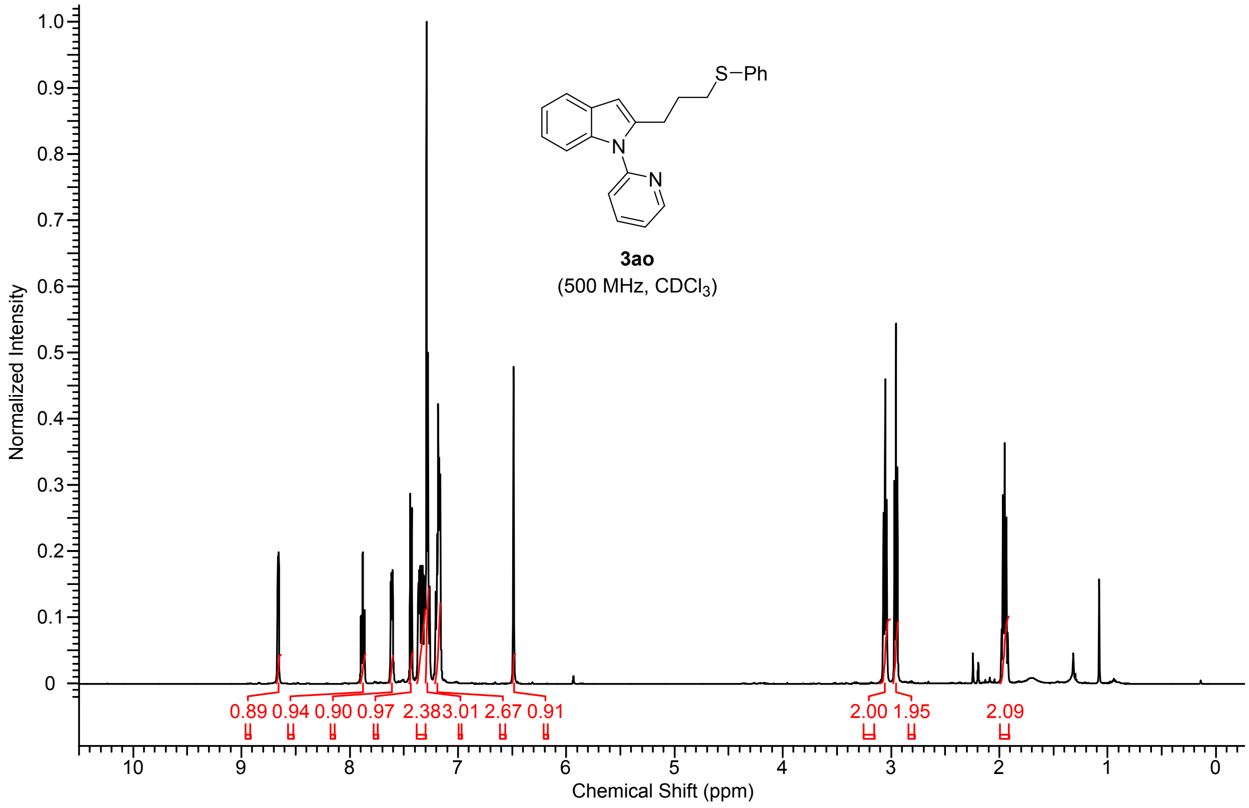
S−Ph
N
N
3ao
(500 MHz, CDCl3)
Normalized Intensity
Chemical Shift (ppm)
0.89 0.94 0.90 0.97 2.38 3.01 2.67 0.91
2.00 1.95
2.09

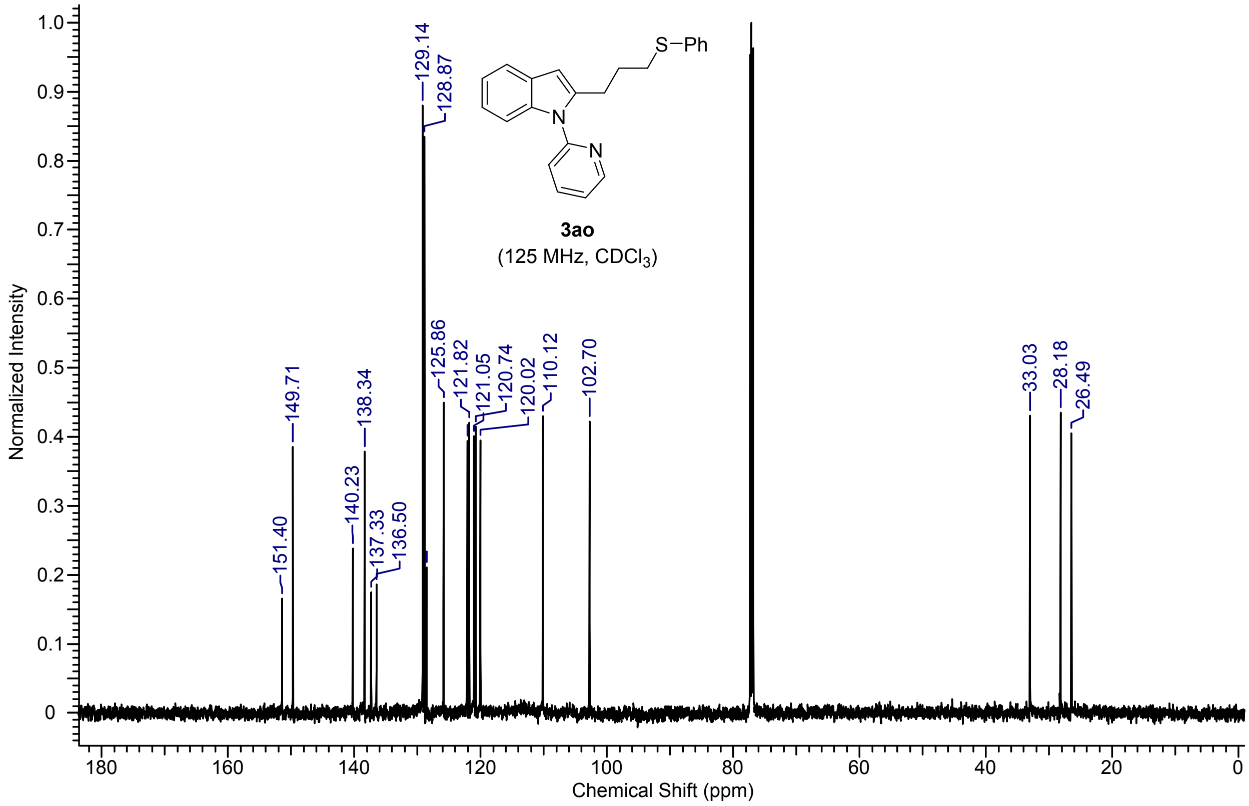
S−Ph
N
N
3ao
(125 MHz, CDCl3)
Normalized Intensity
Chemical Shift (ppm)
151.40
149.71
140.23
138.34
137.33
136.50
129.14
128.87
125.86
121.82
121.05
120.74
120.02
110.12
102.70
33.03
28.18
26.49

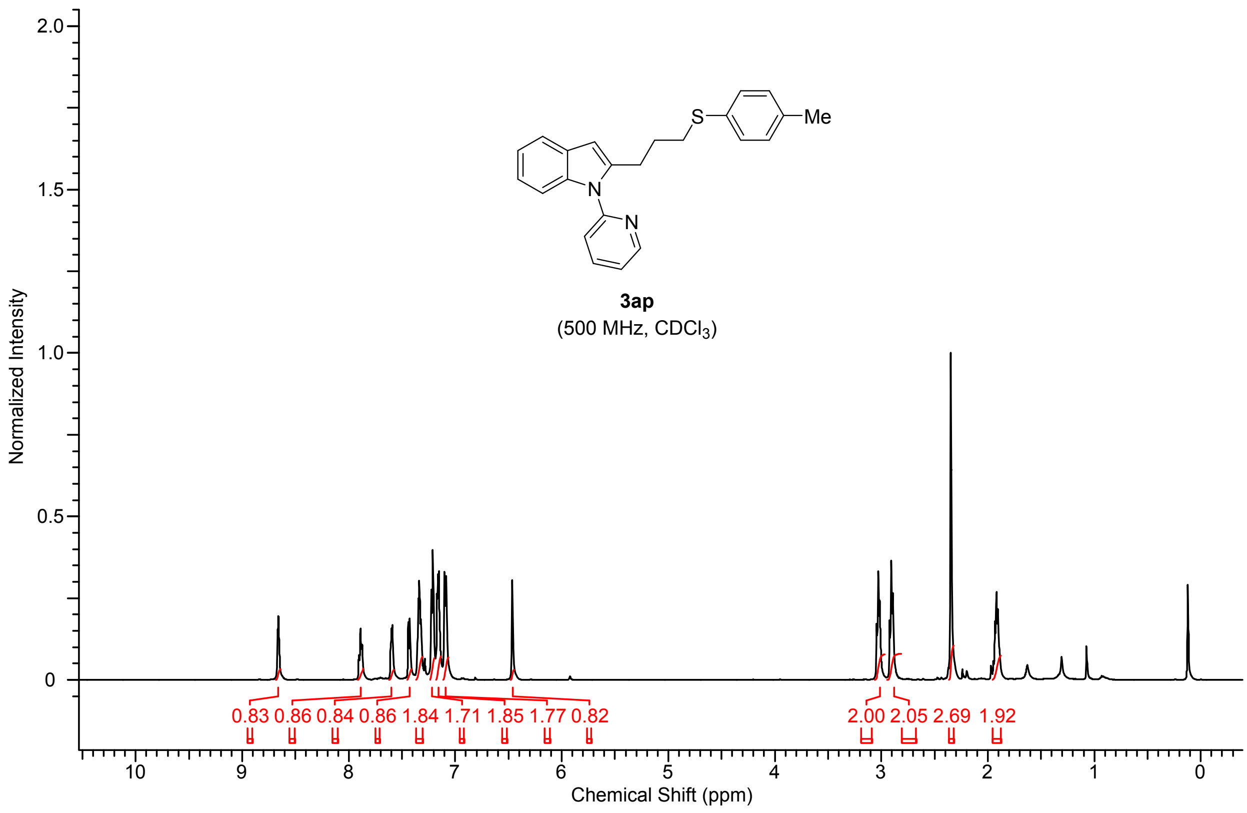

S
Me
N
N
3ap
(500 MHz, CDCl3)
Normalized Intensity
Chemical Shift (ppm)
0.83 0.86 0.84 0.86 1.84 1.71 1.85 1.77 0.82
2.00 2.05 2.69 1.92

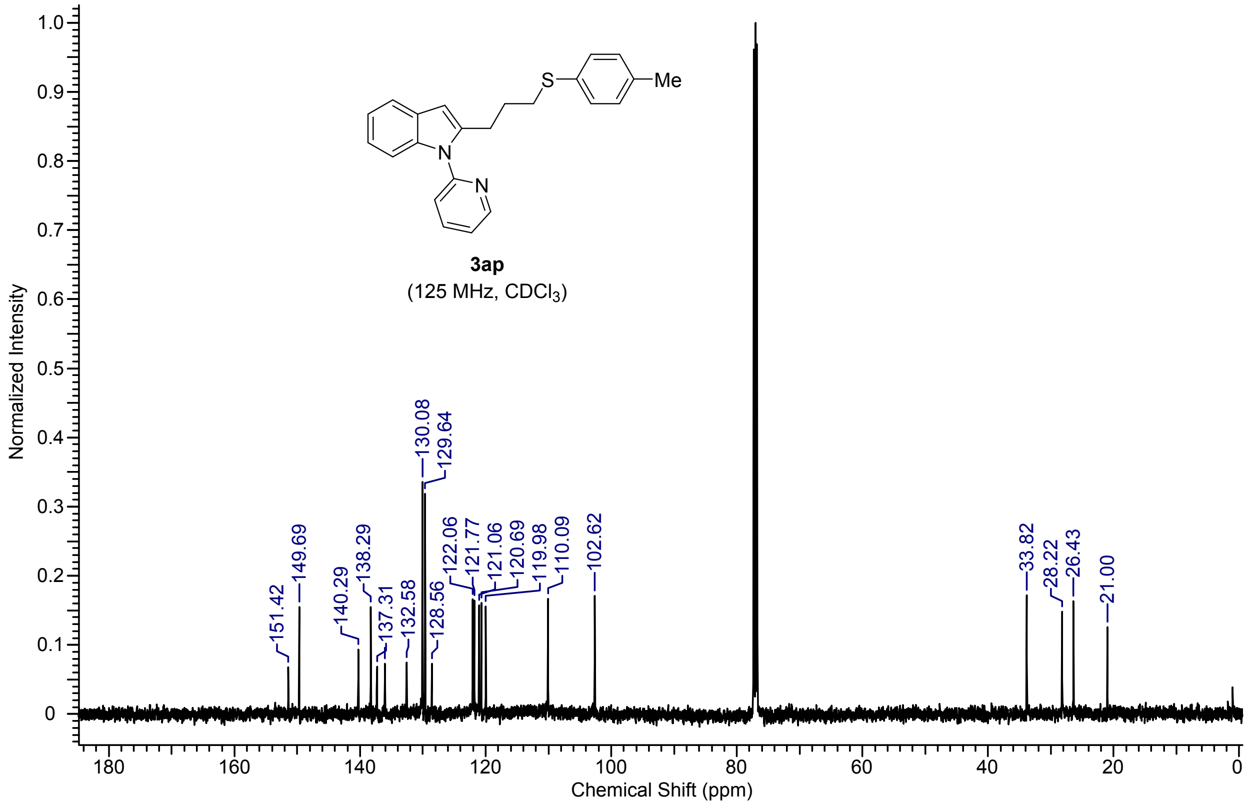

S
Me
N
N
3ap
(125 MHz, CDCl3)
Normalized Intensity
Chemical Shift (ppm)
151.42
149.69
140.29
138.29
137.31
132.58
128.56
130.08
129.64
122.06
121.77
121.06
120.69
119.98
110.09
102.62
33.82
28.22
26.43
21.00

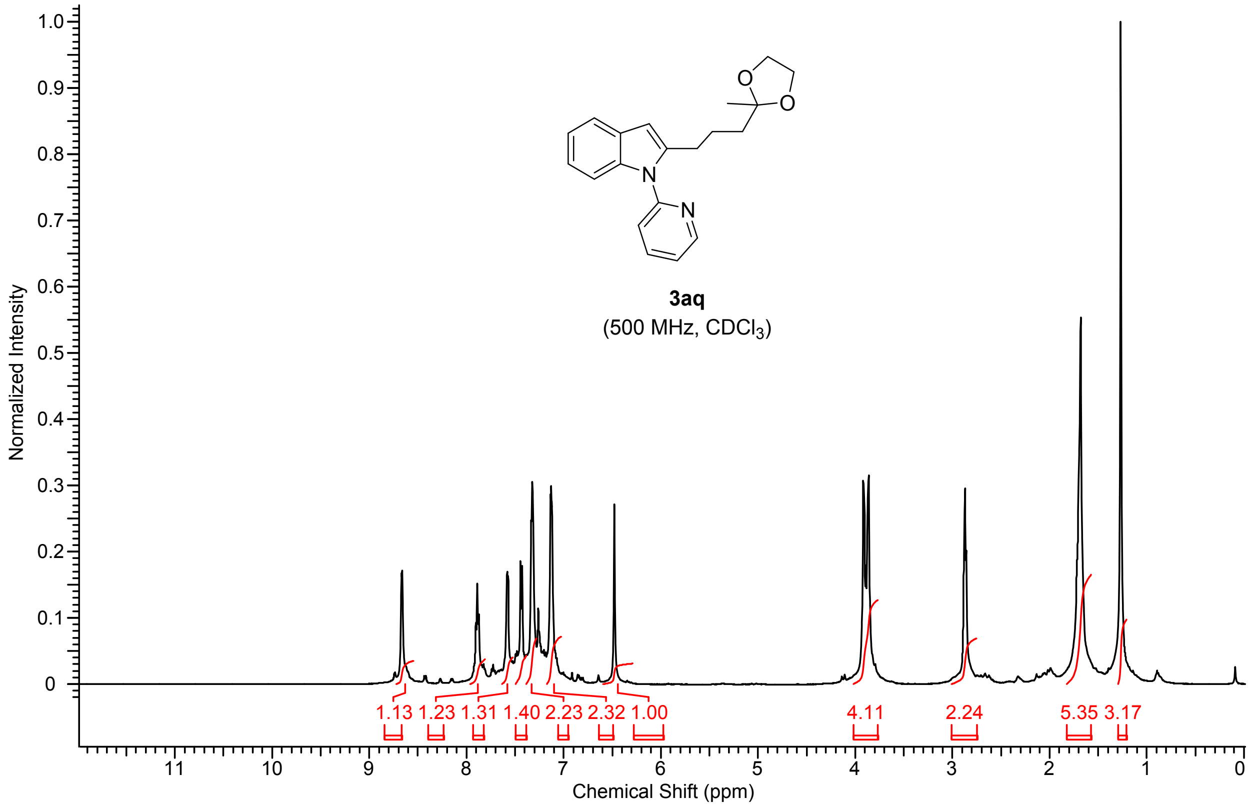

3aq
(500 MHz, CDCl3)
1.13 1.23 1.31 1.40 2.23 2.32 1.00 4.11 2.24 5.35 3.17
Normalized Intensity
Chemical Shift (ppm)

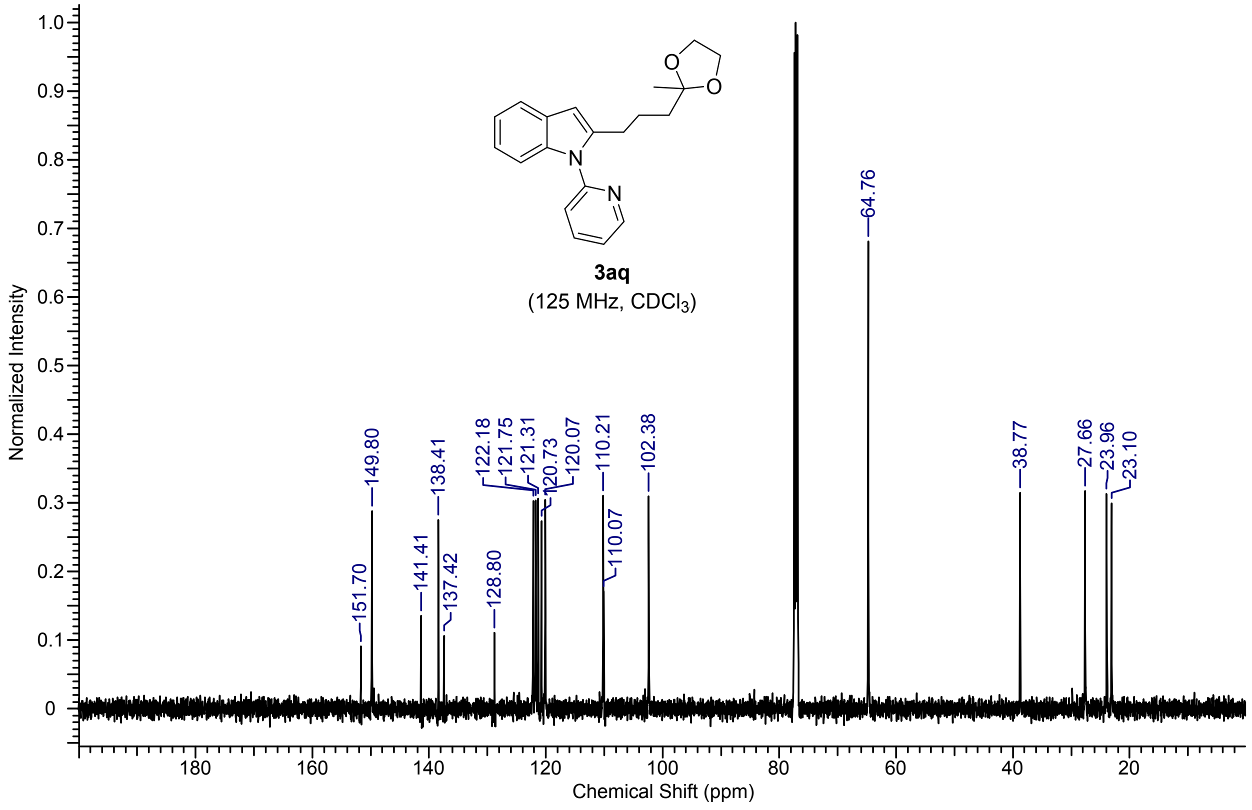

3aq
(125 MHz, CDCl3)
151.70 149.80 141.41 138.41 137.42 128.80 122.18 121.75 121.31 120.73 120.07 110.21 110.07 102.38 64.76 38.77 27.66 23.96 23.10
Normalized Intensity
Chemical Shift (ppm)

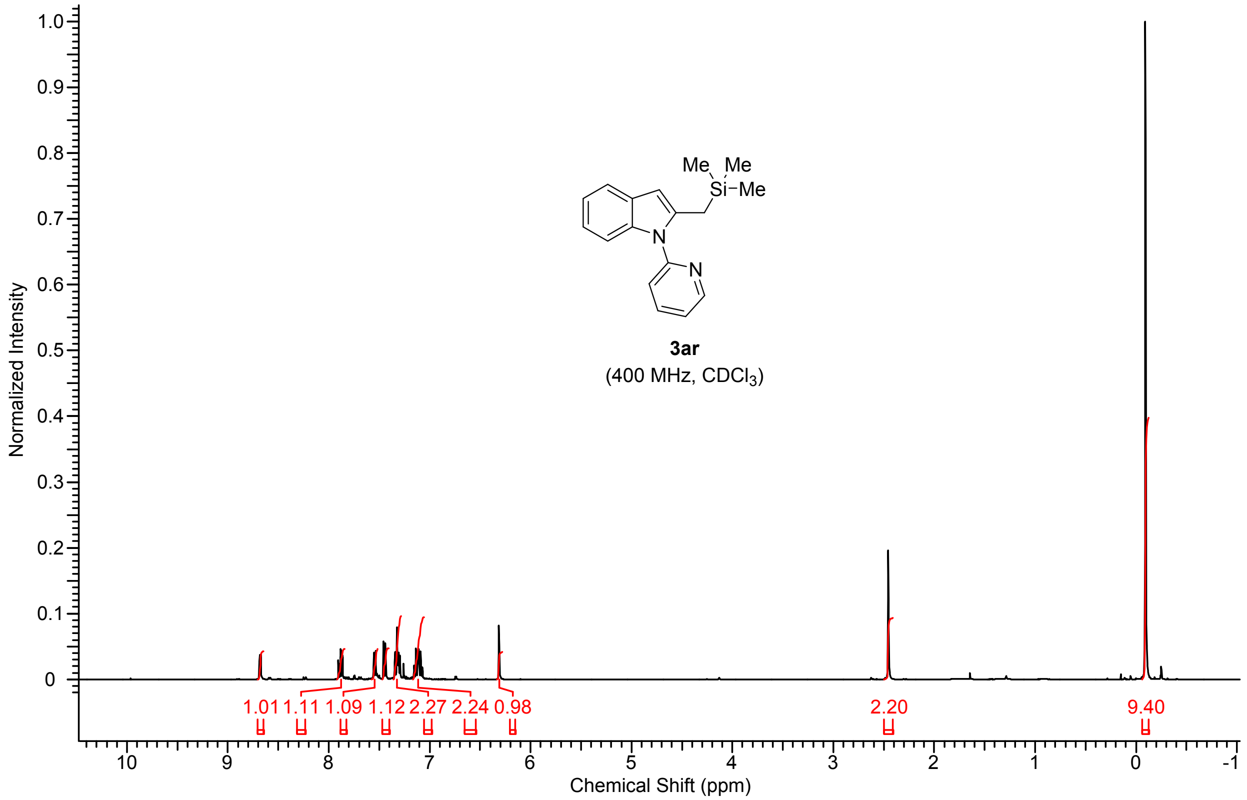
Me
Me
Si
Me
N
N
3ar
(400 MHz, CDCl3)
Normalized Intensity
1.01 1.11 1.09 1.12 2.27 2.24 0.98
2.20
9.40
Chemical Shift (ppm)

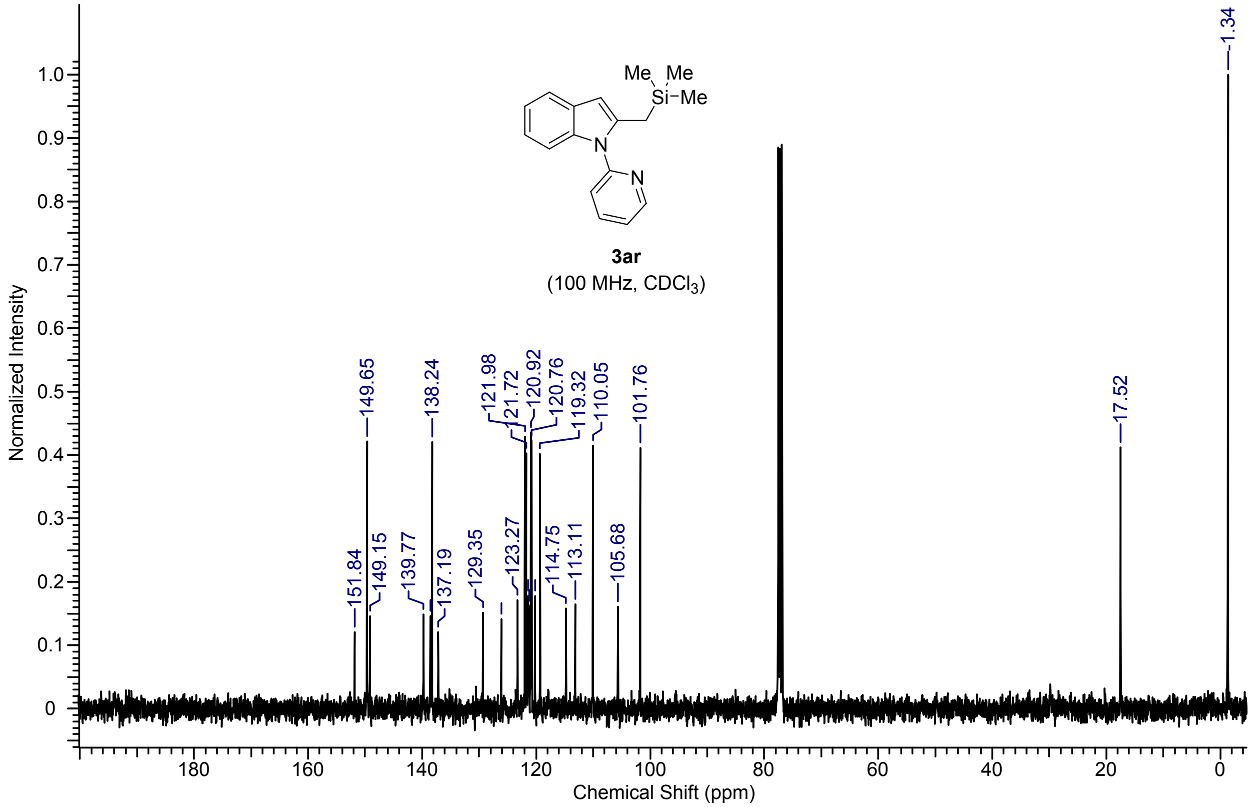
Me
Me
Si
Me
N
N
3ar
(100 MHz, CDCl3)
Normalized Intensity
151.84
149.65
149.15
139.77
138.24
137.19
129.35
123.27
121.98
121.72
120.92
120.76
119.32
114.75
113.11
110.05
105.68
101.76
17.52
-1.34
Chemical Shift (ppm)

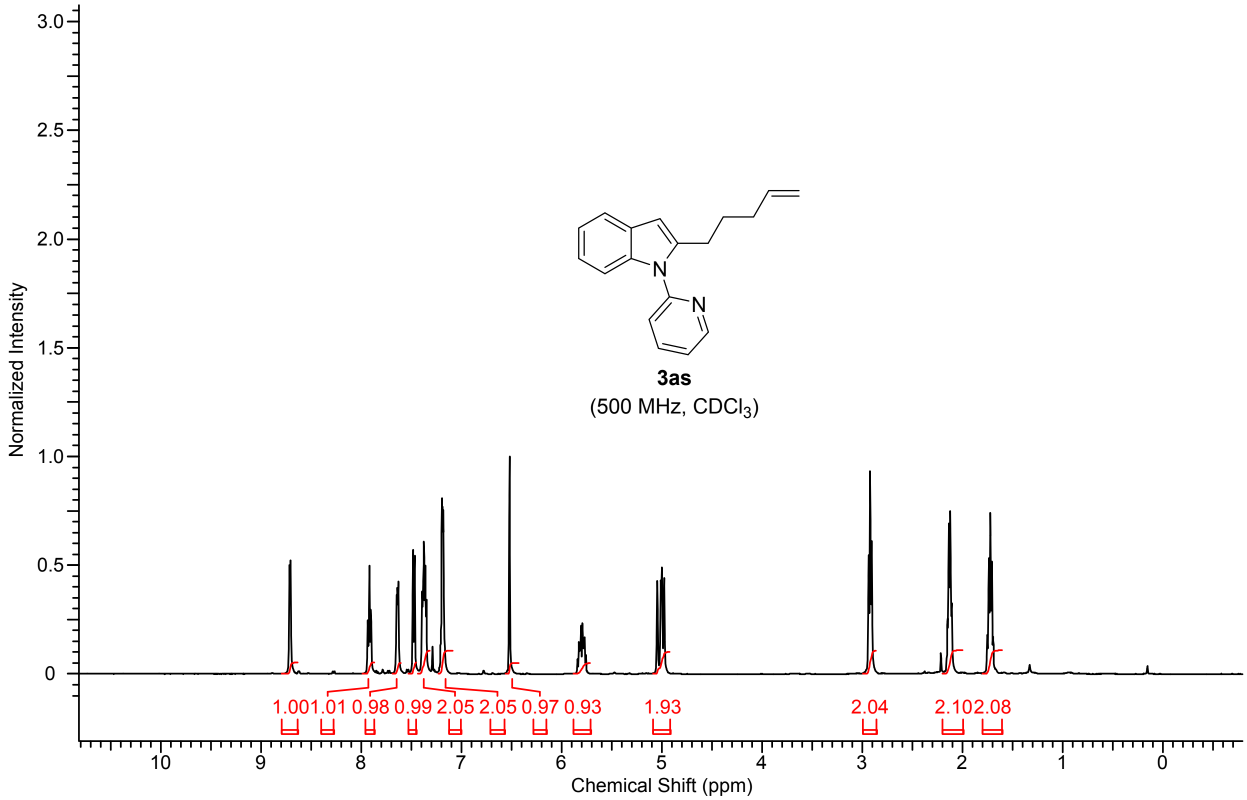

N
3as
(500 MHz, CDCl3)
Normalized Intensity
1.00
1.01
0.98
0.99
2.05
2.05
0.97
0.93
1.93
2.04
2.10
2.08
Chemical Shift (ppm)

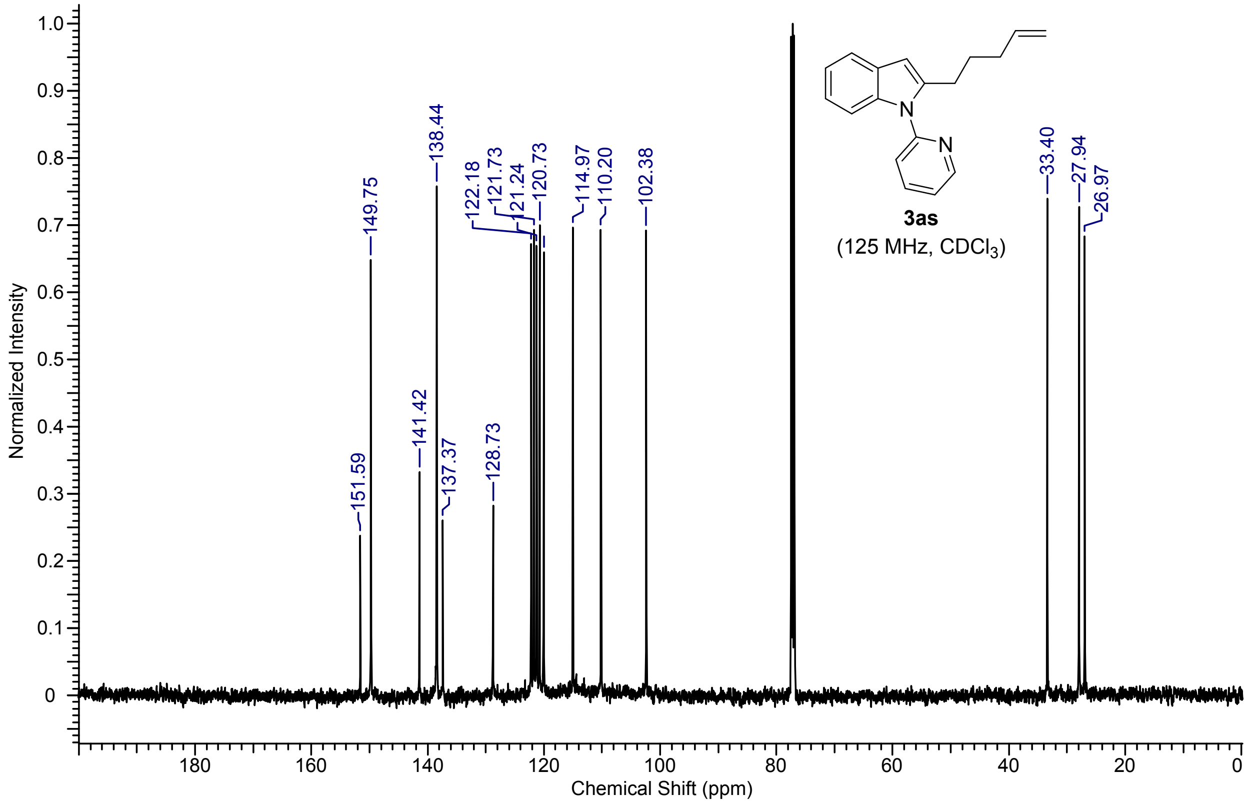

N
3as
(125 MHz, CDCl3)
Normalized Intensity
151.59
149.75
141.42
138.44
137.37
128.73
122.18
121.73
121.24
120.73
114.97
110.20
102.38
33.40
27.94
26.97
Chemical Shift (ppm)

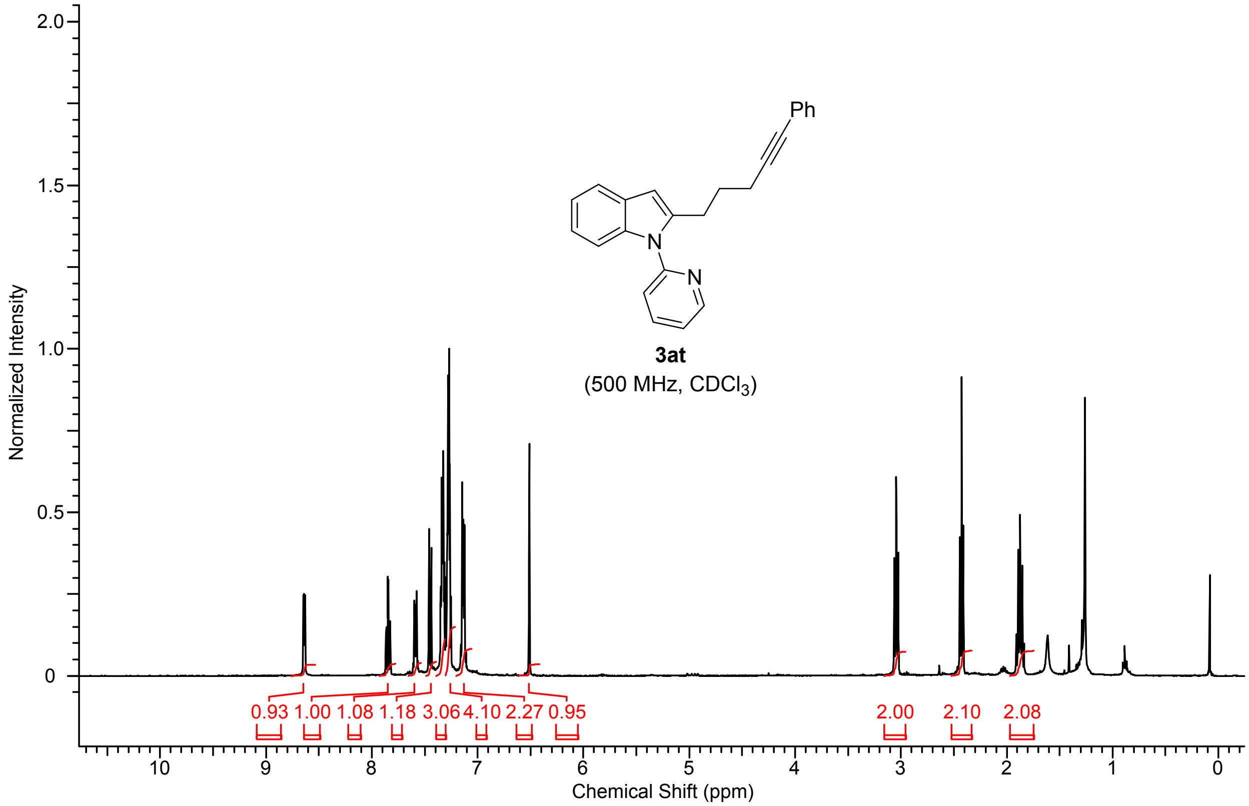
Ph
N
N
3at
(500 MHz, CDCl3)
Normalized Intensity
Chemical Shift (ppm)
0.93 1.00 1.08 1.18 3.06 4.10 2.27 0.95
2.00 2.10 2.08

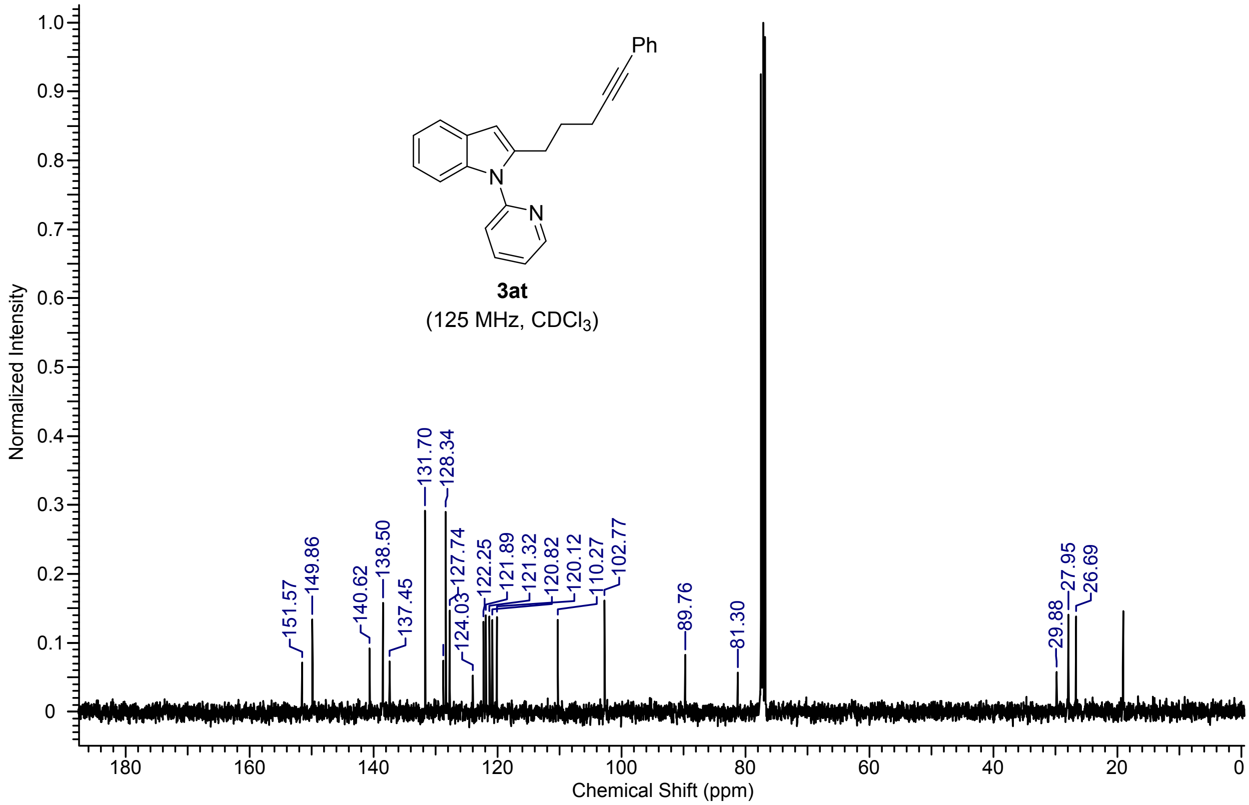
Ph
N
N
3at
(125 MHz, CDCl3)
Normalized Intensity
Chemical Shift (ppm)
151.57 149.86 140.62 138.50 137.45 131.70 128.34 127.74 124.03 122.25 121.89 121.32 120.82 120.12 110.27 102.77 89.76 81.30 29.88 27.95 26.69

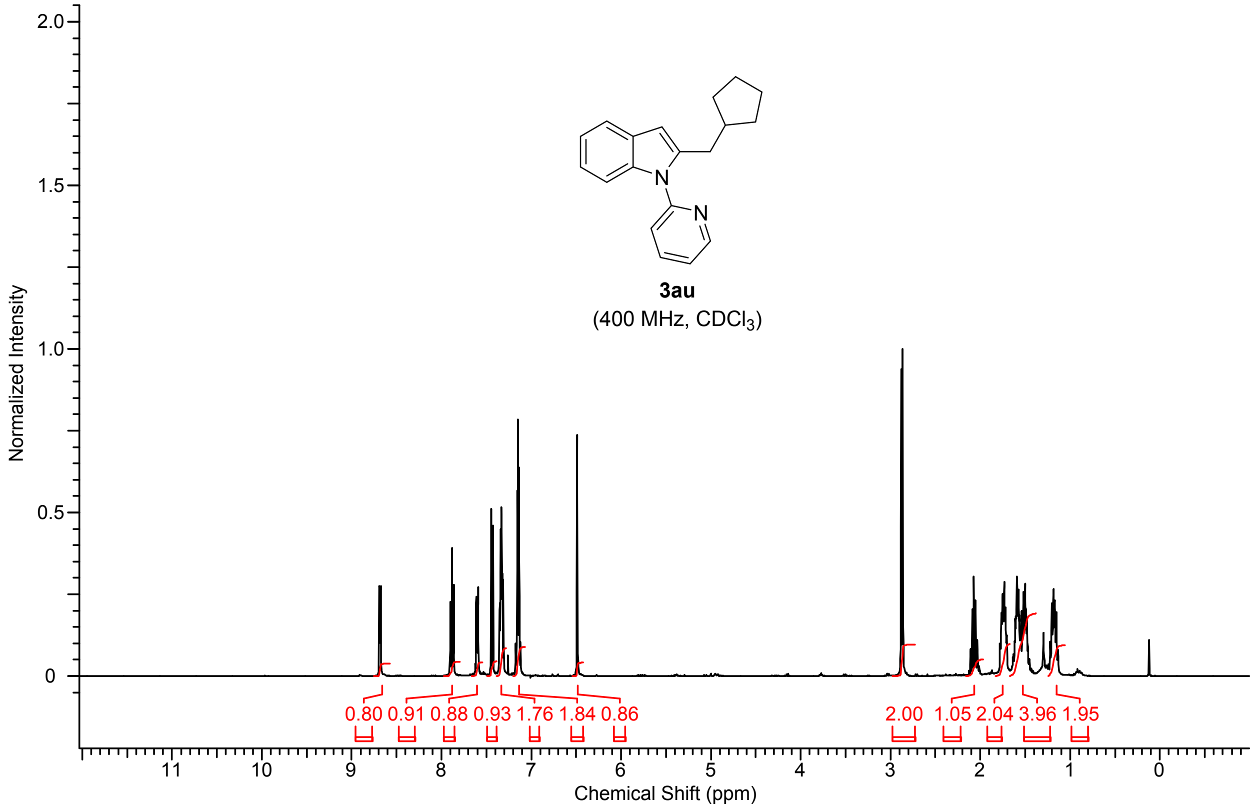

N
3au
(400 MHz, $CDCl_3$)
Normalized Intensity
0.80 0.91 0.88 0.93 1.76 1.84 0.86
2.00 1.05 2.04 3.96 1.95
Chemical Shift (ppm)

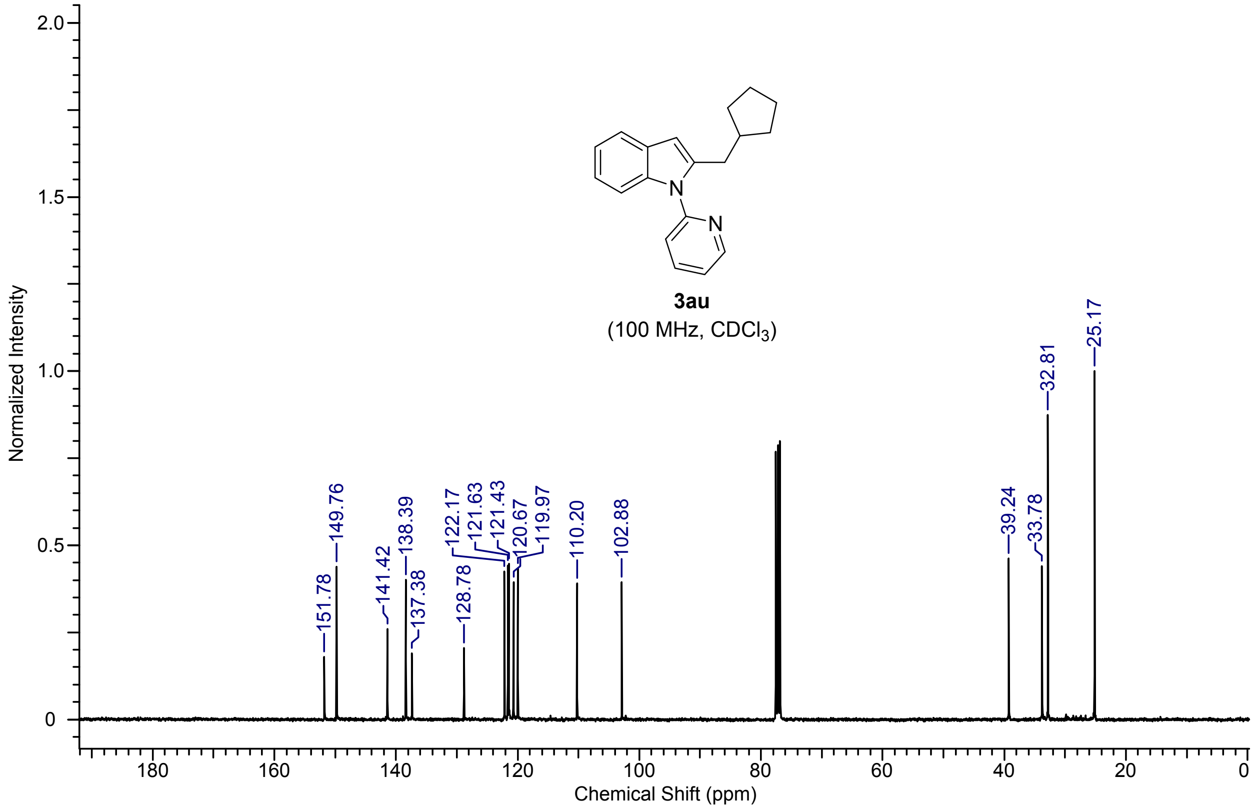

N
3au
(100 MHz, $CDCl_3$)
Normalized Intensity
151.78 149.76 141.42 138.39 137.38 128.78 122.17 121.63 121.43 120.67 119.97 110.20 102.88
39.24 33.78 32.81 25.17
Chemical Shift (ppm)

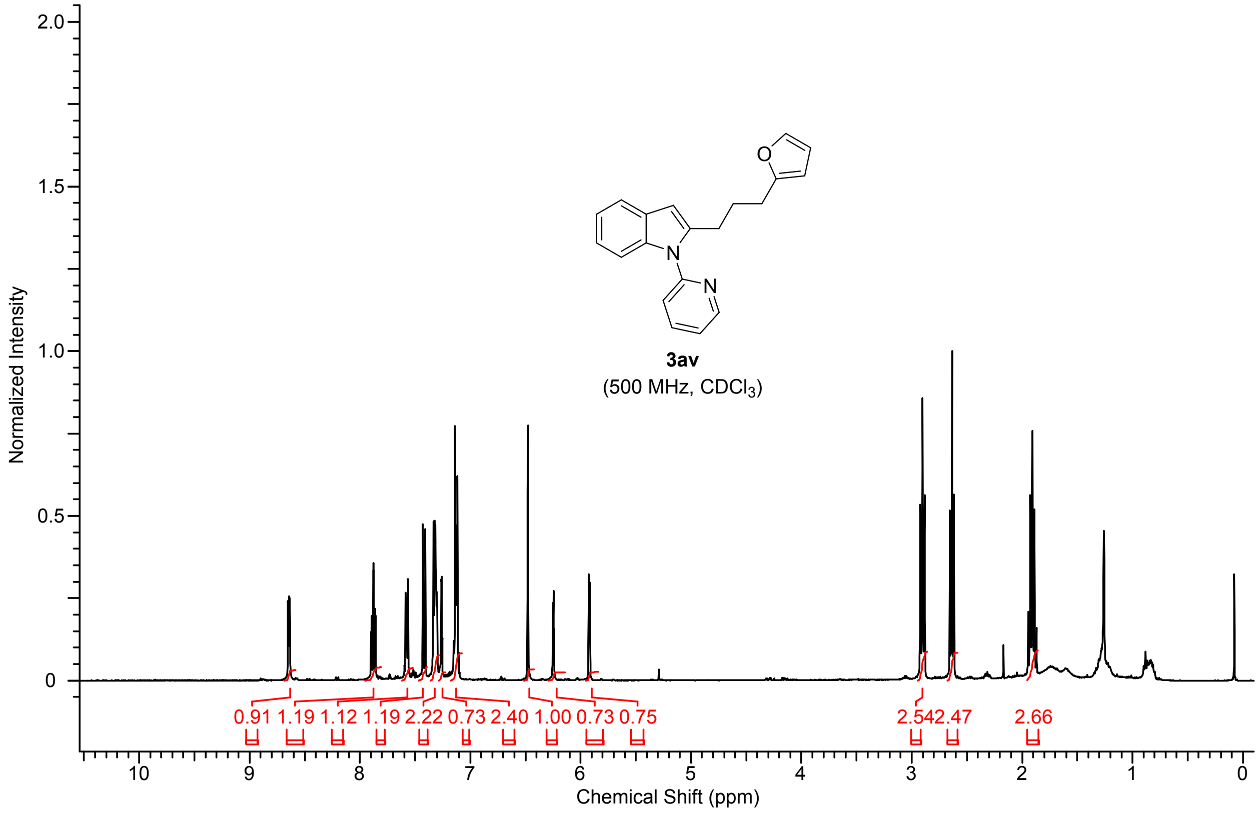

3av
(500 MHz, $CDCl_3$)
Normalized Intensity
Chemical Shift (ppm)
0.91 1.19 1.12 1.19 2.22 0.73 2.40 1.00 0.73 0.75
2.54 2.47
2.66

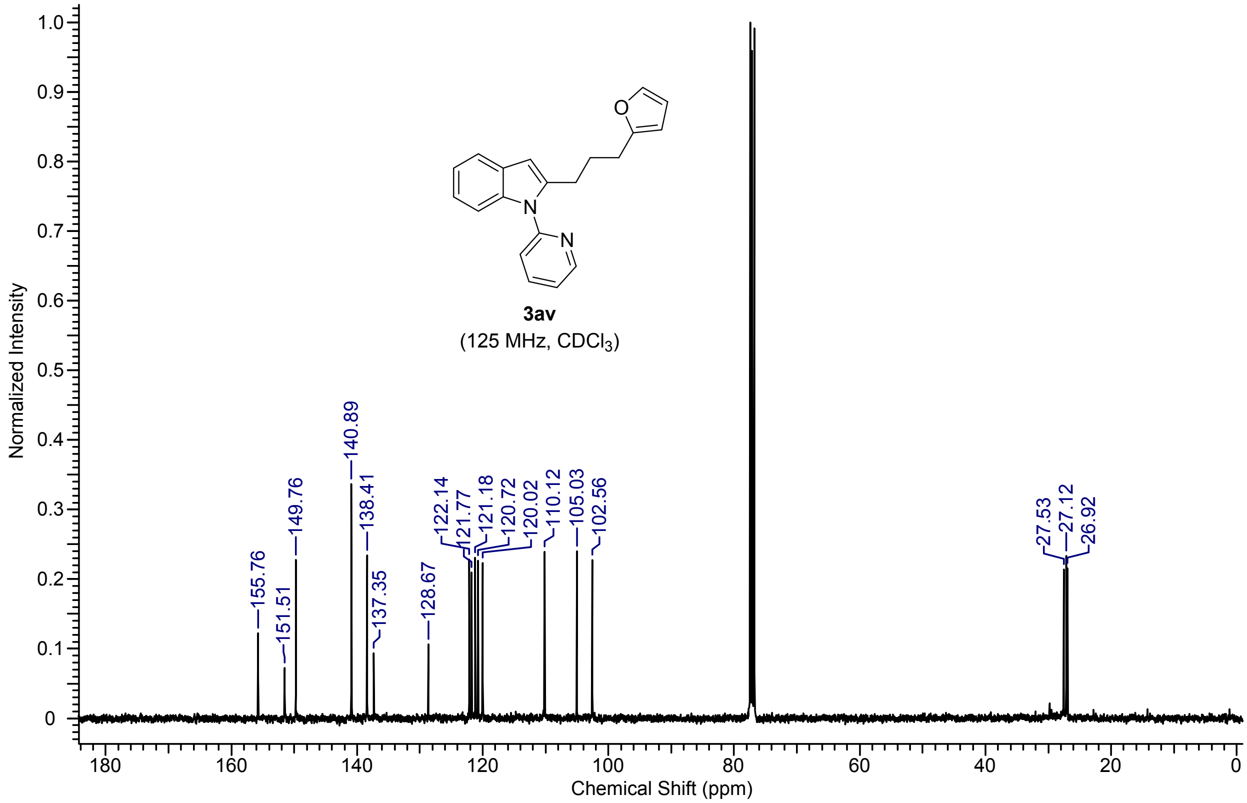

3av
(125 MHz, $CDCl_3$)
Normalized Intensity
Chemical Shift (ppm)
155.76
151.51
149.76
140.89
138.41
137.35
128.67
122.14
121.77
121.18
120.72
120.02
110.12
105.03
102.56
27.53
27.12
26.92

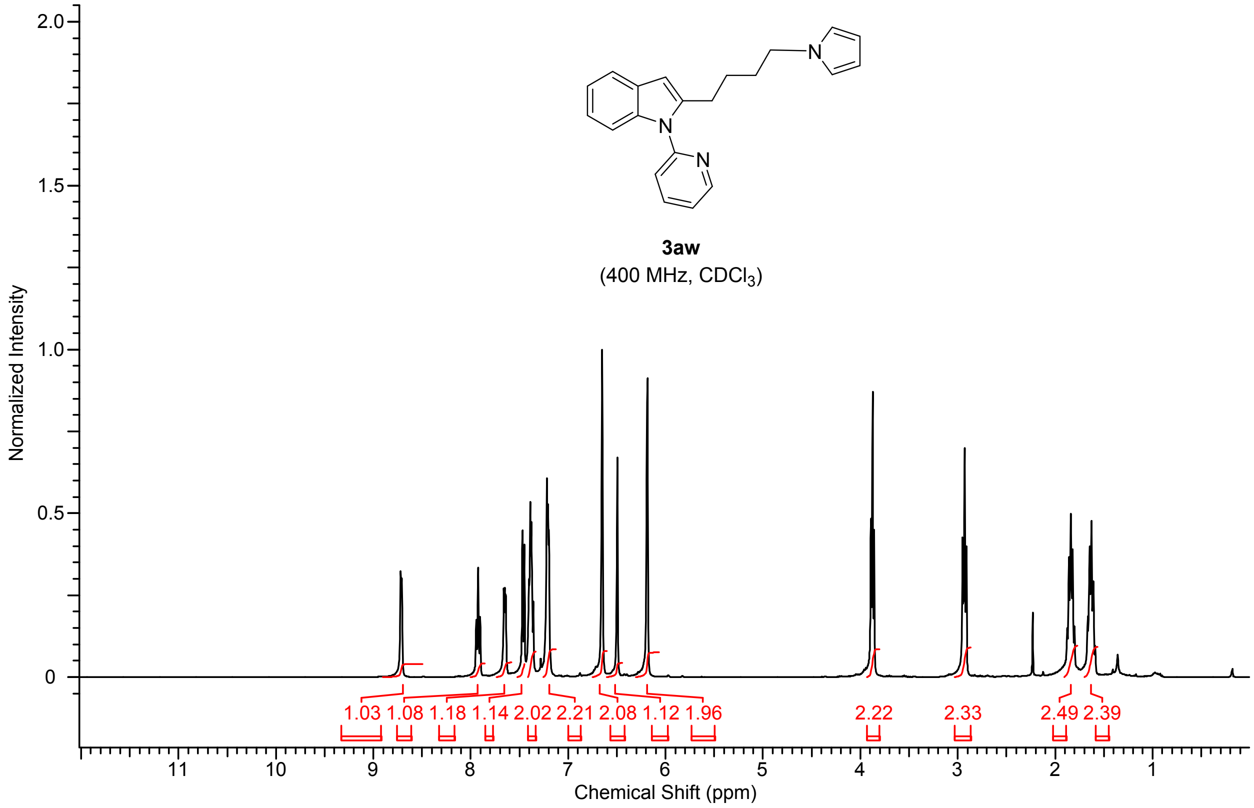

3aw
(400 MHz, $CDCl_3$)
Normalized Intensity
Chemical Shift (ppm)
1.03 1.08 1.18 1.14 2.02 2.21 2.08 1.12 1.96 2.22 2.33 2.49 2.39

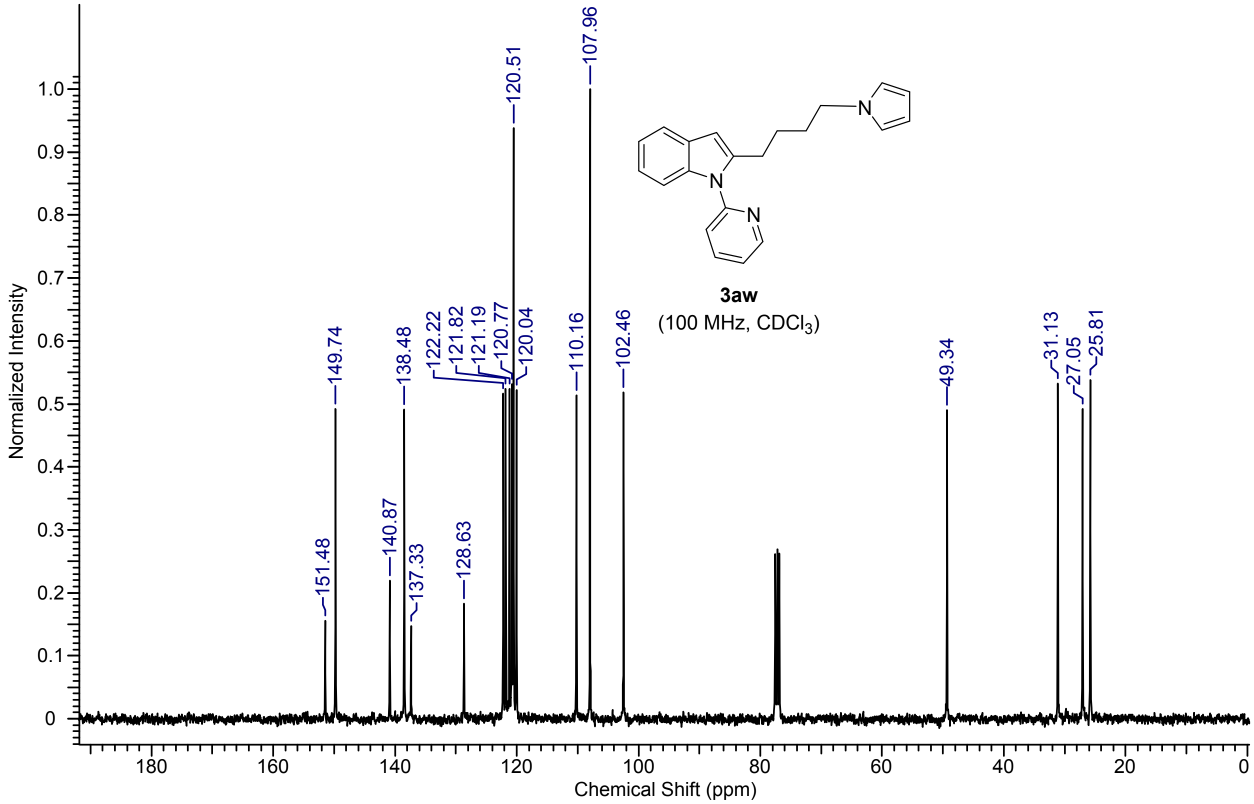

3aw
(100 MHz, $CDCl_3$)
Normalized Intensity
Chemical Shift (ppm)
151.48 149.74 140.87 138.48 137.33 128.63 122.22 121.82 121.19 120.77 120.51 120.04 110.16 107.96 102.46 49.34 31.13 27.05 25.81

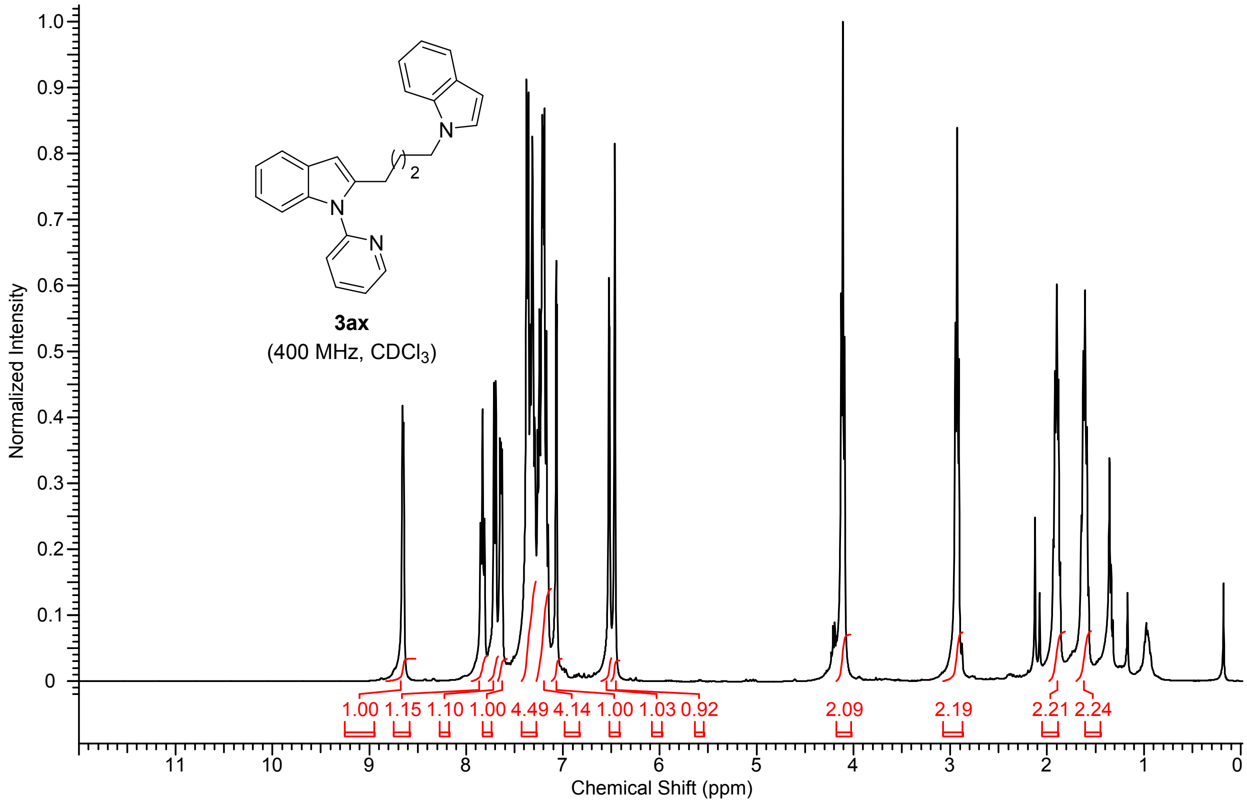
3ax
(400 MHz, CDCl3)
Normalized Intensity
Chemical Shift (ppm)
1.00 1.15 1.10 1.00 4.49 4.14 1.00 1.03 0.92 2.09 2.19 2.21 2.24

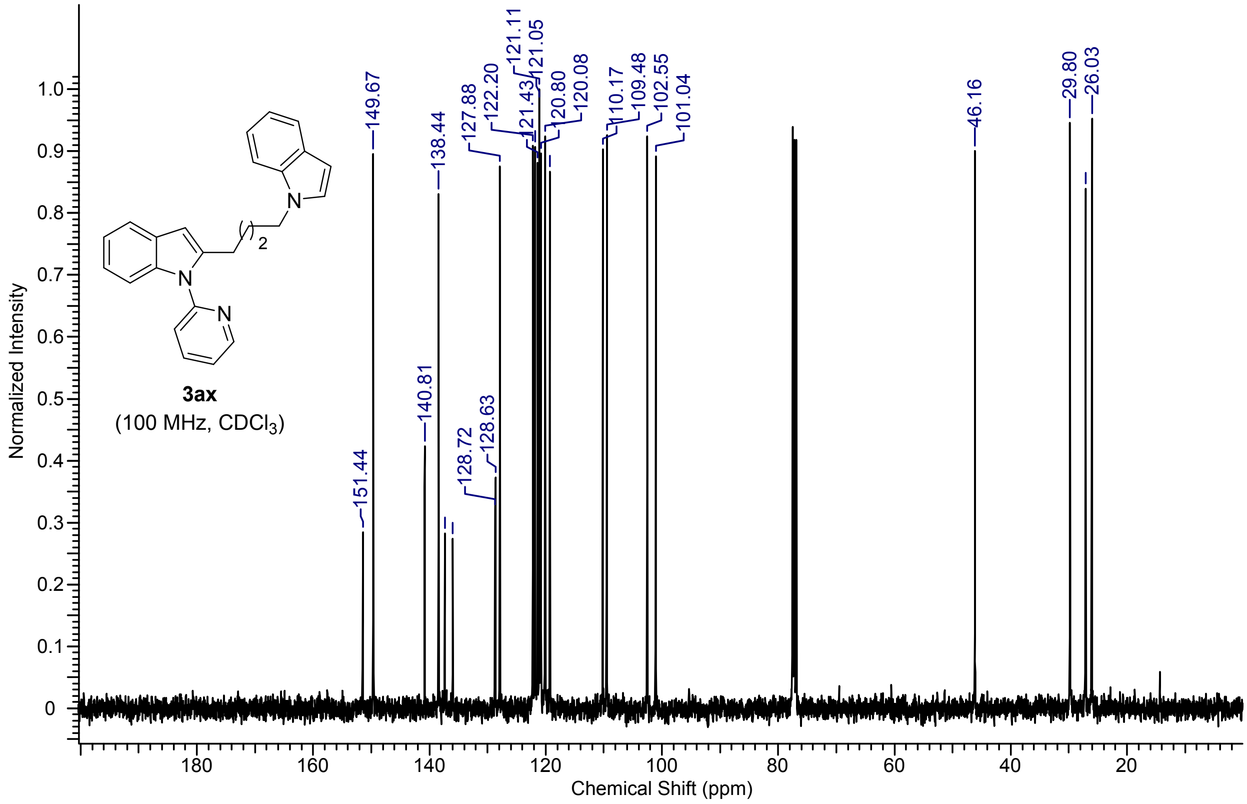
3ax
(100 MHz, CDCl3)
Normalized Intensity
Chemical Shift (ppm)
151.44
149.67
140.81
138.44
128.72
128.63
127.88
122.20
121.43
121.11
121.05
120.80
120.08
110.17
109.48
102.55
101.04
46.16
29.80
26.03

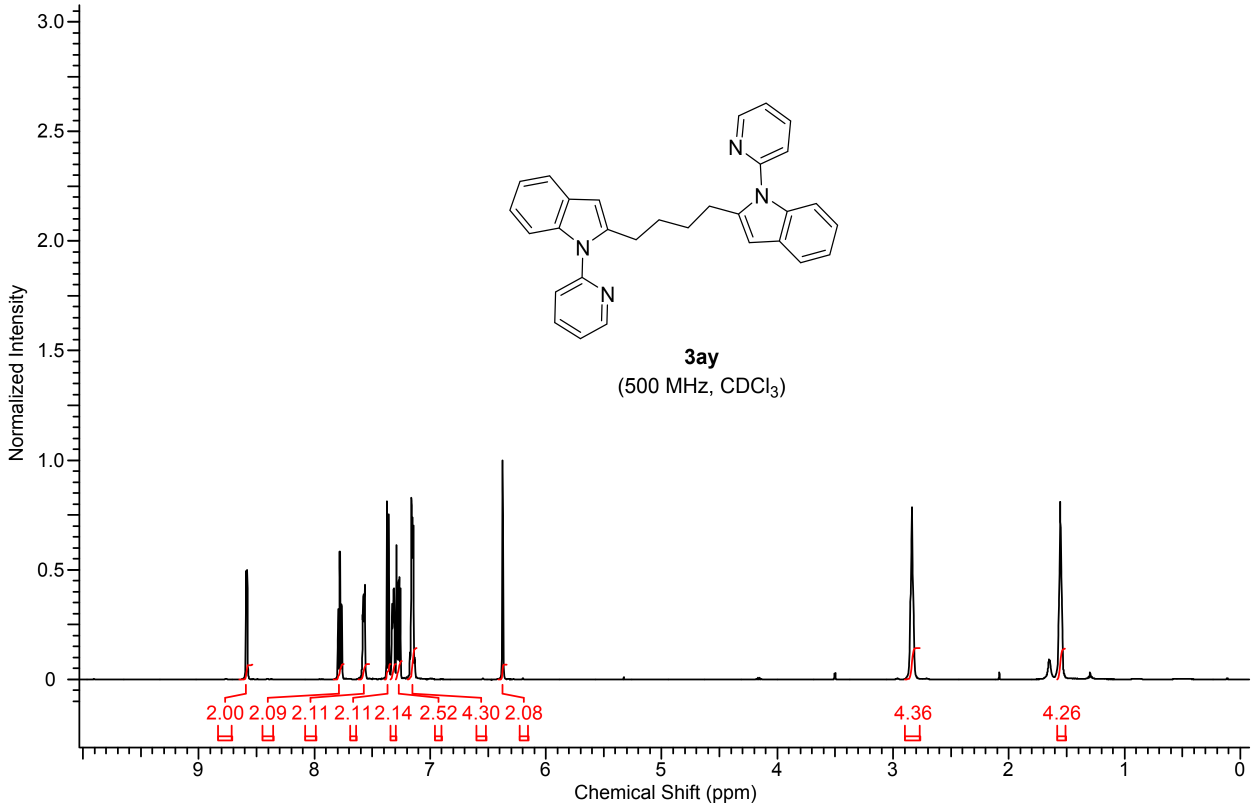
N
N
N
N
3ay
(500 MHz, $CDCl_3$)
Normalized Intensity
Chemical Shift (ppm)
2.00 2.09 2.11 2.11 2.14 2.52 4.30 2.08
4.36
4.26

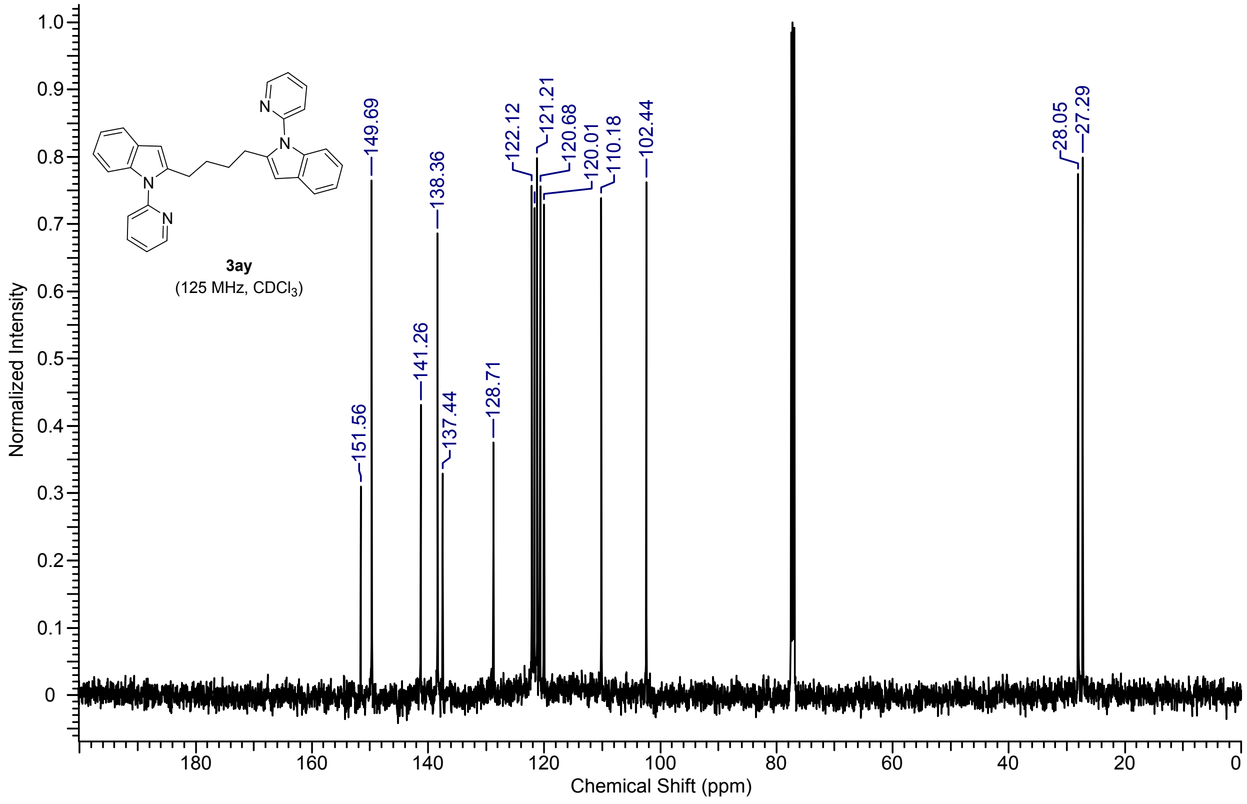
N
N
N
N
3ay
(125 MHz, $CDCl_3$)
Normalized Intensity
Chemical Shift (ppm)
151.56
149.69
141.26
138.36
137.44
128.71
122.12
121.21
120.68
120.01
110.18
102.44
28.05
27.29

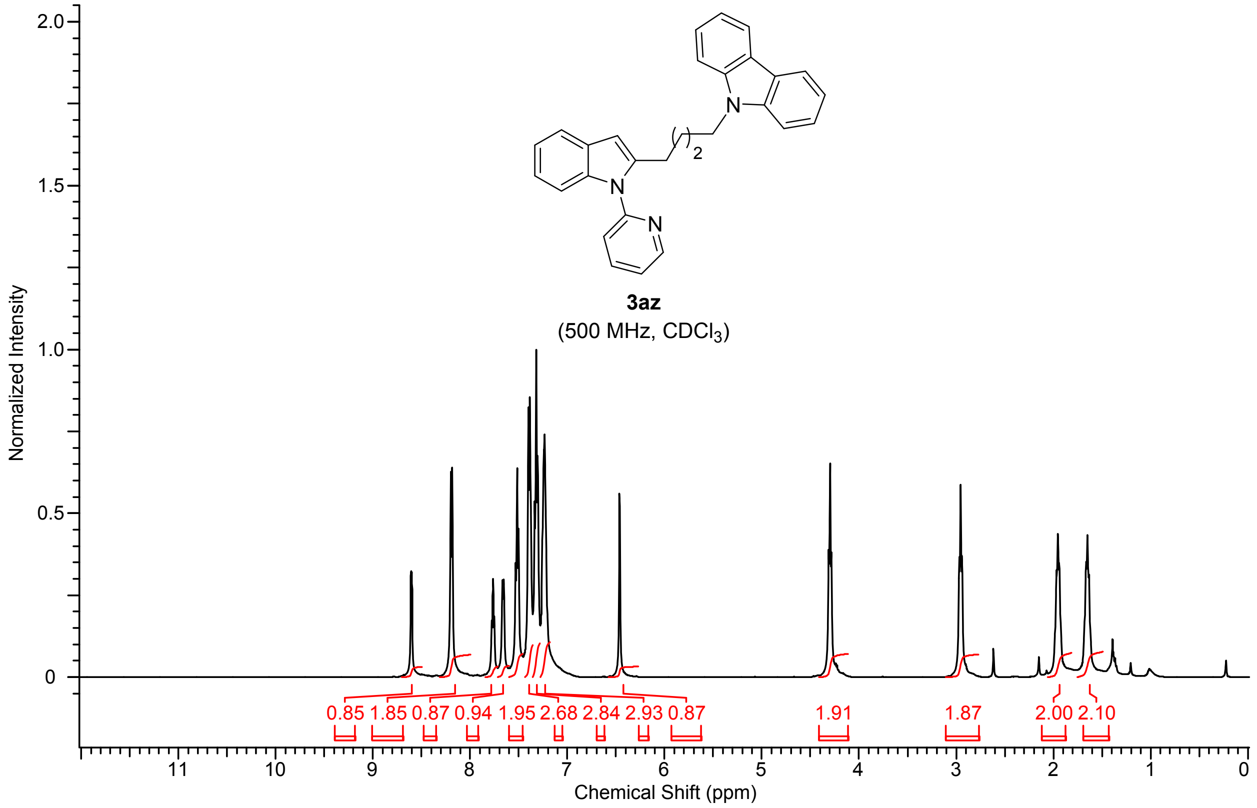

3az
(500 MHz, CDCl3)
0.85 1.85 0.87 0.94 1.95 2.68 2.84 2.93 0.87
1.91
1.87
2.00 2.10
Normalized Intensity
Chemical Shift (ppm)

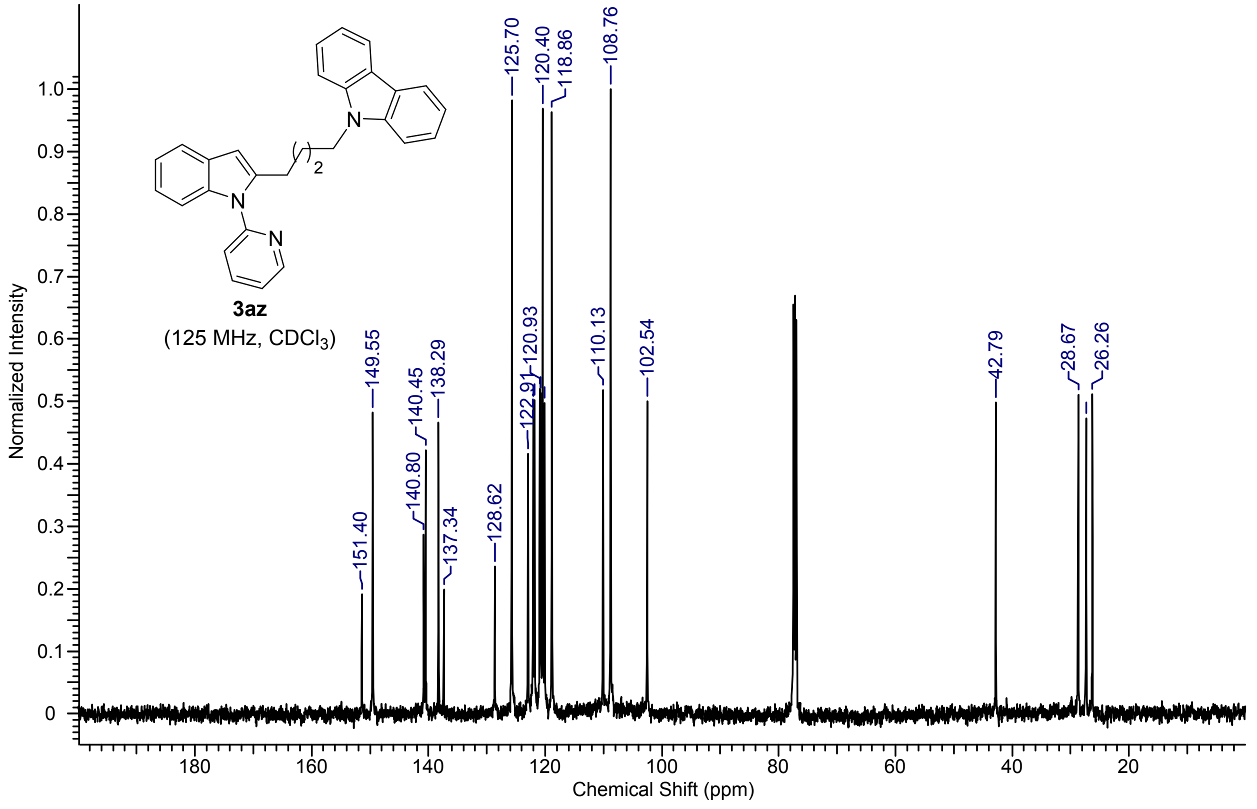

3az
(125 MHz, CDCl3)
151.40
149.55
140.80
140.45
138.29
137.34
128.62
125.70
122.91
120.93
120.40
118.86
110.13
108.76
102.54
42.79
28.67
26.26
Normalized Intensity
Chemical Shift (ppm)

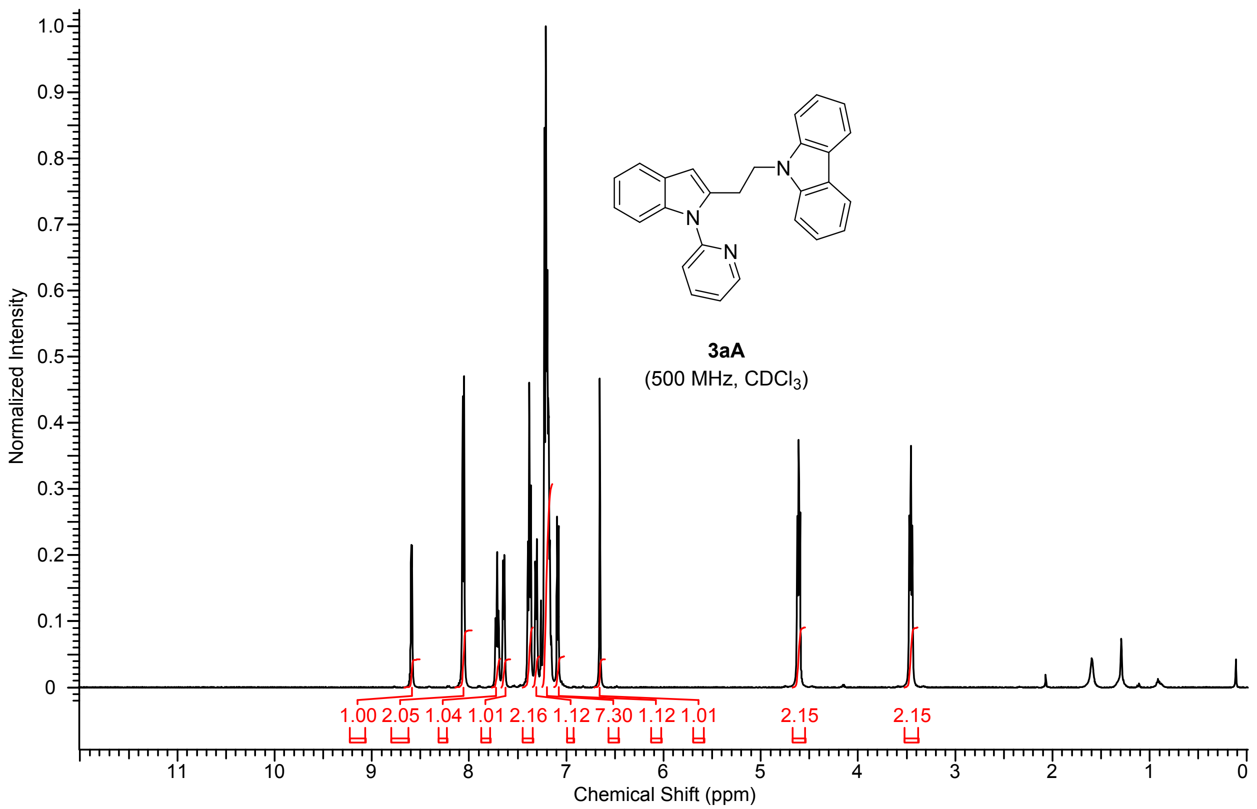

3aA
(500 MHz, $CDCl_3$)
1.00 2.05 1.04 1.01 2.16 1.12 7.30 1.12 1.01
2.15
2.15
Normalized Intensity
Chemical Shift (ppm)

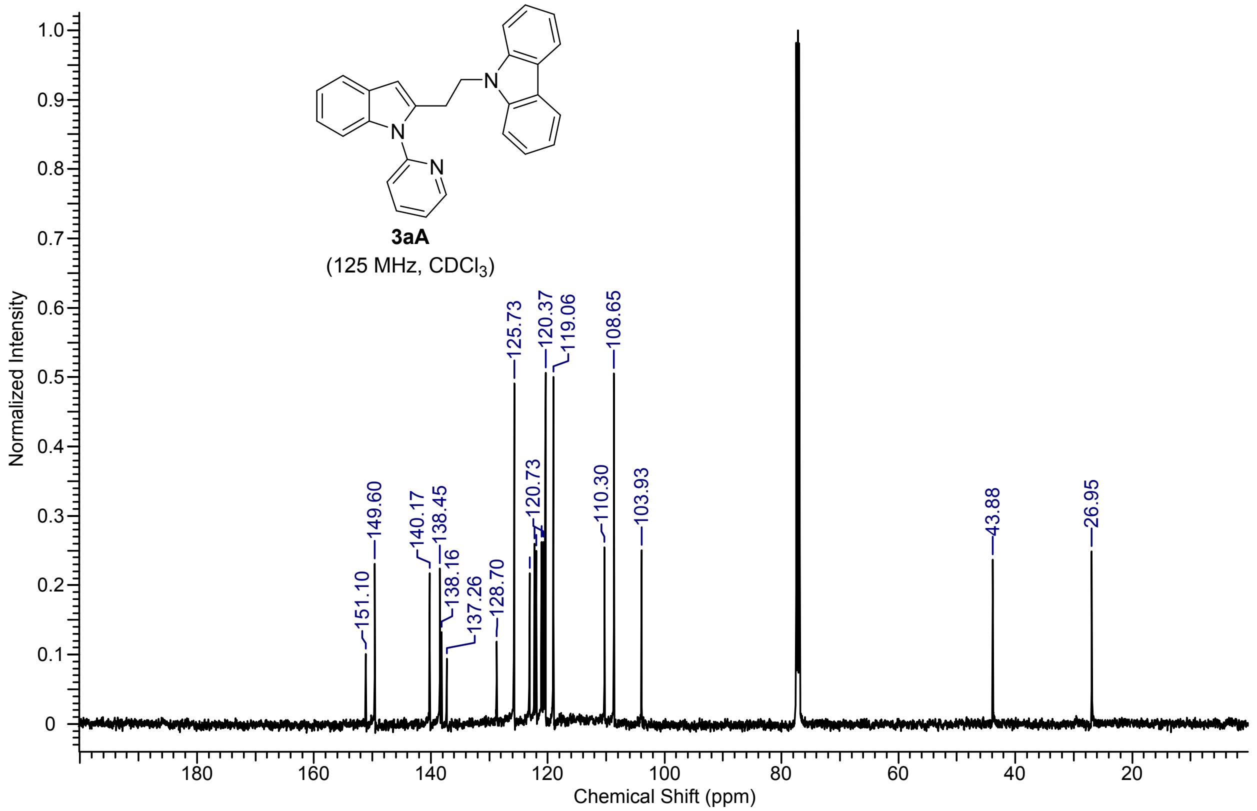

3aA
(125 MHz, $CDCl_3$)
151.10
149.60
140.17
138.45
138.16
137.26
128.70
125.73
120.73
120.37
119.06
110.30
108.65
103.93
43.88
26.95
Normalized Intensity
Chemical Shift (ppm)

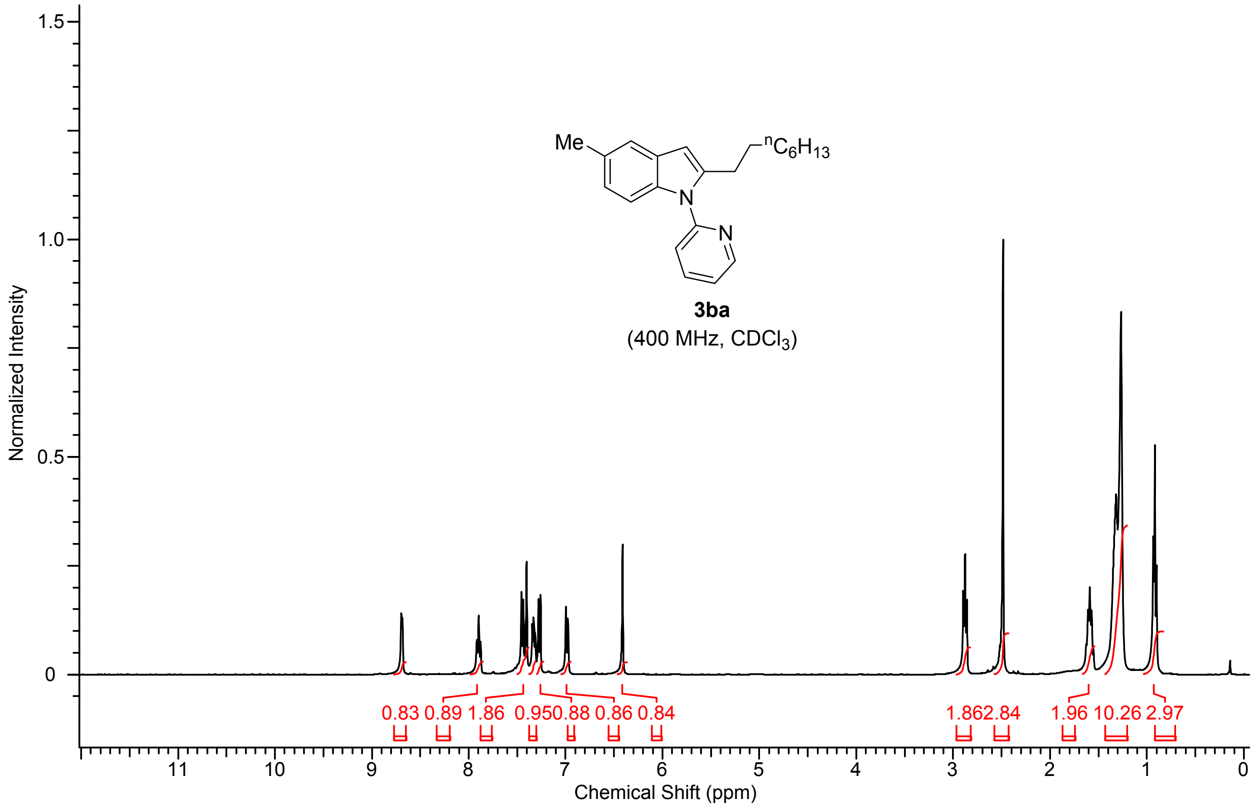
Me
nC6H13
N
N
3ba
(400 MHz, CDCl3)
Normalized Intensity
Chemical Shift (ppm)
0.83 0.89 1.86 0.950.88 0.86 0.84
1.862.84
1.96 10.26 2.97

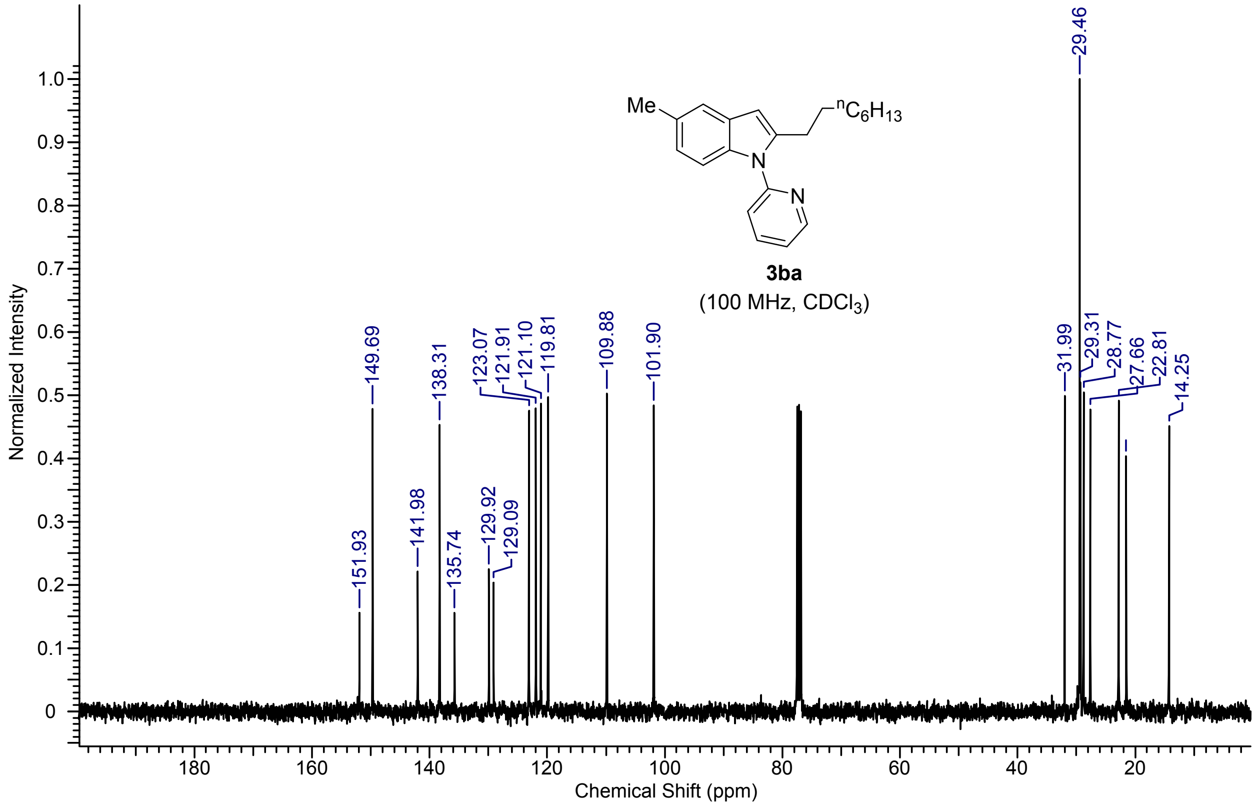
Me
nC6H13
N
N
3ba
(100 MHz, CDCl3)
Normalized Intensity
Chemical Shift (ppm)
151.93
149.69
141.98
138.31
135.74
129.92
129.09
123.07
121.91
121.10
119.81
109.88
101.90
31.99
29.46
29.31
28.77
27.66
22.81
14.25

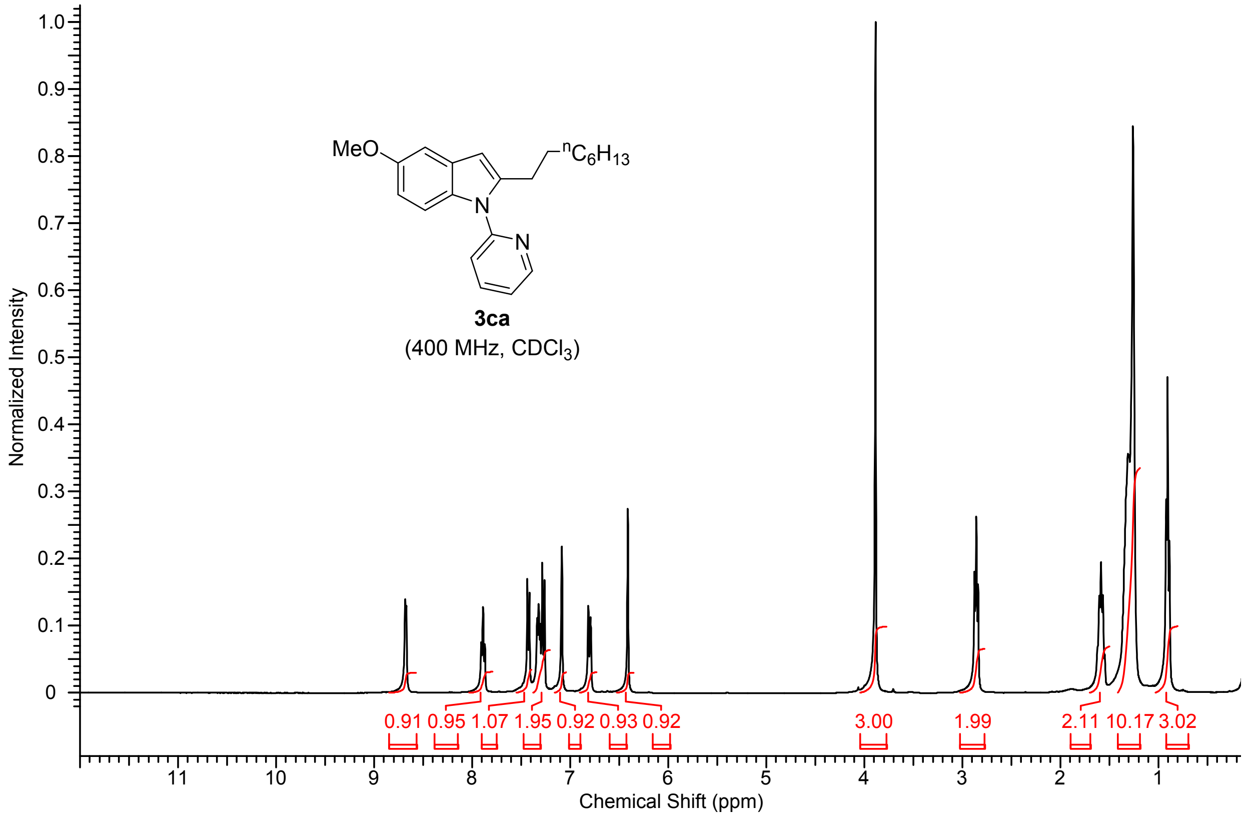

MeO
$^{n}C_6H_{13}$
N
N
3ca
(400 MHz, CDCl$_3$)
Normalized Intensity
Chemical Shift (ppm)
0.91 0.95 1.07 1.95 0.92 0.93 0.92
3.00
1.99
2.11 10.17 3.02

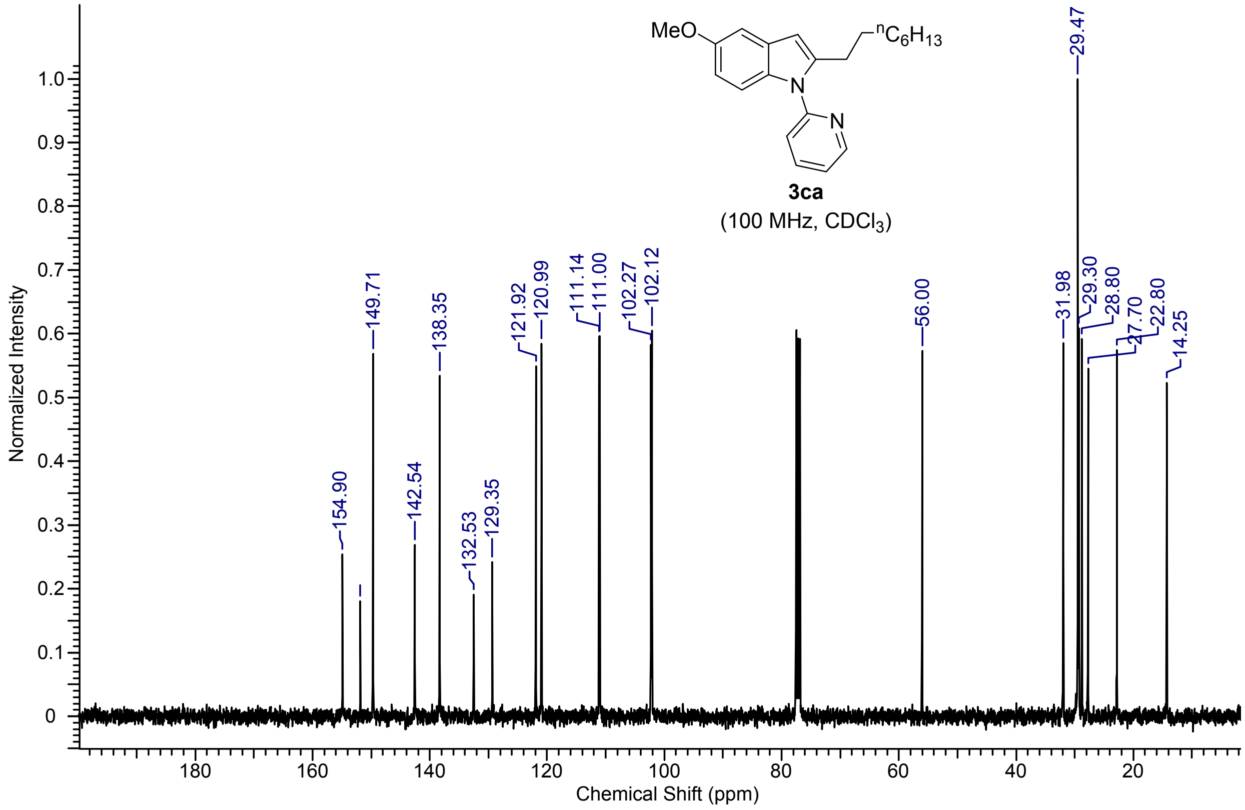

MeO
$^{n}C_6H_{13}$
N
N
3ca
(100 MHz, CDCl$_3$)
Normalized Intensity
Chemical Shift (ppm)
154.90
149.71
142.54
138.35
132.53
129.35
121.92
120.99
111.14
111.00
102.27
102.12
56.00
31.98
29.47
29.30
28.80
27.70
22.80
14.25

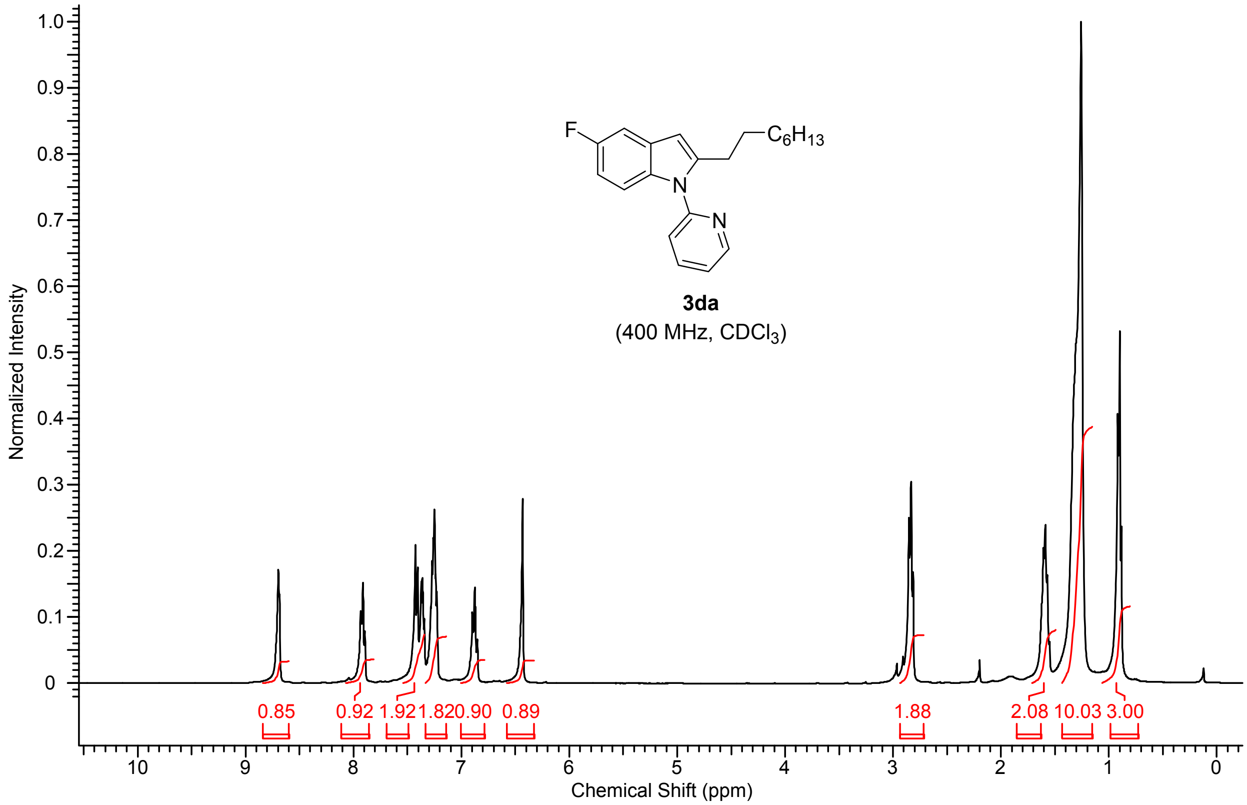
F
N
N
C_6H_{13}
3da
(400 MHz, $CDCl_3$)
0.85
0.92
1.92
1.82
0.90
0.89
1.88
2.08
10.03
3.00
Normalized Intensity
Chemical Shift (ppm)

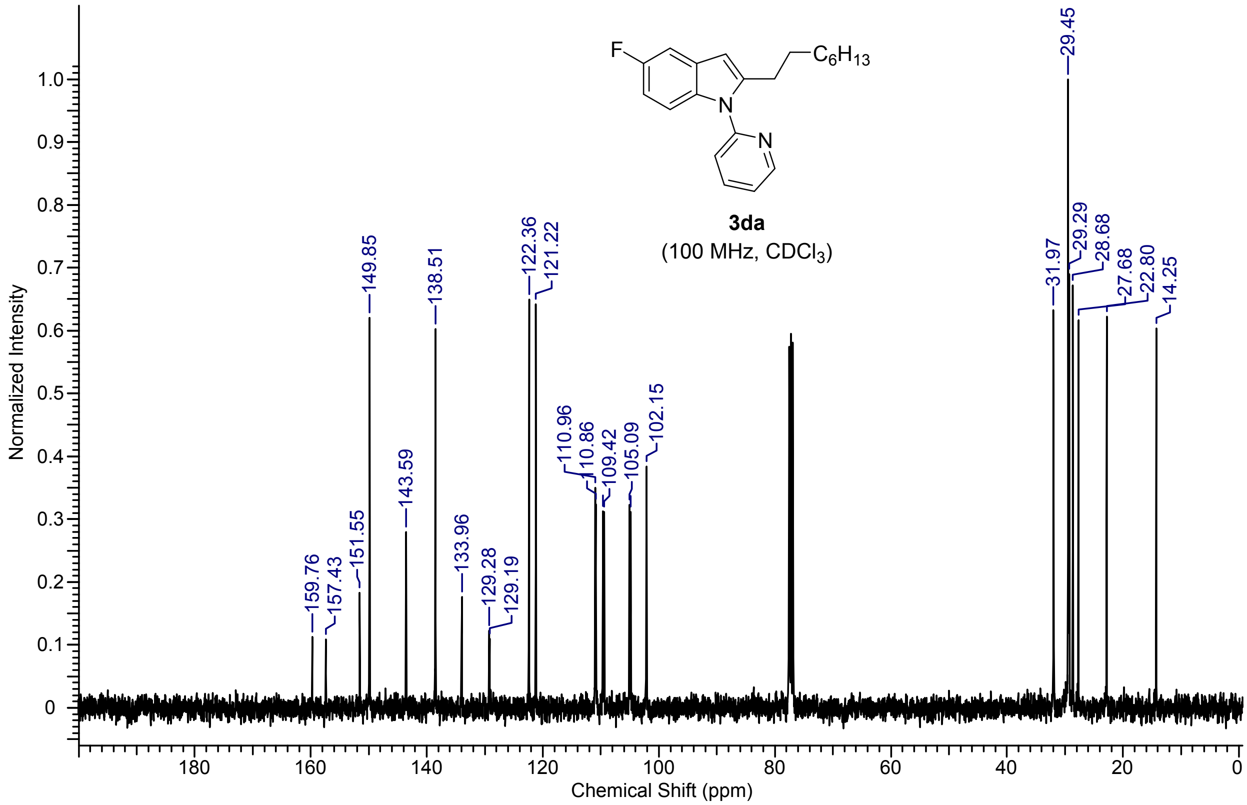
F
N
N
C_6H_{13}
3da
(100 MHz, $CDCl_3$)
159.76
157.43
151.55
149.85
143.59
138.51
133.96
129.28
129.19
122.36
121.22
110.96
110.86
109.42
105.09
102.15
31.97
29.45
29.29
28.68
27.68
22.80
14.25
Normalized Intensity
Chemical Shift (ppm)

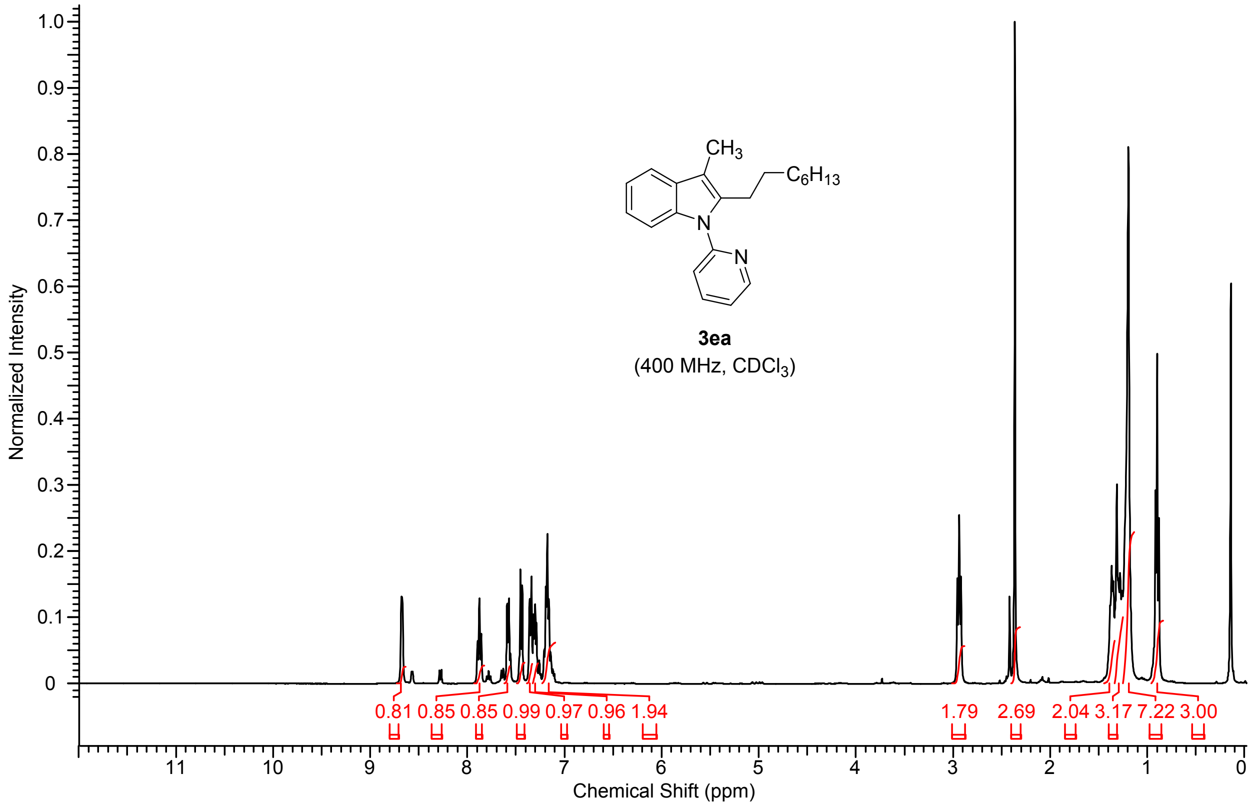

CH3
C6H13
N
N
3ea
(400 MHz, CDCl3)
Normalized Intensity
Chemical Shift (ppm)
0.81 0.85 0.85 0.99 0.97 0.96 1.94
1.79 2.69 2.04 3.17 7.22 3.00

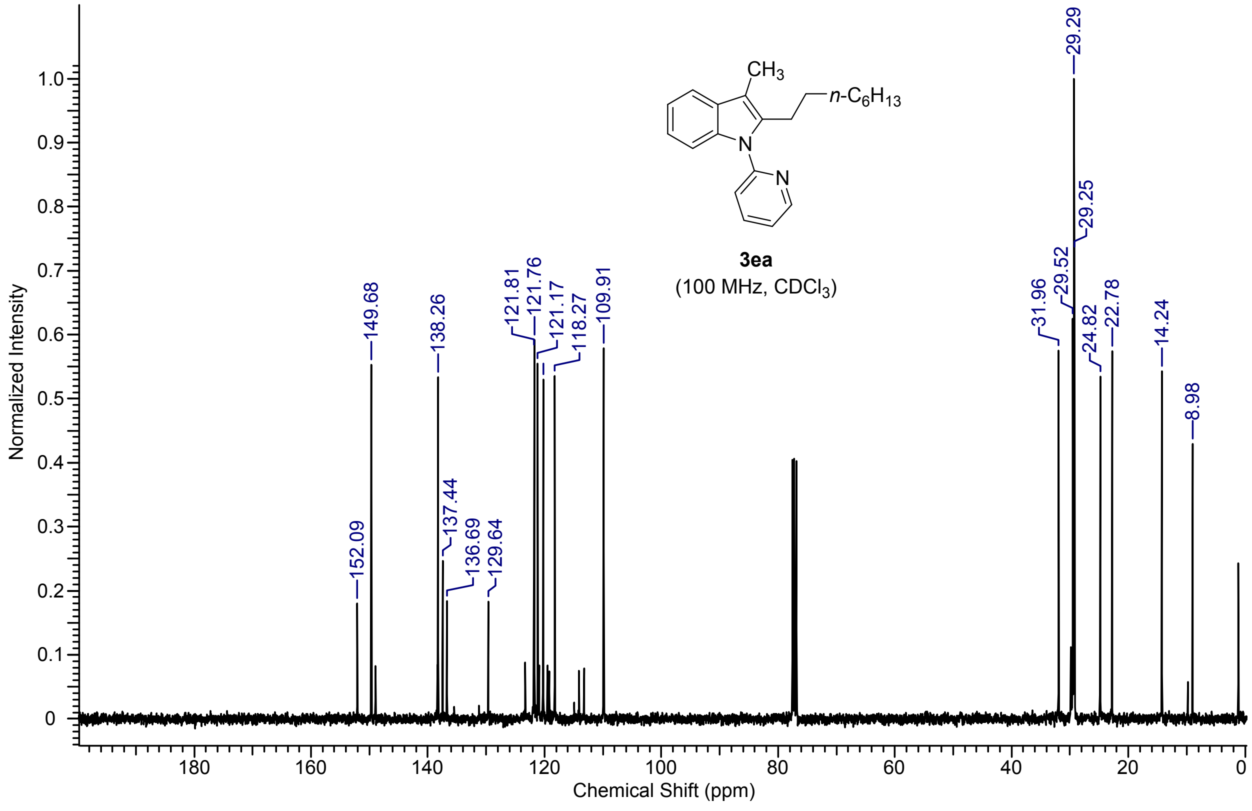

CH3
n-C6H13
N
N
3ea
(100 MHz, CDCl3)
Normalized Intensity
Chemical Shift (ppm)
152.09
149.68
138.26
137.44
136.69
129.64
121.81
121.76
121.17
118.27
109.91
31.96
29.52
29.29
29.25
24.82
22.78
14.24
8.98

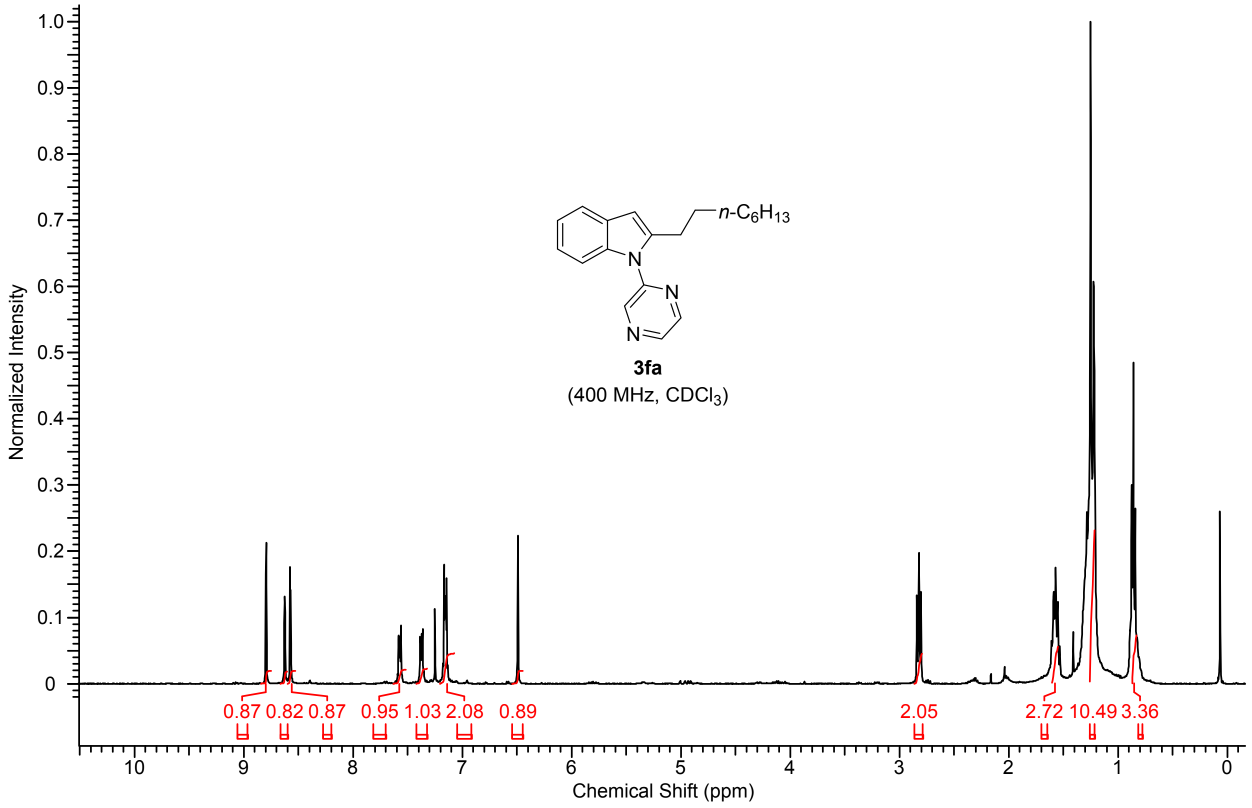

n-C6H13
N
N
N
3fa
(400 MHz, CDCl3)
Normalized Intensity
Chemical Shift (ppm)
0.87 0.82 0.87 0.95 1.03 2.08 0.89 2.05 2.72 10.49 3.36

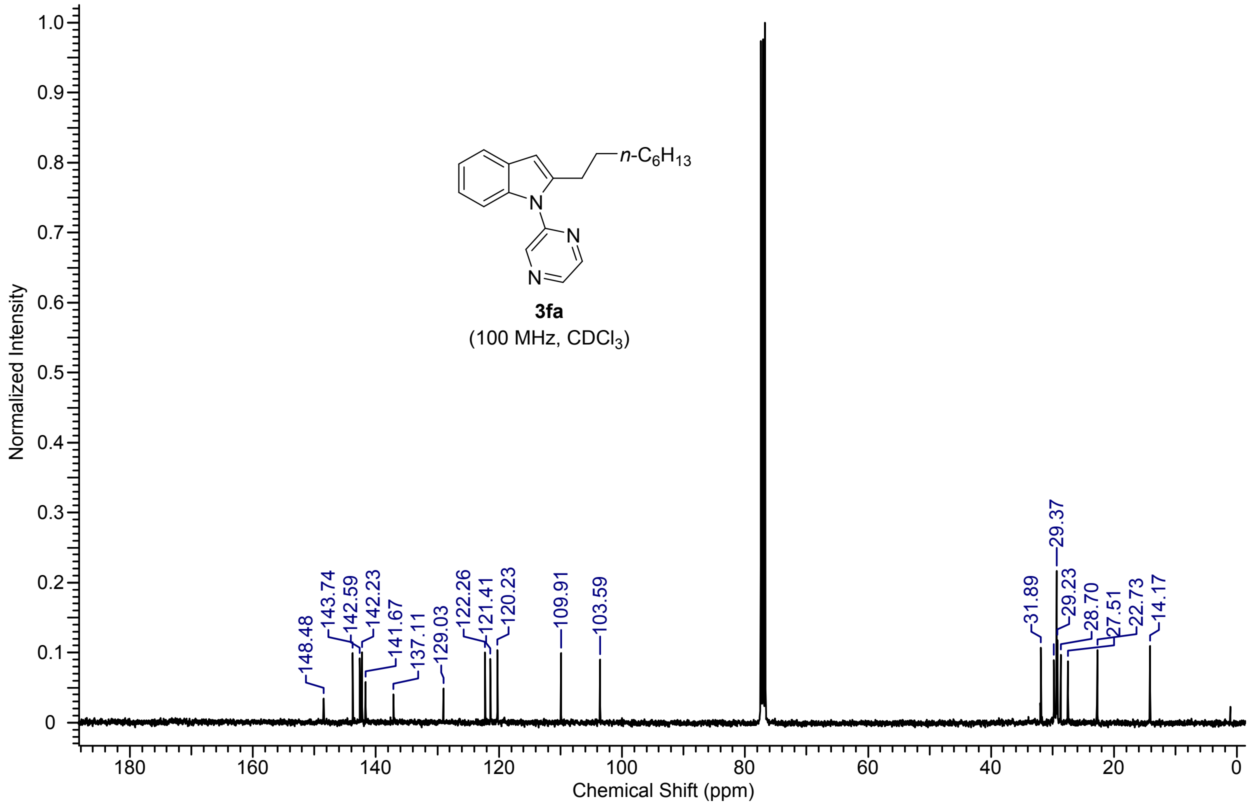

n-C6H13
N
N
N
3fa
(100 MHz, CDCl3)
Normalized Intensity
Chemical Shift (ppm)
148.48 143.74 142.59 142.23 141.67 137.11 129.03 122.26 121.41 120.23 109.91 103.59
31.89 29.37 29.23 28.70 27.51 22.73 14.17

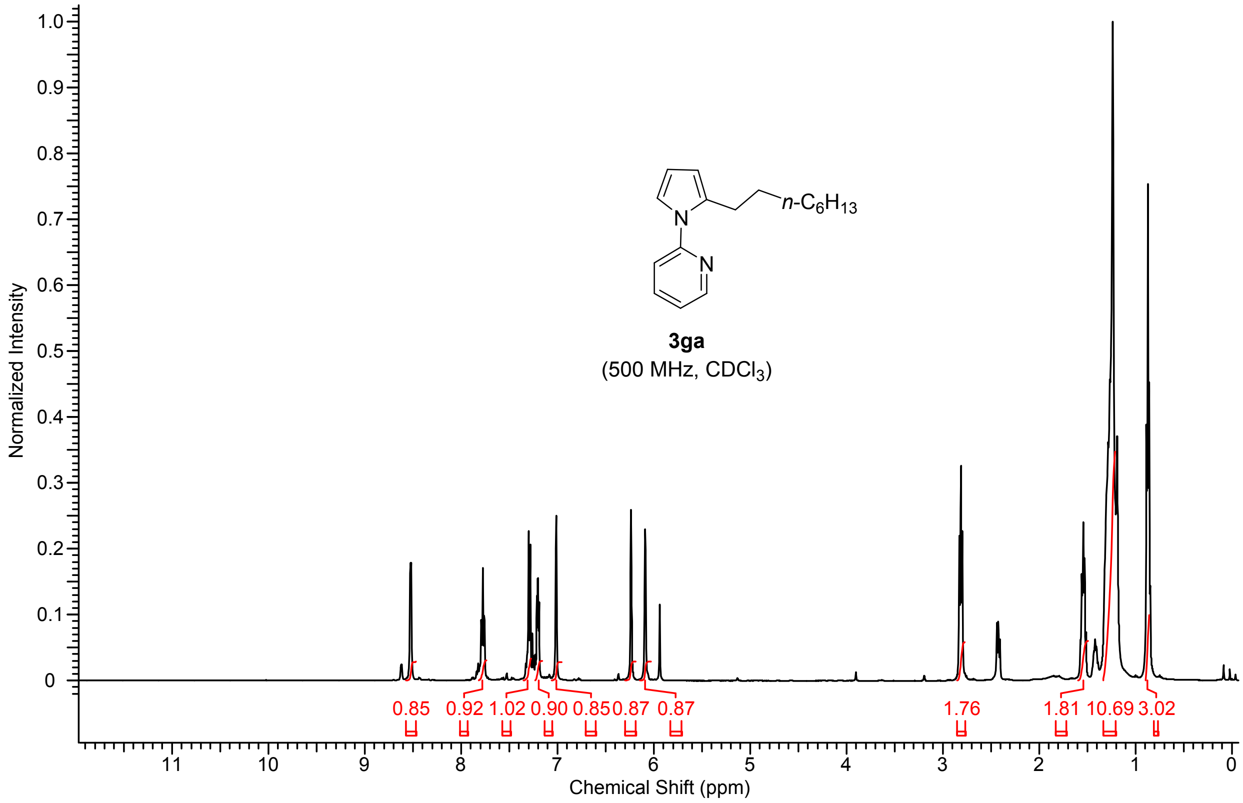

n-C6H13
N
N
3ga
(500 MHz, CDCl3)
Normalized Intensity
0.85
0.92
1.02
0.90
0.85
0.87
0.87
1.76
1.81
10.69
3.02
Chemical Shift (ppm)

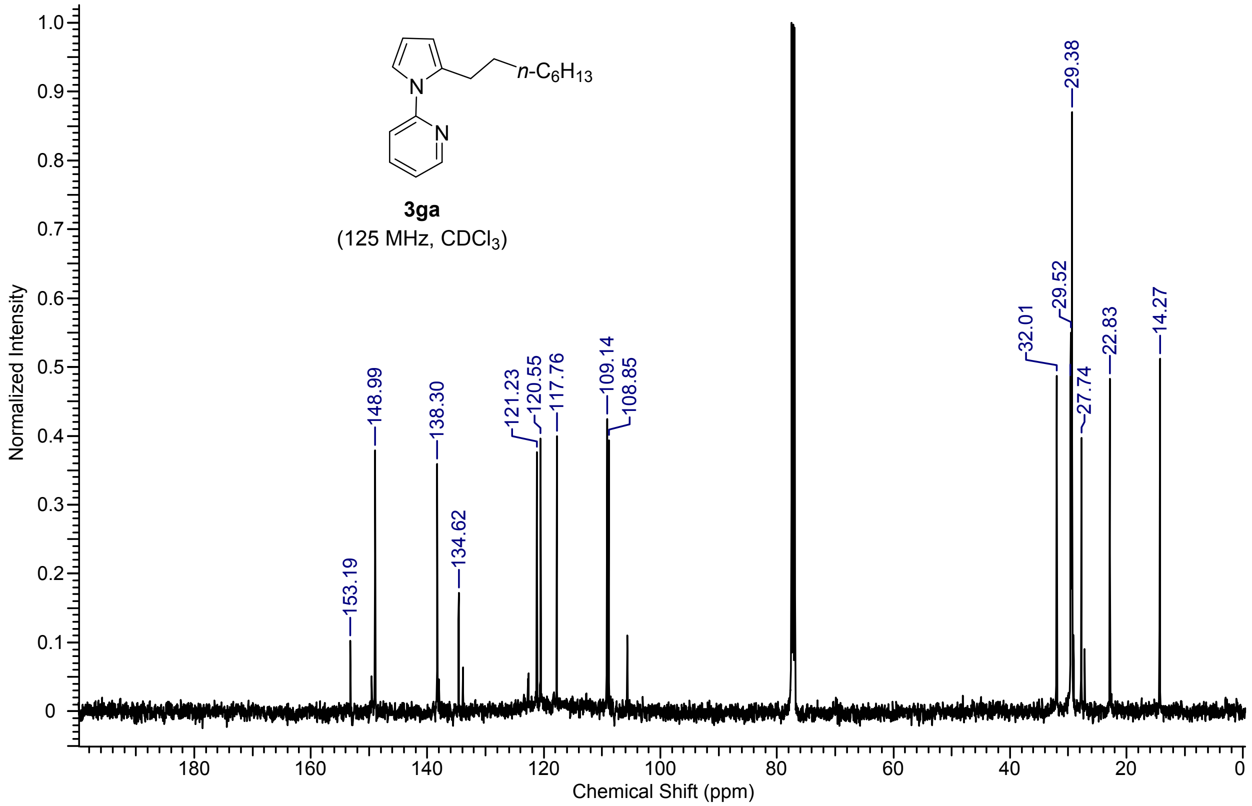

n-C6H13
N
N
3ga
(125 MHz, CDCl3)
Normalized Intensity
153.19
148.99
138.30
134.62
121.23
120.55
117.76
109.14
108.85
32.01
29.52
29.38
27.74
22.83
14.27
Chemical Shift (ppm)

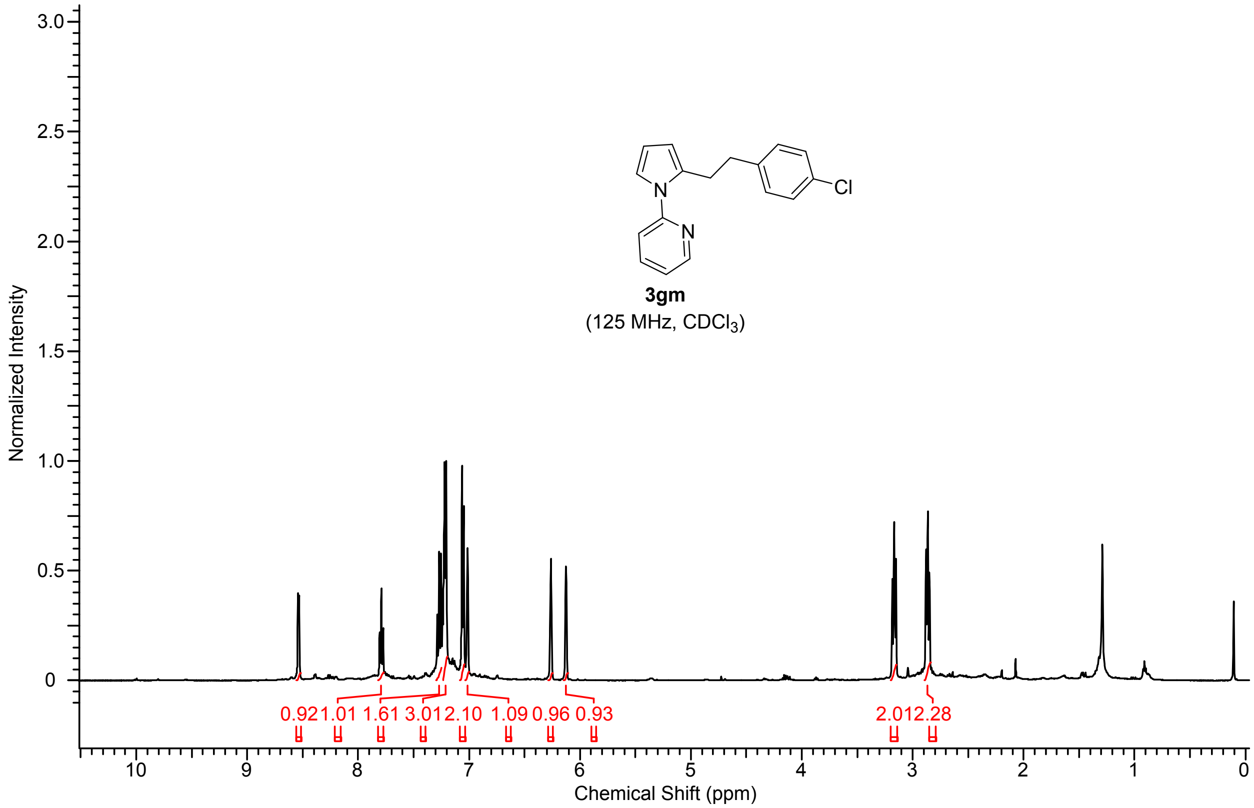

3gm
(125 MHz, CDCl3)
Normalized Intensity
Chemical Shift (ppm)
0.92 1.01 1.61 3.01 2.10 1.09 0.96 0.93 2.01 2.28

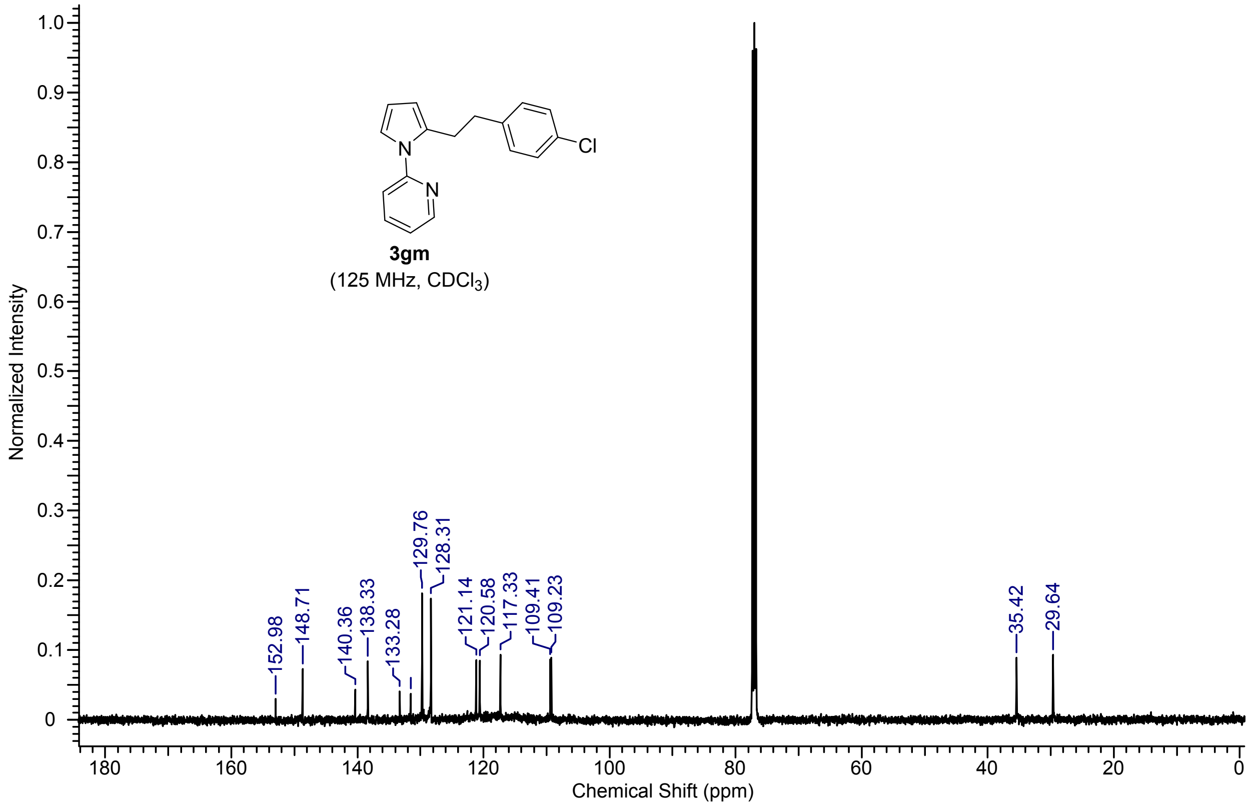

3gm
(125 MHz, CDCl3)
Normalized Intensity
Chemical Shift (ppm)
152.98 148.71 140.36 138.33 133.28 129.76 128.31 121.14 120.58 117.33 109.41 109.23 35.42 29.64

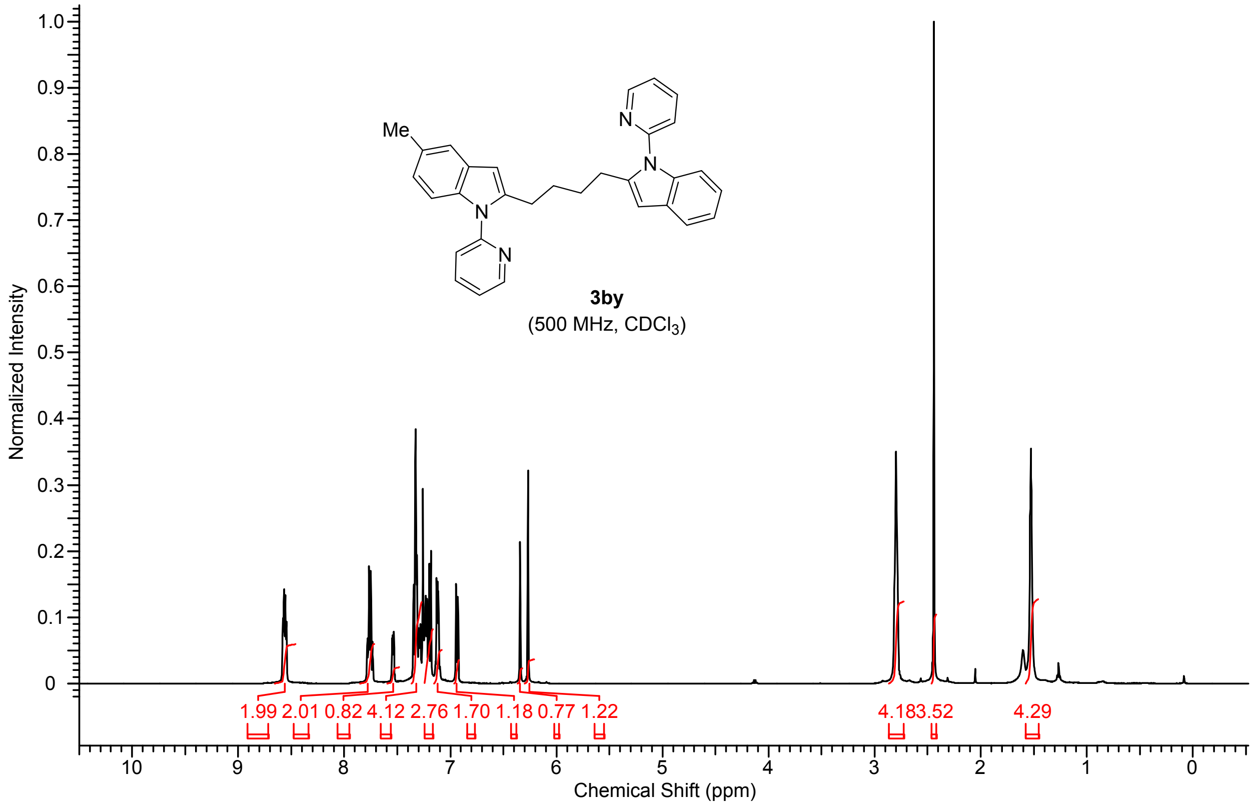

Me
N
N
N
N
3by
(500 MHz, $CDCl_3$)
1.99 2.01 0.82 4.12 2.76 1.70 1.18 0.77 1.22
4.18 3.52
4.29
Normalized Intensity
Chemical Shift (ppm)

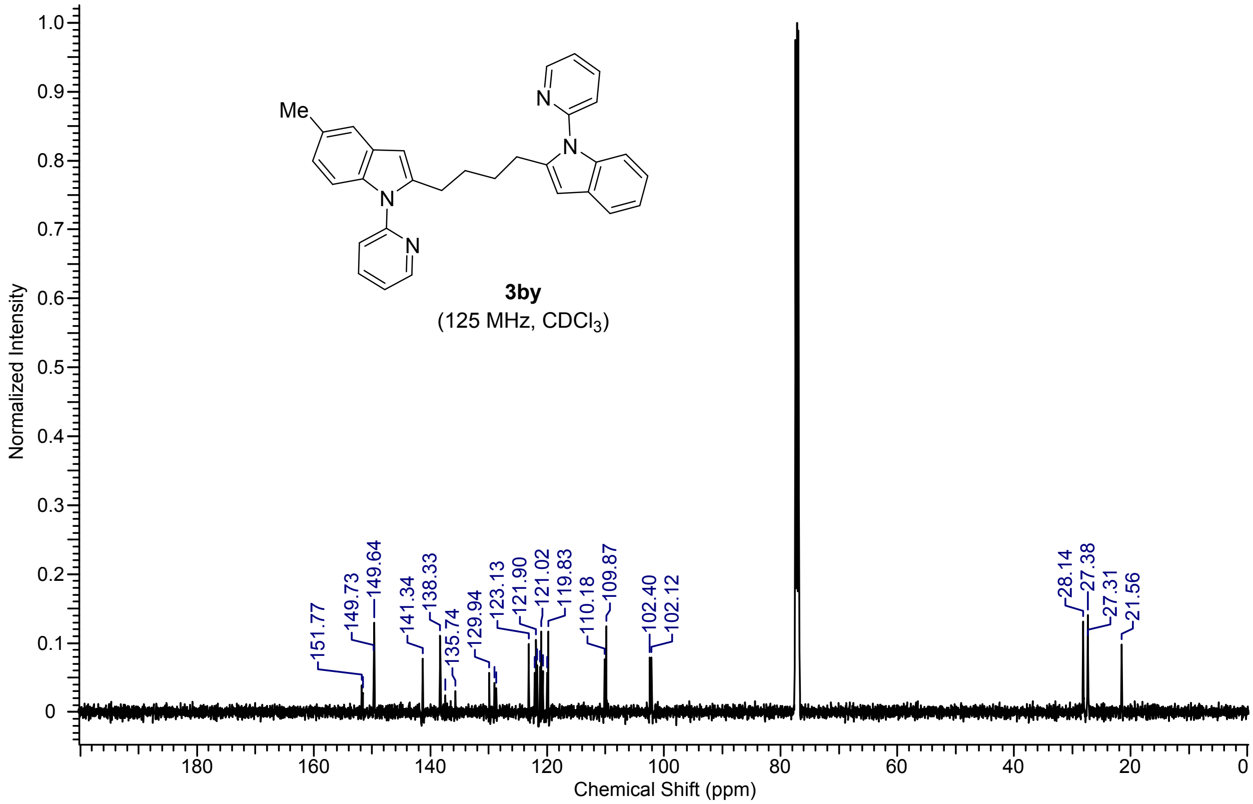

Me
N
N
N
N
3by
(125 MHz, $CDCl_3$)
151.77
149.73
149.64
141.34
138.33
135.74
129.94
123.13
121.90
121.02
119.83
110.18
109.87
102.40
102.12
28.14
27.38
27.31
21.56
Normalized Intensity
Chemical Shift (ppm)

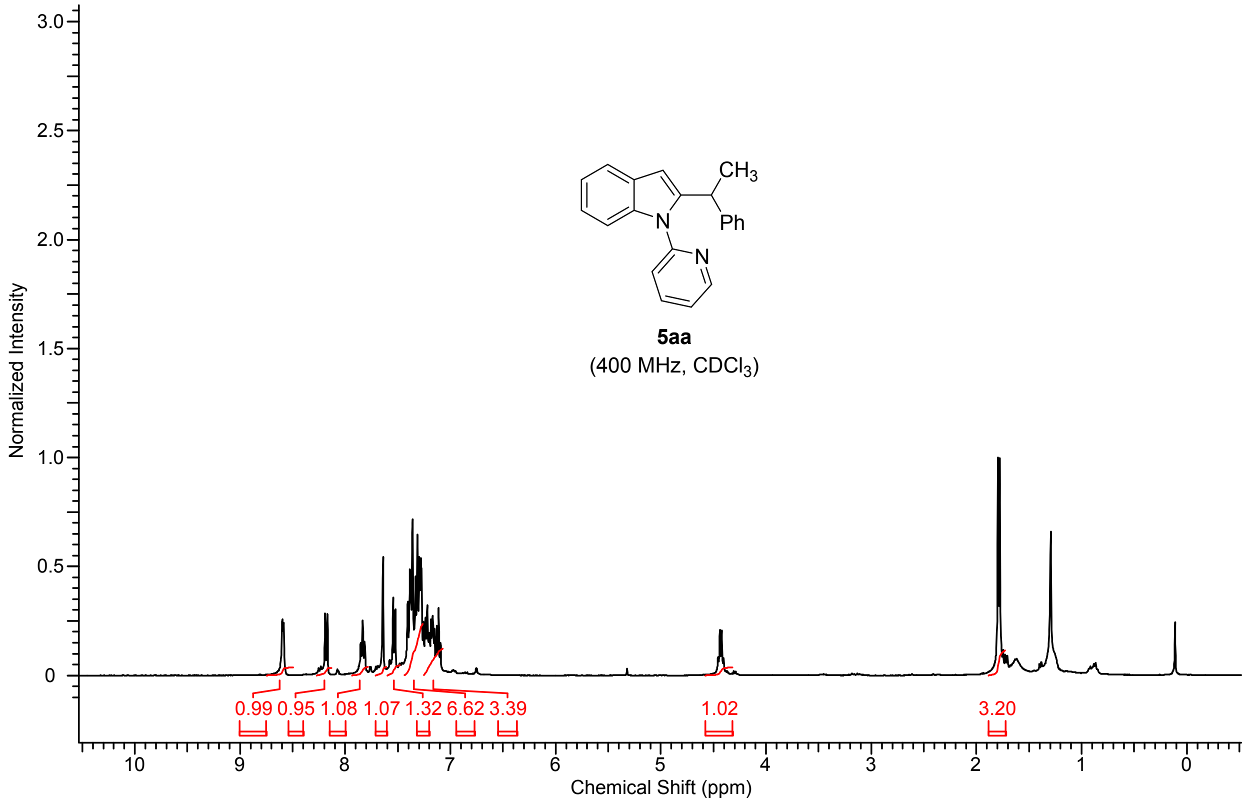

3.0
2.5
2.0
1.5
1.0
0.5
0
Normalized Intensity
CH3
Ph
N
N
5aa
(400 MHz, CDCl3)
0.99 0.95 1.08 1.07 1.32 6.62 3.39
1.02
3.20
10 9 8 7 6 5 4 3 2 1 0
Chemical Shift (ppm)

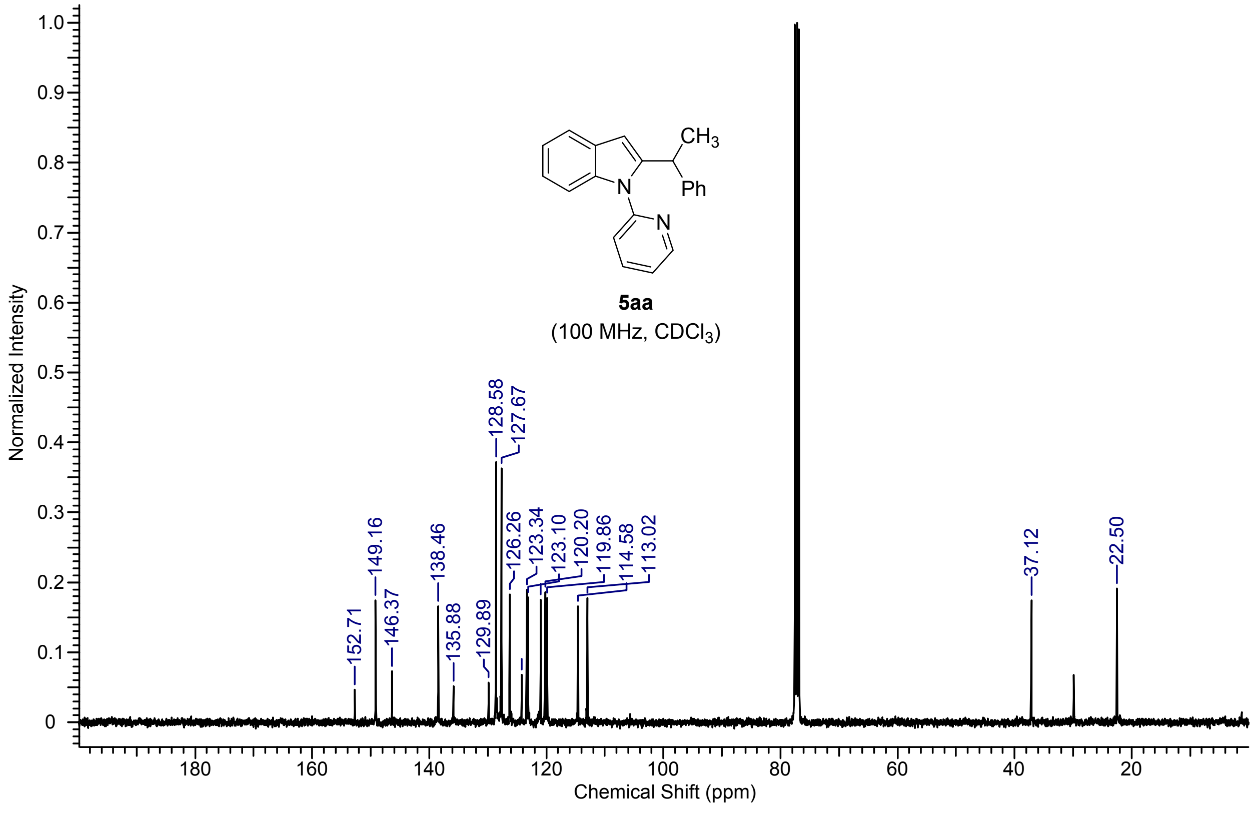

1.0
0.9
0.8
0.7
0.6
0.5
0.4
0.3
0.2
0.1
0
Normalized Intensity
CH3
Ph
N
N
5aa
(100 MHz, CDCl3)
152.71
149.16
146.37
138.46
135.88
129.89
128.58
127.67
126.26
123.34
123.10
120.20
119.86
114.58
113.02
37.12
22.50
180 160 140 120 100 80 60 40 20
Chemical Shift (ppm)

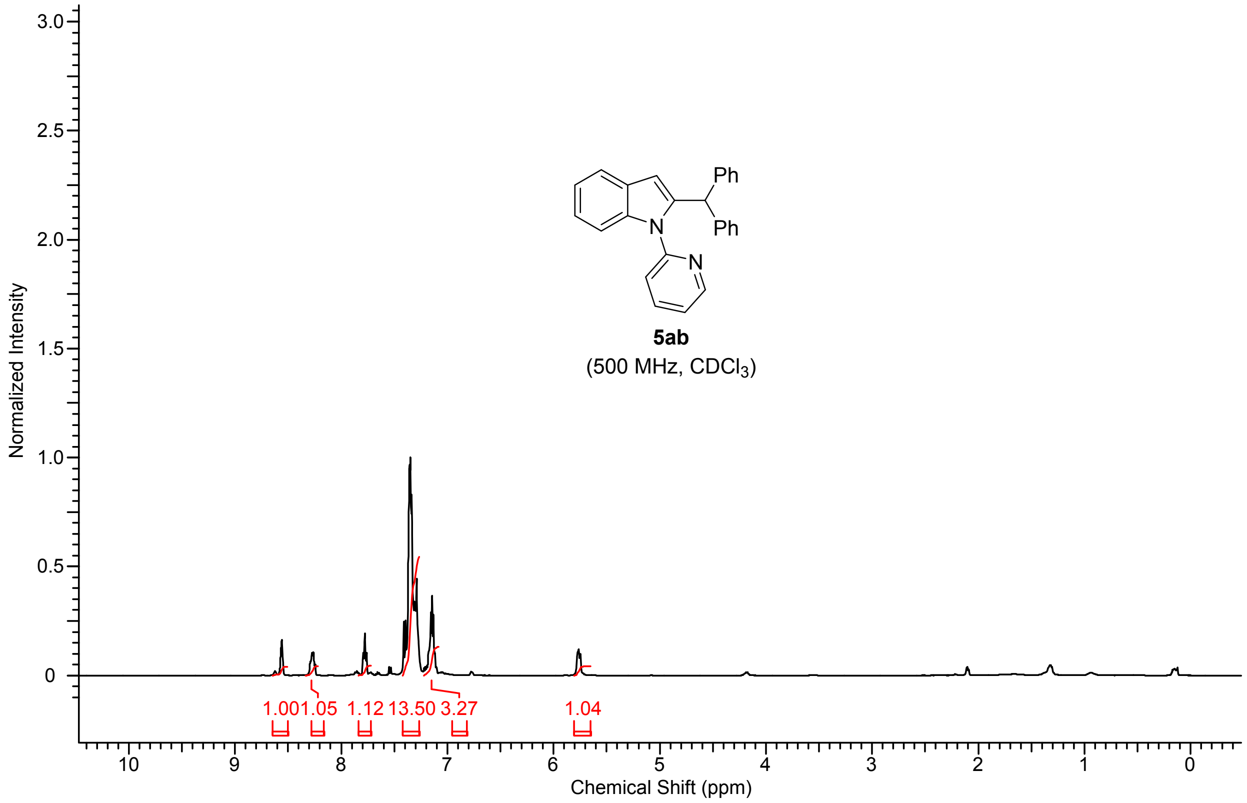
Ph
N
Ph
N
5ab
(500 MHz, CDCl3)
1.00
1.05
1.12
13.50
3.27
1.04
Normalized Intensity
Chemical Shift (ppm)

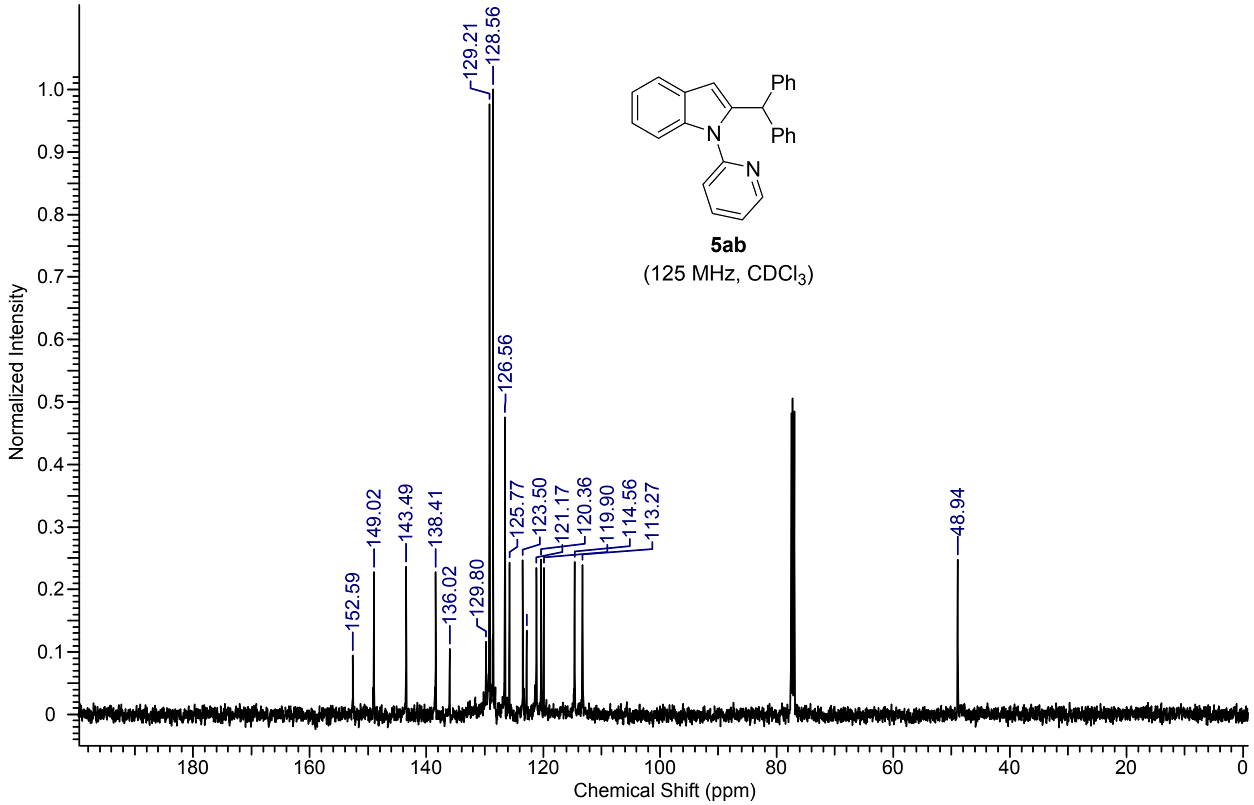
Ph
N
Ph
N
5ab
(125 MHz, CDCl3)
129.21
128.56
126.56
152.59
149.02
143.49
138.41
136.02
129.80
125.77
123.50
121.17
120.36
119.90
114.56
113.27
48.94
Normalized Intensity
Chemical Shift (ppm)

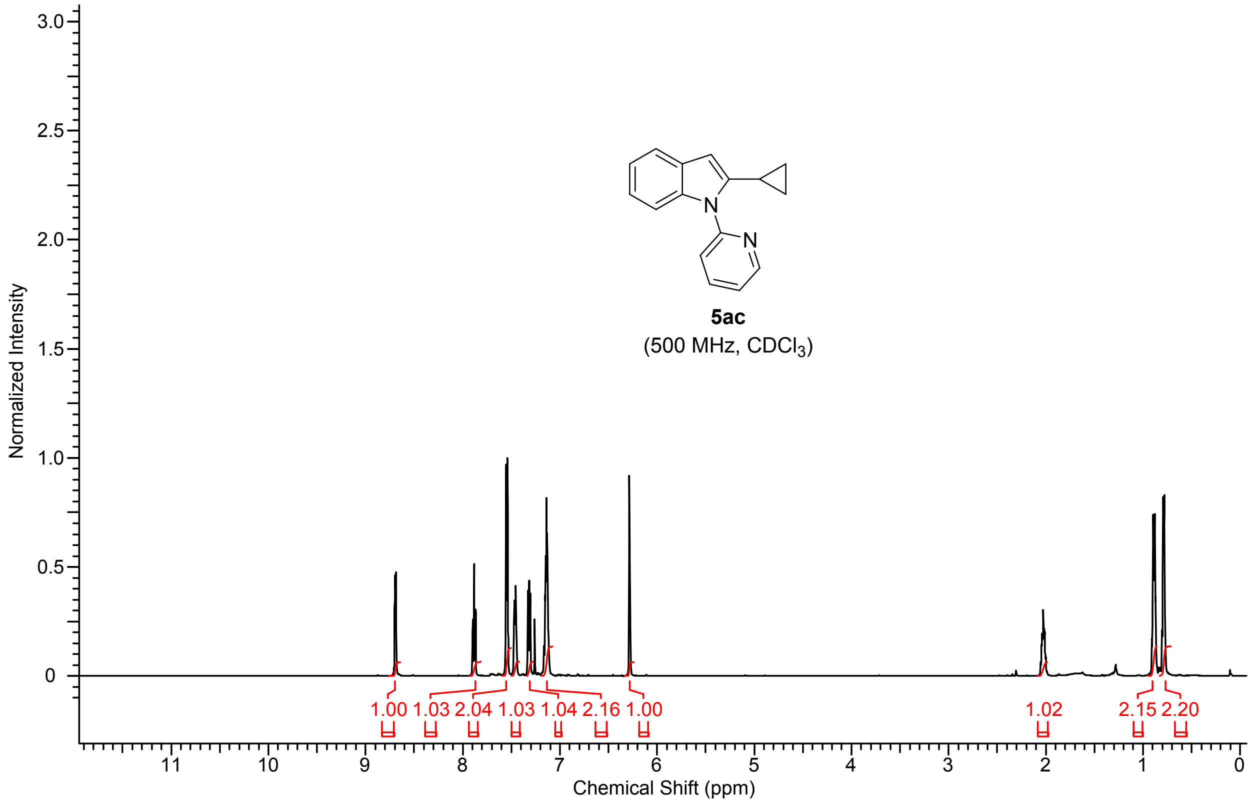
5ac
(500 MHz, CDCl3)
Normalized Intensity
Chemical Shift (ppm)
1.00 1.03 2.04 1.03 1.04 2.16 1.00
1.02
2.15 2.20

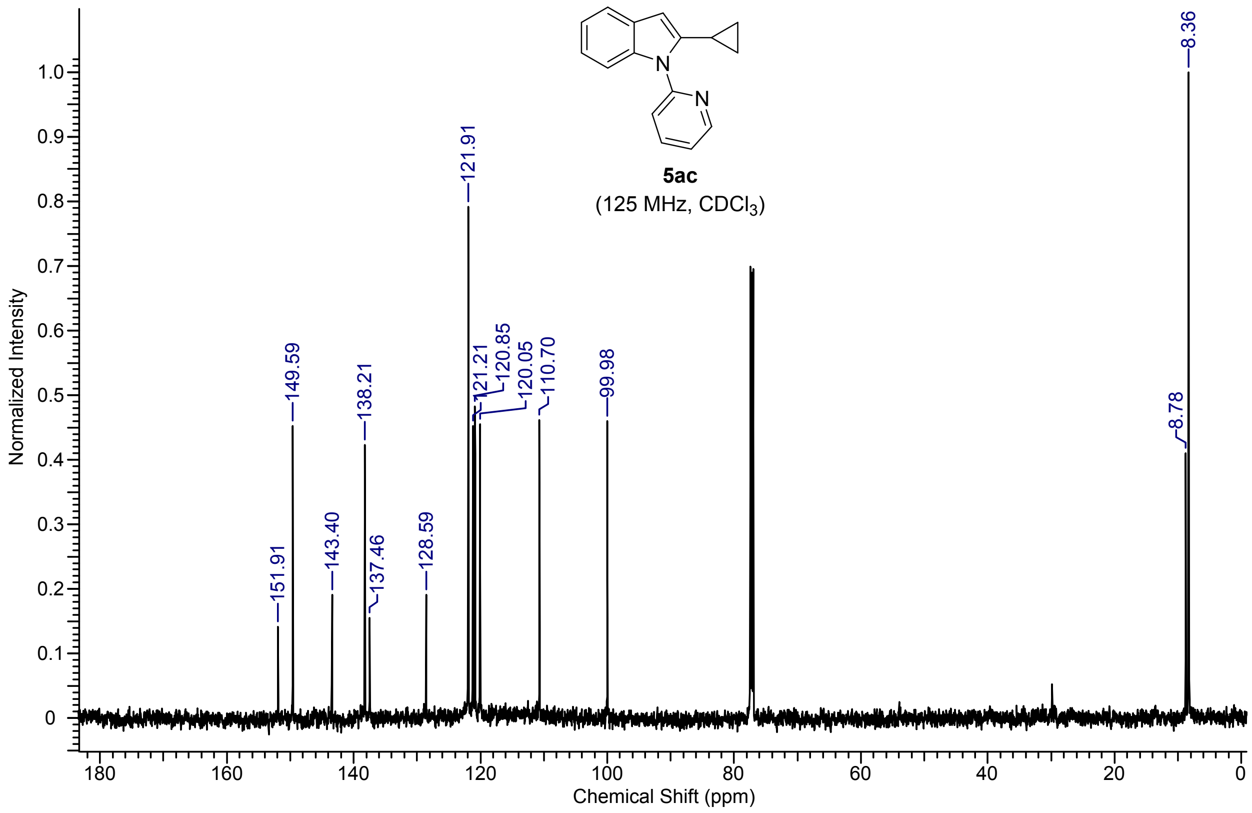
5ac
(125 MHz, CDCl3)
Normalized Intensity
Chemical Shift (ppm)
151.91
149.59
143.40
138.21
137.46
128.59
121.91
121.21
120.85
120.05
110.70
99.98
8.78
8.36

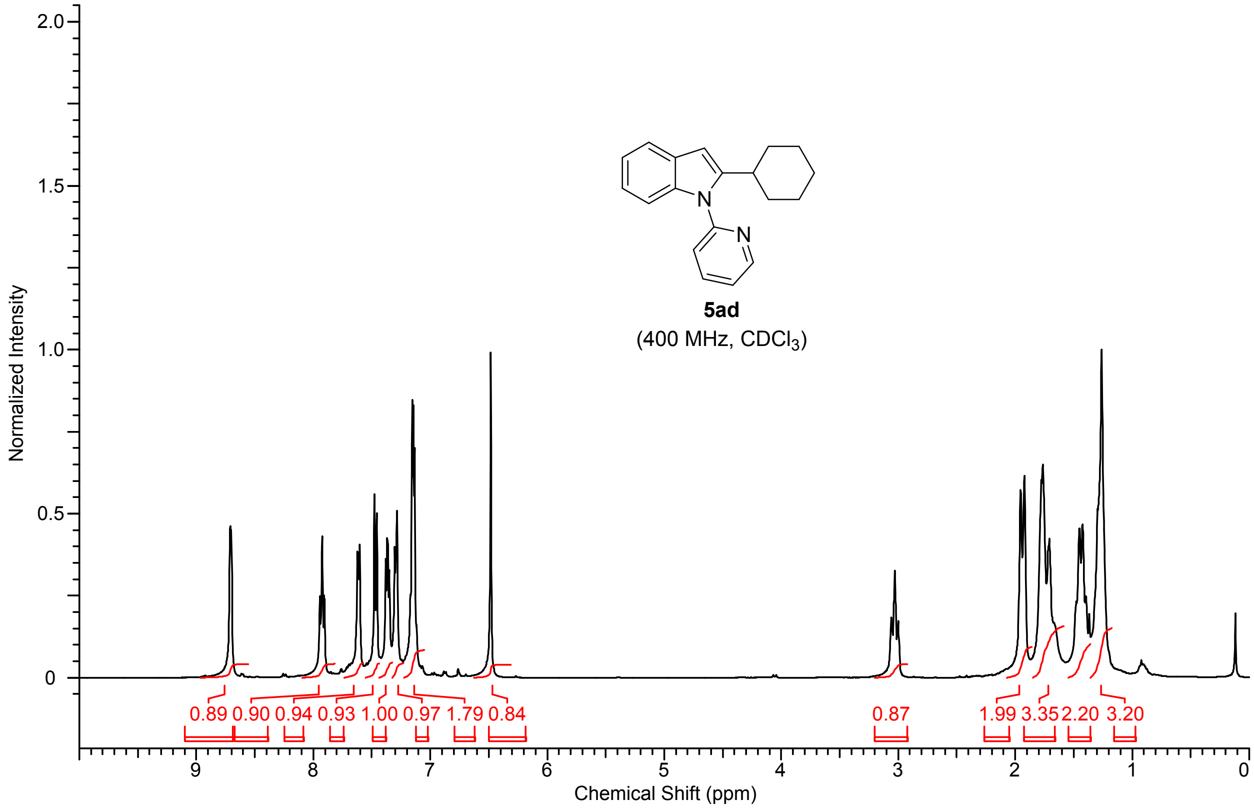
5ad
(400 MHz, CDCl3)
Normalized Intensity
Chemical Shift (ppm)
0.89 0.90 0.94 0.93 1.00 0.97 1.79 0.84
0.87
1.99 3.35 2.20 3.20

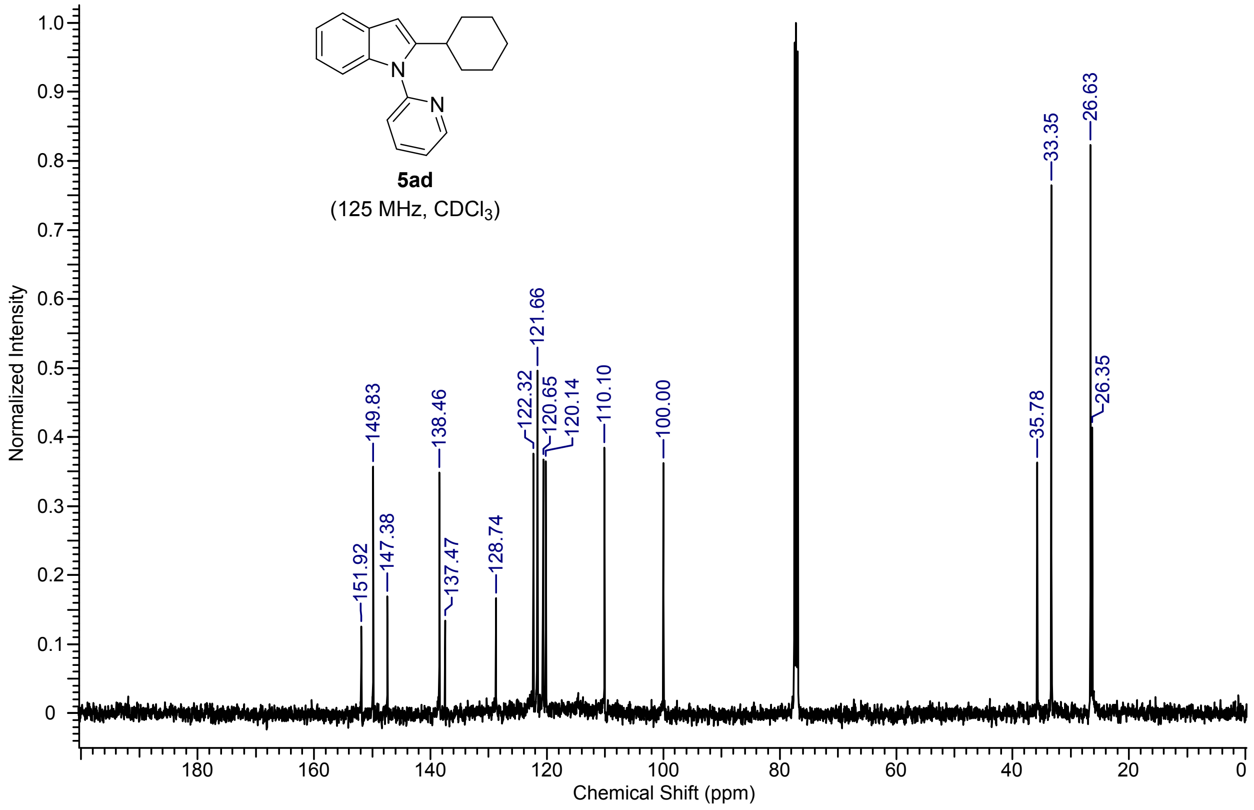
5ad
(125 MHz, CDCl3)
Normalized Intensity
Chemical Shift (ppm)
151.92
149.83
147.38
138.46
137.47
128.74
122.32
121.66
120.65
120.14
110.10
100.00
35.78
33.35
26.63
26.35

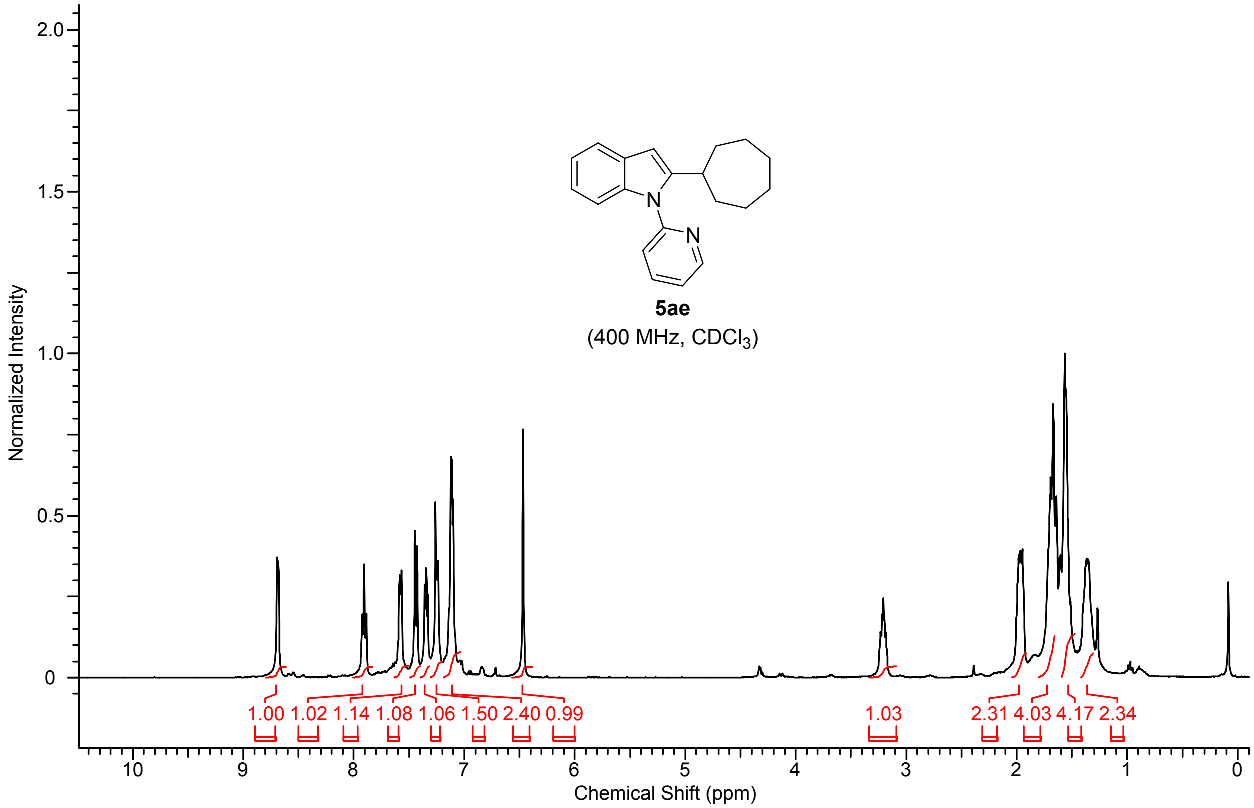

5ae
(400 MHz, CDCl3)
Normalized Intensity
Chemical Shift (ppm)
1.00 1.02 1.14 1.08 1.06 1.50 2.40 0.99
1.03
2.31 4.03 4.17 2.34

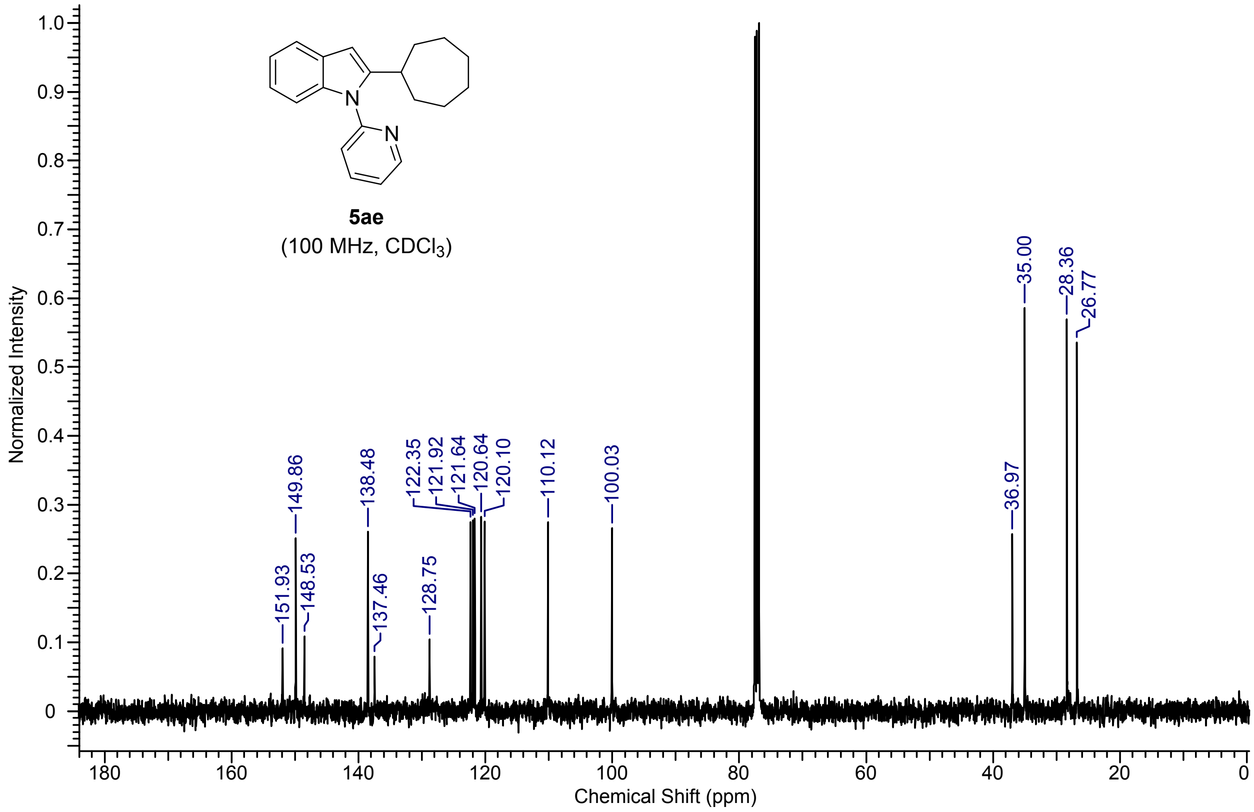

5ae
(100 MHz, CDCl3)
Normalized Intensity
Chemical Shift (ppm)
151.93
149.86
148.53
138.48
137.46
128.75
122.35
121.92
121.64
120.64
120.10
110.12
100.03
36.97
35.00
28.36
26.77

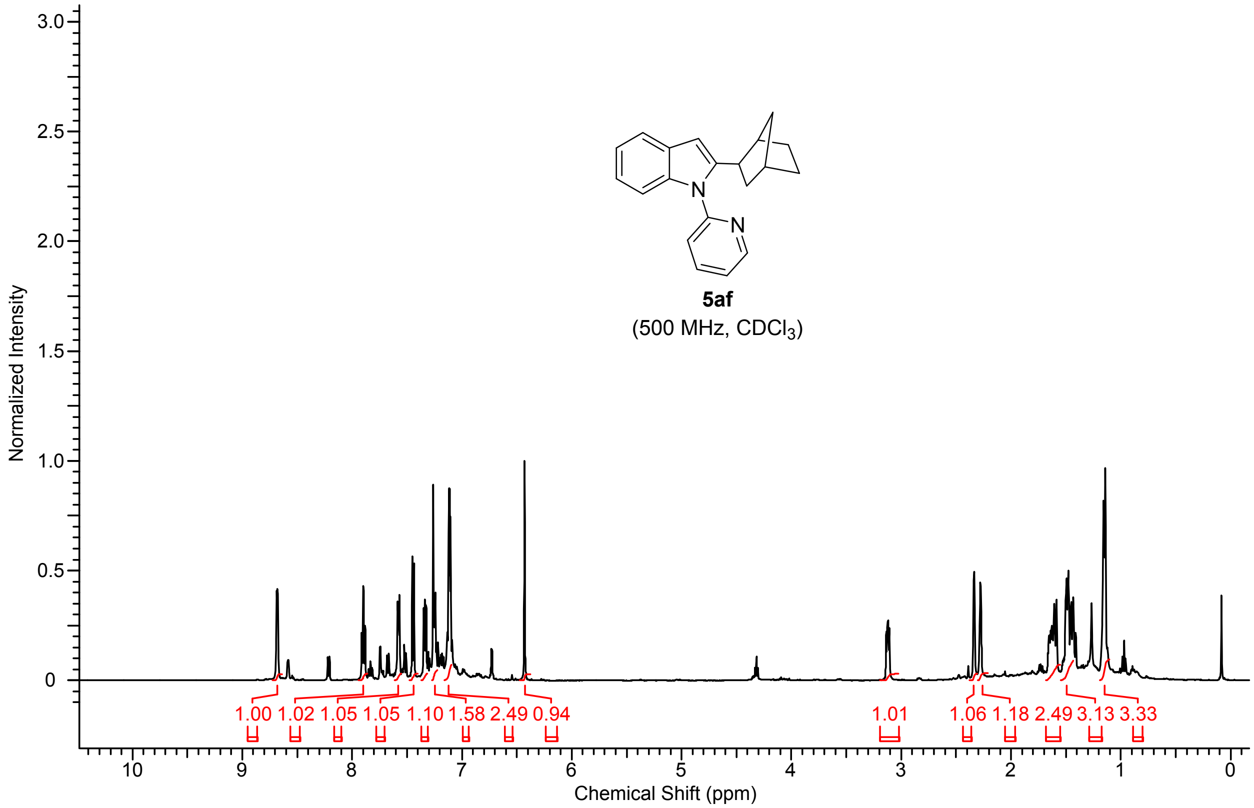

5af
(500 MHz, $CDCl_3$)
Normalized Intensity
Chemical Shift (ppm)
1.00 1.02 1.05 1.05 1.10 1.58 2.49 0.94
1.01
1.06 1.18 2.49 3.13 3.33

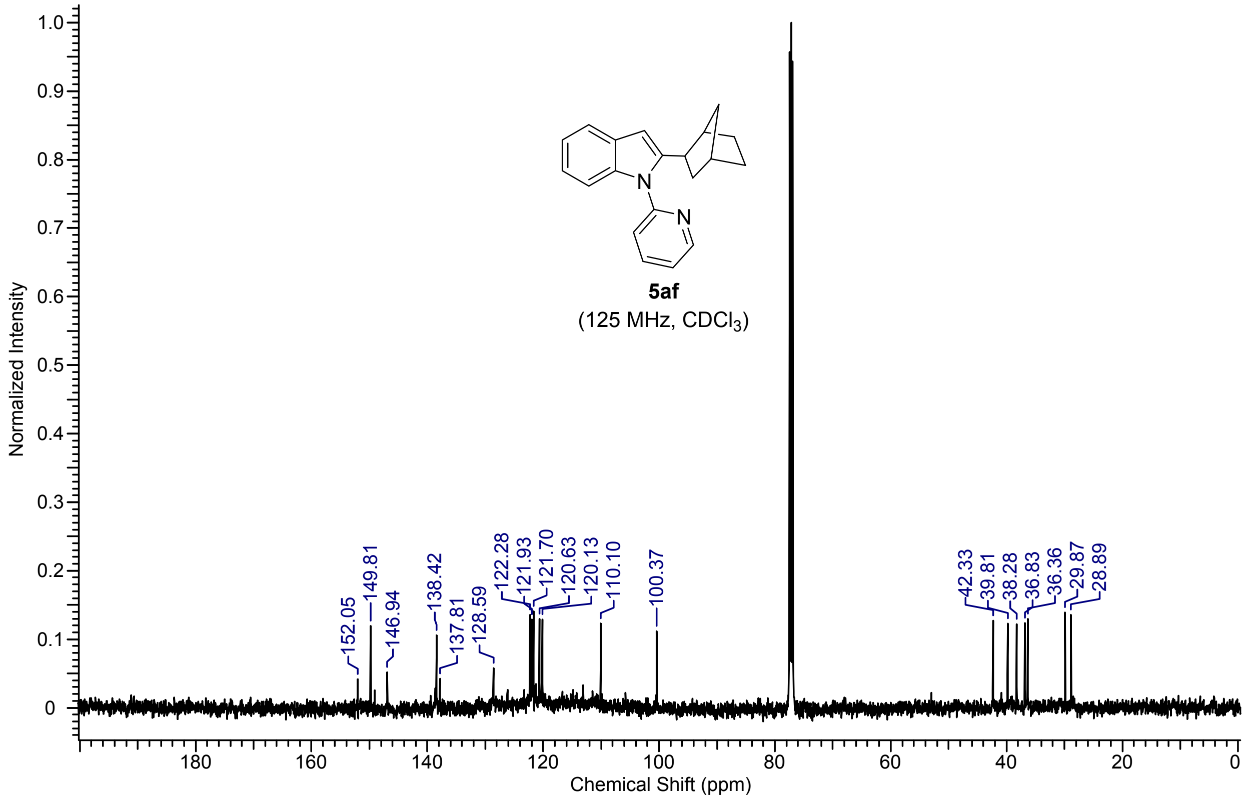

5af
(125 MHz, $CDCl_3$)
Normalized Intensity
Chemical Shift (ppm)
152.05
149.81
146.94
138.42
137.81
128.59
122.28
121.93
121.70
120.63
120.13
110.10
100.37
42.33
39.81
38.28
36.83
36.36
29.87
28.89

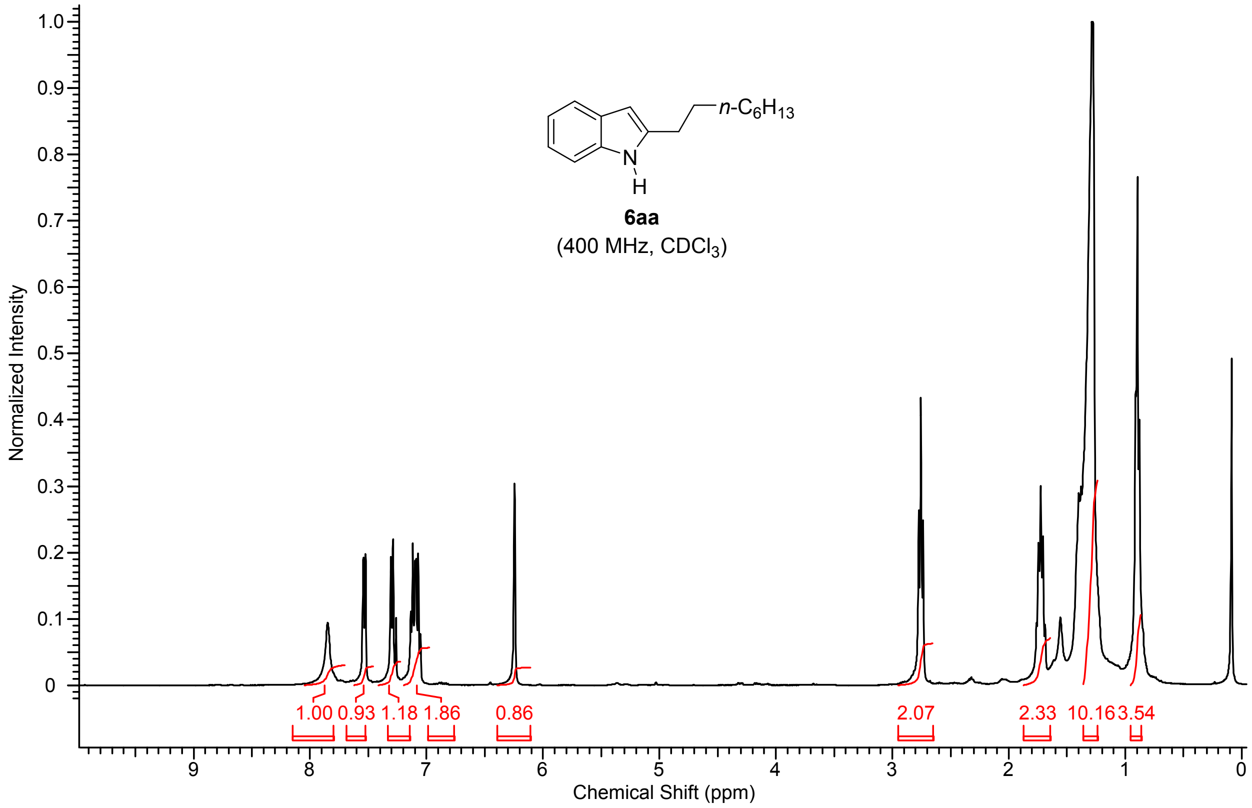
n-C6H13
N
H
6aa
(400 MHz, CDCl3)
Normalized Intensity
Chemical Shift (ppm)
1.00 0.93 1.18 1.86 0.86 2.07 2.33 10.16 3.54

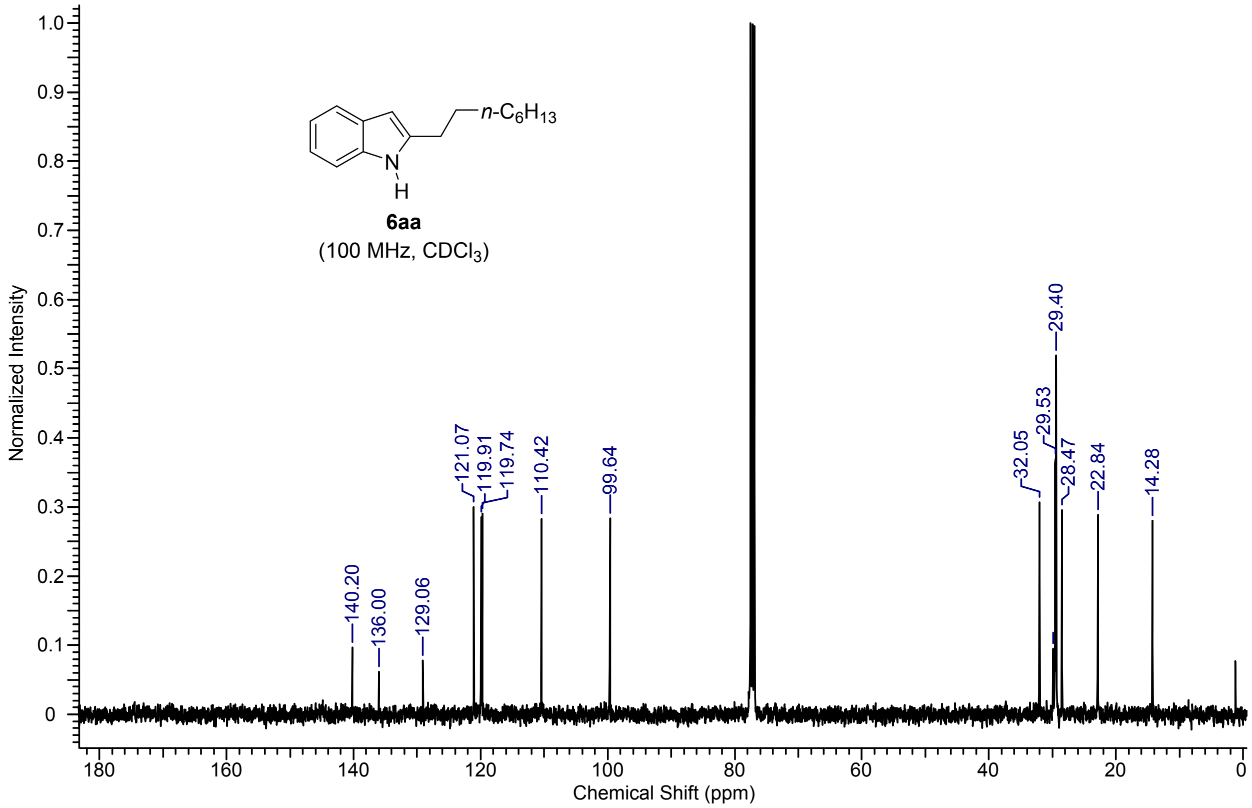
n-C6H13
N
H
6aa
(100 MHz, CDCl3)
Normalized Intensity
Chemical Shift (ppm)
140.20
136.00
129.06
121.07
119.91
119.74
110.42
99.64
32.05
29.53
29.40
28.47
22.84
14.28

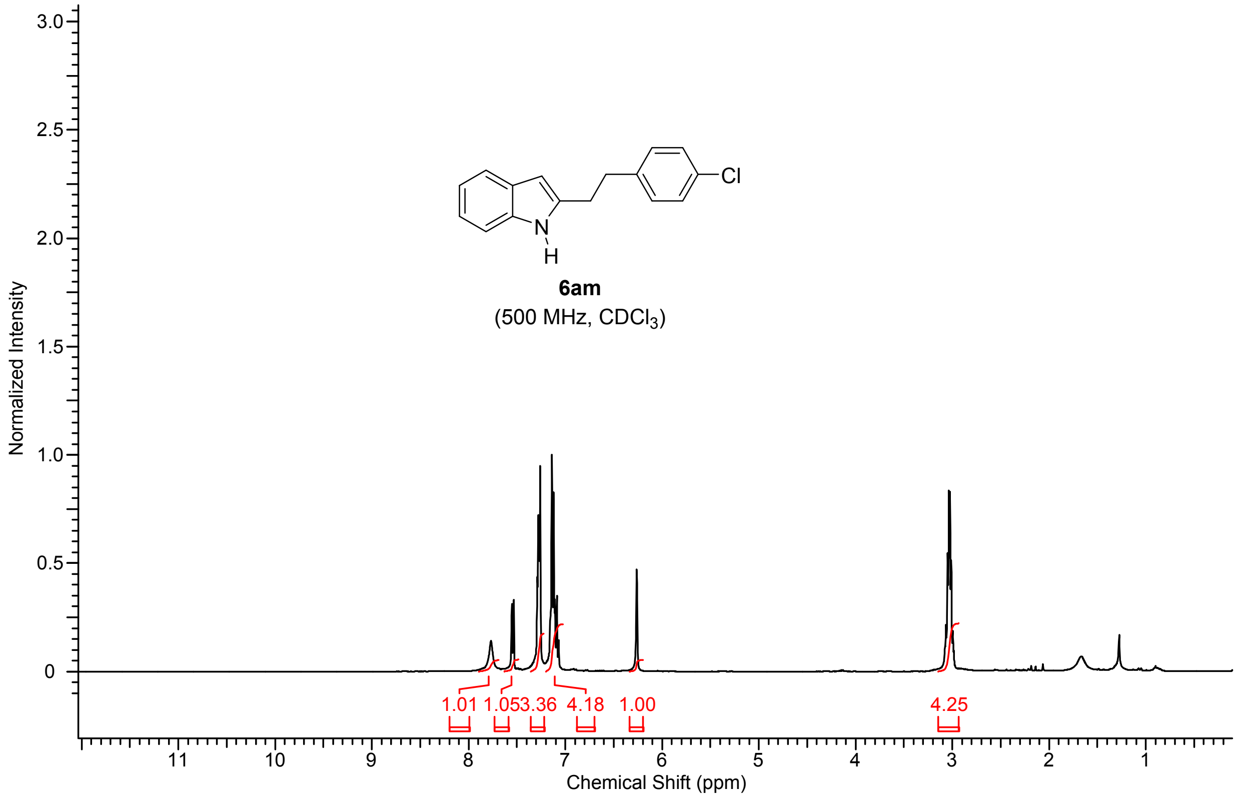
Cl
N
H
6am
(500 MHz, CDCl3)
Normalized Intensity
Chemical Shift (ppm)
1.01
1.05
3.36
4.18
1.00
4.25

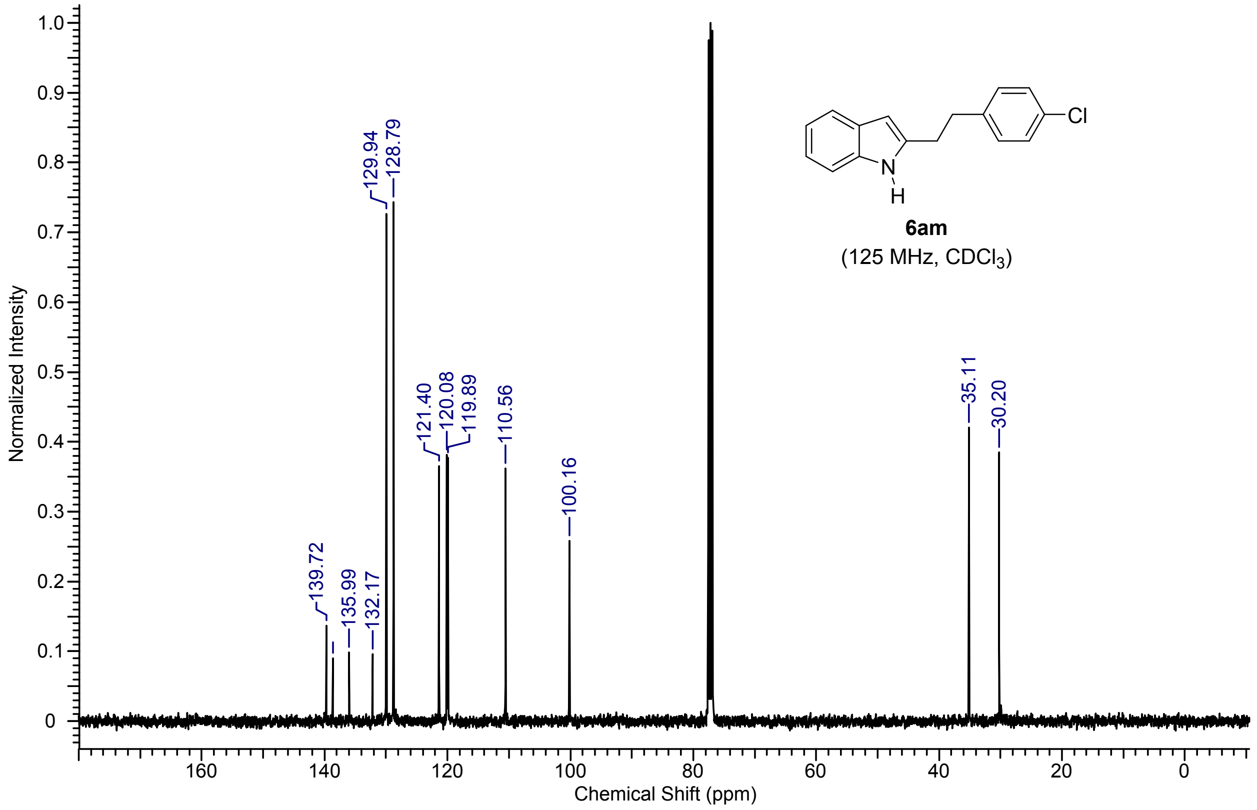
Cl
N
H
6am
(125 MHz, CDCl3)
Normalized Intensity
Chemical Shift (ppm)
139.72
135.99
132.17
129.94
128.79
121.40
120.08
119.89
110.56
100.16
35.11
30.20

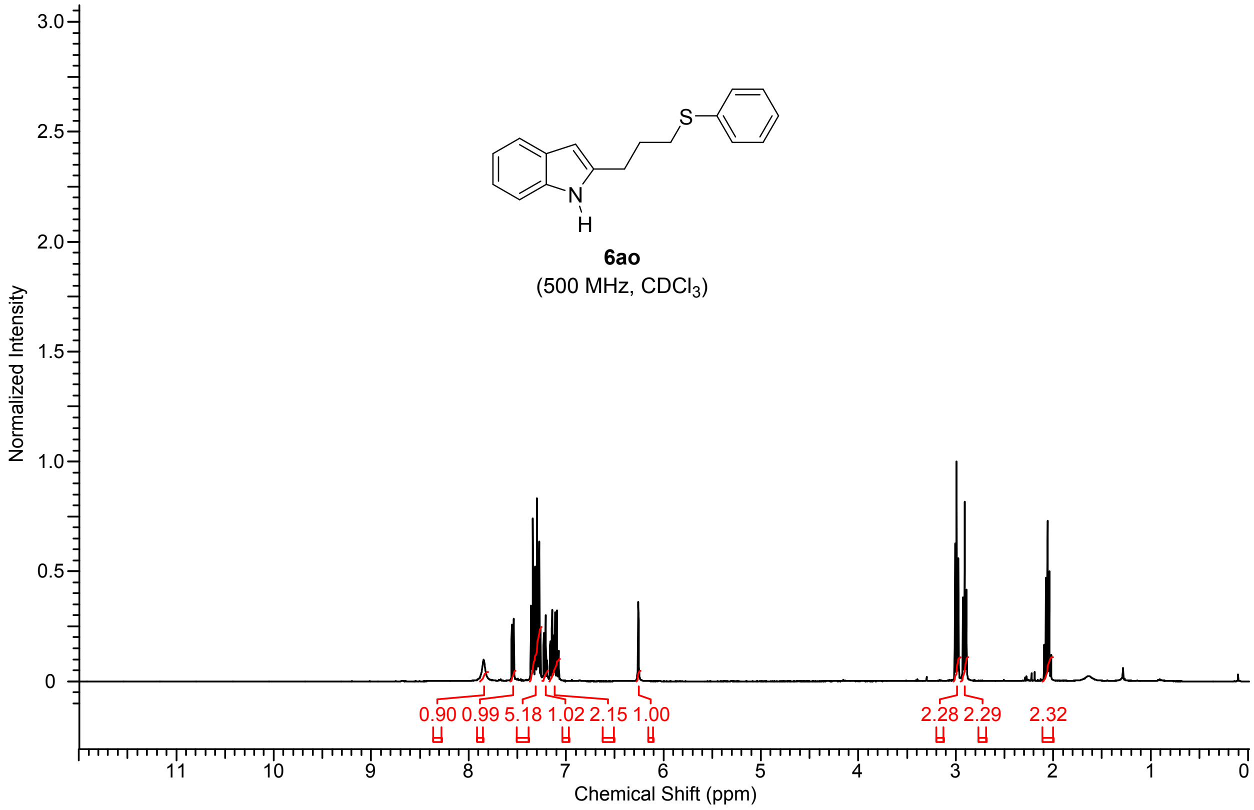

S
N
H
6ao
(500 MHz, CDCl3)
Normalized Intensity
0.90 0.99 5.18 1.02 2.15 1.00
2.28 2.29 2.32
Chemical Shift (ppm)

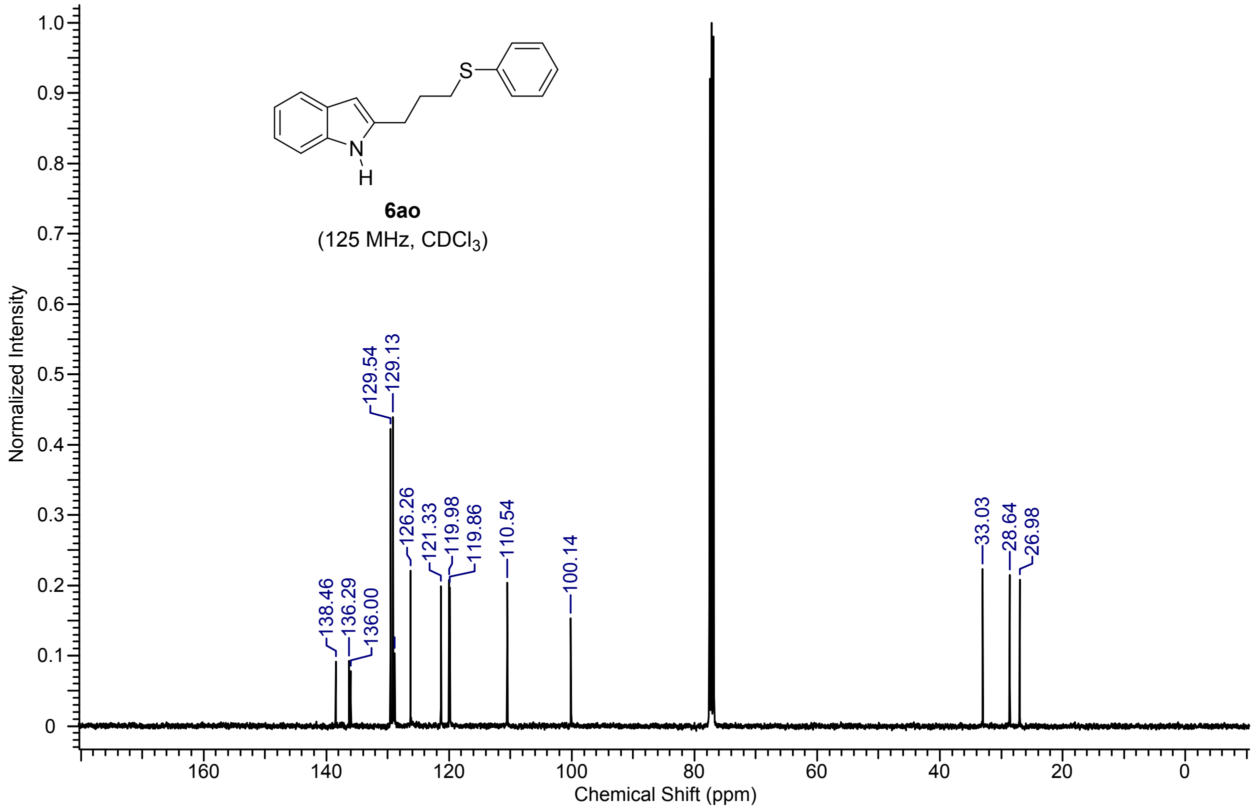

S
N
H
6ao
(125 MHz, CDCl3)
Normalized Intensity
138.46
136.29
136.00
129.54
129.13
126.26
121.33
119.98
119.86
110.54
100.14
33.03
28.64
26.98
Chemical Shift (ppm)
